# Replacement of nitro function by free boronic acid in non-steroidal anti-androgens†

**DOI:** 10.1039/d4md00343h

**Published:** 2024-09-10

**Authors:** Petr Šlechta, Roman Viták, Pavel Bárta, Kateřina Koucká, Monika Berková, Diana Žďárová, Andrea Petríková, Jiří Kuneš, Vladimír Kubíček, Martin Doležal, Radek Kučera, Marta Kučerová-Chlupáčová

**Affiliations:** a Department of Pharmaceutical Chemistry and Pharmaceutical Analysis, Faculty of Pharmacy in Hradec Králové, Charles University Ak. Heyrovského 1203/8 50003 Hradec Králové Czech Republic kucerom@faf.cuni.cz; b Department of Pharmacology and Toxicology, Faculty of Medicine in Pilsen, Charles University Alej Svobody 1655/76 32300 Plzeň Czech Republic; c Department of Biophysics and Physical Chemistry, Faculty of Pharmacy in Hradec Králové, Charles University Ak. Heyrovského 1203/8 50003 Hradec Králové Czech Republic; d Department of Organic and Bioorganic Chemistry, Faculty of Pharmacy in Hradec Králové, Charles University Ak. Heyrovského 1203/8 50003 Hradec Králové Czech Republic

## Abstract

A new series of potential flutamide-like antiandrogens has been designed and synthesized to treat prostate cancer. This new series results from our research, which has been aimed at discovering new compounds that can be used for androgen deprivation treatment. The antiandrogens were designed and synthesized by varying the acyl part, linker, and substitution of the benzene ring in the 4-nitro-3-trifluoromethylanilide scaffold of non-steroidal androgens. In addition, the characteristic feature of the nitro group was replaced by a boronic acid functionality. Compound 9a was found to be more effective against LAPC-4 than the standard antiandrogens flutamide, hydroxyflutamide, and bicalutamide. Moreover, it exhibited lower toxicity against the non-cancerous cell line HK-2. The initial *in silico* study did not show evidence of covalent bonding to the androgen receptor, which was confirmed by an NMR binding experiment with arginine methyl ester. The structure–activity relationships discovered in this study could provide directions for further research on non-steroidal antiandrogens.

## Abstract 2

A screen of 33 boronic acids identified molecules with antiproliferative activity against androgen-dependent prostate cancer cell line LAPC4. Structural similarity with NSAA raises a question whether nitro group could be substituted for boronic acid.

## Graphical abstract

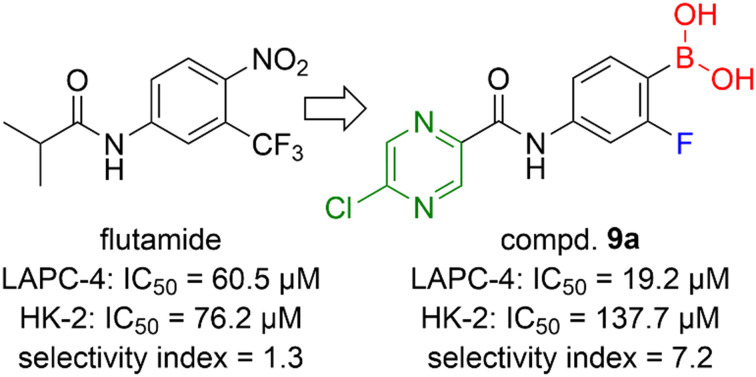

## Introduction

Prostate cancer (PC) is one of the most deadly cancers and one of the most common cancers among men worldwide.^1^ The prognosis and outcome of therapy depend on several factors, such as progression grade, age, or ethnicity. The current treatments for PC include androgen deprivation, surgery, radiotherapy, chemotherapy, and immunotherapy.^2^

**Fig. 1:**
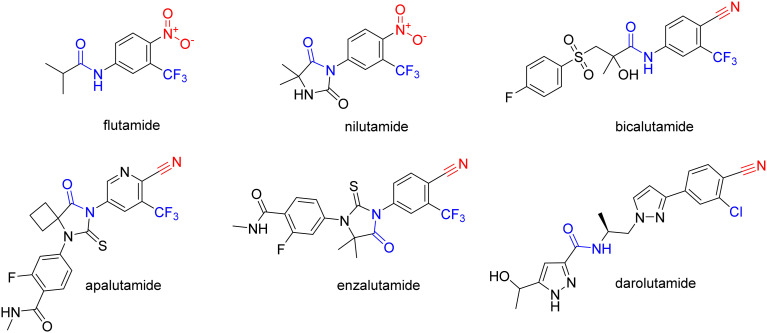
Structures of FDA-approved NSAA with highlighted common structural features.

PC growth relies heavily on androgens and the activation of the androgen receptor (AR).^3^ Androgen deprivation can be achieved by inhibiting androgen biosynthesis with abiraterone-acetate, or by using AR antagonists, such as cyproterone or non-steroidal antiandrogens (NSAAs). NSAAs, such as flutamide, nilutamide, bicalutamide, apalutamide, enzalutamide, and darolutamide, act as AR antagonists and either have been or are currently used to treat PC (Fig. 1).^2^

NSAAs have a common structural feature that can be defined as a 3-trifluoromethyl anilide bearing either a nitro or cyano group in position 4 to the anilide group (as shown in Fig. 1). The importance of this structural pharmacophore for retaining antagonistic activity towards the AR is highlighted by the fact that it appears not only in the structures of all the first FDA-approved NSAA derivatives, such as flutamide but also in modern derivatives, such as enzalutamide. The most significant difference among the generations of NSAAs is that the first candidates contained a nitro function, while later NSAAs mostly continued using a nitrile substitution. The modern derivatives are furthermore distinguishable by a bulkier substitution in the acyl part of the structure. The nitro/nitrile functionality is crucial in the hydrogen-bond (H-bond) interaction with Arg752 within the AR binding pocket.^4^ Flutamide can cause hepatic injury or even liver failure. Its liver toxicity is caused probably by one of its metabolites *N*-hydroxy-4-nitro-3-(trifluoromethyl)aniline^5^ or generally by the nitro group in the benzene ring.^6–8^ In this work, we suggested substituting the nitro group for other more specific functional groups that could form an analogical, or even covalent, interaction with Arg752 within the binding site of the AR. We chose to use the boronic acid functionality because it can form coordinate covalent interactions with nucleophilic structures within biological systems.^9^ These interactions are typically formed with the hydroxy group of the serine residue, but some studies have shown that intramolecular N–B bond formation is also possible.^10–12^ In addition, the change from nitro group to boronic acid can modify the binding capabilities and give rise to hydrogen bond formation.

Generally, boronic acids have gained popularity in medicinal chemistry since the first boron-containing anticancer drugs were registered, *e.g.*, bortezomib.^13–16^ Derivatives of boronic acids can potentially serve as inhibitors of the proteasome, tyrosine kinase inhibitors, tubulin binders, fibroblast activation protein inhibitors, histone deacetylase inhibitors, dipeptidyl peptidase IV inhibitors, or some other protease inhibitors in anticancer therapy.^17–21^ Boronic acid functionality is mostly used as an isostere of carboxylic acid, phosphate, or phenolic hydroxyl.^20,22,23^ To the best of our knowledge, boronic acid has not yet been used as a bioisostere of the nitro group. To investigate whether the mentioned arginine residue would interact in a covalent way, we conducted a series of molecular docking experiments *in silico*. We then synthesized a library of simple flutamide-like compounds and tested them against a panel of cancer cell lines (LAPC-4, PC-3, and Hep G2) and non-cancerous cells (HK-2) to determine their potential activity and selectivity towards PC cell lines. We compared their effectiveness with that of standard drugs: flutamide, its active metabolite hydroxyflutamide (HF), and bicalutamide.

## Results and discussion

### Design of compounds and *in silico* experiment

The presented compounds were designed in order to investigate the ability of boronic acid to bind to the AR and to explore the possibility of replacing the nitro function with a bioisosteric boronic acid. The compounds were inspired by the first and simplest NSAA, flutamide. The replacement was expected to have a direct effect due to the small number of structural moieties.

**Fig. 2:**
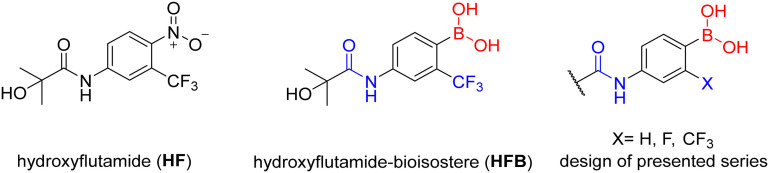
Structure of hydroxyflutamide (HF), hydroxyflutamide-bioisostere (HFB), and design of presented series.

To begin with, three primary structural moieties were used to build the series 1a–20a, 1b–7b, and 1c–5c (structures can be found in ESI† Tables S1–S3). These three moieties include an aromatic core that is substituted with an amide group (an anilide), boronic acid was used in the position of the former nitro function, and a trifluoromethyl substitution was placed in the *ortho* position to the boronic acid (as shown in Fig. 2).

In order to gain a deeper understanding of how each structural component functions when bound to the binding site of the AR, a study was conducted on the available experimental structures of NSAAs bound to the ligand binding domain (LBD) of the AR, using data from the Protein Data Bank (PDB).

It is necessary for flutamide to be metabolized to HF prior to its interaction with the AR so that it can bind to the LBD of the AR effectively. However, PDB does not contain a crystallographic structure for HF bound to the native wild-type (WT) AR.^24^ Only crystallographic structures of HF bound to the resistant AR LBD T877A mutant (PDB ID: 2AX6), or a close bromine derivative of HF bound to sensitive WT AR LBD (PDB ID: 2AX9) are available. It is important to note that the mutation in T877 causes agonist activity of HF, and the close bromine derivative already elicits agonist activity at high concentrations (*i.e.* >100 nM), so both crystallographic structures represent a rather activated conformation of AR LBD.^24^ Despite this, these structures demonstrate the most likely binding mode of HF (and other close derivatives) to the WT AR LBD.^24^ Based on the crystallographic structures, it can be concluded that the amide nitrogen plays an important role in forming an H-bond interaction with the backbone oxygen of Leu704. Additionally, the hydroxy group is positioned within the distance required for H-bonds with Asn705 and the backbone oxygen of Leu704. The nitro group forms an H-bond with Arg752 and Gln711 directly and through a water molecule, which is similar to the binding pattern observed for the cyano group of bicalutamide. The cyano group of bicalutamide also forms an H-bond with Arg752 but is too distant to interact directly with Gln711 (PDB ID: 1Z95).^24^

**Fig. 3:**
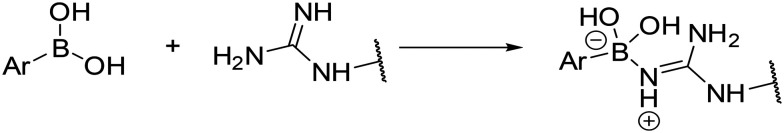
Theoretical formation of B–N coordinate covalent bond between boronic acid and arginine residue.

**Fig. 4:**
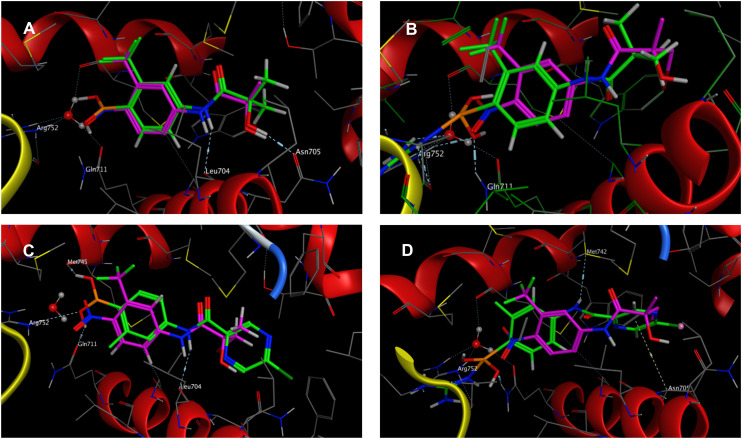
Results of an *in silico* experiment using the structure of WT AR LBD co-crystallized with (*R*)-3-bromo-2-hydroxy-2-methyl-*N*-[4-nitro-3-(trifluoromethyl)phenyl]propanamide (purple colour) as a ligand (PDB ID: 2AX9). A) Results of general (non-covalent) docking of HFB (green colour) in 2AX9. B) Results of covalent docking of HFB (green colour) in 2AX9. C) Results of general (non-covalent) docking of compound 9a (green colour) in 2AX9. D) Results of covalent docking of compound 9a (green colour) in 2AX9.

An *in silico* experiment was conducted to check if the intended boronic compounds could fit in the WT AR LBD. The boronic bioisostere of hydroxyflutamide (HFB, Fig. 2) was used in this experiment, and it was docked to the binding site of WT AR LBD (PDB ID: 2AX9). Two types of docking variations were performed to test the interaction of the boronic acid with the arginine residue. The first docking method was the classical non-covalent binding between the ligand and the target. Subsequently, the HFB was docked covalently, based on the hypothesis that a coordinate covalent interaction would be formed between Arg752 and boronic acid (Fig. 3). The boronic acid has a free vacant p orbital which can accept a free electron pair from nucleophilic nitrogen of the guanidine function. After accepting the electron pair, boronic acid changes its hybridization from sp^2^ to sp^3^ and gains a negative charge. The docking results and calculated interactions were compared with the crystallographically obtained binding poses of HF-like compounds in the WT AR LBD (Fig. 4).

It can be concluded from the findings of the general (non-covalent) docking (Fig. 4A) that HFB forms multiple crucial interactions. As previously described, the nitrogen atom of the anilide group forms an H-bond with the carbonyl oxygen of Leu704, and the hydroxy group creates an H-bond with the carbonyl oxygen of the Asn705 residue. Additionally, the trifluoromethyl group points towards the lipophilic cavity of the binding site. The boronic acid function is located in the same position as the previous nitro group, and it is close enough to Arg752 and Gln711 to form hydrogen bonds (up to 3.3 Å). These findings suggest that the boronic acid may bind in the same way as the nitro group and potentially act as an antagonist of the AR. However, we obtained different results when covalent bonding was used (Fig. 4B). The covalent bond between the nitrogen of Arg752 and the boron atom of the boronic acid caused the molecule to shift towards the arginine direction, which prevented hydrogen bond formation between nitrogen and Leu704 and disrupted the interaction between the hydroxy group and Asn705. On the other hand, the covalent bond formation seemed to offer additional and closer H-bond stabilization with Arg752 and Gln711, which could be beneficial. This potential shift of boronic acid derivatives can cause steric hindrance between the trifluoromethyl group and the binding site of the receptor. To address this issue, a new set of derivatives without a bulky trifluoromethyl substitution was included in the study. However, reducing the lipophilic substitution in the *ortho* position resulted in a significant loss in possible binding affinity with the AR. To compensate for this, large aromatic substitutions were made in the acyl parts. In some cases, this large aromatic substitution further stabilized the binding between the ligand and the AR. For example, if the compound is substituted with 5-chloropyrazinoyl (compound 9a), it should be further stabilized by electrostatic interaction between the pyrazine core and Asn705.^25^ However, in this case, the nitrogen from the amide group will be stabilized by H-bond formation with Met742 (Fig. 4C and D).

### Synthesis of the chemical library

All compounds were prepared using up to three basic synthetic steps: acylation of aniline derivatives, deprotection of pinacol ester, and borylation. Borylation was used only in the synthesis of trifluoromethyl substituted derivatives for the preparation of 4-(4,4,5,5-tetramethyl-1,3,2-dioxaborolan-2-yl)-3-(trifluoromethyl) aniline. This was done to create the starting material for acylation.

**Fig. 5:**
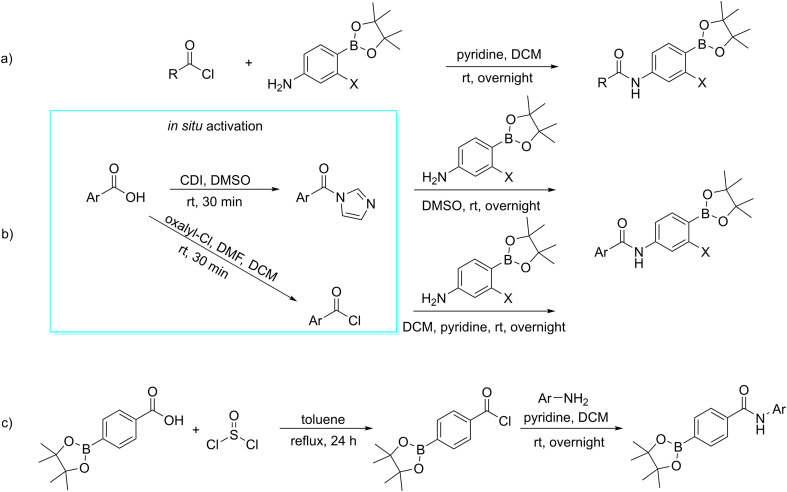
Acylation approaches used for obtaining of a) aliphatic and alicyclic carboxamides, b) (hetero)aromatic carboxamides, c) the borylated benzamide, X = H, F, CF_3_.

The synthetic pathways employed in the reaction for this study made use of acylation. The acylation strategies used are shown in Fig. 5.^26,27^ The amides of aliphatic and alicyclic acids were created by reacting commercially available acyl chlorides with the corresponding aniline dissolved in dichloromethane (DCM) as a solvent and a small excess of pyridine as a base (Fig. 5a). The aromatic and heteroaromatic derivatives, on the other hand, were prepared using the corresponding aryl or heteroaryl carboxylic acids activated *in situ* by 1,1′-carbonyldiimidazole (CDI) and then coupled with proper aniline in one pot, using dimethylsulfoxide (DMSO) as a solvent. The resulting product was isolated by adding water to the reaction mixture, followed by precipitation. In cases of benzoic acids resistant to CDI-activation, they were activated *in situ* to acyl chloride using oxalyl chloride. The carboxylic acid was dissolved in DCM with a small excess of oxalyl chloride, and then a catalytic amount of dimethylformamide (DMF) was added to speed up the activation step. After completing the activation process, the solution of activated acyl chloride was added dropwise to the solution of the corresponding aniline that had been dissolved in DCM and pyridine while the solution was being cooled in an ice bath. Once the reaction was complete, it was kept at room temperature (Fig. 5b). This activation step proved to be very useful for coupling of 4-chlorobenzoic acid, but it was not effective enough for activation of borylated benzoic acid in the synthesis of derivative with a reversed amide bond. In the latter case, carboxylic acid was activated by thionyl chloride in toluene. After the activation was complete, the solvent was evaporated, and the crude acyl chloride was then used for the coupling reaction in DCM and pyridine (Fig. 5c).

Due to the poor stability and described reactivity of free boronic acids,^28,29^ the chromatographic purification was done only for pinacol-protected intermediates. Regardless of the acylation strategy or workup procedure followed by the coupling reaction, the intermediates were always purified using flash chromatography.

**Fig. 6:**
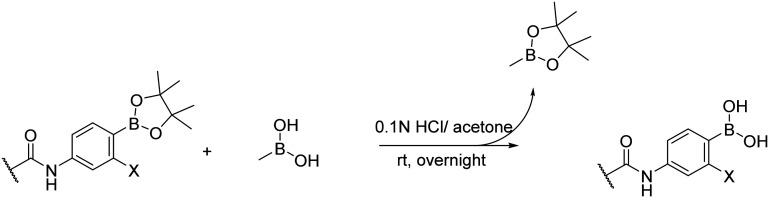
Transesterification of pinacol-protected intermediates using methyl boronic acid.

The transesterification method described by Hinkes *et al.*^30^ was used to remove the pinacol ester protection. This method is straightforward and effective. First, the pinacol-protected intermediate was mixed with an excess of methyl boronic acid and dissolved in the appropriate solvent. The transesterification process was driven by adding the proper amount of acid or base. Once the transesterification was completed, the byproduct methyl boronic pinacol ester and any residues of methyl boronic acid were easily evaporated with the solvent due to their high volatility. Moreover, the higher volatility of methyl boronic pinacol ester drives the reaction to completion during the evaporation process.^31^ During the transesterification process, we experimented with different conditions to optimize the reaction. When we added a 5% solution of TFA in DCM along with methyl boronic acid, our compounds decomposed. However, using milder conditions such as 10 equivalents of methyl boronic acid in 0.1 N hydrochloric acid solution in water with acetone in a 1 : 1 ratio (v/v) produced the deprotected final compounds in sufficient yield and purity (Fig. 6). Some of our compounds contained basic nitrogen within the heterocycle, which made them prone to create salt with heavily evaporable hydrochloric acid. Fortunately, this salt was very unstable due to the partnership between a strong acid and a weak base, so we hydrolyzed it by suspending the solid residue in water and filtering it. In some cases, we observed the formation of heteroboroxines during the deprotection,^30,32^ but this was a reversible process that we easily eliminated by dissolving the sample multiple times in 0.1 N HCl water/acetone solution and evaporating it.

**Fig. 7:**
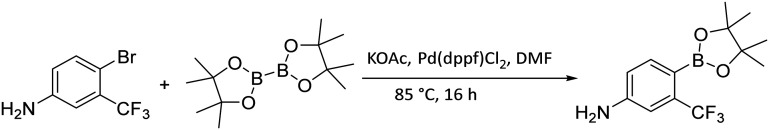
Borylation synthetic scheme.

In order to obtain the closest derivatives of flutamide, it was essential to borylate trifluoro phenyl aniline (Fig. 7). For this, we used Miyaura borylation. The starting materials, 4-bromo-3-(trifluoromethyl)aniline and bis(pinacolato)diboron (B_2_pin_2_), were mixed with potassium acetate (KOAc) in anhydrous DMF. As a catalyst ligand complex [1,1′-bis(diphenylphosphino)ferrocene]dichloropalladium(ii) (Pd(dppf)Cl_2_) was used. Due to the similarity in retention factors of the starting aniline and the borylated aniline, monitoring the reaction progress using TLC became difficult. To overcome this, we used the specific curcumin interaction with compounds containing boron for quick visualization of the borylation process on TLC plates.^33^ This technique was also used for monitoring the correct pinacol release during deprotection.

**Fig. 8:**
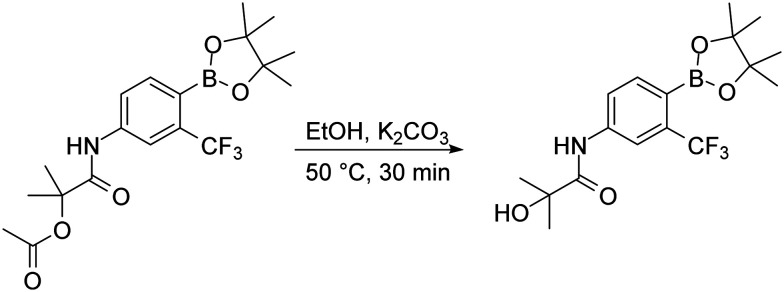
Basic hydrolysis of acetoxy group.

To complete the discussion about used synthetic steps in this work, we need to mention the basic hydrolysis of the acetoxy group of the HFB intermediate, which was necessary for the preparation of the HFB compound. The hydrolysis was made by dissolving the proper acetoxy intermediate in ethanol and heating it for 30 minutes with excess potassium carbonate. After the reaction was complete, simple work-up procedure consisting of potassium carbonate filtration was applied and purification with flash chromatography followed (Fig. 8).

**Fig. 9:**
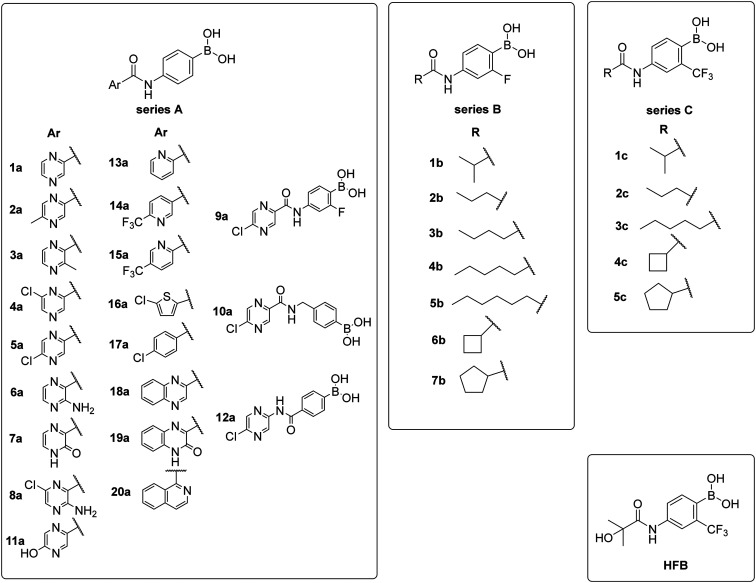
Library of final boronic acids.

In total, we prepared thirty-three final boronic acids HFB, 1a–20a, 1b–7b, and 1c–5c (Fig. 9). The free boronic acids were isolated as solids and they were characterized using melting points, ^1^H-, ^13^C-, and ^11^B-NMR spectra, as well as IR spectra. The purity of non-fluorinated compounds was checked through elemental analysis, whereas for fluorine-contained compounds, HPLC was used. In the ^13^C-NMR spectra, the carbon that is directly attached to the boronic acid was usually not observed due to the quadrupolar relaxation of ^10^B and ^11^B nuclei.^30^

### ^11^B- and ^19^F-NMR experiments

**Fig. 10:**
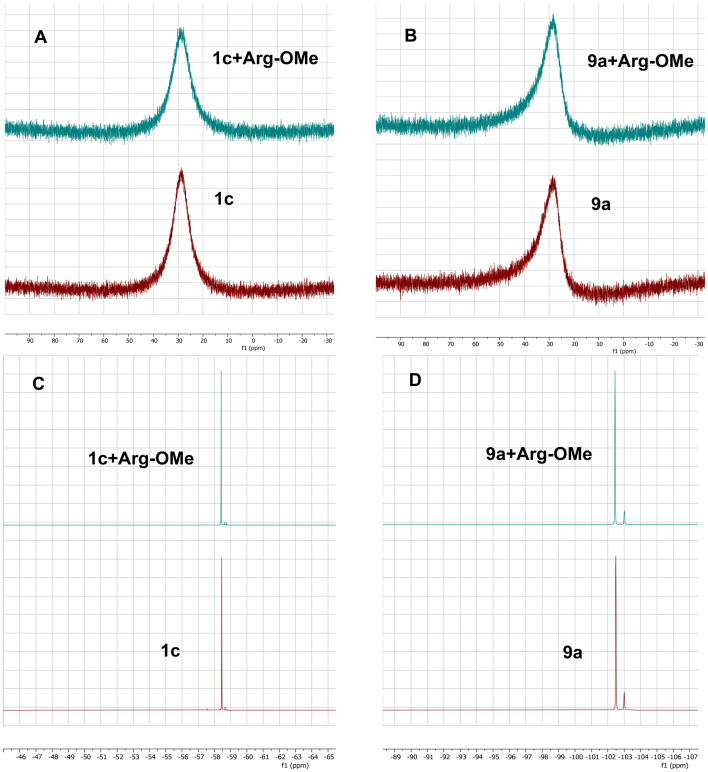
Results of NMR experiments. A) ^11^B-NMR spectrum of compound 1c with and without the **Arg-OMe**. B) ^11^B-NMR spectrum of compound 9a with and without the **Arg-OMe**. C) ^19^F-NMR spectrum of compound 1c with and without the **Arg-OMe**. D) ^19^F-NMR spectrum of compound 9a with and without the **Arg-OMe**.

In order to test whether the presented compounds could form a covalent N–B bond with the Arg752 residue, a simple NMR experiment was conducted. Previous research has explored intramolecular N–B bond formation and its verification using NMR.^34–36^ If the presented compounds could indeed form an N–B covalent bond with the Arg752 residue, there should also be an observable covalent bond formation between the l-arginine and the presented compounds. To avoid any possible covalent coordination between the oxygen of the carboxylic acid from l-arginine, the l-arginine methyl ester (Arg-OMe) was prepared and used as a model compound to simulate the behavior of the Arg752 residue within the WT AR LBD. The compounds 1c and 9a were chosen for the experiment, with 1c being the closest flutamide bioisostere and 9a being the most active compound. Firstly, the ^11^B- and ^19^F-NMR spectra were measured for each compound separately in DMSO-*d*_6_. Then, equimolar mixtures of 1c with Arg-OMe and 9a with Arg-OMe were prepared, and the ^19^F- and ^11^B-NMR spectra were measured again in DMSO-*d*_6_. Based on the results (Fig. 10), no significant changes in chemical shifts were observed compared to the observations of other researchers.^35,36^ It can be concluded that the coordinate covalent bond to Arg-OMe was not created, and therefore, only non-covalent interactions of the prepared compounds with the WT AR LBD should be considered. This finding might be in accordance with the fact, that arginine residue as a poor nucleophile can form a covalent bond with highly reactive 1,2-dicarbonyl derivatives only.^37^

### *In vitro* screening of newly designed analogs of flutamide

The boronic acids that were prepared were tested for their efficacy against various cancer cell lines such as human prostate cancer cell line PC-3 and human liver cancer cell line HepG2. The use of the HepG2 cancer cell line was rationalized due to the fact, that some of the discussed derivatives bear pyrazinamide moiety within their structure. Most of the anti-tuberculosis drugs have issues with severe hepatotoxicity. To cover this issue, we decided to include this cell line in screening. The test was conducted using a commercial CellTiter 96 assay with an incubation time of 24 hours. Furthermore, the anti-proliferative activity of the series was also investigated against the androgen-dependent LAPC-4 human prostate cancer cell line with a prolonged incubation time of 72 hours.^38–40^ Selected compounds from the series were then evaluated for their anti-proliferative activity against the PC-3 prostate cancer cell line with a prolonged incubation time of 72 hours.^39^ The compounds that showed promising results were then tested against non-cancerous immortalized human kidney cell line HK-2 with 72 hours incubation time to determine their selectivity. In the assay, clinically used NSAAs such as flutamide, HF as its active metabolite, and bicalutamide were used as a reference for comparison. For detailed results, refer to the ESI† (Tables S1–S4).

In order to investigate the effect of prepared boronic acids we focused on their antiproliferative activity (selected compounds in Table 1) against androgen-dependent prostate cancer cell line LAPC-4, that expresses the WT AR.^38–41^ Surprisingly, we found that carboxamides substituted with 5-chloropyrazine (5a, 9a, and 10a) were the most active compounds. The position of chlorine in the heterocycle played a crucial role, as the shift of chlorine to position 6 in the pyrazine ring (4a) led to the diminishment of the activity, and the compounds without chlorine substitution did not show any activity. Moreover, the replacement of the 5-chloro substitution for the 5-hydroxy group (11a) caused a complete loss of activity against the LAPC-4 cell line. The addition of fluorine in the *ortho* position in the benzene core next to the free boronic acid resulted in a significant increase in the antiproliferative activity. This increase could be explained by the closer similarity to NSAAs containing a trifluoromethyl group or chlorine substitution in this position.

**Table 1:** Comparison of antiproliferative activity of the selected boronic acids with standards and the calculated selectivity index (SI); comparing activity against HK-2/LAPC-4. The complete results of antiproliferative activity of all synthesized compounds are in the Tables S1–S3 in the ESI†

Structure	Code	IC_50_ (μM)					
		HepG2 (24 h)	PC-3 (24 h)	PC-3 (72 h)	HK-2 (72 h)	LAPC-4 (72 h)	SI
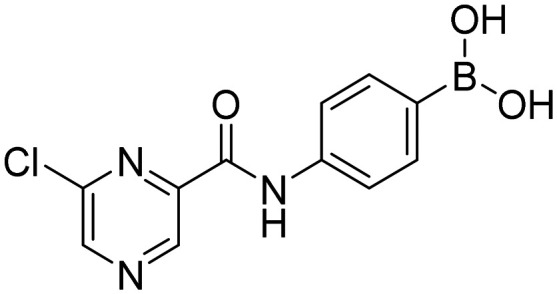	4a	>500	>250	na	na	>100	na
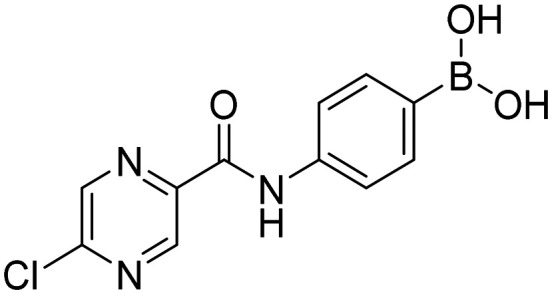	5a	139.3	133.4	121.6	194.5	27.8	7.0
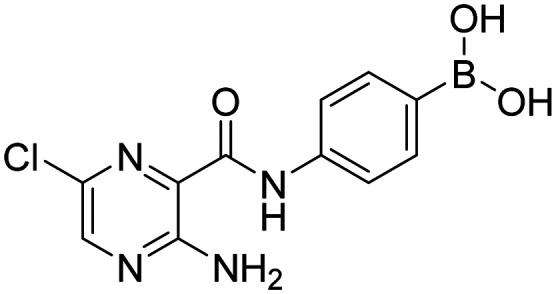	8a	126.4	193.3	375.2	422.4	78.3	5.4
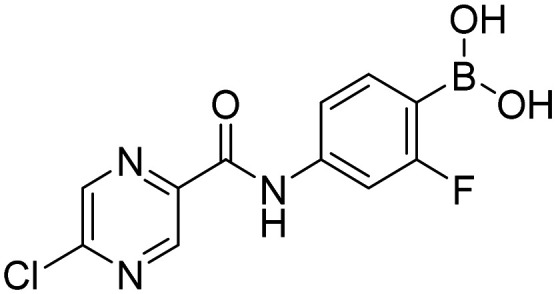	9a	71.3	118.4	78.1	137.7	19.2	7.2
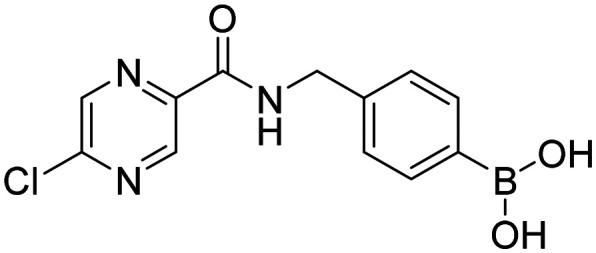	10a	187	>1000	227.9	183.7	48.0	3.8
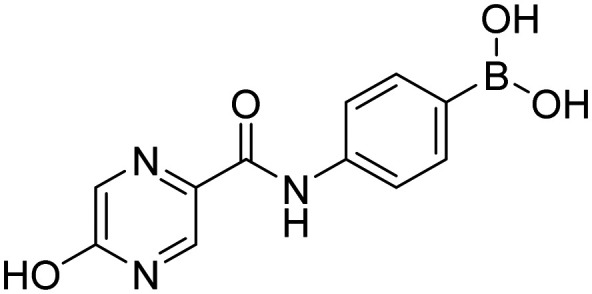	11a	>1000	296	779.6	>1000	>100	na
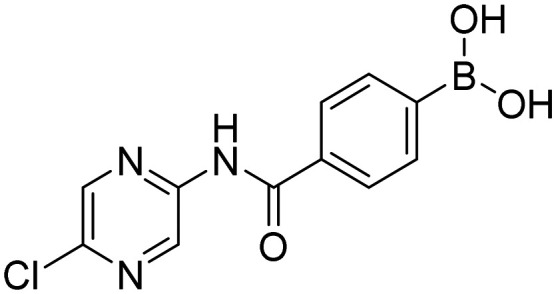	12a	>1000	na	na	na	>100	na
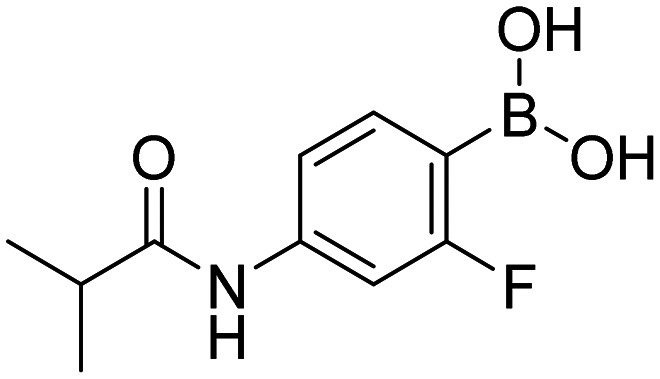	1b	>1000	>1000	na	na	>100	na
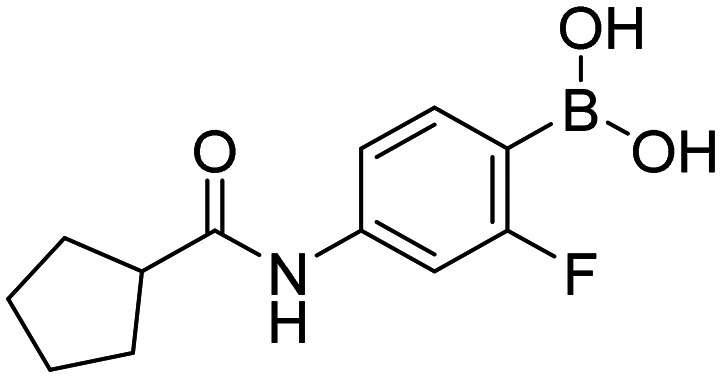	7b	>500	>1000	na	na	>100	na
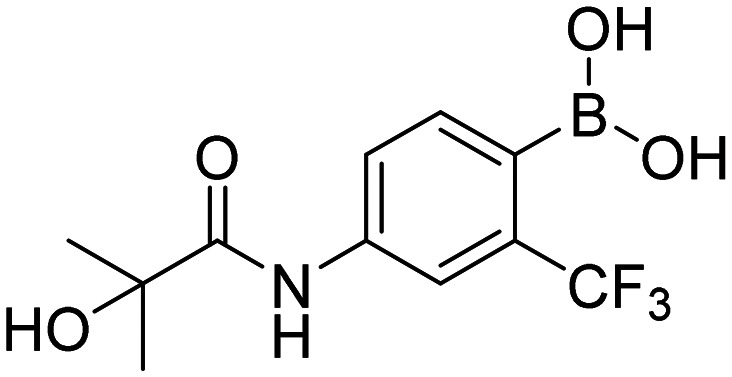	HFB	>1000	>1000	>1000	>1000	403.8	na
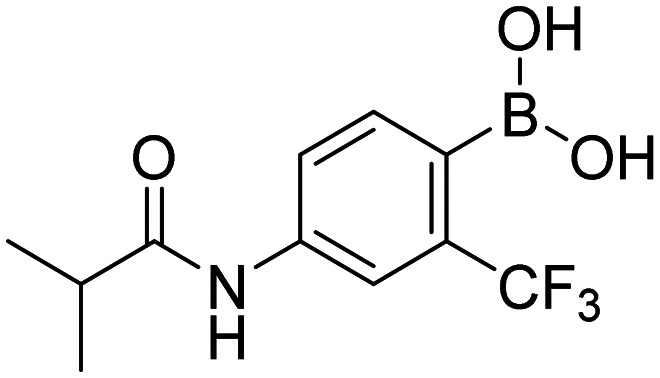	1c	>1000	>1000	697.1	>1000	>100	na
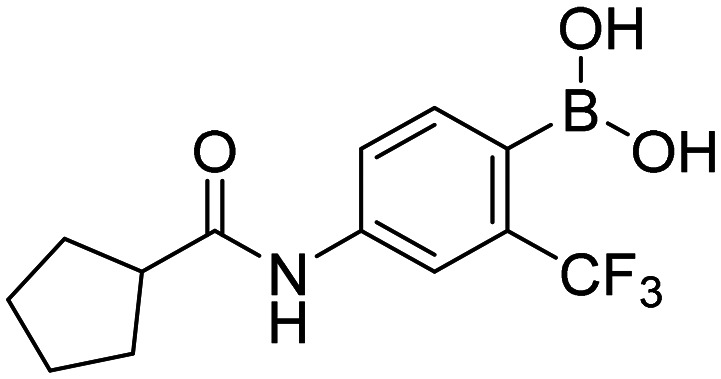	5c	>1000	>1000	185.6	>1000	>100	na
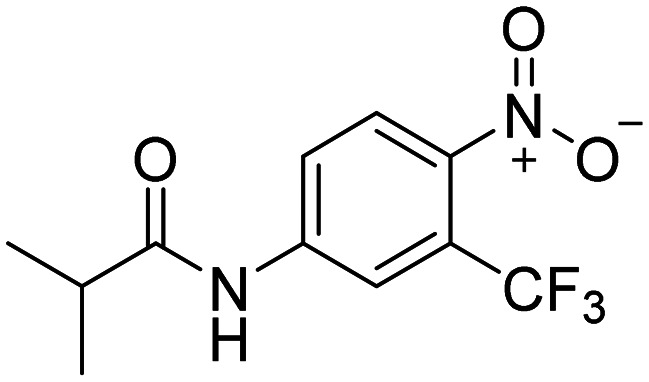	**Flutamide**	137.2	34.7	50.8	76.2	60.5	1.3
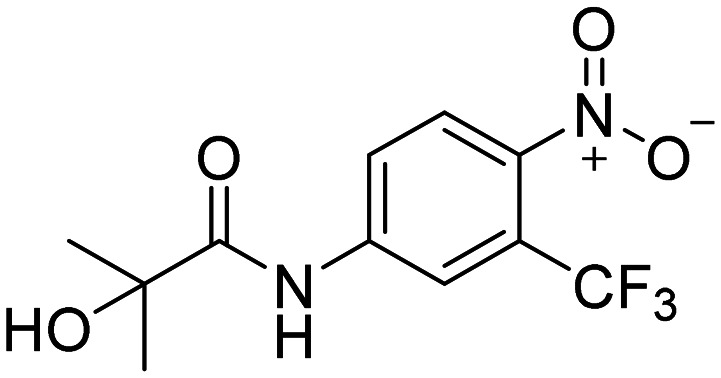	HF	103.2	229.2	41.8	231.0	136.1	1.7
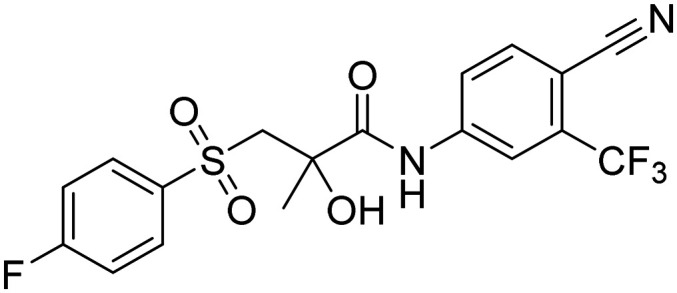	**Bicalutamide**	na	na	na	na	37.2	na

Interestingly, pyrazine contributed more to the antiproliferative activity than the fluorine substitution in the benzene ring. This was evident from the loss of activity of the amides derived from aliphatic or alicyclic acids with the preserved fluoro substitution in the *ortho* position of the benzene ring. In addition, the amide linker and its arrangement played an important role in retaining activity. The extension of the amide linker between the two aromatic cores by a methylene group led to a decrease in the antiproliferative activity (10a), and the employment of the *retro* amide led to the complete loss of activity (12a). This follows from the conclusions of our *in silico* study (Design of compounds and *in silico* experiment) that the position of the amide group, especially its nitrogen, is important for the H-bond interaction with Leu704 within the AR LBD. These structure–activity (against LAPC-4 cell line) relationships can potentially guide us to the thesis that boronic acid acts as a sufficient bioisostere for the nitro or nitrile function in the structure of NSAAs. On the contrary, the logical replacement of fluorine for the trifluoromethyl group in the *ortho* position neighbouring to the free boronic acid did not bring the intended increase in antiproliferative activity. These *ortho* trifluoromethyl substituted derivatives did not exert any activity against the discussed cancer cell line. Notably compounds 1c and HFB, the complete boronic bioisosteres of flutamide and HF, resp., could be taken as disproof of the above-mentioned statement because it did not exert any activity at the tested concentrations against any of the tested cancer cell lines besides mild activity of 1c against PC-3 and HFB against LAPC-4 with 72 hours of incubation. These findings highlight the importance of 5-chloro substitution of pyrazinamide derivatives in the specificity of its effect.

The results of screening against PC-3 and HepG2 cancer cell lines, with 24 hours of incubation time, mostly correlated with the results of LAPC-4 screening. It is noteworthy that the achieved IC_50_ values were higher against PC-3 and HepG2 than those against LAPC-4. However, this still supports the idea that the discussed compounds are likely to have an antiandrogen effect. When a longer incubation time was used, the activity of selected compounds against the PC-3 cancer cell line increased. Considering that the PC-3 cancer cell line is androgen-independent, it is important to take into account the possibility of multi-target activity of the presented series.^40^ Such activity could prove to be beneficial in cases of castration-resistant tumors.^42^

Out of the compounds studied, three (5a, 9a, and 10a) showed similar or better antiproliferative activity against the LAPC-4 cancer cell line compared to flutamide, HF, and bicalutamide. These compounds also exhibited mild activity against other tested cancer cell lines, similar to that of flutamide, but with better selectivity. To test the toxicity of these compounds, their antiproliferative activity against the non-cancerous HK-2 cell line was determined. The ratio of IC_50_ values against the androgen-dependent cancer cell line LAPC-4 and the non-cancerous cell line HK-2 was then calculated as the selectivity index (SI). All of the tested compounds showed lower toxicity against the HK-2 cell line in comparison to flutamide, and achieved better selectivity than flutamide, as well as HF. This confirms the toxicity and lack of selectivity of flutamide. Although HFB and 1c share structural similarities with HF and flutamide, they did not show any significant activity. We conclude that flutamide is a small molecule that lacks the necessary selectivity towards the AR and achieves its activity mainly due to the toxicity of its nitro group.^7^ The compound 9a from the series exhibited the highest activity with an IC_50_ of 19.2 μM against LAPC-4 and a selectivity index (SI) of 7.2 compared to flutamide (IC_50_ = 60.5 μM and SI = 1.3). An experimental proof of the real target for the most promising compounds is needed for validation in the future.

## Conclusion

This work proposes the replacement of the nitro/nitrile group in the structure of NSAAs with free boronic acid. A total of thirty-three new compounds containing boronic acid were prepared and their structures and biological activity against several cancer cell lines were fully characterized. *In silico* studies confirmed the ability of the presented series to bind to the WT AR LBD. However, the possibility of the N–B covalent binding of the presented compounds to the Arg752 residue was disproved based on the results of ^11^B- and ^19^F-NMR experiments.

From *in vitro* studies, it was concluded that boronic acid cannot function as a sufficient bioisostere of the nitro group in the structure of NSAAs. This is because the complete flutamide bioisostere 4-isobutyramido-2-(trifluoromethyl)phenylboronic acid did not exert any activity against the androgen-dependent prostatic cancer cell line LAPC-4.

However, several compounds from the presented series showed significant antiproliferative activity against both the androgen-dependent LAPC-4 and the androgen-independent PC-3 prostate cancer cell lines. The significant activity against the LAPC-4 cancer cell line was in accordance with the potential binding to the androgen receptor. Certain compounds (5a, 9a) have demonstrated anti-LAPC-4 activity that surpasses the effectiveness of flutamide, hydroxyflutamide, and bicalutamide, while exhibiting lower toxicity against non-cancerous HK-2 cells. These findings highlight the potential of boronic acids in the field of prostate cancer treatment and suggest that further research should be undertaken to verify their mechanisms of action. With additional structural modifications, this series of compounds may lead to the development of more effective and selective treatments in the future.

## Experimental section

### *In silico* study

**Fig. 11:**
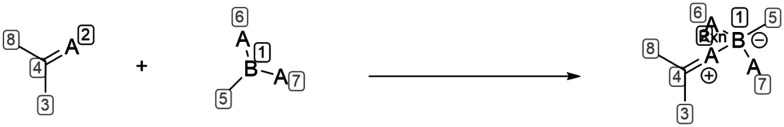
Reaction scheme used for definition of covalent bond in MOE.

Docking studies were conducted using the Dock utility in MOE 2022.02 (Chemical Computing Group, Montreal, QC, Canada), employing the AMBER10: EHT force field. The ligand binding domain of the WT AR, co-crystallized with (*R*)-3-bromo-2-hydroxy-2-methyl-*N*-[4-nitro-3-(trifluoromethyl)phenyl]propanamide (PDB ID: 2AX9), was downloaded from the RCSB PDB. Firstly, 2AX9 was adjusted by removing the solvent molecules, except for the water molecule that formed H-bond interactions between the ligand and two amino acids Arg752 and Gln711. Then, the WT AR LBD was prepared for subsequent docking using the QuickPrep function (with default settings). The structures of compounds 9a and 1c were designed in ChemDraw 20.0 (PerkinElmer Informatics, Waltham, MA, USA). After importing into MOE, the structures were prepared by selecting the protonation state at pH = 7, calculating partial charges, and minimizing energy until RMS = 0.00001 kcal mol^−1^ Å^−1^. The compounds were docked in 2AX9 using the general docking utility with a defined pharmacophore model. The pharmacophore model was defined based on the previous ligand of 2AX9, and the position of the oxygen atom from the amide bond was set as an acceptor, the position of the nitrogen atom from the amide bond was set as a donor, and the *R*-value was set as 1 Å. The thirty best poses from placement were refined to the final five poses for each entry (minimized inside the rigid receptor), and scored using the GBVI/WSA dG scoring function. The compounds were also docked in 2AX9 using the covalent docking utility. The reactive site was defined as the nitrogen atom of guanidine function from Arg752 residue. The scheme describing the covalent bond formation between the boronic acid function and guanidine function was drawn in ChemDraw 20.0 and was saved as an .rdf file and then used to define the reaction (Fig. 11). The thirty best poses from placement were refined to the final five poses for every entry (minimized inside the rigid receptor) and scored using the GBVI/WSA dG scoring function. Finally, the resulting poses were analyzed visually.

### Chemistry

#### General information

All reagents and solvents were purchased from Sigma-Aldrich (Schnelldorf, Germany) or Fluorochem (Hadfield, Derbyshire, UK). They were used without purification with the exception of starting 4-bromo-3-(trifluoromethyl)aniline purchased from Fluorochem (Hadfield, Derbyshire, UK), that required flash purification before being used in borylation. Thin-layer chromatography (TLC) using silica gel 60 F_254_ sheets (Merck, Darmstadt, Germany) was used to check the reaction progress and purity of products. Flash chromatography was performed with PuriFlash XS 420+ (Interchim, Montluçon, France) using original columns (PF-30-SIHP-JP-F0080, spherical silica, particle size 30 μm). UV-vis detection at 254 and 280 nm was used for detection. Yields are given in percentage and refer to the amount of pure product after all purification steps. Melting points were determined in an open capillary with a Stuart SMP30 melting point apparatus (Bibby Scientific Limited, Staffordshire, UK) and were uncorrected. The NMR spectra were recorded with a Varian VNMR (Varian, Palo Alto, CA, USA) at 500 MHz for ^1^H-NMR spectra, 126 MHz for ^13^C-NMR spectra, and 160 MHz for ^11^B-NMR spectra. They were also recorded with Jeol JNM-ECZ600R (Jeol Ltd., Tokyo, Japan) at 600 MHz for ^1^H-NMR spectra, 151 MHz for ^13^C-NMR spectra, 193 MHz for ^11^B-NMR spectra, and 565 MHz for ^19^F-NMR spectra. The chemical shifts reported as *δ* values in ppm were indirectly referred to tetramethylsilane (TMS) *via* the solvent signal (2.49 for ^1^H and 39.7 for ^13^C in DMSO-*d*_6_), (7.24 for ^1^H and 77.0 for ^13^C in CDCl_3_). IR spectra were recorded with a NICOLET 6700 FT-IR spectrophotometer (Thermo Scientific, Waltham, MA, USA) using the ATR-Ge method. The following instruments were used to perform elemental analyses and determine the purity of compounds in the study. Elemental analyses for non-fluorinated compounds were conducted using a Vario Micro Cube elemental analyzer (Elementar Analysensysteme, Hanau, Germany). Results were reported as percentages. The purity of the fluorinated compounds was determined using an Agilent Technologies 1200 SL liquid chromatograph (Agilent Technologies, Santa Clara, CA, USA), which included a vacuum microdegasser, 1200 SL binary pump, 1200 SL plus autosampler, TCC Infinity 1290 column thermostat and 1200 SL diode-array detector. The column used was a ZORBAX XDB C-18 250 × 4.6 mm, 5 μm (Agilent Technologies, Santa Clara, CA, USA). The mobile phase was water/acetonitrile in a ratio of 7 : 3 (v/v), with a flow rate of 0.6 mL min^−1^ and the temperature of the column set to 25 °C. The chromatographic system was controlled by Agilent ChemStation (Agilent Technologies, Santa Clara, CA, USA), version B.04.02 extended by a spectral module. The area percentage method was used at a wavelength of 250 nm to determine purity where it was proven to have >95% purity. Mass spectra in both positive and negative modes (APCI-MS) were measured using the Expression® compact mass spectrometer (Advion, Ithaca, NY, USA) with a single-quad detector. Samples were applied as solids by the ASAP probe method (Advion, Ithaca, NY, USA). Fluorinated compounds were determined as >95% pure by HPLC analysis, and for non-fluorinated compounds, the difference between the experimental and the calculated percentage for individual elements was determined as less than 0.4% by elemental analysis.

#### General synthetic procedures

##### Synthesis of l-arginine methyl ester dihydrochloride

l-Arginine (1.0 g) was suspended in 15 mL of methanol. Hydrogen chloride was then passed through this suspension for 10 minutes. The reaction was refluxed for 15 minutes and after the indicated time, the solvent was evaporated. The remaining solid was redissolved in 15 mL of methanol and the procedure was repeated two more times. Once the procedure was complete, the solvent was evaporated and the resulting foam was redissolved in 4 mL of methanol. A small amount of diethyl ether was added to this solution until it became slightly opalescent. The opalescent solution was left in the refrigerator overnight to complete the crystallization process. Finally, the solvent was removed to produce the final product, Arg-OMe.^43^

##### Synthesis of 4-(4,4,5,5-tetramethyl-1,3,2-dioxaborolan-2-yl)-3-(trifluoromethyl)aniline

The starting 4-bromo-3-(trifluoromethyl)aniline (16.7 mmol) was first purified by flash chromatography using gradient elution from 0 to 100% ethyl acetate in hexane. Clean aniline isolated and dried over anhydrous phosphorus pentoxide under reduced pressure 1.33 kPa at room temperature overnight. The round-bottom flask was then charged with the purified 4-bromo-3-(trifluoromethyl)aniline (1 eq., 15.2 mmol), KOAc (2 eq., 30.4 mmol), and bis(pinacolato)diboron (1 eq., 15.2 mmol) in anhydrous DMF (37 mL), last bis[(diphenylphosphino)ferrocene]dichloropalladium(ii) (0.05 eq., 0.76 mmol) was added to the reaction. The reaction proceeded under anhydrous conditions with argon atmosphere and was refluxed at 85 °C for 16 hours. After the indicated time, the solvent was evaporated and water (45 mL) was added to the reaction residue, then the water mixture was extracted three times with EtOAc (45 mL). The organic layers were collected and dried with anhydrous Na_2_SO_4_. The mixture was purified with silica gel column chromatography with DCM (as a mobile phase).

##### Acylation procedures

Compounds 1aP–20aP, except for compounds 12aP and 17aP were prepared by following procedure. The corresponding (hetero)arylcarboxylic acid (4 mmol) was activated with 1,1′-carbonyldiimidazole (CDI; 4.4 mmol) in DMSO (12 mL) for 1 hour. After a short initial heating, it proceeded at room temperature. Then 4-(4,4,5,5-tetramethyl-1,3,2-dioxaborolan-2-yl)aniline (4 mmol) dissolved in DMSO (10 mL) was added and the reaction mixture was stirred at room temperature overnight. The work-up was accomplished by the addition of deionized water (20 mL). Within one hour of stirring, precipitation appeared. The precipitate was filtrated, properly washed with water, and purified using flash chromatography with the mobile phase consisting of hexane/ethyl acetate using gradient elution.

Compound 12aP was prepared by following procedure. In a round-bottom flask 4-(4,4,5,5-tetramethyl-1,3,2-dioxaborolan-2-yl)benzoic acid (6 mmol) was dissolved in toluene (25 mL) and thionyl chloride was added (6.6 mmol). The reaction was refluxed for 24 hours. Then, the mixture was evaporated to dryness and dissolved in DCM (8 mL). Another round-bottom flask was charged with 5-chloropyrazin-2-amine (4 mmol), pyridine (10 mmol), and DCM (9 mL). The acyl chloride was added dropwise to the amine while cooling in an ice bath. After mixing the two components, the mixture was stirred at room temperature overnight. After completion of the reaction, the mixture was evaporated to dryness. The crude residue was dissolved in water (20 mL) and extracted three times with EtOAc (30 mL). The collected organic layers were dried with anhydrous Na_2_SO_4_. The crude product was purified with flash chromatography using gradient elution hexane/EtOAc.

Compound 17aP was prepared by following procedure. The round-bottom flask was charged with 4-chlorobenzoic acid (4 mmol), oxalyl chloride (4.8 mmol), and DCM (10 mL). A few drops of DMF were added to accelerate the activation process and the mixture was stirred for 15 minutes at room temperature. Another flask was charged with 4-(4,4,5,5-tetramethyl-1,3,2-dioxaborolan-2-yl)aniline (4 mmol), pyridine (7.2 mmol), and DCM (10 mL). Acyl chloride was added dropwise to amine while cooling in an ice bath. The mixture was stirred at room temperature overnight. After completion of the reaction, the mixture was evaporated to dryness. The crude residue was dissolved in water (20 mL) and extracted three times with EtOAc (30 mL). The collected organic layers were dried with anhydrous Na_2_SO_4_. The crude product was purified with flash chromatography using gradient elution hexane/EtOAc.

Compounds HFB-AP, 1bP–7bP and 1cP–5cP were prepared by following procedure. The round-bottom flask was charged with the appropriate fluorine-substituted aniline (3 mmol), pyridine (4.5 mmol), and DCM (8 mL). The mixture was placed in an ice bath. The solution of corresponding acyl-chloride (3 mmol) dissolved in DCM (8 mL) was dropwise added to the mixture. The mixture was stirred at room temperature overnight. After completion of the reaction, the mixture was evaporated to dryness. The crude residue was dissolved in water (20 mL) and extracted three times with EtOAc (30 mL). The collected organic layers were dried with anhydrous Na_2_SO_4_. The crude product was purified with flash chromatography using isocratic elution hexane/EtOAc (7 : 3 ratio).

##### Basic hydrolysis of acetoxy group

Compound HFB-P was prepared by following procedure. The round bottom flask was charged with 2-methyl-1-oxo-1-{[4-(4,4,5,5-tetramethyl-1,3,2-dioxaborolan-2-yl)-3-(trifluoromethyl)phenyl]amino}propan-2-yl acetate (2 mmol), potassium carbonate (3 mmol) and ethanol (50 ml). The mixture was placed on the heating block with reflux condenser. The solution was stirred at 79 °C for 30 minutes. After indicated time the reaction was filtered to get rid of potassium carbonate. The crude product was purified with flash chromatography using gradient elution hexane/EtOAc.

##### Deprotection of pinacol esters

After purification, the intermediates underwent subsequent deprotection *via* the transesterification approach. The corresponding pinacol ester (1 eq.) and methyl boronic acid (10 eq.) were mixed and dissolved in the mixture of acetone and 0.2 N HCl (1 : 1 v/v), amount as required. Upon completion of the transesterification, the reaction mixture was concentrated to dryness, then redissolved in deionized water and evaporated to dryness one more time to avoid boron anhydride formation to obtain free boronic acid.^30^ The free boronic acid was then suspended in deionized water, filtrated, and washed properly with water to get rid of residual impurities.

All final compounds were dried over anhydrous phosphorus pentoxide under reduced pressure 1.33 kPa at room temperature overnight before characterization and purity check.

#### Characterization and purity of the prepared compounds

For methyl ester of l-arginine and synthetic intermediates (compounds BAF, HFB-AP, HFB-P, 1aP–20aP, 1bP–7bP, and 1cP–5cP), the appearance, reaction yield, and interpretation of ^1^H-NMR and ^13^C-NMR (using below mentioned abbreviations) are reported. For each compound presented in the main text (compounds HFB, 1a–20a, 1b–7b and 1c–5c), we report on the appearance, reaction yield, melting point, elemental analysis or HPLC analysis, and interpretation of ^1^H-NMR (used abbreviations: ArH – benzene hydrogen, PzH – pyrazine hydrogen, PyH – pyridine hydrogen, QxH – quinoxaline hydrogen, Tf – thiophene hydrogen, LmH – hydrogen involved in lactam–lactim tautomerism), ^13^C-NMR (used abbreviations: PyC – pyridine carbon), ^11^B-NMR, IR, and MS spectra.

The ^13^C NMR spectra of intermediate 7aP and compound 7a were not possible to measure due to the poor solubility, therefore they are not included in the interpretations.

##### l-Arginine methyl ester dihydrochloride (Arg-OMe)

White solid. Yield: 80%. ^1^H NMR (500 MHz, DMSO-*d*_6_) *δ* 8.72 (bs, 3H, NH_3_^+^), 7.99 (t, *J* = 5.9 Hz, 1H, NH), 7.54 (bs, 2H, NH_2_), 7.16 (bs, 2H, NH_2_), 4.02 (t, *J* = 6.4 Hz, 1H, CH), 3.73 (s, 3H, CH_3_), 3.19–3.06 (m, 2H, CH_2_), 1.90–1.76 (m, 2H, CH_2_), 1.66–1.42 (m, 2H, CH_2_). ^13^C NMR (126 MHz, DMSO-*d*_6_) *δ* 169.98, 157.36, 53.01, 51.67, 40.16, 27.34, 24.45.

##### 4-(4,4,5,5-Tetramethyl-1,3,2-dioxaborolan-2-yl)-3-(trifluoromethyl)aniline (BAF)

Beige solid. Yield: 24%. ^1^H NMR (600 MHz, DMSO-*d*_6_) *δ* 7.44 (d, *J* = 8.2 Hz, 1H, ArH), 6.89 (d, *J* = 2.2 Hz, 1H, ArH), 6.70 (dd, *J* = 8.2, 2.3 Hz, 1H, ArH), 5.85 (s, 2H, NH_2_), 1.23 (s, 12H, CH_3_). ^13^C NMR (151 MHz, DMSO-*d*_6_) *δ* 151.63, 138.04, 134.74 (q, *J* = 30.3 Hz), 124.97 (q, *J* = 273.8 Hz, CF_3_), 115.59, 110.87 (q, *J* = 5.3 Hz), 83.69, 24.91.

##### 2-Methyl-1-oxo-1-{[4-(4,4,5,5-tetramethyl-1,3,2-dioxaborolan-2-yl)-3-(trifluoromethyl)phenyl]amino}propan-2-yl acetate (HFB-AP)

White solid. Yield: 71%. ^1^H NMR (600 MHz, DMSO-*d*_6_) *δ* 9.91 (s, 1H, CONH), 8.08 (d, *J* = 2.0 Hz, 1H, ArH), 7.92 (dd, *J* = 8.2, 2.0 Hz, 1H, ArH), 7.68 (d, *J* = 8.2 Hz, 1H, ArH), 2.06 (s, 3H, CH_3_), 1.55 (s, 6H, CH_3_), 1.28 (s, 12H, CH_3_). ^13^C NMR (151 MHz, DMSO-*d*_6_) *δ* 171.95, 170.13, 141.55, 136.56, 133.57 (q, *J* = 31.1 Hz), 125.56 (q, *J* = 273.5 Hz, CF_3_), 122.53, 117.10, 84.55, 80.16, 24.88, 24.82, 21.94.

##### 2-Hydroxy-2-methyl-*N*-[4-(4,4,5,5-tetramethyl-1,3,2-dioxaborolan-2-yl)-3-(trifluoromethyl)phenyl]propanamide (HFB-P)

White solid. Yield: 54%. ^1^H NMR (600 MHz, DMSO-*d*_6_) *δ* 10.01 (s, 1H, CONH), 8.27 (d, *J* = 2.0 Hz, 1H, ArH), 8.01 (dd, *J* = 8.2, 2.0 Hz, 1H, ArH), 7.66 (d, *J* = 8.2 Hz, 1H, ArH), 5.75 (s, 1H, OH), 1.35 (s, 6H, CH_3_), 1.28 (s, 12H, CH_3_). ^13^C NMR (151 MHz, DMSO-*d*_6_) *δ* 176.85, 141.46, 136.47, 133.62 (q, *J* = 31.0 Hz), 124.68 (q, *J* = 273.6 Hz, CF_3_), 122.15, 116.76 (q, *J* = 5.3 Hz), 84.52, 73.04, 28.08, 24.88.

##### 4-(2-Hydroxy-2-methylpropanamido)-2-(trifluoromethyl)phenylboronic acid (HFB)

White solid. Yield: 98%. mp 134.2–136.7 °C. ^1^H NMR (500 MHz, DMSO-*d*_6_) *δ* 9.86 (s, 1H, CONH), 8.21 (bs, 2H, BO_2_H_2_), 8.17 (d, *J* = 2.0 Hz, 1H, ArH), 7.88 (dd, *J* = 8.1, 2.0 Hz, 1H, ArH), 7.43 (d, *J* = 8.1 Hz, 1H, ArH), 5.75 (bs, 1H, OH), 1.34 (s, 6H, CH_3_). ^13^C NMR (151 MHz, DMSO-*d*_6_) *δ* 176.58, 139.42, 133.53, 131.60 (q, *J* = 30.4 Hz), 126.15 (q, *J* = 273.9 Hz, CF_3_), 122.48, 116.37, 73.00, 28.14. ^11^B NMR (193 MHz, DMSO-*d*_6_) *δ* 28.42. IR (ATR-Ge, cm^−1^): IR (ATR-Ge, cm^−1^): 3230 (*ν* O–H), 1673 (*δ* C=O), 1521 (*δ* CONH), 1368 (*ν* B–O), 632 (*δ* BO_2_). HPLC purity 97.7%. MS: [M + H]^+^ = 291.9 (exact mass 291.1).

##### *N*-[4-(4,4,5,5-Tetramethyl-1,3,2-dioxaborolan-2-yl)phenyl]pyrazine-2-carboxamide (1aP)

White solid. Yield: 62%. ^1^H NMR (500 MHz, DMSO-*d*_6_) *δ* 10.78 (s, 1H, CONH), 9.28 (d, *J* = 1.5 Hz, 1H, PzH), 8.92 (d, *J* = 2.5 Hz, 1H, PzH), 8.80 (dd, *J* = 2.5, 1.5 Hz, 1H, PzH), 7.92 (d, *J* = 8.3 Hz, 2H, ArH), 7.67 (d, *J* = 8.3 Hz, 2H, ArH), 1.28 (s, 12H, CH_3_). ^13^C NMR (126 MHz, DMSO-*d*_6_) *δ* 162.06, 147.92, 145.16, 144.26, 143.39, 141.12, 135.30, 119.71, 83.70, 24.84.

##### 5-Methyl-*N*-[4-(4,4,5,5-tetramethyl-1,3,2-dioxaborolan-2-yl)phenyl]pyrazine-2-carboxamide (2aP)

White solid. Yield: 36%. ^1^H NMR (600 MHz, DMSO-*d*_6_) *δ* 10.70 (s, 1H, CONH), 9.14 (d, *J* = 1.4 Hz, 1H, PzH), 8.70–8.66 (m, 1H, PzH), 7.96–7.87 (m, 2H, ArH), 7.70–7.61 (m, 2H, ArH), 2.62 (s, 3H, CH_3_), 1.28 (s, 12H, CH_3_). ^13^C NMR (151 MHz, DMSO-*d*_6_) *δ* 162.57, 157.84, 143.62, 143.28, 142.77, 141.59, 135.66, 120.04, 84.06, 25.22, 21.97.

##### 3-Methyl-*N*-[4-(4,4,5,5-tetramethyl-1,3,2-dioxaborolan-2-yl)phenyl]pyrazine-2-carboxamide (3aP)

Beige solid. Yield: 60%. ^1^H NMR (600 MHz, DMSO-*d*_6_) *δ* 10.72 (s, 1H, CONH), 8.72 (d, *J* = 2.4 Hz, 1H, PzH), 8.60 (d, *J* = 2.4 Hz, 1H, PzH), 7.83 (d, *J* = 8.0 Hz, 2H, ArH), 7.66 (d, *J* = 8.0 Hz, 2H, ArH), 2.75 (s, 3H, CH_3_), 1.28 (s, 12H, CH_3_). ^13^C NMR (151 MHz, DMSO-*d*_6_) *δ* 164.56, 153.62, 146.33, 145.83, 141.87, 141.28, 135.78, 119.60, 84.08, 25.25, 22.98.

##### 6-Chloro-*N*-[4-(4,4,5,5-tetramethyl-1,3,2-dioxaborolan-2-yl)phenyl]pyrazine-2-carboxamide (4aP)

Light yellow solid. Yield: 35%. ^1^H NMR (600 MHz, DMSO-*d*_6_) *δ* 10.73 (s, 1H, CONH), 9.25–9.19 (m, 1H, PzH), 9.08–9.04 (m, 1H, PzH), 7.91–7.88 (m, 2H, ArH), 7.71–7.65 (m, 2H, ArH), 1.29 (s, 12H, CH_3_). ^13^C NMR (151 MHz, DMSO-*d*_6_) *δ* 161.40, 148.04, 147.42, 145.62, 142.93, 141.33, 135.67, 120.32, 84.10, 25.23.

##### 5-Chloro-*N*-(4-(4,4,5,5-tetramethyl-1,3,2-dioxaborolan-2-yl)phenyl)pyrazine-2-carboxamide (5aP)

White solid. Yield: 36%. ^1^H NMR (600 MHz, DMSO-*d*_6_) *δ* 10.80 (s, 1H, CONH), 9.10 (d, *J* = 1.4 Hz, 1H, PzH), 8.92 (d, *J* = 1.4 Hz, 1H, PzH), 7.95–7.87 (m, 2H, ArH), 7.68–7.65 (m, 2H, ArH), 1.28 (s, 12H, CH_3_). ^13^C NMR (151 MHz, DMSO-*d*_6_) *δ* 161.65, 151.52, 144.63, 144.32, 143.47, 141.41, 135.67, 120.20, 84.09, 25.25.

##### 3-Amino-*N*-[4-(4,4,5,5-tetramethyl-1,3,2-dioxaborolan-2-yl)phenyl]pyrazine-2-carboxamide (6aP)

Yellow solid. Yield: 16%. ^1^H NMR (500 MHz, DMSO-*d*_6_) *δ* 10.55 (s, 1H, CONH), 8.28 (d, *J* = 2.3 Hz, 1H, PzH), 7.91 (d, *J* = 2.3 Hz, 1H, PzH), 7.89–7.83 (m, 2H, ArH), 7.67–7.61 (m, 2H, ArH), 7.58 (bs, 2H, NH_2_), 1.28 (s, 12H, CH_3_). ^13^C NMR (126 MHz, DMSO-*d*_6_) *δ* 165.16, 155.94, 147.98, 141.45, 135.58, 131.45, 125.61, 119.79, 83.96, 25.15.

##### 3-Oxo-*N*-[4-(4,4,5,5-tetramethyl-1,3,2-dioxaborolan-2-yl)phenyl]-3,4-dihydropyrazine-2-carboxamide (7aP)

Yellow solid. Yield: 66%. ^1^H NMR (500 MHz, DMSO-*d*_6_) *δ* 13.21 (bs, 1H, LmH), 11.53 (bs, 1H, CONH), 7.81 (s, 1H, PzH), 7.75–7.70 (m, 2H, ArH), 7.69–7.64 (m, 3H, ArH/PzH), 1.28 (s, 12H, CH_3_).

##### 3-Amino-6-chloro-*N*-[4-(4,4,5,5-tetramethyl-1,3,2-dioxaborolan-2-yl)phenyl]pyrazine-2-carboxamide (8aP)

Yellow solid. Yield: 14%. ^1^H NMR (500 MHz, CDCl_3_) *δ* 9.55 (s, 1H, CONH), 8.21 (s, 1H, PzH), 7.86–7.80 (m, 2H, ArH), 7.74–7.69 (m, 2H, ArH), 1.35 (s, 12H, CH_3_). ^13^C NMR (126 MHz, CDCl_3_) *δ* 162.89, 153.89, 146.88, 139.73, 135.87, 133.62, 124.07, 118.73, 83.78, 24.86.

##### 5-Chloro-*N*-[3-fluoro-4-(4,4,5,5-tetramethyl-1,3,2-dioxaborolan-2-yl)phenyl]pyrazine-2-carboxamide (9aP)

White solid. Yield: 15%. ^1^H NMR (600 MHz, DMSO-*d*_6_) *δ* 11.02 (s, 1H, CONH), 9.10 (d, *J* = 1.4 Hz, 1H, PzH), 8.93 (d, *J* = 1.4 Hz, 1H, PzH), 7.80–7.73 (m, 2H, ArH), 7.62 (dd, *J* = 8.5, 7.0 Hz, 1H, ArH), 1.28 (s, 12H, CH_3_). ^13^C NMR (151 MHz, DMSO-*d*_6_) *δ* 167.11 (d, *J* = 247.9 Hz), 161.99, 151.70, 144.76, 144.02, 143.57, 143.50, 137.47 (d, *J* = 9.6 Hz), 116.27, 107.33 (d, *J* = 28.9 Hz), 84.11, 25.15.

##### 5-Chloro-*N*-[4-(4,4,5,5-tetramethyl-1,3,2-dioxaborolan-2-yl)benzyl]pyrazine-2-carboxamide (10aP)

White solid. Yield: 12%. ^1^H NMR (600 MHz, DMSO-*d*_6_) *δ* 9.53 (t, *J* = 6.4 Hz, 1H, CONH), 9.00 (s, 1H, PzH), 8.87 (s, 1H, PzH), 7.61 (d, *J* = 7.7 Hz, 2H, ArH), 7.32 (d, *J* = 7.7 Hz, 2H, ArH), 4.51 (d, *J* = 6.4 Hz, 2H, CH_2_), 1.26 (s, 12H, CH_3_). ^13^C NMR (151 MHz, DMSO-*d*_6_) *δ* 162.76, 151.35, 144.06, 144.04, 143.72, 143.10, 135.02, 127.33, 84.10, 43.05, 25.19.

##### 5-Hydroxy-*N*-[4-(4,4,5,5-tetramethyl-1,3,2-dioxaborolan-2-yl)phenyl]pyrazine-2-carboxamide (11aP)

Brown solid. Yield: 57%. ^1^H NMR (600 MHz, DMSO-*d*_6_) *δ* 12.89 (bs, 1H, OH), 10.22 (s, 1H, CONH), 8.11–8.08 (m, 1H, PzH), 8.02 (d, *J* = 1.3 Hz, 1H, PzH), 7.88–7.82 (m, 2H, ArH), 7.64–7.58 (m, 2H, ArH), 1.27 (s, 12H, CH_3_). ^13^C NMR (151 MHz, DMSO-*d*_6_) *δ* 161.88, 156.77, 146.86, 141.86, 135.62, 131.53, 123.88, 119.79, 84.01, 25.22.

##### *N*-(5-Chloropyrazin-2-yl)-4-(4,4,5,5-tetramethyl-1,3,2-dioxaborolan-2-yl)benzamide (12aP)

White solid. Yield: 33%. ^1^H NMR (600 MHz, DMSO-*d*_6_) *δ* 11.38 (s, 1H, CONH), 9.24 (d, *J* = 1.4 Hz, 1H, PzH), 8.62 (d, *J* = 1.4 Hz, 1H, PzH), 8.06–7.99 (m, 2H, ArH), 7.82–7.74 (m, 2H, ArH), 1.31 (s, 12H, CH_3_). ^13^C NMR (151 MHz, DMSO-*d*_6_) *δ* 166.63, 148.55, 142.68, 142.45, 136.88, 136.13, 135.00, 134.85, 128.11, 84.59, 25.22.

##### *N*-[4-(4,4,5,5-Tetramethyl-1,3,2-dioxaborolan-2-yl)phenyl]picolinamide (13aP)

White solid. Yield: 21%. ^1^H NMR (600 MHz, DMSO-*d*_6_) *δ* 10.69 (s, 1H, CONH), 8.75–8.71 (m, 1H, PyH), 8.18–8.13 (m, 1H, PyH), 8.09–8.03 (m, 1H, PyH), 7.96–7.90 (m, 2H, ArH), 7.70–7.67 (m, 1H, PyH), 7.67–7.63 (m, 2H, ArH), 1.28 (s, 12H, CH_3_). ^13^C NMR (151 MHz, DMSO-*d*_6_) *δ* 163.21, 150.29, 149.01, 141.67, 138.72, 135.71, 127.58, 122.99, 119.81, 84.05, 25.22.

##### *N*-[4-(4,4,5,5-Tetramethyl-1,3,2-dioxaborolan-2-yl)phenyl]-6-(trifluoromethyl)nicotinamide (14aP)

White solid. Yield: 23%. ^1^H NMR (500 MHz, DMSO-*d*_6_) *δ* 10.73 (s, 1H, CONH), 9.24 (d, *J* = 2.1 Hz, 1H, PyH), 8.59–8.53 (m, 1H, PyH), 8.09 (dd, *J* = 8.2, 0.8 Hz, 1H, PyH), 7.83–7.78 (m, 2H, ArH), 7.72–7.66 (m, 2H, ArH), 1.28 (s, 12H, CH_3_). ^13^C NMR (126 MHz, DMSO-*d*_6_) *δ* 163.51, 149.82, 148.66 (q, *J* = 34.0 Hz, PyC), 141.86, 138.40, 135.72, 134.14, 124.44, 121.83 (q, *J* = 274.3 Hz, CF_3_), 121.06, 119.81, 84.01, 25.15.

##### *N*-[4-(4,4,5,5-Tetramethyl-1,3,2-dioxaborolan-2-yl)phenyl]-5-(trifluoromethyl)picolinamide (15aP)

White solid. Yield: 50%. ^1^H NMR (600 MHz, DMSO-*d*_6_) *δ* 10.85 (s, 1H, CONH), 9.12–9.09 (m, 1H, PyH), 8.49 (dd, *J* = 8.2, 2.3 Hz, 1H, PyH), 8.33 (dt, *J* = 8.2, 0.8 Hz, 1H, PyH), 7.96–7.91 (m, 2H, ArH), 7.70–7.64 (m, 2H, ArH), 1.29 (s, 12H, CH_3_). ^13^C NMR (151 MHz, DMSO-*d*_6_) *δ* 162.21, 153.94, 145.86, 141.44, 136.40, 135.70, 128.15, 127.93, 123.42, 120.10, 84.10, 25.23.

##### 5-Chloro-*N*-[4-(4,4,5,5-tetramethyl-1,3,2-dioxaborolan-2-yl)phenyl]thiophene-2-carboxamide (16aP)

Beige solid. Yield: 28%. ^1^H NMR (600 MHz, DMSO-*d*_6_) *δ* 10.37 (s, 1H, CONH), 7.93 (d, *J* = 4.1 Hz, 1H, TfH), 7.76–7.70 (m, 2H, ArH), 7.68–7.63 (m, 2H, ArH), 7.26 (d, *J* = 4.1 Hz, 1H, TfH), 1.28 (s, 12H, CH_3_). ^13^C NMR (151 MHz, DMSO-*d*_6_) *δ* 159.46, 141.80, 139.53, 135.76, 134.74, 129.92, 128.87, 124.19, 119.85, 84.06, 25.23.

##### 4-Chloro-*N*-[4-(4,4,5,5-tetramethyl-1,3,2-dioxaborolan-2-yl)phenyl]benzamide (17aP)

Beige solid. Yield: 49%. ^1^H NMR (600 MHz, DMSO-*d*_6_) *δ* 10.40 (s, 1H, CONH), 8.00–7.96 (m, 2H, ArH), 7.82–7.78 (m, 2H, ArH), 7.68–7.64 (m, 2H, ArH), 7.61–7.58 (m, 2H, ArH), 1.28 (s, 12H, CH_3_). ^13^C NMR (151 MHz, DMSO-*d*_6_) *δ* 165.14, 142.40, 137.08, 135.69, 134.04, 130.24, 129.02, 119.88, 84.04, 25.24.

##### *N*-[4-(4,4,5,5-Tetramethyl-1,3,2-dioxaborolan-2-yl)phenyl]quinoxaline-2-carboxamide (18aP)

Brown solid. Yield: 69%. ^1^H NMR (600 MHz, DMSO-*d*_6_) *δ* 10.90 (s, 1H, CONH), 9.53 (s, 1H, QxH), 8.34–8.26 (m, 1H, QxH), 8.25–8.19 (m, 1H, QxH), 8.06–7.99 (m, 2H, QxH), 7.98 (d, *J* = 8.2 Hz, 2H, ArH), 7.71 (d, *J* = 8.2 Hz, 2H, ArH), 1.29 (s, 12H, CH_3_). ^13^C NMR (151 MHz, DMSO-*d*_6_) *δ* 162.74, 145.14, 144.48, 143.51, 141.53, 140.18, 135.79, 135.73, 132.71, 131.95, 130.12, 129.75, 120.06, 84.11, 25.24.

##### 3-Oxo-*N*-[4-(4,4,5,5-tetramethyl-1,3,2-dioxaborolan-2-yl)phenyl]-3,4-dihydroquinoxaline-2-carboxamide (19aP)

Yellow solid. Yield: 43%. ^1^H NMR (500 MHz, DMSO-*d*_6_) *δ* 12.96 (bs, 1H, LmH), 11.19 (bs, 1H, CONH), 7.91–7.86 (m, 1H, QxH), 7.75–7.67 (m, 4H, ArH), 7.65 (t, *J* = 7.8 Hz, 1H, QxH), 7.42–7.36 (m, 2H, QxH), 1.29 (s, 12H, CH_3_). ^13^C NMR (126 MHz, DMSO-*d*_6_) *δ* 161.93, 154.01, 151.72, 141.29, 135.73, 132.72, 132.27, 131.38, 129.53, 124.27, 118.79, 115.92, 83.76, 24.90.

##### *N*-[4-(4,4,5,5-Tetramethyl-1,3,2-dioxaborolan-2-yl)phenyl]isoquinoline-1-carboxamide (20aP)

Yellow solid. Yield: 16%. ^1^H NMR (600 MHz, DMSO-*d*_6_) *δ* 10.90 (s, 1H, CONH), 8.83–8.79 (m, 1H, ArH), 8.65–8.60 (m, 1H, ArH), 8.11–8.06 (m, 2H, ArH), 7.92 (d, *J* = 8.2 Hz, 2H, ArH), 7.88–7.82 (m, 1H, ArH), 7.79–7.73 (m, 1H, ArH), 7.70 (d, *J* = 8.2 Hz, 2H, ArH), 1.29 (s, 12H, CH_3_). ^13^C NMR (151 MHz, DMSO-*d*_6_) *δ* 165.44, 151.80, 142.10, 141.43, 137.17, 135.83, 131.43, 129.26, 127.84, 126.75, 125.98, 124.19, 119.61, 84.07, 25.25.

##### *N*-[3-Fluoro-4-(4,4,5,5-tetramethyl-1,3,2-dioxaborolan-2-yl)phenyl]isobutyramide (1bP)

White solid. Yield: 25%. ^1^H NMR (600 MHz, DMSO-*d*_6_) *δ* 10.12 (s, 1H, CONH), 7.58–7.50 (m, 2H, ArH), 7.30 (dd, *J* = 8.2, 1.9 Hz, 1H, ArH), 2.58 (m, 1H, CH), 1.27 (s, 12H, CH_3_), 1.09 (d, *J* = 6.8 Hz, 6H, CH_3_). ^13^C NMR (151 MHz, DMSO-*d*_6_) *δ* 176.39, 167.37 (d, *J* = 247.7 Hz, CF), 144.87 (d, *J* = 11.8 Hz), 137.48 (d, *J* = 9.6 Hz), 114.76, 105.78 (d, *J* = 29.0 Hz), 83.96, 35.61, 25.16, 19.87.

##### *N*-[3-Fluoro-4-(4,4,5,5-tetramethyl-1,3,2-dioxaborolan-2-yl)phenyl]butyramide (2bP)

White solid. Yield: 14%.^1^H NMR (600 MHz, DMSO-*d*_6_) *δ* 10.16 (s, 1H, CONH), 7.58–7.50 (m, 2H, ArH), 7.28 (dd, *J* = 8.2, 1.8 Hz, 1H, ArH), 2.29 (t, *J* = 7.4 Hz, 2H, CH_2_), 1.59 (m, 2H, CH_2_), 1.26 (s, 12H, CH_3_), 0.90 (t, *J* = 7.4 Hz, 3H, CH_3_). ^13^C NMR (151 MHz, DMSO-*d*_6_) *δ* 172.34, 167.39 (d, *J* = 247.8 Hz, CF), 144.73 (d, *J* = 11.7 Hz), 137.51 (d, *J* = 9.8 Hz), 114.64, 105.67 (d, *J* = 29.1 Hz), 83.96, 38.92, 25.15, 18.88, 14.11.

##### *N*-[3-Fluoro-4-(4,4,5,5-tetramethyl-1,3,2-dioxaborolan-2-yl)phenyl]pentanamide (3bP)

White solid. Yield: 28%. ^1^H NMR (600 MHz, DMSO-*d*_6_) *δ* 10.16 (s, 1H, CONH), 7.57–7.50 (m, 2H, ArH), 7.28 (dd, *J* = 8.2, 1.8 Hz, 1H, ArH), 2.31 (t, *J* = 7.5 Hz, 2H, CH_2_), 1.55 (p, *J* = 7.5 Hz, 2H, CH_2_), 1.36–1.28 (m, 2H, CH_2_), 1.26 (s, 12H, CH_3_), 0.88 (t, *J* = 7.4 Hz, 3H, CH_3_). ^13^C NMR (151 MHz, DMSO-*d*_6_) *δ* 172.47, 167.40 (d, *J* = 247.8 Hz, CF), 144.75 (d, *J* = 11.7 Hz), 137.51 (d, *J* = 9.6 Hz), 114.63, 105.66 (d, *J* = 29.0 Hz), 83.96, 36.74, 27.56, 25.15, 22.32, 14.23.

##### *N*-[3-Fluoro-4-(4,4,5,5-tetramethyl-1,3,2-dioxaborolan-2-yl)phenyl]hexanamide (4bP)

Colourless oil. Yield: 51%. ^1^H NMR (600 MHz, DMSO-*d*_6_) *δ* 10.13 (s, 1H, CONH), 7.55–7.47 (m, 2H, ArH), 7.25 (dd, *J* = 8.2, 1.9 Hz, 1H, ArH), 2.27 (t, *J* = 7.5 Hz, 2H, CH_2_), 1.54 (m, 2H, CH_2_), 1.24 (s, 15H, CH_3_), 0.83 (t, *J* = 6.9 Hz, 3H, CH_3_). ^13^C NMR (151 MHz, DMSO-*d*_6_) *δ* 172.48, 167.40 (d, *J* = 247.3 Hz, CF), 144.75 (d, *J* = 12.1 Hz), 137.51 (d, *J* = 9.8 Hz), 114.62, 105.66 (d, *J* = 29.0 Hz), 83.95, 36.99, 31.38, 25.15, 25.11, 22.41, 14.37.

##### *N*-[3-Fluoro-4-(4,4,5,5-tetramethyl-1,3,2-dioxaborolan-2-yl)phenyl]heptanamide (5bP)

Colourless oil. Yield: 48%. ^1^H NMR (600 MHz, DMSO-*d*_6_) *δ* 10.16 (s, 1H, CONH), 7.58–7.50 (m, 2H, ArH), 7.27 (dd, *J* = 8.2, 1.9 Hz, 1H, ArH), 2.30 (t, *J* = 7.4 Hz, 2H, CH_2_), 1.56 (h, *J* = 7.4 Hz, 2H, CH_2_), 1.26 (s, 14H, CH_2_ and CH_3_), 1.32–1.21 (m, 4H, CH_2_), 0.85 (t, *J* = 7.0 Hz, 3H, CH_3_). ^13^C NMR (151 MHz, DMSO-*d*_6_) *δ* 172.47, 167.40 (d, *J* = 247.4 Hz, CF), 144.75 (d, *J* = 12.5 Hz), 137.51 (d, *J* = 9.3 Hz), 114.62, 105.66 (d, *J* = 29.2 Hz), 83.95, 37.03, 31.55, 28.83, 25.38, 25.15, 22.51, 14.44.

##### *N*-[3-Fluoro-4-(4,4,5,5-tetramethyl-1,3,2-dioxaborolan-2-yl)phenyl]cyclobutanecarboxamide (6bP)

White solid. Yield: 33%. ^1^H NMR (600 MHz, DMSO-*d*_6_) *δ* 10.01 (s, 1H, CONH), 7.59–7.50 (m, 2H, ArH), 7.29 (dd, *J* = 8.2, 1.9 Hz, 1H, ArH), 3.22 (p, *J* = 8.2 Hz, 1H, CH), 2.26–2.15 (m, 2H, CH_2_), 2.15–2.05 (m, 2H, CH_2_), 1.99–1.87 (m, 1H, CH_2_), 1.84–1.75 (m, 1H, CH_2_), 1.26 (s, 12H, CH_3_). ^13^C NMR (126 MHz, DMSO-*d*_6_) *δ* 173.70, 167.01 (d, *J* = 247.8 Hz, CF), 144.45 (d, *J* = 11.9 Hz), 137.14 (d, *J* = 9.9 Hz), 114.36, 105.39 (d, *J* = 29.1 Hz), 83.60, 39.83, 24.80, 24.71, 17.86.

##### *N*-[3-Fluoro-4-(4,4,5,5-tetramethyl-1,3,2-dioxaborolan-2-yl)phenyl]cyclopentanecarboxamide (7bP)

White solid. Yield: 14%. ^1^H NMR (600 MHz, DMSO-*d*_6_) *δ* 10.15 (s, 1H, CONH), 7.58–7.50 (m, 2H, ArH), 7.30 (dd, *J* = 8.2, 1.9 Hz, 1H, ArH), 2.76 (p, *J* = 7.8 Hz, 1H, CH), 1.93–1.67 (m, 4H, CH_2_), 1.66–1.47 (m, 4H, CH_2_), 1.26 (s, 12H, CH_3_). ^13^C NMR (151 MHz, DMSO-*d*_6_) *δ* 175.56, 167.39 (d, *J* = 247.7 Hz, CF), 144.88 (d, *J* = 11.6 Hz), 137.48 (d, *J* = 9.7 Hz), 114.71, 105.74 (d, *J* = 29.0 Hz), 83.95, 45.93, 30.54, 26.17, 25.15.

##### *N*-[4-(4,4,5,5-Tetramethyl-1,3,2-dioxaborolan-2-yl)-3-(trifluoromethyl)phenyl]isobutyramide (1cP)

White solid. Yield: 48%. ^1^H NMR (600 MHz, DMSO-*d*_6_) *δ* 10.20 (s, 1H, CONH), 8.09 (d, *J* = 2.0 Hz, 1H, ArH), 7.82 (dd, *J* = 8.2, 2.0 Hz, 1H, ArH), 7.67 (d, *J* = 8.2 Hz, 1H, ArH), 2.59 (m, 1H, CH), 1.28 (s, 12H, CH_3_), 1.10 (d, *J* = 6.7 Hz, 6H, CH_3_). ^13^C NMR (151 MHz, DMSO-*d*_6_) *δ* 176.48, 142.09, 136.82, 133.77 (q, *J* = 30.5 Hz), 124.66 (q, *J* = 273.2 Hz, CF_3_), 121.45, 116.01 (q, *J* = 5.6 Hz), 84.52, 35.63, 24.90, 19.86.

##### *N*-[4-(4,4,5,5-Tetramethyl-1,3,2-dioxaborolan-2-yl)-3-(trifluoromethyl)phenyl]butyramide (2cP)

White solid. Yield: 74%. ^1^H NMR (600 MHz, DMSO-*d*_6_) *δ* 10.24 (s, 1H, CONH), 8.08 (d, *J* = 2.0 Hz, 1H, ArH), 7.80 (dd, *J* = 8.2, 2.0 Hz, 1H, ArH), 7.67 (d, *J* = 8.2 Hz, 1H, ArH), 2.31 (t, *J* = 7.3 Hz, 2H, CH_2_), 1.65–1.56 (m, 2H, CH_2_), 1.27 (s, 12H, CH_3_), 0.90 (t, *J* = 7.4 Hz, 3H, CH_3_). ^13^C NMR (151 MHz, DMSO-*d*_6_) *δ* 172.42, 141.96, 136.85, 133.79 (q, *J* = 31.0 Hz), 125.56 (q, *J* = 274.9 Hz, CF_3_), 121.32, 115.88 (q, *J* = 5.4 Hz), 84.50, 38.89, 24.88, 18.86, 14.10.

##### *N*-[4-(4,4,5,5-Tetramethyl-1,3,2-dioxaborolan-2-yl)-3-(trifluoromethyl)phenyl]hexanamide (3cP)

Colourless oil. Yield: 70%. ^1^H NMR (600 MHz, DMSO-*d*_6_) *δ* 10.24 (s, 1H, CONH), 8.07 (d, *J* = 2.0 Hz, 1H, ArH), 7.80 (dd, *J* = 8.2, 2.0 Hz, 1H, ArH), 7.67 (d, *J* = 8.2 Hz, 1H, ArH), 2.32 (t, *J* = 7.4 Hz, 2H, CH_2_), 1.62–1.54 (m, 2H, CH_2_), 1.34–1.23 (m, 4H, CH_2_), 1.27 (s, 12H, CH_3_), 0.86 (t, *J* = 6.9 Hz, 3H, CH_3_). ^13^C NMR (151 MHz, DMSO-*d*_6_) *δ* 172.57, 141.98, 136.85, 133.79 (q, *J* = 30.9 Hz), 124.65 (q, *J* = 273.4 Hz, CF_3_), 121.30, 115.86 (q, *J* = 5.9 Hz), 84.50, 36.95, 31.37, 25.09, 24.88, 22.41, 14.35.

##### *N*-[4-(4,4,5,5-Tetramethyl-1,3,2-dioxaborolan-2-yl)-3-(trifluoromethyl)phenyl]cyclobutanecarboxamide (4cP)

White solid. Yield: 71%. ^1^H NMR (600 MHz, DMSO-*d*_6_) *δ* 10.09 (s, 1H, CONH), 8.09 (d, *J* = 2.0 Hz, 1H, ArH), 7.82 (dd, *J* = 8.2, 2.0 Hz, 1H, ArH), 7.67 (d, *J* = 8.2 Hz, 1H, ArH), 3.23 (p, *J* = 8.5 Hz, 1H, CH), 2.27–2.17 (m, 2H, CH_2_), 2.17–2.07 (m, 2H, CH_2_), 1.99–1.88 (m, 1H, CH_2_), 1.85–1.75 (m, 1H, CH_2_), 1.27 (s, 12H, CH_3_). ^13^C NMR (151 MHz, DMSO-*d*_6_) *δ* 174.17, 142.04, 136.83, 133.78 (q, *J* = 31.0 Hz), 124.66 (q, *J* = 271.9 Hz, CF_3_), 121.41, 116.00 (q, *J* = 5.6 Hz), 84.50, 40.18, 25.06, 24.89, 18.21.

##### *N*-[4-(4,4,5,5-Tetramethyl-1,3,2-dioxaborolan-2-yl)-3-(trifluoromethyl)phenyl]cyclopentanecarboxamide (5cP)

White solid. Yield: 76%. ^1^H NMR (600 MHz, DMSO-*d*_6_) *δ* 10.24 (s, 1H, CONH), 8.10 (d, *J* = 2.0 Hz, 1H, ArH), 7.81 (dd, *J* = 8.2, 2.0 Hz, 1H, ArH), 7.67 (d, *J* = 8.2 Hz, 1H, ArH), 2.78 (p, *J* = 7.9 Hz, 1H, CH), 1.89–1.80 (m, 2H, CH_2_), 1.77–1.61 (m, 4H, CH_2_), 1.60–1.50 (m, 2H, CH_2_), 1.27 (s, 12H, CH_3_). ^13^C NMR (151 MHz, DMSO-*d*_6_) *δ* 175.66, 142.10, 136.82, 133.78 (q, *J* = 31.0 Hz), 124.66 (q, *J* = 273.6 Hz, CF_3_), 121.39, 115.97 (q, *J* = 5.7 Hz), 84.49, 45.89, 30.52, 26.19, 24.88.

##### 4-(Pyrazine-2-carboxamido)phenylboronic acid (1a)

White solid. Yield: 79%. mp 253.3–255.4 °C. ^1^H NMR (600 MHz, DMSO-*d*_6_) *δ* 10.69 (s, 1H, CONH), 9.29 (d, *J* = 1.5 Hz, 1H, PzH), 8.92 (d, *J* = 2.6 Hz, 1H, PzH), 8.80 (m, 1H, PzH), 7.96 (bs, 2H, BO_2_H_2_), 7.86 (d, *J* = 8.1 Hz, 2H, ArH), 7.78 (d, *J* = 8.3 Hz, 2H, ArH). ^13^C NMR (151 MHz, DMSO-*d*_6_) *δ* 162.25, 148.26, 145.59, 144.60, 143.76, 140.28, 135.30, 119.78. ^11^B NMR (160 MHz, DMSO-*d*_6_) *δ* 28.19. IR (ATR-Ge, cm^−1^): 3338 (*ν* O–H), 3128 (*ν* C–H arom.), 1678 (*δ* C=O), 1534 (*δ* CONH), 1347 (*ν* B–O), 629 (*δ* BO_2_). Anal. calcd. for C_11_H_10_BN_3_O_3_ (MW 243.03): C, 54.36; H, 4.15; N, 17.29. Found: C, 54.06; H, 3.93; N, 17.01. MS: [M + H]^+^ = 244.0 (exact mass 243.08).

##### 4-(5-Methylpyrazine-2-carboxamido)phenylboronic acid (2a)

White solid. Yield: 73%. mp 255.2–256.9 °C. ^1^H NMR (500 MHz, DMSO-*d*_6_) *δ* 10.60 (s, 1H, CONH), 9.15 (d, *J* = 1.4 Hz, 1H, PzH), 8.68 (d, *J* = 1.4 Hz, 1H, PzH), 7.87–7.82 (m, 2H, ArH), 7.80–7.75 (m, 2H, ArH), 2.62 (s, 3H, CH_3_). ^13^C NMR (126 MHz, DMSO-*d*_6_) *δ* 162.00, 157.39, 143.20, 142.90, 142.46, 139.99, 134.91, 119.35, 21.61. ^11^B NMR (160 MHz, DMSO-*d*_6_) *δ* 27.80. IR (ATR-Ge, cm^−1^): 3341 (*ν* O–H), 1684 (*δ* C=O), 1536 (*δ* CONH), 1351 (*ν* B–O), 648 (*δ* BO_2_). Anal. calcd. for C_12_H_12_BN_3_O_3_ (MW 257.06): C, 56.07; H, 4.71; N, 16.35. Found: C, 55.67; H, 4.94; N, 15.98. MS: [M + H]^+^ = 258.0 (exact mass 257.10).

##### 4-(3-Methylpyrazine-2-carboxamido)phenylboronic acid (3a)

Beige solid. Yield: 42%. mp 258–258.9 °C. ^1^H NMR (600 MHz, DMSO-*d*_6_) *δ* 10.62 (s, 1H, CONH), 8.72 (d, *J* = 2.6 Hz, 1H, PzH), 8.60 (d, *J* = 2.6 Hz, 1H, PzH), 7.95 (bs, 2H, BO_2_H_2_), 7.81–7.73 (m, 4H, ArH), 2.75 (s, 3H, CH_3_). ^13^C NMR (151 MHz, DMSO-*d*_6_) *δ* 164.40, 153.59, 146.21, 145.96, 141.31, 140.66, 135.38, 119.29, 22.97. ^11^B NMR (160 MHz, DMSO-*d*_6_) *δ* 29.45. IR (ATR-Ge, cm^−1^): 3346 (*ν* O–H), 3176 (*ν* C–H arom.), 1679 (*δ* C=O), 1527 (*δ* CONH), 1348 (*ν* B–O), 638 (*δ* BO_2_). Anal. calcd. for C_12_H_12_BN_3_O_3_ (MW 257.06): C, 56.07; H, 4.71; N, 16.35. Found: C, 55.67; H, 4.38; N, 15.99. MS: [M + H]^+^ = 257.8 (exact mass 257.10).

##### 4-(6-Chloropyrazine-2-carboxamido)phenylboronic acid (4a)

Light yellow solid. Yield: 80%. mp 263.8–265.2 °C. ^1^H NMR (600 MHz, DMSO-*d*_6_) *δ* 10.61 (bs, 1H, CONH), 9.22 (s, 1H, PzH), 9.05 (s, 1H, PzH), 7.97 (s, 2H, BO_2_H_2_), 7.82 (d, *J* = 8.5 Hz, 2H, ArH), 7.79 (d, *J* = 8.5 Hz, 2H, ArH). ^13^C NMR (151 MHz, DMSO-*d*_6_) *δ* 161.22, 147.97, 147.42, 145.71, 142.89, 140.08, 135.27, 130.57, 120.04. ^11^B NMR (160 MHz, DMSO-*d*_6_) *δ* 27.20. IR (ATR-Ge, cm^−1^): 3356 (*ν* O–H), 1692 (*δ* C=O), 1532 (*δ* CONH), 1337 (*ν* B–O), 634 (*δ* BO_2_). Anal. calcd. for C_11_H_9_BClN_3_O_3_ (MW 277.47): C, 47.62; H, 3.27; N, 15.14. Found: C, 47.26; H, 3.58; N, 14.86. MS: [M + H]^+^ = 278.0 (exact mass 277.04).

##### 4-(5-Chloropyrazine-2-carboxamido)phenylboronic acid (5a)

White solid. Yield: 89%. mp 235.8–237.8 °C. ^1^H NMR (500 MHz, DMSO-*d*_6_) *δ* 10.70 (bs, 1H, CONH), 9.07 (s, 1H, PzH), 8.87 (s, 1H, PzH), 7.80 (d, *J* = 8.1 Hz, 2H, ArH), 7.76 (d, *J* = 8.1 Hz, 2H, ArH). ^13^C NMR (126 MHz, DMSO-*d*_6_) *δ* 161.39, 151.44, 144.31, 144.07, 143.45, 139.88, 135.21, 119.74. ^11^B NMR (160 MHz, DMSO-*d*_6_) *δ* 28.74. IR (ATR-Ge, cm^−1^): 3364 (*ν* O–H), 1677 (*δ* C=O), 1527 (*δ* CONH), 1344 (*ν* B–O), 633 (*δ* BO_2_). Anal. calcd. for C_11_H_9_BClN_3_O_3_ (MW 277.47): C, 47.62; H, 3.27; N, 15.14. Found: C, 47.98; H, 3.27; N, 14.96. MS: [M + H]^+^ = 277.9 (exact mass 277.04).

##### 4-(3-Aminopyrazine-2-carboxamido)phenylboronic acid (6a)

Yellow solid. Yield: 74%. mp 248.4–249.3 °C. ^1^H NMR (600 MHz, DMSO-*d*_6_) *δ* 10.46 (s, 1H, CONH), 8.28 (d, *J* = 2.3 Hz, 1H, PzH), 7.93 (s, 2H, BO_2_H_2_), 7.91 (d, *J* = 2.3 Hz, 1H, PzH), 7.79 (d, *J* = 8.3 Hz, 2H, ArH), 7.76 (d, *J* = 8.3 Hz, 2H, ArH), 7.60–7.56 (bs, 2H, NH_2_). ^13^C NMR (151 MHz, DMSO-*d*_6_) *δ* 165.09, 156.00, 147.96, 140.29, 135.27, 131.51, 125.83, 119.58. ^11^B NMR (160 MHz, DMSO-*d*_6_) *δ* 31.70. IR (ATR-Ge, cm^−1^): 3335 (*ν* O–H), 1666 (*δ* C=O), 1539 (*δ* CONH), 1356 (*ν* B–O), 641 (*δ* BO_2_). Anal. calcd. for C_11_H_11_BN_4_O_3_ (MW 258.04): C, 51.2; H, 4.3; N, 21.71. Found: C, 50.87; H, 4.36; N, 21.4. MS: [M + H]^+^ = 258.9 (exact mass 258.09).

##### 4-(3-Oxo-3,4-dihydropyrazine-2-carboxamido)phenylboronic acid (7a)

Yellow solid. Yield: 72%. mp 276.3–277.6 °C. ^1^H NMR (600 MHz, DMSO-*d*_6_) *δ* 13.21 (bs, 1H, LmH), 11.41 (bs, 1H, CONH), 7.95 (s, 2H, BO_2_H_2_), 7.81 (s, 1H, PzH), 7.78 (d, *J* = 8.0 Hz, 2H, ArH), 7.70 (s, 1H, PzH), 7.67 (d, *J* = 8.0 Hz, 2H, ArH). ^11^B NMR (160 MHz, DMSO-*d*_6_) *δ* 30.98. IR (ATR-Ge, cm^−1^): 3321 (*ν* O–H), 3100 (*ν* C–H arom.), 1696 (*δ* C=O), 1541 (*δ* CONH), 1322 (*ν* B–O), 637 (*δ* BO_2_). Anal. calcd. for C_11_H_10_BN_3_O_4_ (MW 259.03): C, 51.01; H, 3.89; N, 16.22. Found: C, 50.68; H, 3.68; N, 15.9. MS: [M + H]^+^ = 260.3 (exact mass 259.08).

##### 4-(3-Amino-6-chloropyrazine-2-carboxamido)phenylboronic acid (8a)

Yellow solid. Yield: 81%. mp 247.7–248.9 °C. ^1^H NMR (600 MHz, DMSO-*d*_6_) *δ* 10.25 (s, 1H, CONH), 8.37 (s, 1H, PzH), 7.94 (bs, 2H, BO_2_H_2_), 7.79–7.74 (m, 4H, ArH), 7.72 (bs, 2H, NH_2_). ^13^C NMR (151 MHz, DMSO-*d*_6_) *δ* 164.10, 154.80, 147.29, 140.07, 135.23, 132.22, 130.14, 124.43, 119.94. ^11^B NMR (160 MHz, DMSO-*d*_6_) *δ* 28.26. IR (ATR-Ge, cm^−1^): 3347 (*ν* O–H), 1665 (*δ* C=O), 1528 (*δ* CONH), 1340 (*ν* B–O), 633 (*δ* BO_2_). Anal. calcd. for C_11_H_10_BClN_4_O_3_ (MW 292.49): C, 45.17; H, 3.45; N, 19.16. Found: C, 44.77; H, 3.57; N, 18.82. MS: [M + H]^+^ = 293.2 (exact mass 292.05).

##### 4-(5-Chloropyrazine-2-carboxamido)-2-fluorophenylboronic acid (9a)

White solid. Yield: 92%. mp 237.6–239.2 °C. ^1^H NMR (600 MHz, DMSO-*d*_6_) *δ* 10.93 (s, 1H, CONH), 9.11 (s, 1H, PzH), 8.93 (s, 1H, PzH), 8.07 (bs, 2H, BO_2_H_2_), 7.75–7.70 (m, 1H, ArH), 7.69–7.65 (m, 1H, ArH), 7.60–7.53 (m, 1H, ArH). ^13^C NMR (151 MHz, DMSO-*d*_6_) *δ* 166.23 (d, *J* = 243.9 Hz), 161.78, 151.63, 144.72, 144.11, 143.48, 141.68 (d, *J* = 11.5 Hz), 136.33 (d, *J* = 10.4 Hz), 116.07, 107.25 (d, *J* = 29.9 Hz). ^11^B NMR (160 MHz, DMSO-*d*_6_) *δ* 29.40. IR (ATR-Ge, cm^−1^): 3339 (*ν* O–H), 1675 (*δ* C=O), 1528 (*δ* CONH), 1346 (*ν* B–O), 641 (*δ* BO_2_). HPLC purity 95.8%. MS: [M + H]^+^ = 295.9 (exact mass 295.03).

##### 4-[(5-Chloropyrazine-2-carboxamido)methyl]phenylboronic acid (10a)

White solid. Yield: 94%. mp 246–248.7 °C. ^1^H NMR (600 MHz, DMSO-*d*_6_) *δ* 9.48 (t, *J* = 6.4 Hz, 1H, CONH), 9.08–8.93 (m, 1H, PzH), 8.87 (s, 1H, PzH), 7.95 (s, 2H, BO_2_H_2_), 7.72 (d, *J* = 7.6 Hz, 2H, ArH), 7.27 (d, *J* = 7.6 Hz, 2H, ArH), 4.49 (d, *J* = 6.4 Hz, 2H, CH_2_). ^13^C NMR (151 MHz, DMSO-*d*_6_) *δ* 162.73, 151.30, 144.14, 144.04, 143.69, 141.46, 134.67, 133.10, 126.88, 43.03. ^11^B NMR (160 MHz, DMSO-*d*_6_) *δ* 28.26. IR (ATR-Ge, cm^−1^): 3293 (*ν* O–H), 1657 (*δ* C=O), 1525 (*δ* CONH), 1343 (*ν* B–O), 644 (*δ* BO_2_). Anal. calcd. for C_12_H_11_BClN_3_O_3_ (MW 291.5): C, 49.45; H, 3.8; N, 14.42. Found: C, 49.80; H, 3.83; N, 14.1. MS: [M + H]^+^ = 292.9 (exact mass 291.06).

##### 4-(5-Hydroxypyrazine-2-carboxamido)phenylboronic acid (11a)

Brown solid. Yield: 88%. mp 247.6–249.6 °C. ^1^H NMR (600 MHz, DMSO-*d*_6_) *δ* 12.87 (bs, 1H, OH), 10.12 (s, 1H, CONH), 8.10 (s, 1H, PzH), 8.03 (d, *J* = 1.2 Hz, 1H, PzH), 7.91 (bs, 2H, BO_2_H_2_), 7.78 (d, *J* = 8.3 Hz, 2H, ArH), 7.74 (d, *J* = 8.3 Hz, 2H, ArH). ^13^C NMR (151 MHz, DMSO-*d*_6_) *δ* 161.71, 156.78, 146.84, 140.61, 135.24, 131.39, 129.62, 126.90, 119.47. ^11^B NMR (160 MHz, DMSO-*d*_6_) *δ* 28.05. IR (ATR-Ge, cm^−1^): 3341 (*ν* O–H), 1650 (*δ* C=O), 1526 (*δ* CONH), 1326 (*ν* B–O), 615 (*δ* BO_2_). Anal. calcd. for C_11_H_10_BN_3_O_4_ (MW 259.03): C, 51.01; H, 3.89; N, 16.22. Found: C, 50.63; H, 3.78; N, 15.86. [M + H]^+^ = 259.9 (exact mass 259.08).

##### 4-[(5-Chloropyrazin-2-yl)carbamoyl]phenylboronic acid (12a)

White solid. Yield: 66%. mp 281.9–283.1 °C. ^1^H NMR (600 MHz, DMSO-*d*_6_) *δ* 11.29 (s, 1H, CONH), 9.25 (d, *J* = 1.4 Hz, 1H, PzH), 8.62 (d, *J* = 1.4 Hz, 1H, PzH), 8.25 (bs, 2H, BO_2_H_2_), 8.00–7.97 (m, 2H, ArH), 7.92–7.89 (m, 2H, ArH). ^13^C NMR (151 MHz, DMSO-*d*_6_) *δ* 166.91, 148.63, 142.65, 142.35, 139.37, 136.84, 134.88, 134.54, 127.62. ^11^B NMR (160 MHz, DMSO-*d*_6_) *δ* 27.20. IR (ATR-Ge, cm^−1^): 3566 (*ν* N–H amide), 3305 (*ν* O–H), 3063 (*ν* C–H arom.), 1670 (*δ* C=O), 1542 (*δ* CONH), 1336 (*ν* B–O), 857 (*ν* B–O). Anal. calcd. for C_11_H_9_BClN_3_O_3_ (MW 277.47): C, 47.62 H, 3.27; N, 15.14. Found: C, 47.91; H, 3.18; N, 14.81. MS: [M + H]^+^ = 278.0 (exact mass 277.04).

##### 4-(Picolinamido)phenylboronic acid (13a)

White solid. Yield: 71%. mp 254.1–255.7 °C. ^1^H NMR (600 MHz, DMSO-*d*_6_) *δ* 10.60 (s, 1H. CONH), 8.76–8.71 (m, 1H, PyH), 8.16 (d, *J* = 7.8 Hz, 1H, PyH), 8.06 (td, *J* = 7.7, 1.8 Hz, 1H, PyH), 7.94 (s, 2H, BO_2_H_2_), 7.86 (d, *J* = 8.2 Hz, 2H, ArH), 7.78 (d, *J* = 8.2 Hz, 2H, ArH), 7.67 (dd, *J* = 7.8, 4.5 Hz, 1H, PyH). ^13^C NMR (151 MHz, DMSO-*d*_6_) *δ* 163.01, 150.39, 148.98, 140.45, 138.71, 135.33, 127.50, 122.92, 119.48. ^11^B NMR (160 MHz, DMSO-*d*_6_) *δ* 29.65. IR (ATR-Ge, cm^−1^): 3310 (*ν* O–H), 1679 (*δ* C=O), 1541 (*δ* CONH), 1342 (*ν* B–O), 640 (*δ* BO_2_). Anal. calcd. for C_12_H_11_BN_2_O_3_ (MW 242.04): C, 59.55; H, 4.58; N, 11.57. Found: C, 59.21; H, 4.39; N, 11.4. MS: [M + H]^+^ = 243.0 (exact mass 242.09).

##### 4-[6-(Trifluoromethyl)nicotinamido]phenylboronic acid (14a)

White solid. Yield: 97%. mp 246.2–247.5 °C. ^1^H NMR (600 MHz, DMSO-*d*_6_) *δ* 10.64 (s, 1H, CONH), 9.24 (d, *J* = 2.2 Hz, 1H, PyH), 8.56 (dd, *J* = 8.1, 2.2 Hz, 1H, PyH), 8.09 (d, *J* = 8.1 Hz, 1H, PyH), 7.96 (s, 2H, BO_2_H_2_), 7.80 (d, *J* = 8.3 Hz, 2H, ArH), 7.73 (d, *J* = 8.3 Hz, 2H, ArH). ^13^C NMR (151 MHz, DMSO-*d*_6_) *δ* 163.47, 149.86, 148.70 (q, *J* = 34.1 Hz, PyC), 140.72, 138.42, 135.38, 134.35, 130.35, 121.93 (q, *J* = 274.2 Hz, CF_3_), 121.14, 119.65. ^11^B NMR (160 MHz, DMSO-*d*_6_) *δ* 28.45. IR (ATR-Ge, cm^−1^): 3303 (*ν* O–H), 1655 (*δ* C=O), 1524 (*δ* CONH), 1330 (*ν* B–O), 640 (*δ* BO_2_). HPLC purity 95.5%. [M + H]^+^ = 311.0 (exact mass 310.07).

##### 4-[5-(Trifluoromethyl)picolinamido]phenylboronic acid (15a)

White solid. Yield: 94%. mp 262.3–264.6 °C. ^1^H NMR (600 MHz, DMSO-*d*_6_) *δ* 10.75 (s, 1H, CONH), 9.11–9.09 (m, 1H, PyH), 8.53–8.46 (m, 1H, PyH), 8.38–8.30 (m, 1H, PyH), 7.96 (s, 2H, BO_2_H_2_), 7.90–7.83 (m, 2H, ArH), 7.82–7.76 (m, 2H, ArH). ^13^C NMR (151 MHz, DMSO-*d*_6_) *δ* 161.98, 154.01, 145.85, 140.21, 136.37, 135.31, 130.36, 127.99 (q, *J* = 32.6 Hz, PyC), 123.91 (q, *J* = 272.5 Hz, CF_3_), 123.34, 119.76. ^11^B NMR (160 MHz, DMSO-*d*_6_) *δ* 29.99. IR (ATR-Ge, cm^−1^): 3361 (*ν* O–H), 1678 (*δ* C=O), 1532 (*δ* CONH), 1323 (*ν* B–O), 630 (*δ* BO_2_). HPLC purity 96.7%. MS: [M + H]^+^ = 311.0 (exact mass 310.07).

##### 4-(5-Chlorothiophene-2-carboxamido)phenylboronic acid (16a)

Beige solid. Yield: 87%. mp 246.6–248.4 °C. ^1^H NMR (600 MHz, DMSO-*d*_6_) *δ* 10.29 (s, 1H, CONH), 7.93 (s, 2H, BO_2_H_2_), 7.92 (d, *J* = 4.1 Hz, 1H, TfH), 7.79–7.73 (m, 2H, ArH), 7.69–7.63 (m, 2H, ArH), 7.26 (d, *J* = 4.1 Hz, 1H, TfH). ^13^C NMR (151 MHz, DMSO-*d*_6_) *δ* 159.37, 140.55, 139.73, 135.34, 134.54, 129.73, 128.82, 119.64. ^11^B NMR (160 MHz, DMSO-*d*_6_) *δ* 30.72. IR (ATR-Ge, cm^−1^): 3318 (*ν* O–H), 1635 (*δ* C=O), 1536 (*δ* CONH), 1327 (*ν* B–O), 624 (*δ* BO_2_). Anal. calcd. for C_11_H_9_BClNO_3_S (MW 281.52): C, 46.93; H, 3.22; N, 4.98. Found: C, 47.01; H, 3.21; N, 4.91. MS: [M + H]^+^ = 281.9 (exact mass 281.01).

##### 4-(4-Chlorobenzamido)phenylboronic acid (17a)

Beige solid. Yield: 91%. mp 261.2–264.1 °C. ^1^H NMR (600 MHz, DMSO-*d*_6_) *δ* 10.31 (s, 1H, CONH), 8.02–7.94 (m, 2H, ArH), 7.93 (s, 2H, BO_2_H_2_), 7.81–7.75 (m, 2H, ArH), 7.75–7.69 (m, 2H, ArH), 7.64–7.56 (m, 2H, ArH). ^13^C NMR (151 MHz, DMSO-*d*_6_) *δ* 165.03, 141.17, 136.96, 135.27, 134.20, 130.19, 129.77, 129.00, 119.65. ^11^B NMR (160 MHz, DMSO-*d*_6_) *δ* 28.17. IR (ATR-Ge, cm^−1^): 3300 (*ν* O–H), 1644 (*δ* C=O), 1525 (*δ* CONH), 1323 (*ν* B–O), 625 (*δ* BO_2_). Anal. calcd. for C_13_H_11_BClNO_3_ (MW 275.5): C, 56.68; H, 4.02; N, 5.08. Found: C, 56.86; H, 3.78; N, 4.95. MS: [M + H]^+^ = 275.9 (exact mass 275.05).

##### 4-(Quinoxaline-2-carboxamido)phenylboronic acid (18a)

Brown solid. Yield: 86%. mp 262.9–265.2 °C. ^1^H NMR (500 MHz, DMSO-*d*_6_) *δ* 10.80 (s, 1H, CONH), 9.54 (s, 1H, QxH), 8.32–8.26 (m, 1H, QxH), 8.24–8.18 (m, 1H, QxH), 8.00 (bs, 2H, BO_2_H_2_), 7.91 (d, *J* = 8.0 Hz, 2H, ArH), 7.83 (d, *J* = 8.0 Hz, 2H, ArH). ^13^C NMR (126 MHz, DMSO-*d*_6_) *δ* 162.19, 144.85, 144.12, 143.13, 139.93, 139.82, 135.01, 132.29, 131.56, 129.74, 129.30, 119.42. ^11^B NMR (160 MHz, DMSO-*d*_6_) *δ* 28.08. IR (ATR-Ge, cm^−1^): 3358 (*ν* O–H), 1679 (*δ* C=O), 1532 (*δ* CONH), 1346 (*ν* B–O), 621 (*δ* BO_2_). Anal. calcd. for C_15_H_12_BN_3_O_3_ (MW 293.09): C, 61.47; H, 4.13; N, 14.34. Found: C, 61.18; H, 4.03; N, 14.24. MS: [M + H]^+^ = 294.1 (exact mass 293.10).

##### 4-(3-Oxo-3,4-dihydroquinoxaline-2-carboxamido)phenylboronic acid (19a)

Yellow solid. Yield: 89%. mp 283.1–284.9 °C. ^1^H NMR (600 MHz, DMSO-*d*_6_) *δ* 12.92 (bs, 1H, LmH), 11.09 (s, 1H, CONH), 7.97 (s, 2H, BO_2_H_2_), 7.89 (dd, *J* = 8.3, 1.4 Hz, 1H, QxH), 7.80 (d, *J* = 8.1 Hz, 2H, ArH), 7.68–7.65 (m, 2H, ArH), 7.65–7.62 (m, 1H, QxH), 7.39 (ddd, *J* = 8.5, 3.8, 2.2 Hz, 2H, QxH). ^13^C NMR (151 MHz, DMSO-*d*_6_) *δ* 162.12, 154.40, 152.12, 140.46, 135.63, 133.05, 132.57, 131.75, 129.87, 124.60, 118.85, 116.24. ^11^B NMR (160 MHz, DMSO-*d*_6_) *δ* 27.76. IR (ATR-Ge, cm^−1^): 3419 (*ν* O–H), 3041 (*ν* C–H arom.), 1700 (*δ* C=O), 1551 (*δ* CONH), 1322 (*ν* B–O), 628 (*δ* BO_2_). Anal. calcd. for C_15_H_12_BN_3_O_4_ (MW 309.09): C, 58.29; H, 3.91; N, 13.6. Found: C, 58.21; H, 3.67; N, 13.52. MS: [M + H]^+^ = 310.4 (exact mass 309.09).

##### 4-(Isoquinoline-1-carboxamido)phenylboronic acid (20a)

Yellow solid. Yield: 74%. mp 259.9–261.3 °C. ^1^H NMR (600 MHz, DMSO-*d*_6_) *δ* 10.82 (s, 1H, CONH), 8.82 (d, *J* = 8.6 Hz, 1H, ArH), 8.62 (d, *J* = 5.7 Hz, 1H, ArH), 8.09 (t, *J* = 6.7 Hz, 2H, ArH), 7.89–7.83 (m, 3H, ArH), 7.81 (d, *J* = 8.1 Hz, 2H, ArH), 7.77 (t, *J* = 7.8 Hz, 1H, ArH), 6.54 (s, 2H, BO_2_H_2_). ^13^C NMR (151 MHz, DMSO-*d*_6_) *δ* 165.17, 151.90, 141.26, 140.87, 137.22, 135.43, 131.52, 129.96, 129.28, 127.84, 126.85, 125.96, 124.17, 119.31. ^11^B NMR (160 MHz, DMSO-*d*_6_) *δ* 29.22. IR (ATR-Ge, cm^−1^): 3320 (*ν* O–H), 3053 (*ν* C–H arom.), 1669 (*δ* C=O), 1526 (*δ* CONH), 1361 (*ν* B–O), 647 (*δ* BO_2_). Anal. calcd. for C_16_H_13_BN_2_O_3_ (MW 292.1): C, 65.79; H, 4.49; N, 9.59. Found: C, 65.59; H, 4.46; N, 9.51. [M + H]^+^ = 293.0 (exact mass 292.10).

##### (2-Fluoro-4-isobutyramidophenyl)boronic acid (1b)

White solid. Yield: 53%. mp 191.7–196.5 °C. ^1^H NMR (600 MHz, DMSO-*d*_6_) *δ* 10.00 (s, 1H, CONH), 7.95 (bs, 2H, BO_2_H_2_), 7.54–7.45 (m, 2H, ArH), 7.29–7.21 (m, 1H, ArH), 2.57 (dt, *J* = 13.7, 6.9 Hz, 1H, CH), 1.09 (d, *J* = 6.7 Hz, 6H, CH_3_). ^13^C NMR (151 MHz, DMSO-*d*_6_) *δ* 176.17, 166.58 (d, *J* = 242.9 Hz, CF), 143.16 (d, *J* = 11.7 Hz), 136.46 (d, *J* = 10.4 Hz), 114.51, 105.74 (d, *J* = 30.0 Hz), 35.55, 19.93. ^11^B NMR (193 MHz, DMSO-*d*_6_) *δ* 26.95. IR (ATR-Ge, cm^−1^): 3251 (*ν* O–H), 1680 (*δ* C=O), 1596 (*δ* CONH), 1357 (*ν* B–O), 631 (*δ* BO_2_). HPLC purity 99.7%. MS: [M + H]^+^ = 226.0 (exact mass 225.1).

##### (4-Butyramido-2-fluorophenyl)boronic acid (2b)

White solid. Yield: 55%. mp 200.3–202.6 °C. ^1^H NMR (600 MHz, DMSO-*d*_6_) *δ* 10.05 (s, 1H, CONH), 7.96 (bs, 2H, BO_2_H_2_), 7.49 (dd, *J* = 9.5, 6.3 Hz, 2H, ArH), 7.23 (t, *J* = 6.5 Hz, 1H, ArH), 2.28 (t, *J* = 7.5 Hz, 2H, CH_2_), 1.59 (m, 2H, CH_2_), 0.90 (t, *J* = 7.5 Hz, 3H, CH_3_). ^13^C NMR (151 MHz, DMSO-*d*_6_) *δ* 172.11, 166.57 (d, *J* = 242.8 Hz, CF), 143.02 (d, *J* = 11.9 Hz), 136.46 (d, *J* = 10.3 Hz), 114.40, 106.05, 105.64 (d, *J* = 30.3 Hz), 38.89, 18.94, 14.13. ^11^B NMR (193 MHz, DMSO-*d*_6_) *δ* 26.87. IR (ATR-Ge, cm^−1^): 3300 (*ν* O–H), 1669 (*δ* C=O), 1515 (*δ* CONH), 1316 (*ν* B–O), 645 (*δ* BO_2_). HPLC purity 99.9%. MS: [M + H]^+^ = 226.0 (exact mass 225.1).

##### (2-Fluoro-4-pentanamidophenyl)boronic acid (3b)

White solid. Yield: 68%. mp 199.5–201.7 °C. ^1^H NMR (600 MHz, DMSO-*d*_6_) *δ* 10.05 (s, 1H, CONH), 7.95 (bs, 2H, BO_2_H_2_), 7.53–7.45 (m, 2H, ArH), 7.22 (dd, *J* = 8.1, 1.9 Hz, 1H, ArH), 2.30 (t, *J* = 7.5 Hz, 2H, CH_2_), 1.56 (p, *J* = 7.4 Hz, 2H, CH_2_), 1.31 (h, *J* = 7.4 Hz, 2H, CH_2_), 0.88 (t, *J* = 7.4 Hz, 3H, CH_3_). ^13^C NMR (151 MHz, DMSO-*d*_6_) *δ* 172.25, 166.57 (d, *J* = 242.9 Hz, CF), 143.03 (d, *J* = 11.6 Hz), 136.46 (d, *J* = 10.8 Hz), 115.92, 114.39, 105.63 (d, *J* = 29.6 Hz), 36.70, 27.62, 22.33, 14.25. ^11^B NMR (193 MHz, DMSO-*d*_6_) *δ* 26.64. IR (ATR-Ge, cm^−1^): 3311 (*ν* O–H), 1670 (*δ* C=O), 1589 (*δ* CONH), 1338 (*ν* B–O), 642 (*δ* BO_2_). HPLC purity 99.0%. MS: [M + H]^+^ = 240.1, [M–H]^−^ = 238.0 (exact mass 239.1).

##### (2-Fluoro-4-hexanamidophenyl)boronic acid (4b)

White solid. Yield: 86%. mp 199.9–205.2 °C. ^1^H NMR (600 MHz, DMSO-*d*_6_) *δ* 10.04 (s, 1H, CONH), 7.95 (bs, 2H, BO_2_H_2_), 7.53–7.45 (m, 2H, ArH), 7.26–7.18 (m, 1H, ArH), 2.29 (t, *J* = 7.5 Hz, 2H, CH_2_), 1.57 (p, *J* = 7.6 Hz, 2H, CH_2_), 1.34–1.26 (m, 2H, CH_2_), 1.28–1.22 (m, 2H, CH_2_), 0.86 (t, *J* = 6.9 Hz, 3H, CH_3_). ^13^C NMR (151 MHz, DMSO-*d*_6_) *δ* 172.25, 166.58 (d, *J* = 242.9 Hz, CF), 143.03 (d, *J* = 11.9 Hz), 136.46 (d, *J* = 10.4 Hz), 114.39, 105.63 (d, *J* = 29.5 Hz), 36.96, 31.40, 25.17, 22.42, 14.38. ^11^B NMR (193 MHz, DMSO-*d*_6_) *δ* 26.78. IR (ATR-Ge, cm^−1^): 3306 (*ν* O–H), 1668 (*δ* C=O), 1585 (*δ* CONH), 1340 (*ν* B–O), 644 (*δ* BO_2_). HPLC purity 99.0%. MS: [M + H]^+^ = 254.1 (exact mass 253.1).

##### (2-Fluoro-4-heptanamidophenyl)boronic acid (5b)

White solid. Yield: 82%. mp 200.8–203.4 °C. ^1^H NMR (500 MHz, DMSO-*d*_6_) *δ* 10.05 (s, 1H, CONH), 7.97 (bs, 2H, BO_2_H_2_), 7.53–7.45 (m, 2H, ArH), 7.25–7.19 (m, 1H, ArH), 2.29 (t, *J* = 7.4 Hz, 2H, CH_2_), 1.56 (p, *J* = 7.2 Hz, 2H, CH_2_), 1.33–1.21 (m, 6H, CH_2_), 0.88–0.82 (m, 3H, CH_3_). ^13^C NMR (126 MHz, DMSO-*d*_6_) *δ* 172.16, 166.48 (d, *J* = 243.2 Hz, CF), 142.95 (d, *J* = 11.8 Hz), 136.38 (d, *J* = 10.9 Hz), 114.29, 105.53 (d, *J* = 30.0 Hz), 36.91, 31.49, 28.77, 25.37, 22.44, 14.39. ^11^B NMR (193 MHz, DMSO-*d*_6_) *δ* 27.11. IR (ATR-Ge, cm^−1^): 3313 (*ν* O–H), 1666 (*δ* C=O), 1586 (*δ* CONH), 1342 (*ν* B–O), 644 (*δ* BO_2_). HPLC purity 98.8%. MS: [M + H]^+^ = 268.0 (exact mass 267.1).

##### 4-(Cyclobutanecarboxamido)-2-fluorophenylboronic acid (6b)

White solid. Yield: 63%. mp 198.5–200.5 °C. ^1^H NMR (500 MHz, DMSO-*d*_6_) *δ* 9.91 (s, 1H, CONH), 7.97 (bs, 2H, BO_2_H_2_), 7.55–7.45 (m, 2H, ArH), 7.27–7.21 (m, 1H, ArH), 3.27–3.16 (m, 1H, CH), 2.27–2.15 (m, 2H, CH_2_), 2.14–2.04 (m, 2H, CH_2_), 1.99–1.86 (m, 1H, CH_2_), 1.85–1.73 (m, 1H, CH_2_). ^13^C NMR (126 MHz, DMSO-*d*_6_) *δ* 173.50, 166.21 (d, *J* = 243.2 Hz, CF), 142.74 (d, *J* = 11.8 Hz), 136.11 (d, *J* = 10.8 Hz), 114.14, 105.38 (d, *J* = 30.0 Hz), 39.82, 24.75, 17.90. ^11^B NMR (193 MHz, DMSO-*d*_6_) *δ* 26.88. IR (ATR-Ge, cm^−1^): 3273 (*ν* O–H), 1655 (*δ* C=O), 1600 (*δ* CONH), 1353 (*ν* B–O), 643 (*δ* BO_2_). HPLC purity 99.8%. MS: [M + H]^+^ = 237.9, [M–H]^−^ = 235.7 (exact mass 237.1).

##### 4-(Cyclopentanecarboxamido)-2-fluorophenylboronic acid (7b)

White solid. Yield: 62%. mp 171.7–177.7 °C. ^1^H NMR (600 MHz, DMSO-*d*_6_) *δ* 10.04 (s, 1H, CONH), 7.94 (s, 2H, BO_2_H_2_), 7.54–7.44 (m, 2H, ArH), 7.24 (dd, *J* = 8.1, 2.0 Hz, 1H, ArH), 2.76 (p, *J* = 7.9 Hz, 1H, CH), 1.87–1.79 (m, 2H, CH_2_), 1.74–1.61 (m, 4H, CH_2_), 1.59–1.49 (m, 2H, CH_2_). ^13^C NMR (151 MHz, DMSO-*d*_6_) *δ* 175.34, 166.58 (d, *J* = 243.8 Hz, CF), 143.17 (d, *J* = 11.9 Hz), 136.45 (d, *J* = 10.4 Hz), 114.47, 105.70 (d, *J* = 30.3 Hz), 45.88, 30.56, 26.19. ^11^B NMR (193 MHz, DMSO-*d*_6_) *δ* 26.67. IR (ATR-Ge, cm^−1^): 3298 (*ν* O–H), 1666 (*δ* C=O), 1582 (*δ* CONH), 1316 (*ν* B–O), 644 (*δ* BO_2_). HPLC purity 99.9%. MS: [M + H]^+^ = 252.0 (exact mass 251.1).

##### 4-Isobutyramido-2-(trifluoromethyl)phenylboronic acid (1c)

White solid. Yield: 55%. mp 131.7–135.3 °C. ^1^H NMR (600 MHz, DMSO-*d*_6_) *δ* 10.05 (s, 1H, CONH), 8.19 (s, 2H, BO_2_H_2_), 8.01 (d, *J* = 2.0 Hz, 1H, ArH), 7.71 (dd, *J* = 8.0, 2.0 Hz, 1H, ArH), 7.44 (d, *J* = 8.0 Hz, 1H, ArH), 2.58 (m, 1H, CH), 1.10 (d, *J* = 6.7 Hz, 6H, CH_3_). ^13^C NMR (151 MHz, DMSO-*d*_6_) *δ* 176.22, 140.09, 133.78, 131.72 (q, *J* = 30.3 Hz), 130.99, 125.24 (q, *J* = 273.2 Hz, CF_3_), 121.74, 115.70 (q, *J* = 5.5 Hz), 35.55, 19.93. ^11^B NMR (193 MHz, DMSO-*d*_6_) *δ* 26.04. IR (ATR-Ge, cm^−1^): 3280 (*ν* O–H), 1656 (*δ* C=O), 1525 (*δ* CONH), 1325 (*ν* B–O), 633 (*δ* BO_2_). HPLC purity 99.3%. MS: [M + H]^+^ = 276.0 (exact mass 275.1).

##### 4-Butyramido-2-(trifluoromethyl)phenylboronic acid (2c)

White solid. Yield: 52%. mp 118.8–123.5 °C. ^1^H NMR (600 MHz, DMSO-*d*_6_) *δ* 10.10 (s, 1H, CONH), 8.19 (s, 2H, BO_2_H_2_), 7.99 (d, *J* = 2.0 Hz, 1H, ArH), 7.69 (dd, *J* = 8.2, 2.0 Hz, 1H, ArH), 7.44 (d, *J* = 8.2 Hz, 1H, ArH), 2.29 (t, *J* = 7.3 Hz, 2H, CH_2_), 1.60 (h, *J* = 7.4 Hz, 2H, CH_2_), 0.91 (t, *J* = 7.4 Hz, 3H, CH_3_). ^13^C NMR (151 MHz, DMSO-*d*_6_) *δ* 172.16, 139.95, 133.79, 131.72 (q, *J* = 30.3 Hz), 131.00 (s, CB), 125.23 (q, *J* = 273.6 Hz, CF_3_), 121.63, 115.54 (q, *J* = 4.8 Hz), 38.84, 18.94, 14.12. ^11^B NMR (193 MHz, DMSO-*d*_6_) *δ* 26.94. IR (ATR-Ge, cm^−1^): 3312 (*ν* O–H), 1676 (*δ* C=O), 1531 (*δ* CONH), 1323 (*ν* B–O), 658 (*δ* BO_2_). HPLC purity 99.5%. MS: [M + H]^+^ = 275.9 (exact mass 275.1).

##### 4-Hexanamido-2-(trifluoromethyl)phenylboronic acid (3c)

White solid. Yield: 99%. mp 140.1–147.7 °C. ^1^H NMR (600 MHz, DMSO-*d*_6_) *δ* 10.09 (s, 1H, CONH), 8.19 (s, 2H, BO_2_H_2_), 7.99 (s, 1H, ArH), 7.69 (d, *J* = 8.1 Hz, 1H, ArH), 7.44 (d, *J* = 8.1 Hz, 1H, ArH), 2.30 (t, *J* = 7.5 Hz, 2H, CH_2_), 1.63–1.55 (m, 2H, CH_2_), 1.35–1.23 (m, 4H, CH_2_), 0.87 (t, *J* = 6.8 Hz, 3H, CH_3_). ^13^C NMR (151 MHz, DMSO-*d*_6_) *δ* 172.31, 139.98, 133.80, 131.72 (q, *J* = 30.3 Hz), 125.23 (q, *J* = 273.3 Hz, CF_3_), 121.62, 115.53, 36.91, 31.40, 25.18, 22.43, 14.38. ^11^B NMR (193 MHz, DMSO-*d*_6_) *δ* 25.28. IR (ATR-Ge, cm^−1^): 3320 (*ν* O–H), 1673 (*δ* C=O), 1531 (*δ* CONH), 1324 (*ν* B–O), 652 (*δ* BO_2_). HPLC purity 99.7%. MS: [M + H]^+^ = 304.0 (exact mass 303.1).

##### 4-(Cyclobutanecarboxamido)-2-(trifluoromethyl)phenylboronic acid (4c)

White solid. Yield: 40%. mp 141.2–149.1 °C. ^1^H NMR (600 MHz, DMSO-*d*_6_) *δ* 9.95 (s, 1H, CONH), 8.19 (s, 2H, BO_2_H_2_), 8.01 (d, *J* = 2.0 Hz, 1H, ArH), 7.71 (dd, *J* = 8.2, 2.0 Hz, 1H, ArH), 7.43 (d, *J* = 8.2 Hz, 1H, ArH), 3.22 (pd, *J* = 8.5, 1.0 Hz, 1H, CH), 2.26–2.17 (m, 2H, CH_2_), 2.15–2.06 (m, 2H, CH_2_), 1.99–1.88 (m, 1H, CH_2_), 1.85–1.76 (m, 1H, CH_2_). ^13^C NMR (151 MHz, DMSO-*d*_6_) *δ* 173.91, 140.01, 133.78, 131.71 (q, *J* = 30.3 Hz), 130.97 (s, CB), 125.23 (q, *J* = 273.5 Hz, CF_3_), 121.72, 115.67 (q, *J* = 5.1 Hz), 40.13, 25.10, 18.23. ^11^B NMR (193 MHz, DMSO-*d*_6_) *δ* 25.87. IR (ATR-Ge, cm^−1^): 3271 (*ν* O–H), 1659 (*δ* C=O), 1516 (*δ* CONH), 1319 (*ν* B–O), 651 (*δ* BO_2_). HPLC purity 99.6%. MS: [M + H]^+^ = 287.9 (exact mass 287.1).

##### 4-(Cyclopentanecarboxamido)-2-(trifluoromethyl)phenylboronic acid (5c)

White solid. Yield: 74%. mp 136.0–140.0 °C. ^1^H NMR (600 MHz, DMSO-*d*_6_) *δ* 10.09 (s, 1H, CONH), 8.19 (s, 2H, BO_2_H_2_), 8.01 (d, *J* = 2.0 Hz, 1H, ArH), 7.70 (dd, *J* = 8.0, 2.0 Hz, 1H, ArH), 7.43 (d, *J* = 8.0 Hz, 1H, ArH), 2.77 (p, *J* = 7.9 Hz, 1H, CH), 1.88–1.79 (m, 2H, CH_2_), 1.76–1.61 (m, 4H, CH_2_), 1.59–1.50 (m, 2H, CH_2_). ^13^C NMR (151 MHz, DMSO-*d*_6_) *δ* 175.41, 140.11, 133.78, 131.72 (q, *J* = 30.3 Hz), 130.93 (s, CB), 125.24 (q, *J* = 273.2 Hz, CF_3_), 121.68, 115.64 (q, *J* = 5.0 Hz), 45.83, 30.56, 26.22. ^11^B NMR (193 MHz, DMSO-*d*_6_) *δ* 25.06. IR (ATR-Ge, cm^−1^): 3274 (*ν* O–H), 1655 (*δ* C=O), 1517 (*δ* CONH), 1315 (*ν* B–O), 652 (*δ* BO_2_). HPLC purity 99.8%. MS: [M + H]^+^ = 302.2 (exact mass 301.1).

### Cell viability assays

#### LAPC-4 cancer cell line

The androgen-dependent human prostate cell line LAPC-4 was kindly provided by assoc. prof. Hatina (Charles University, Faculty of Medicine in Pilsen) with the consent of Dr. Reiter (University of California) and maintained in Iscove's modified Dulbecco's medium (IMDM) medium (Sigma-Aldrich®, USA). LAPC-4 cell line was supplemented with 10% of fetal bovine serum (Sigma-Aldrich®, USA), 100 IU mL^−1^ of penicillin, and 100 μg mL^−1^ of streptomycin (Sigma-Aldrich®, USA) at 37 °C in a 5% CO_2_ incubator (Sigma-Aldrich®, USA).

For LAPC-4 all the analogs were dissolved in dimethylsulfoxide (DMSO) as stock solutions, which were further diluted in IMDM into six different concentrations from 1 μM to 100 μM. LAPC-4 cells were seeded into a flat bottom 96-well plate at the density of 1 × 10^4^ per well and treated with different concentrations of the tested compounds. The final concentration of DMSO was <0.1% (v/v) in all experiments. The cells were incubated for 72 hours at 37 °C in 5% CO_2_ atmosphere. After 72 hours, 10 μl of Cell Proliferation Reagent WST-1 (Roche Diagnostics GmbH, Germany) were added to each well and incubated for another 4 hours at 37 °C and 5% CO_2_ atmosphere. Flutamide (Sigma-Aldrich®, USA) and bicalutamide (Sigma-Aldrich®, USA) were used as positive controls throughout the experiment. Each experiment was performed in three technical replicates and repeated at least three times.

The cell viability was determined using the WST-1 (4-[3-(4-iodophenyl)-2-(4-nitrophenyl)-2*H*-5-tetrazolium]-1,3-benzene disulfonate) assay. The analysis was based on the reduction of tetrazolium salts into formazan; the rate of WST-1 cleavage by mitochondrial dehydrogenases correlates with the number of viable cells. The absorbance of each well was measured by using a microplate reader (Synergy H1, BioTek, USA) at 450 nm. Presented IC_50_ values for all compounds are the mean from all independent experiments.

The graph was plotted between % cell inhibition (or normalized value) *versus* concentration of different analogs by using non-linear regression analysis and IC_50_ values of the analogs were calculated. Statistical analyses were performed using GraphPad Prism 7.02 (GraphPad Software, Boston, MA USA).

#### HepG2, PC-3, HK-2 cell lines

All used cell cultures are commercially available from American Type Culture Collection (ATCC) and were purchased less than 10 years ago. Cell lines were tested on mycoplasma contamination upon their delivery. Human hepatocellular carcinoma cells HepG2 (ATCC HB-8065) and human proximal tubule kidney cells HK-2 (ATCC CRL-2190) were cultured in Dulbecco's modified Eagles medium high glucose (denoted DMEM high glucose) supplemented with fetal bovine serum (10%), non-essential amino acids (10%), and penicillin/streptomycin (10%). Human prostate adenocarcinoma cells PC-3 (ATCC CRL-1435) were cultured in Kaighn's modification of Ham's F-12 medium (denoted F-12K medium) supplemented with fetal bovine serum (10%) and penicillin/streptomycin (10%). All media and supplements were purchased from Merck (USA) unless otherwise stated. All cell lines were cultured at 37 °C, 5% CO_2_ in a humidified atmosphere and grown to confluence. Before the experimental day, cells were seeded into a 96-well plate with the initial cell number of 1.0 × 10^4^ in 100 μL of cell media per well. After 24 h incubation at 37 °C, cells were treated with the tested compounds in the final volume of 100 μL containing 1% DMSO (v/v). Cells were incubated with the compounds at the following concentrations 1, 5, 10, 25, 50, 100, 250, 500, and 1000 μM for 24 h (all cell lines) and 72 h (HK-2 and PC-3 cells only). Controls were treated with cell culture medium only and with 1% DMSO (v/v) dissolved in appropriate cell culture medium at 37 °C for either 24 h or 72 h. The absolute mortality of cells was also analyzed with 10% DMSO (v/v) dissolved in appropriate cell culture medium at 37 °C for 24 h or 72 h. Controls and compound concentrations were made in triplicate of wells.

When either 24 h or 72 h incubation was over, the reagent from the kit CellTiter 96 AQueous One Solution Cell Proliferation Assay (CellTiter 96; PROMEGA, Fitchburg, USA) was added. After 2 h incubation at 37 °C, absorbance in each sample well was recorded at 490 nm (TECAN, Infinite M200, Austria).

The 50% inhibitory concentrations (IC_50_) values were determined from the dose–response curves in GraphPad Prism 10 (GraphPad Software, Boston, MA USA). Viability (%) was plotted as a function of concentration (log values), fitted to a sigmoidal curve, and based on this curve, the half maximal inhibitory concentration value was determined representing the concentration of a compound required for 50% inhibition.

## Abbreviations

AR: Androgen receptor
Arg-OMe: l-Arginine methyl ester
HF: Hydroxyflutamide
HFB: Hydroxyflutamide bioisostere
LBD: Ligand binding domain
NSAAs: Non-steroidal antiandrogens
SI: Selectivity index
WT: Wild-type

## Note

The compounds do not belong to the group of PAINS which was virtually screened in the two assays at https://zinc15.docking.org/patterns/home and https://advisor.docking.org/.

## Data availability

The data underlying this study are available in the published article and its ESI† files.

## Author contributions

Petr Šlechta: conceptualization, methodology, software, investigation, data curation, writing – original draft, visualization. Roman Viták: methodology, validation, formal analysis, investigation, data curation, writing – review & editing. Pavel Bárta: methodology, validation, formal analysis, investigation, data curation, writing – review & editing. Kateřina Koucká: investigation. Monika Berková: software, investigation. Diana Žďárová: investigation. Andrea Petríková: investigation. Jiří Kuneš: methodology, validation, investigation, data curation. Vladimír Kubíček: methodology, validation, formal analysis, investigation, data curation. Martin Doležal: resources, supervision, funding acquisition. Radek Kučera: resources, supervision, funding acquisition. Marta Kučerová-Chlupáčová: conceptualization, writing – original draft, visualization, project administration.

## Conflicts of interest

The authors declare no competing financial interest nor any other conflict of interest.

## Supplementary Material

MD-OLF-D4MD00343H-s001

MD-OLF-D4MD00343H-s002
