# Supplementary material for: Replacement of nitro function by free boronic acid in non-steroidal anti-androgens
Source: RSC Med Chem. 2024 Sep 10;15(12):4018–38. doi: 10.1039/d4md00343h (PMC11428147; doi:10.1039/d4md00343h)

Supplementary Information (SI) for RSC Medicinal Chemistry.
This journal is © The Royal Society of Chemistry 2024

Replacement of nitro function by free boronic acid in the group of non-steroidal anti-androgens.

Petr Šlechta[1], Roman Viták[2], Pavel Bárta[3], Kateřina Koucká[1], Monika Berková[1], Diana Žďárová[1], Andrea Petríková[1], Jiří Kuneš[4], Vladimír Kubíček[3], Martin Doležal[1], Radek Kučera[2], and Marta Kučerová-Chlupáčová[1*]

[1] Department of Pharmaceutical Chemistry and Pharmaceutical Analysis, Faculty of Pharmacy in Hradec Králové, Charles University, Ak. Heyrovského 1203/8, 50003 Hradec Králové, Czech Republic

[2] Department of Pharmacology and Toxicology, Faculty of Medicine in Pilsen, Charles University, alej Svobody 1655/76, 32300 Plzeň, Czech Republic

[3] Department of Biophysics and Physical Chemistry, Faculty of Pharmacy in Hradec Králové, Charles University, Ak. Heyrovského 1203/8, 50003 Hradec Králové, Czech Republic

[4] Department of Organic and Bioorganic Chemistry, Faculty of Pharmacy in Hradec Králové, Charles University, Ak. Heyrovského 1203/8, 50003 Hradec Králové, Czech Republic

*Email: kucerom@faf.cuni.cz

Supporting information

Table of contents

Results of *in vitro* cytotoxicity screening **S1**

Table S1 .... S1

Table S2 .... S4

Table S3 .... S5

Table S4 .... S5

NMR spectra of final compounds **S6**

Results of *in vitro* cytotoxicity screening

Table S1: Complete results of in vitro cytotoxicity screening for aromatic amides against the tested cell lines with indicated incubation time and calculated selectivity index (SI).

**Structure**	**Code**	**IC_{50} (µM)**					
		HepG2 (24 h)	PC3 (24 h)	PC3 (72 h)	HK-2 (72 h)	LAPC4 (72 h)	SI (HK-2/LAPC4)
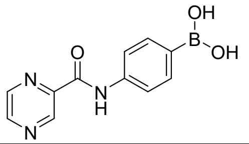	**1a**	>250	>100	na	na	>100	na
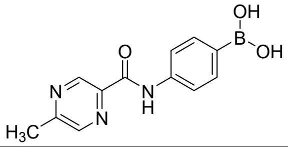	**2a**	>100	>100	na	na	>100	na
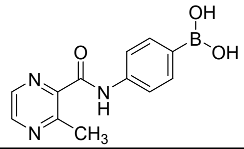	**3a**	>500	>1000	na	na	>100	na

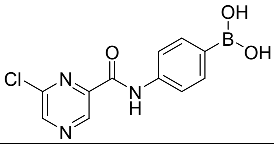	**4a**	>500	>250	na	na	>100	na
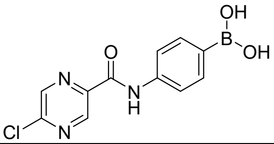	**5a**	139.3	133.4	121.6	194.5	27.8	7.0
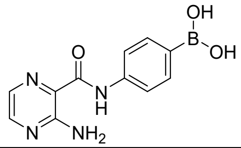	**6a**	>250	>250	na	na	>100	na
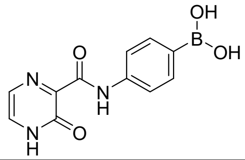	**7a**	>500	>500	na	na	>100	na
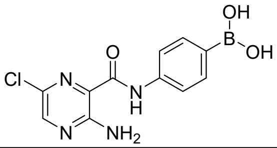	**8a**	126.4	193.3	375.2	422.4	78.3	5.4
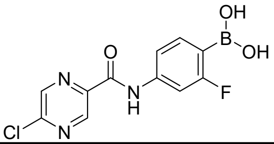	**9a**	71.26	118.4	78.1	137.7	19.2	7.2
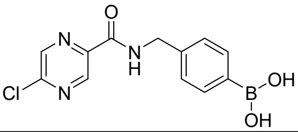	**10a**	187	>1000	227.9	183.7	48.0	3.8
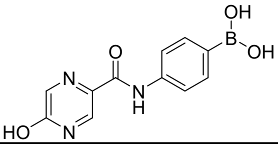	**11a**	>1000	296	779.6	1000	>100	na
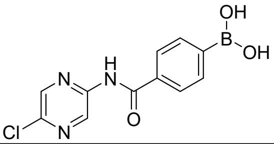	**12a**	>1000	na	na	na	>100	na
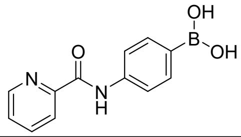	**13a**	>250	>250	na	na	>100	na
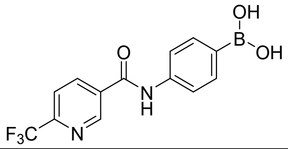	**14a**	>100	>100	na	na	>100	na
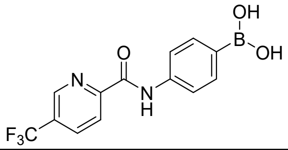	**15a**	>25	>50	na	na	>100	na

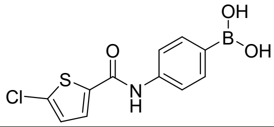	**16a**	>50	>50	na	na	>100	na
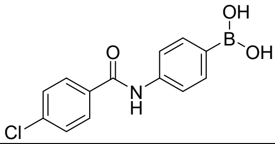	**17a**	>25	>100	na	na	>100	na
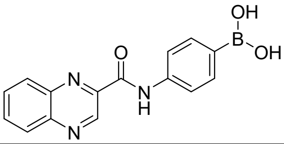	**18a**	>50	>50	na	na	>100	na
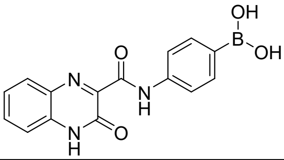	**19a**	>50	>50	na	na	>100	na
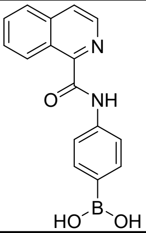	**20a**	322.2	>250	na	na	>100	na

NOTE: na= not available.

Table S2: Complete results of in vitro cytotoxicity screening for aliphatic and alicyclic amides against the tested cell lines with indicated incubation time and calculated selectivity index (SI).

**Structure**	**Code**	**IC_{50} (µM)**					
		HepG2 (24 h)	PC3 (24 h)	PC3 (72 h)	HK-2 (72 h)	LAPC4 (72 h)	SI (HK-2/LAPC4)
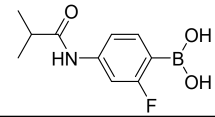	**1b**	>1000	>1000	na	na	>100	na
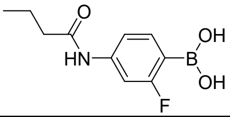	**2b**	>1000	>1000	na	na	>100	na
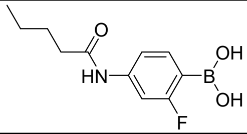	**3b**	>1000	877.7	na	na	>100	na
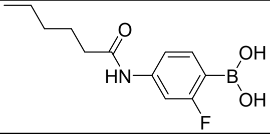	**4b**	>250	>250	na	na	>100	na
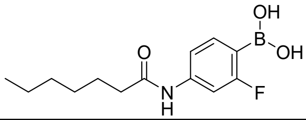	**5b**	>250	>250	na	na	>100	na
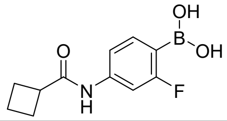	**6b**	>1000	>1000	na	na	>100	na
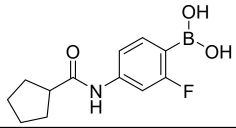	**7b**	>500	>1000	na	na	>100	na

NOTE: na= not available.

Table S3: Complete results of in vitro cytotoxicity screening for aliphatic and alicyclic amides substituted with trifluoromethyl group against the tested cell lines with indicated incubation time and calculated selectivity index (SI).

Structure	Code	IC_{50} (µM)					
		HepG2 (24h)	PC3 (24h)	PC3 (72h)	HK-2 (72h)	LAPC4 (72h)	SI (HK-2/LAPC4)
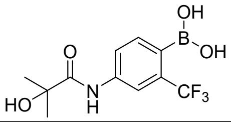	**HFB**	>1000	>1000	>1000	>1000	403.8	na
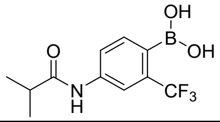	**1c**	>1000	>1000	697.1	>1000	>100	na
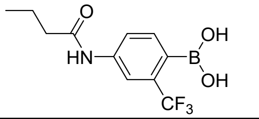	**2c**	>1000	>1000	na	na	>100	na
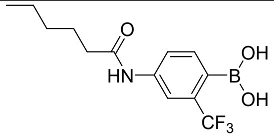	**3c**	>1000	>500	na	na	>100	na
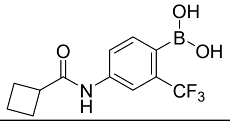	**4c**	>1000	>1000	na	na	>100	na
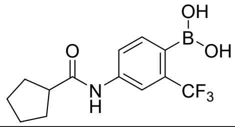	**5c**	>1000	>1000	185.6	>1000	>100	na

NOTE: na= not available.

Table S4: Results of in vitro cytotoxicity screening for standards against the tested cell lines with indicated incubation time and calculated selectivity index (SI).

Structure	Code	IC_{50} (µM)					
		HepG2 (24 h)	PC3 (24 h)	PC3 (72 h)	HK-2 (72 h)	LAPC4 (72 h)	SI (HK-2/LAPC4)
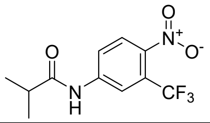	**flutamide**	137.2	34.7	50.8	76.2	60.5	1.3
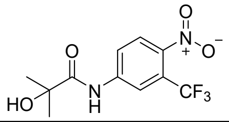	**HF**	103.2	229.2	41.8	231.0	136.1	1.7
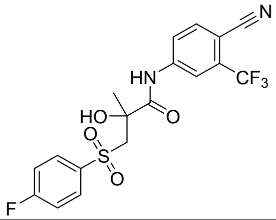	**bicalutamide (racemic mixture)**	na	na	na	na	37.2	na

NOTE: na= not available.

NMR spectra of final compounds

HFB ^{1}H NMR spectrum

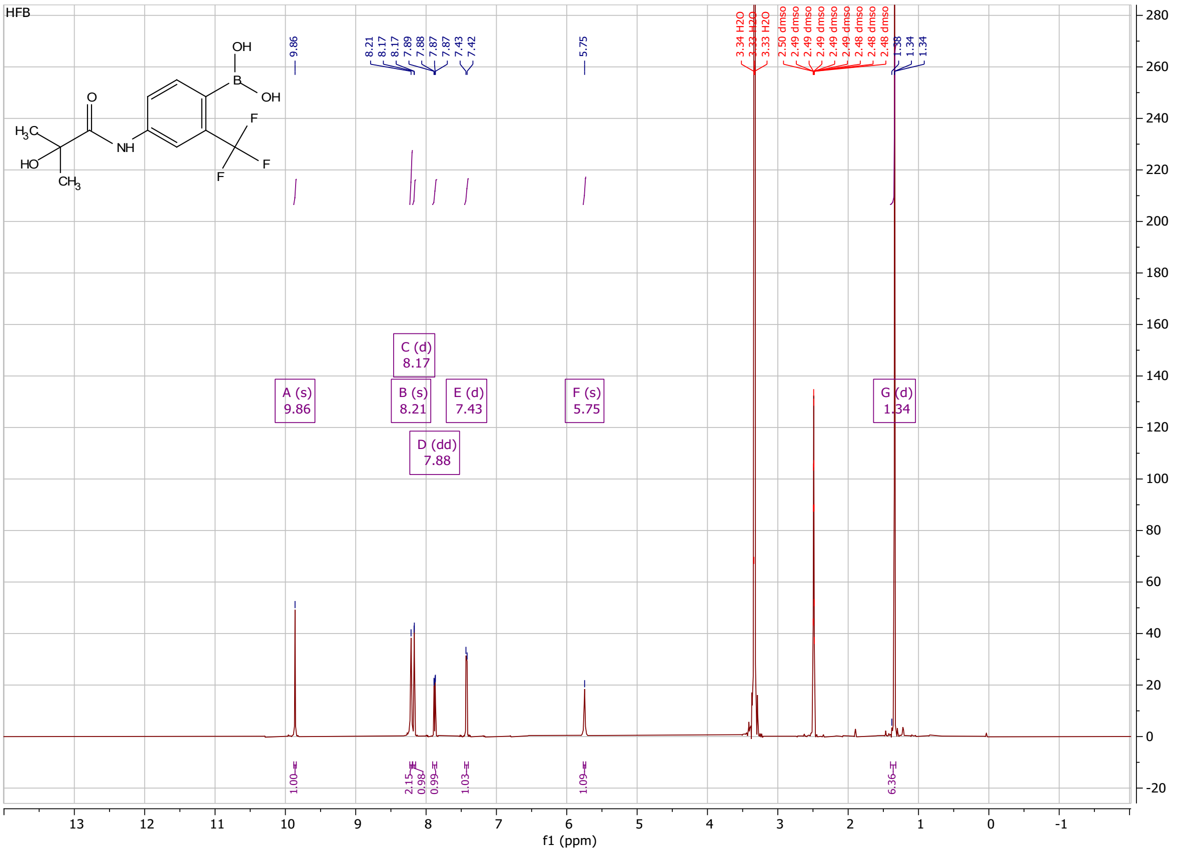

HFB ^{13}C NMR spectrum

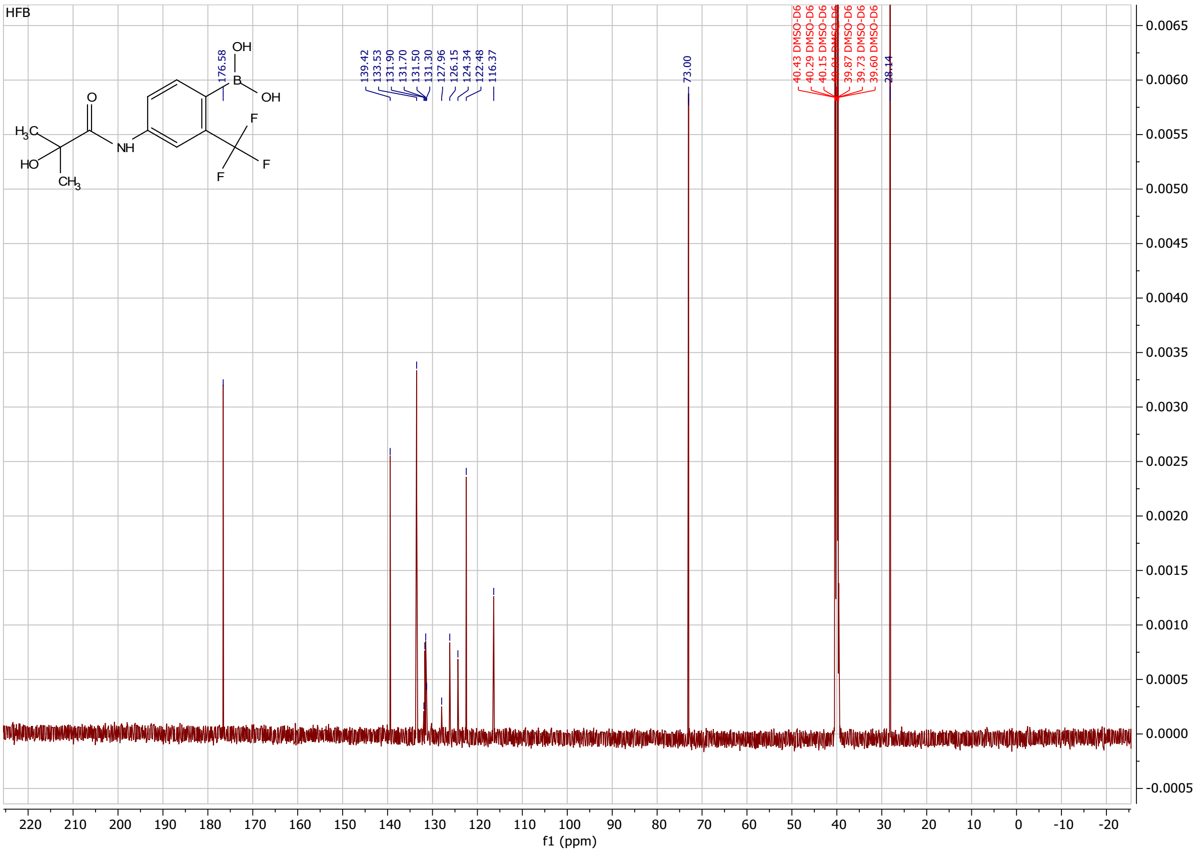

HFB ^{11}B NMR spectrum

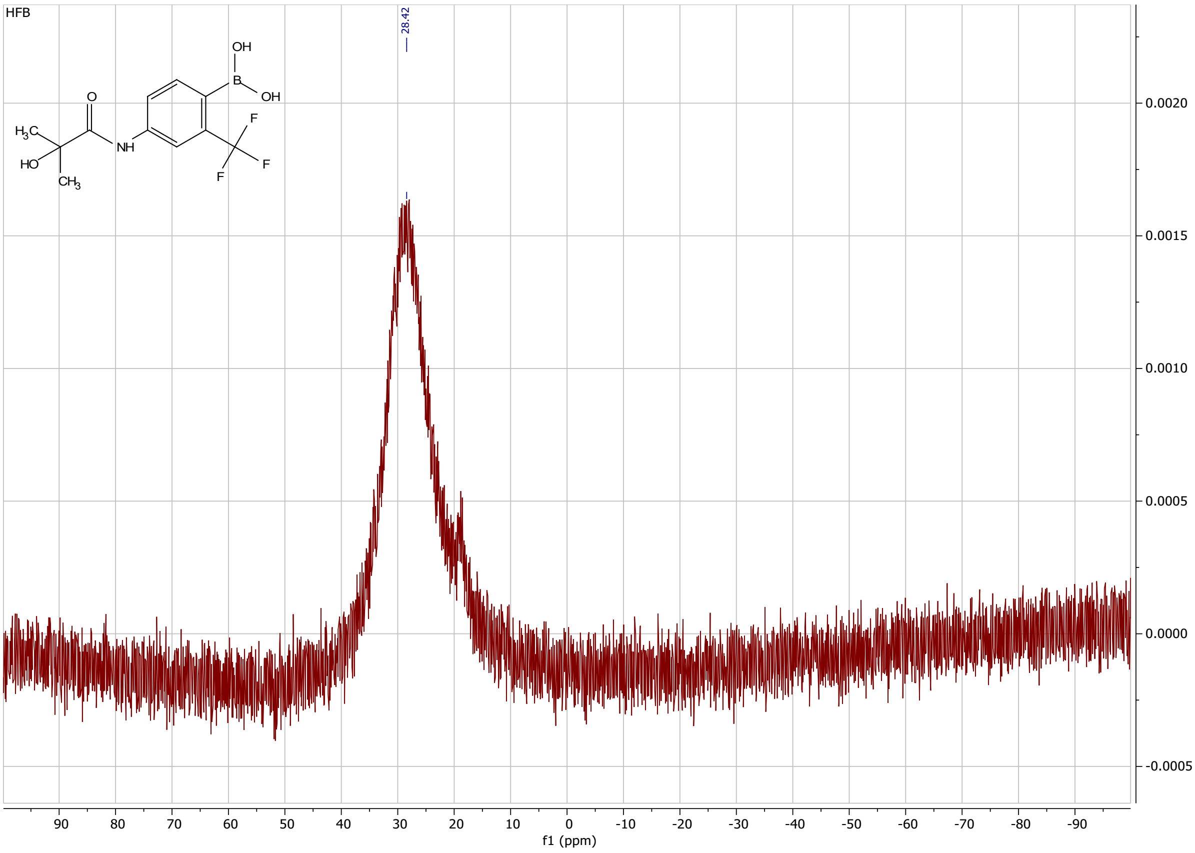

1a ^{1}H NMR spectrum

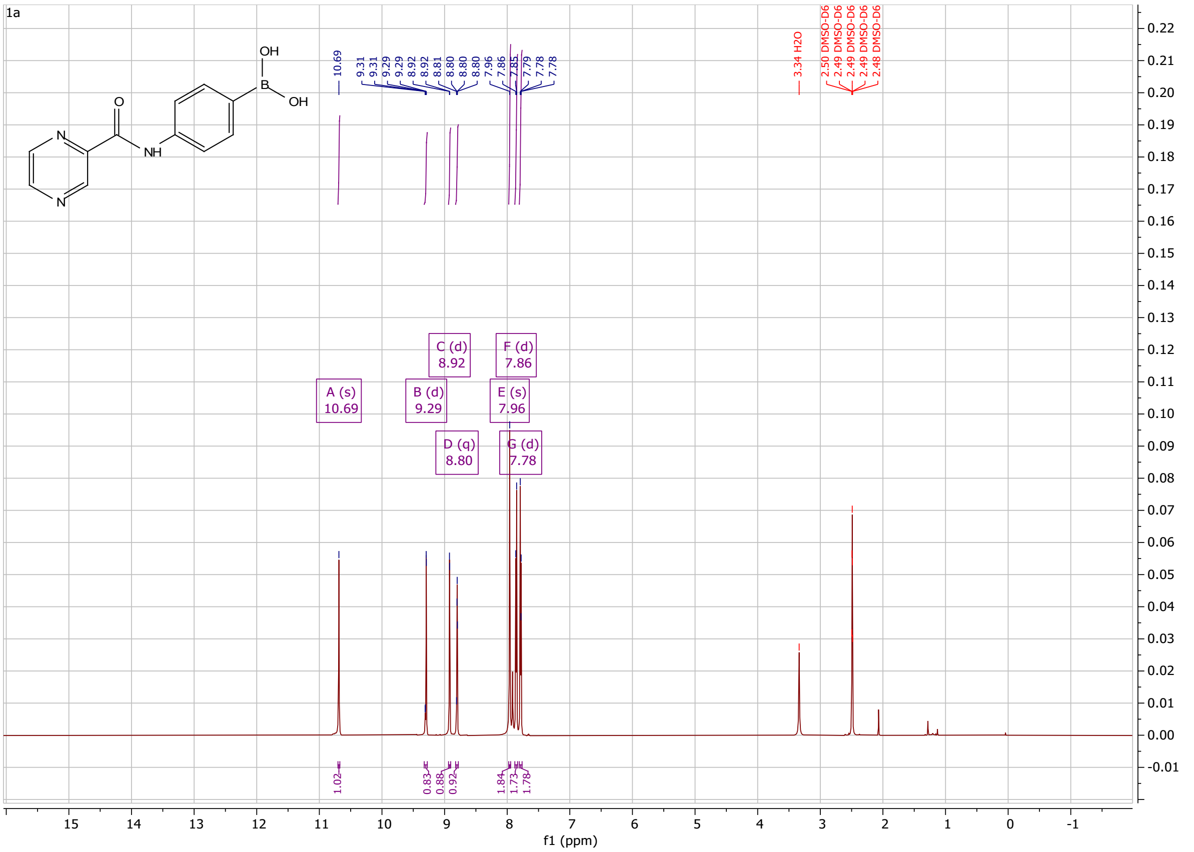

1a ^{13}C NMR spectrum

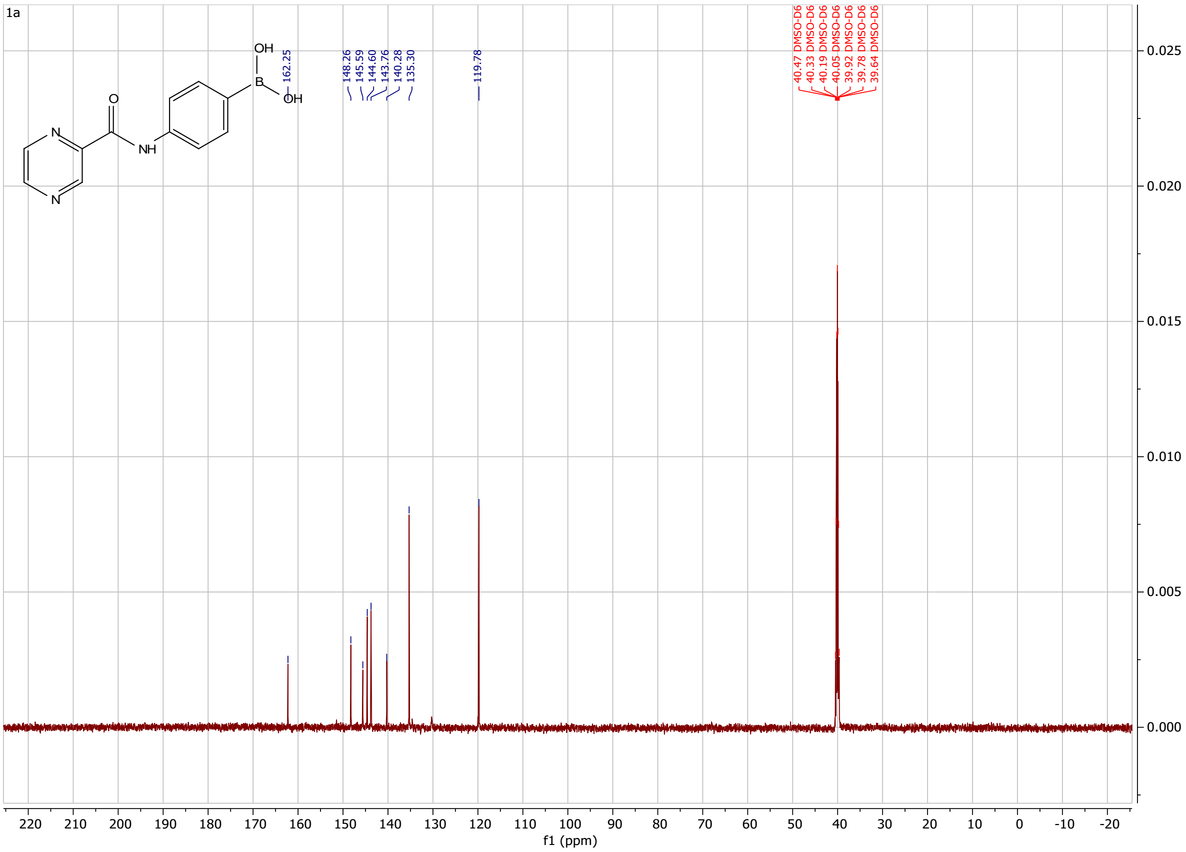

1a ^{11}B NMR spectrum

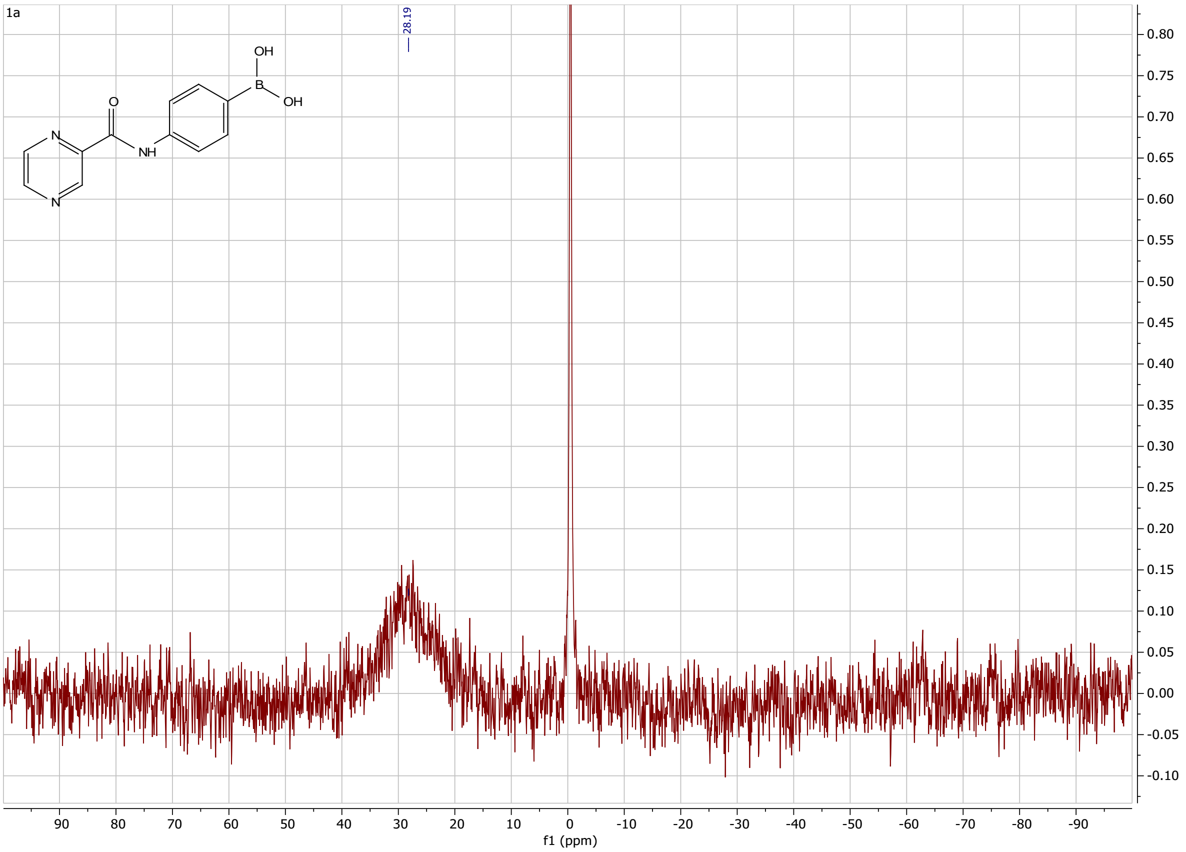

2a ^{1}H NMR spectrum

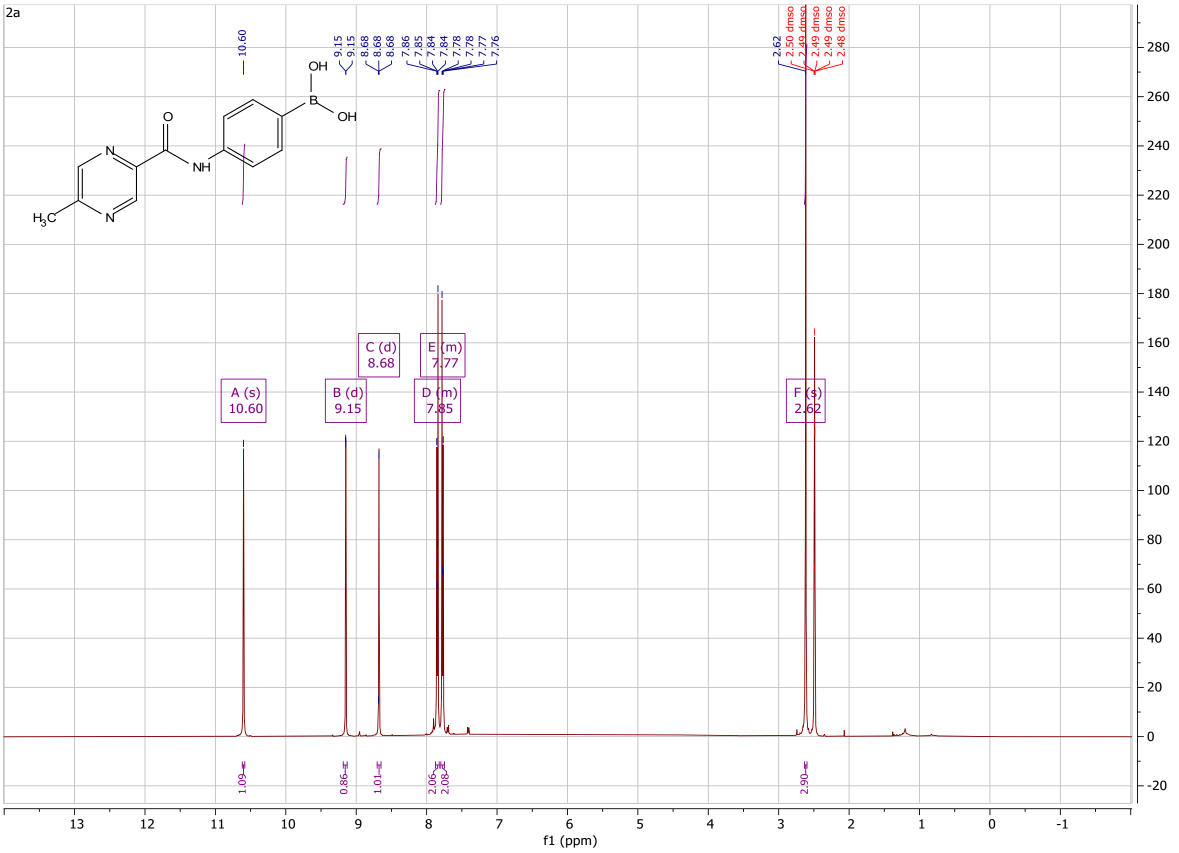

2a ^{13}C NMR spectrum

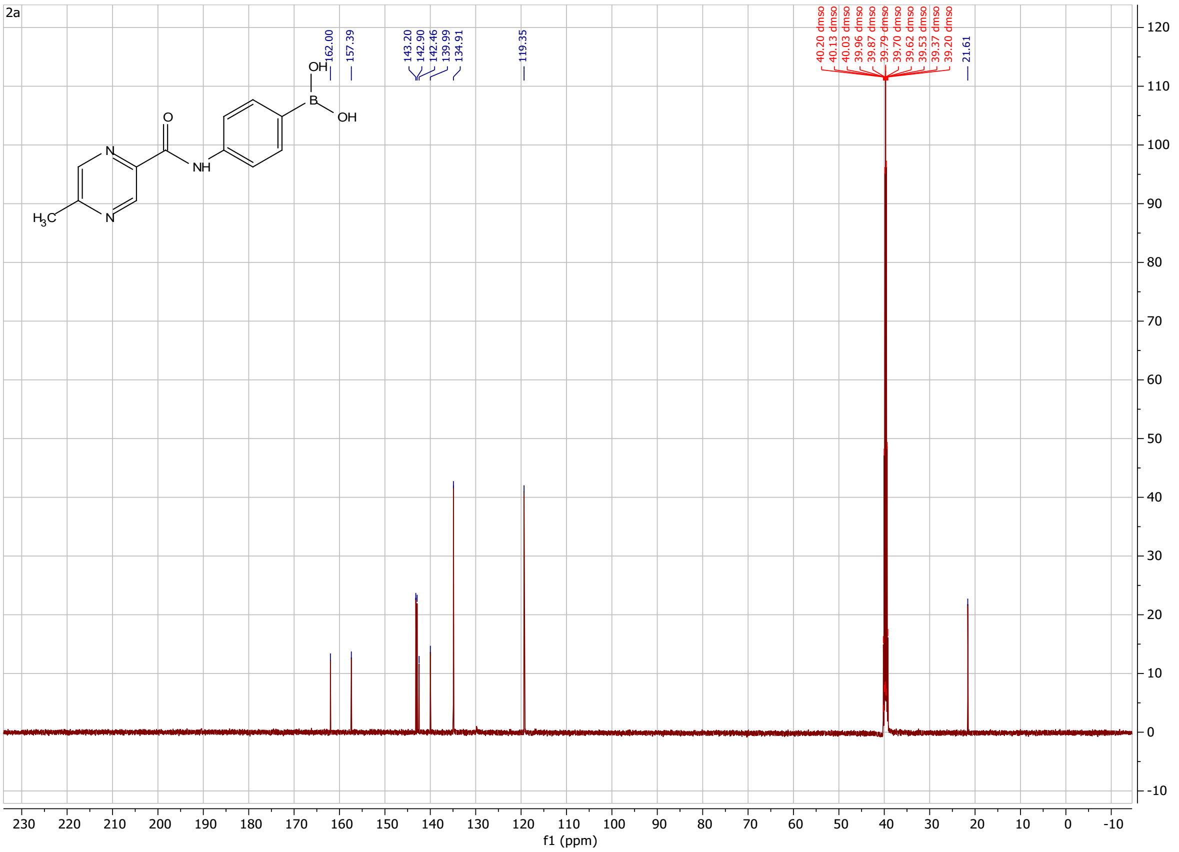

2a ^{11}B NMR spectrum

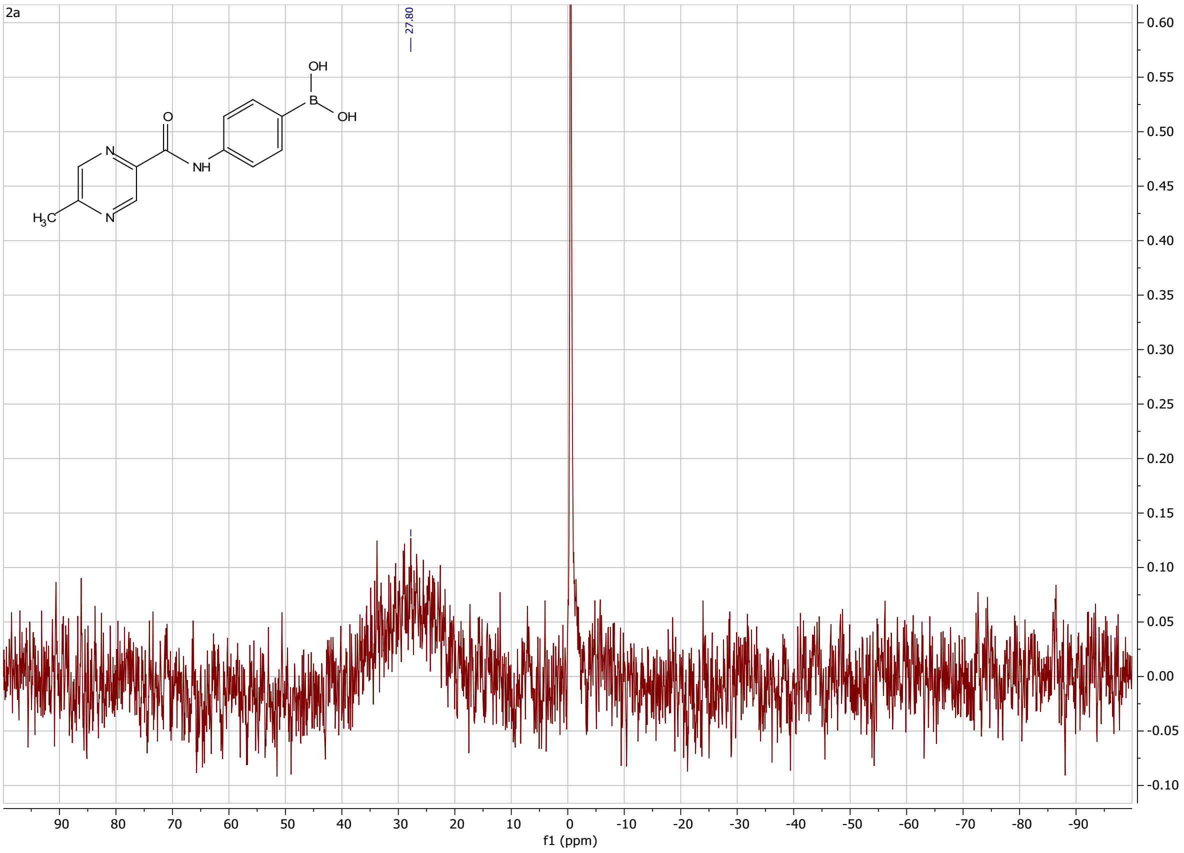

3a ^{1}H NMR spectrum

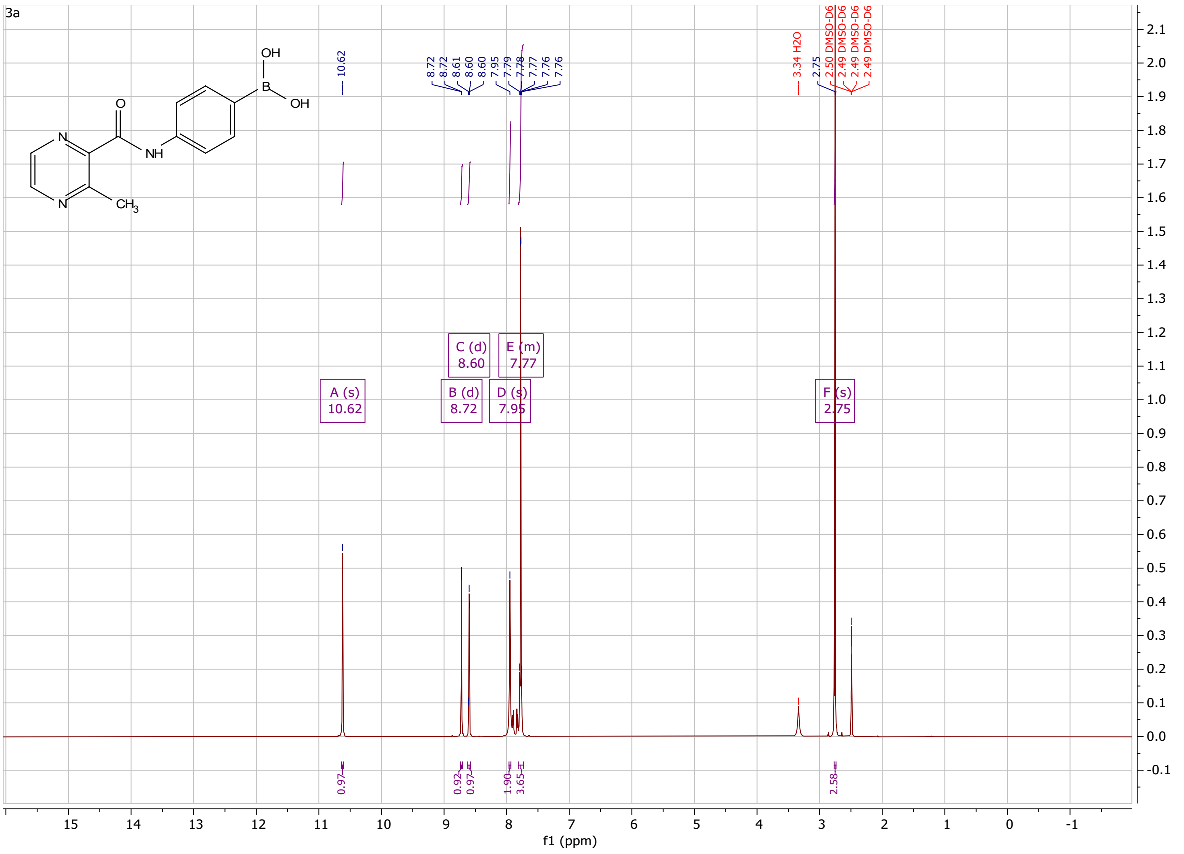

3a ^{13}C NMR spectrum

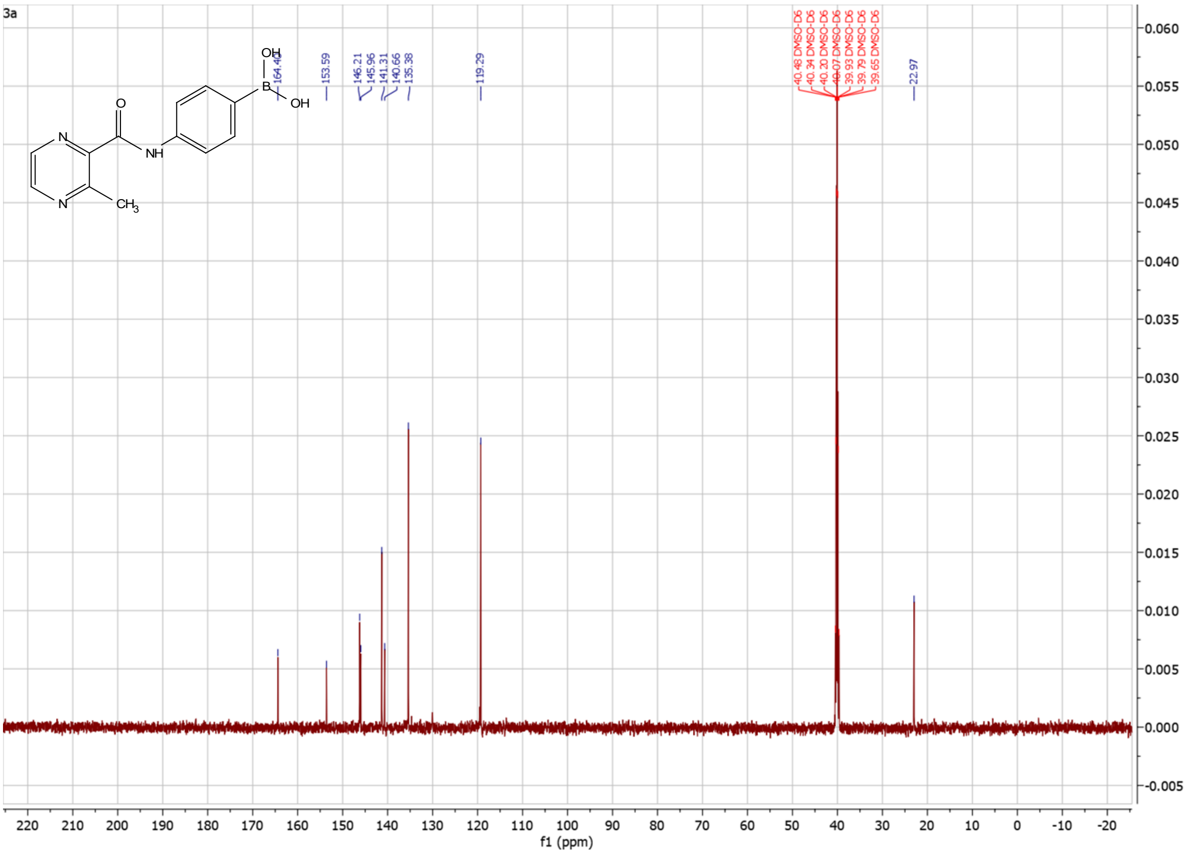

3a ^{11}B NMR spectrum

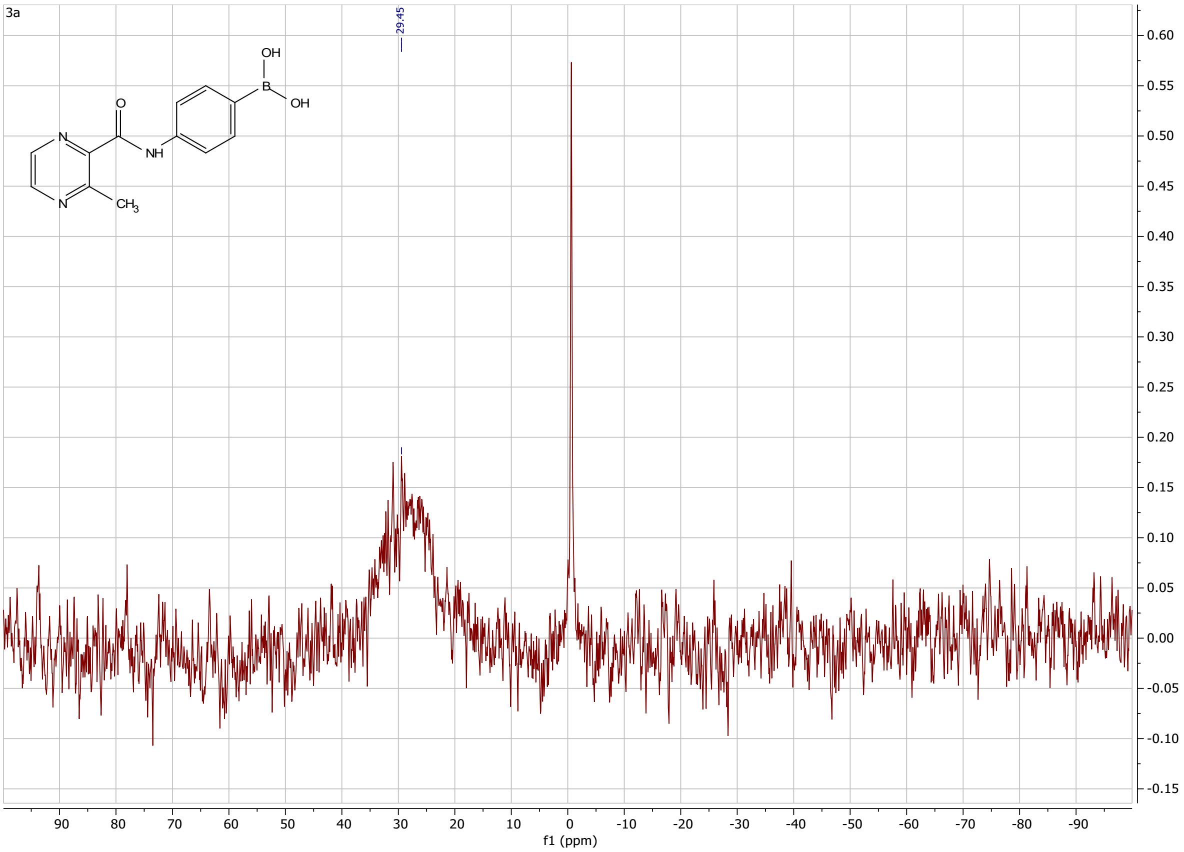

4a ^{1}H NMR spectrum

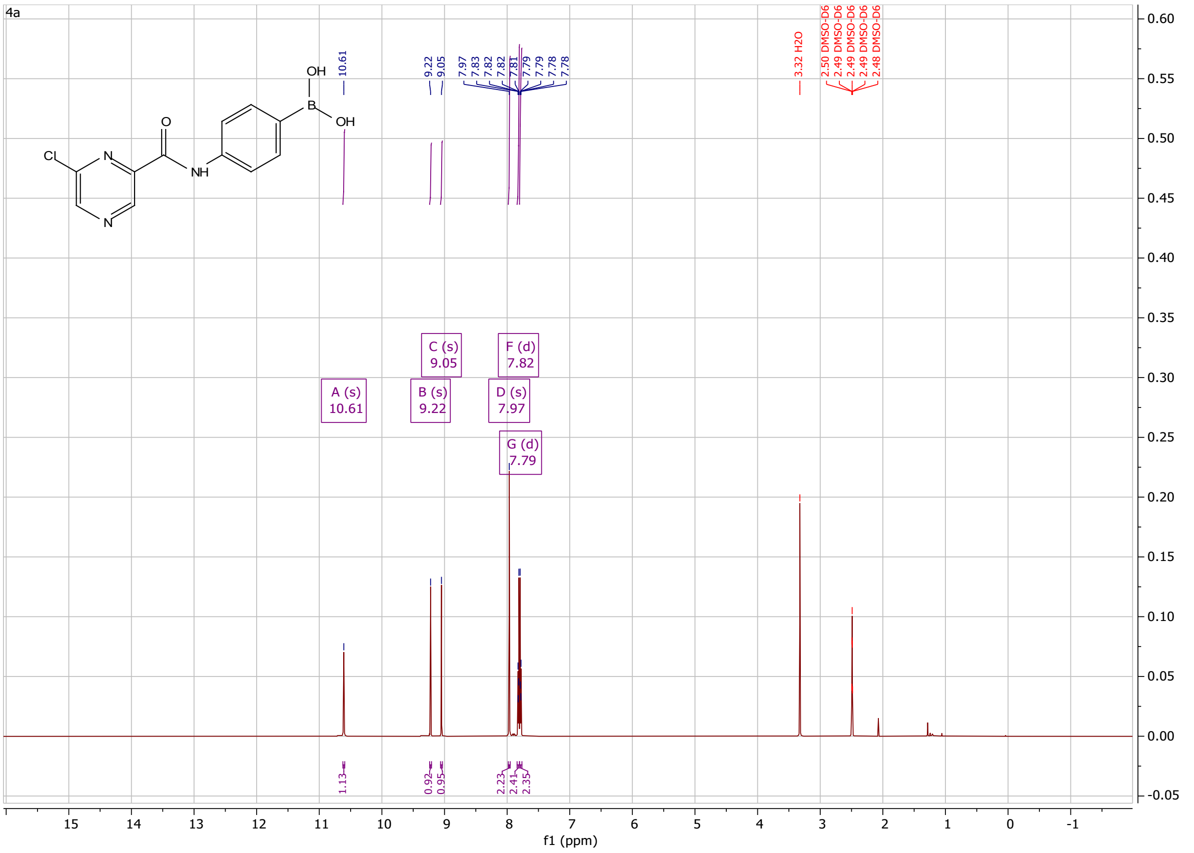

4a ^{13}C NMR spectrum

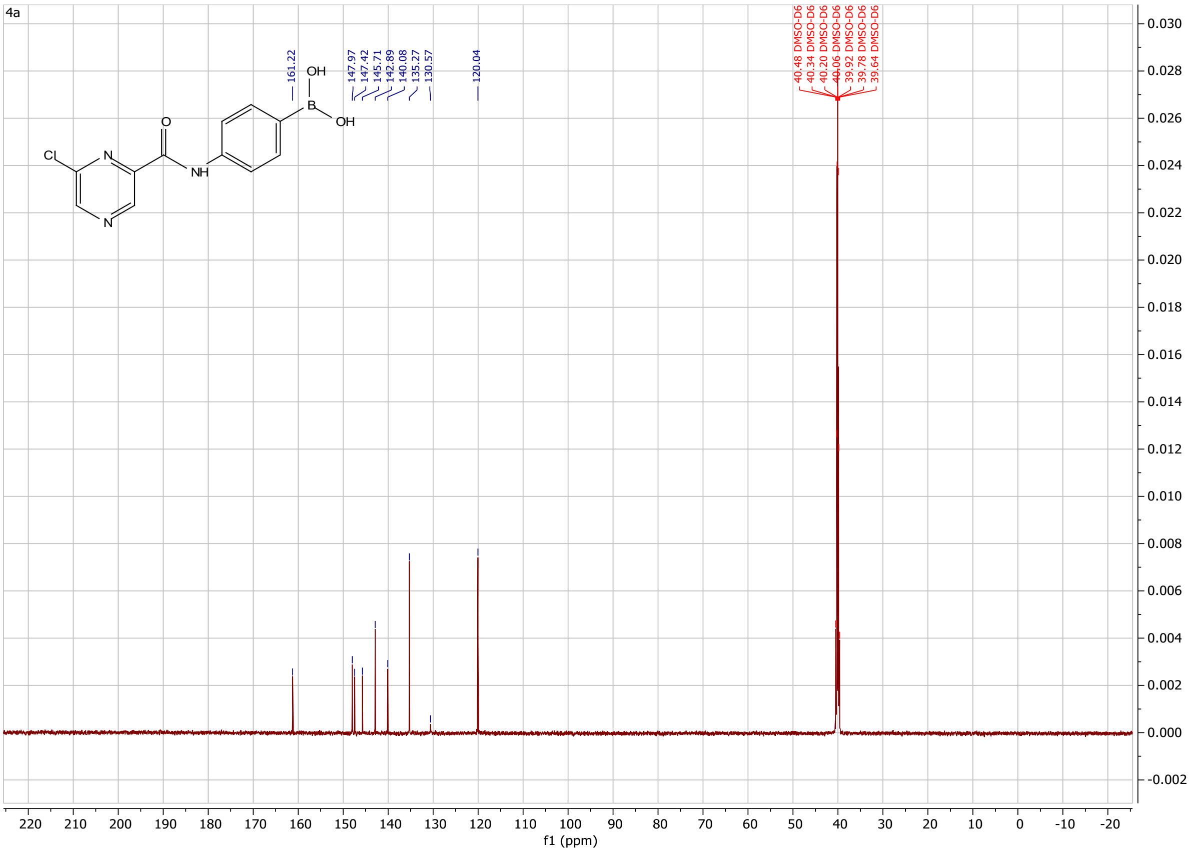

4a ^{11}B NMR spectrum

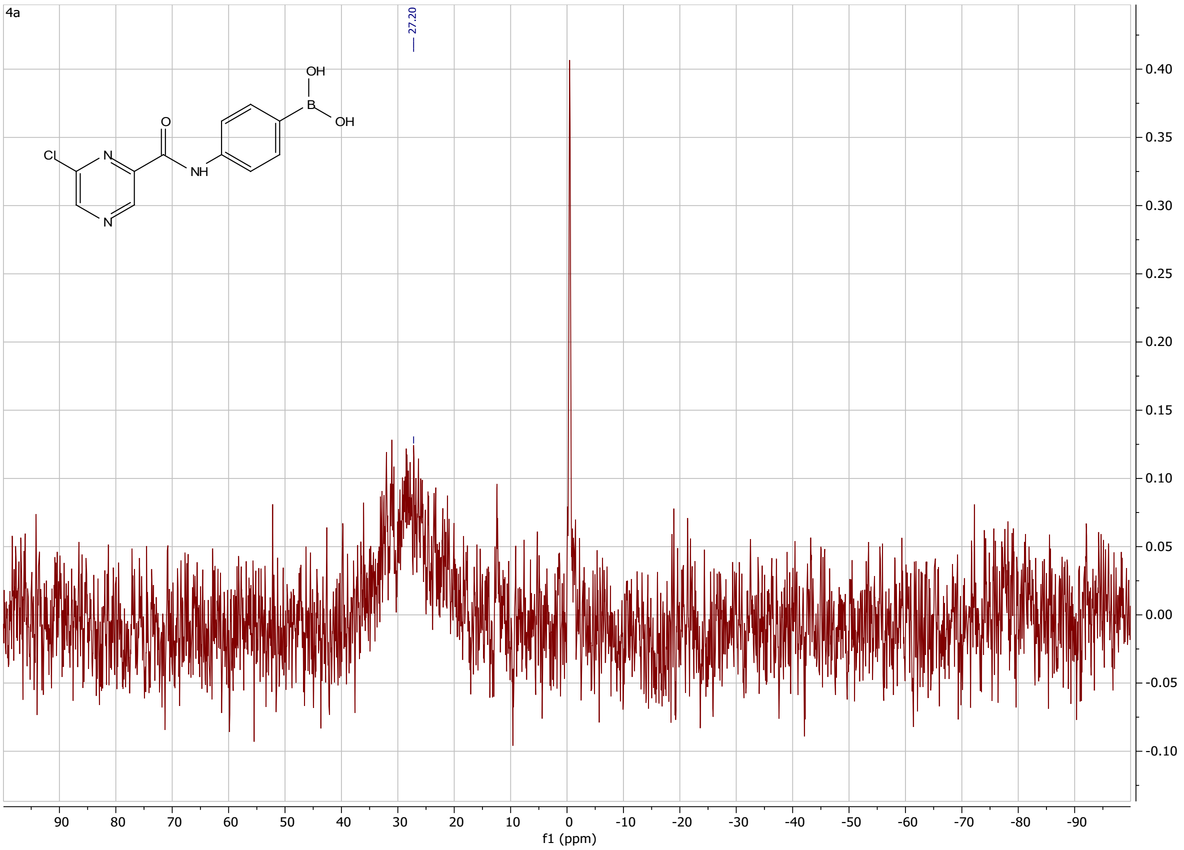

5a ^{1}H NMR spectrum

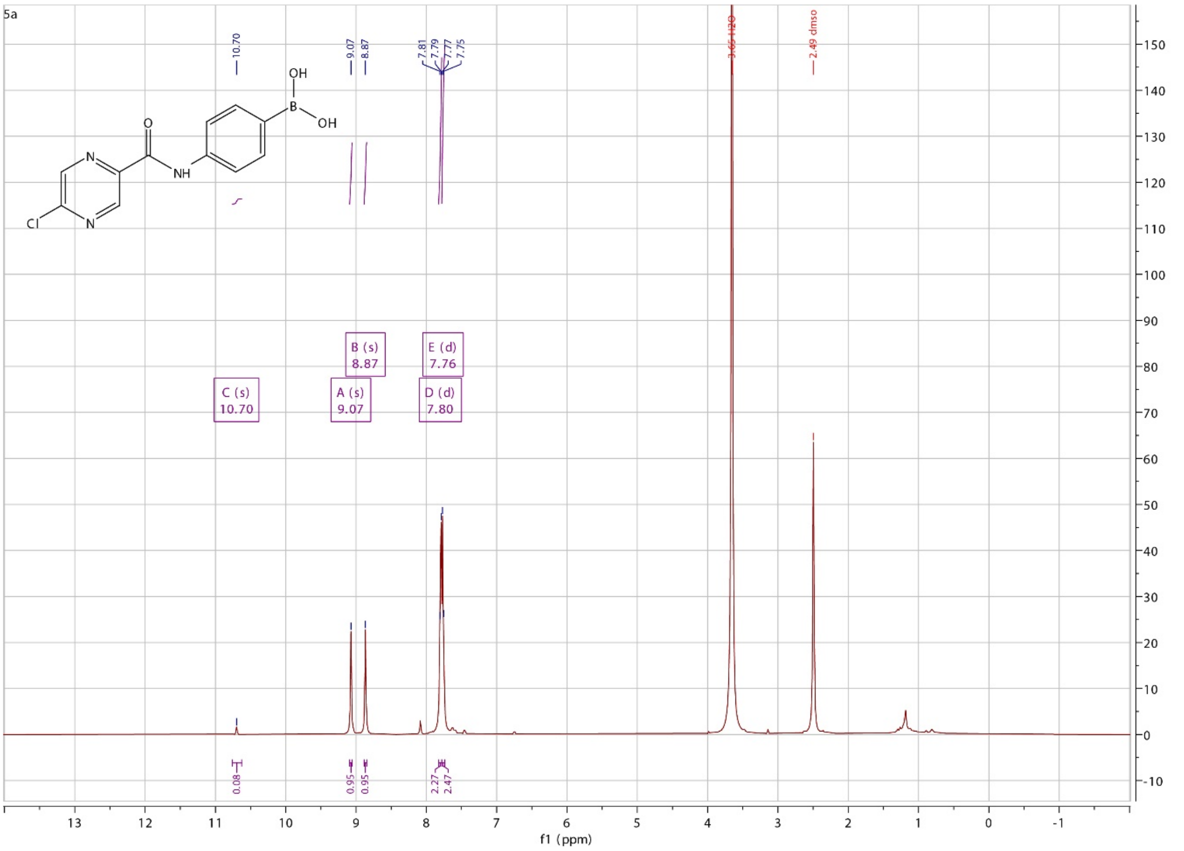

5a ^{13}C NMR spectrum

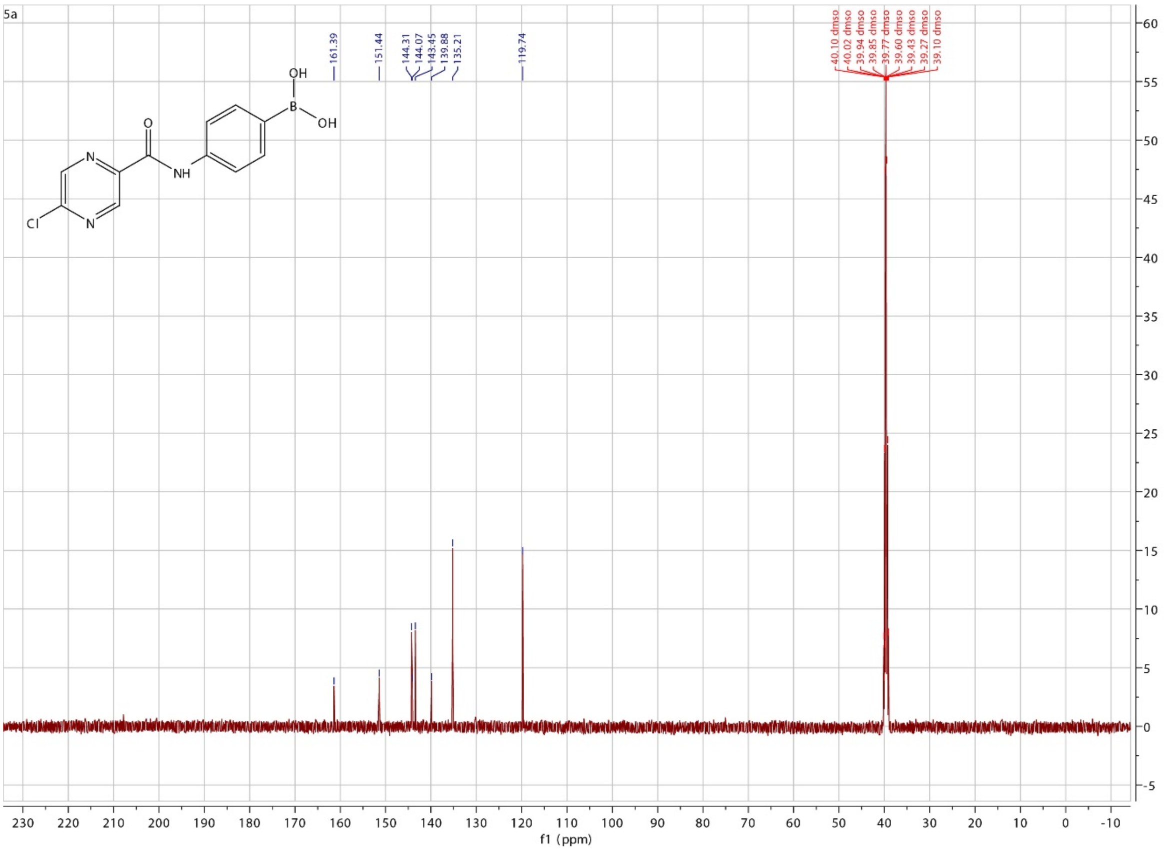

5a ^{11}B NMR spectrum

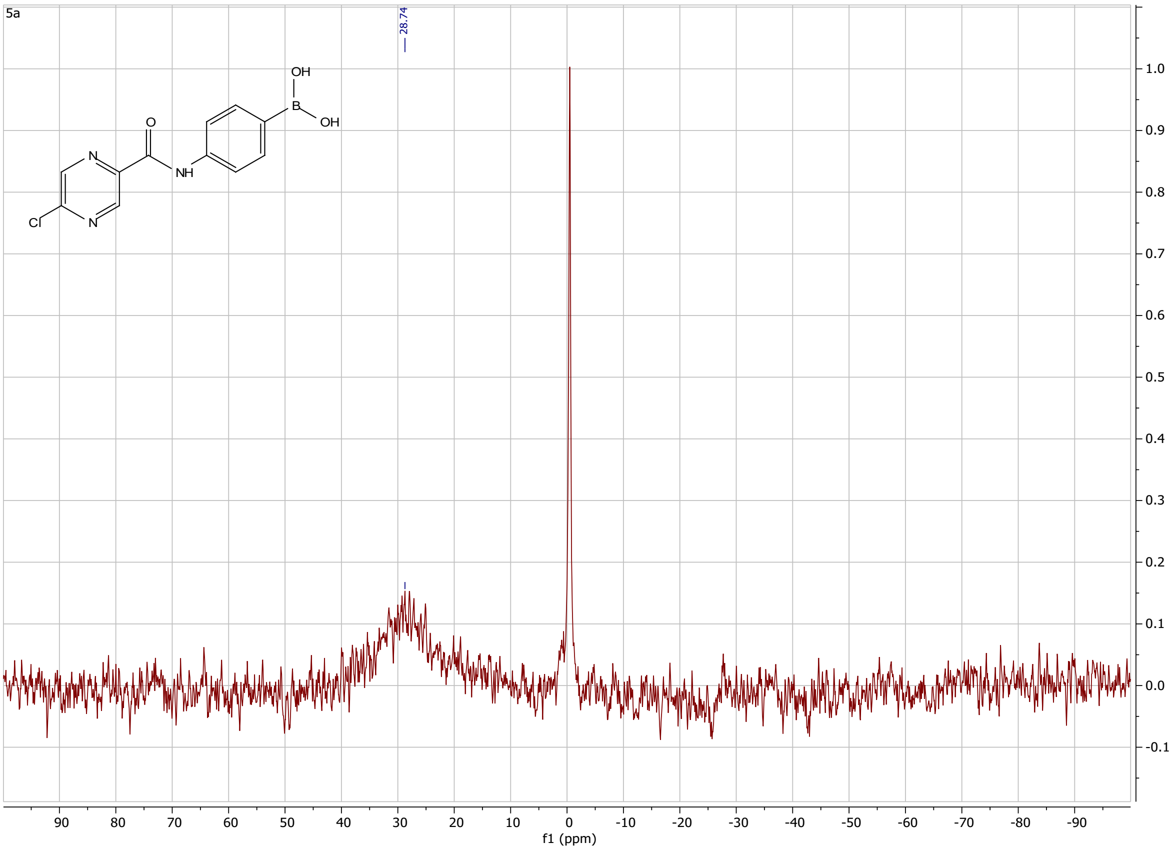

6a ^{1}H NMR spectrum

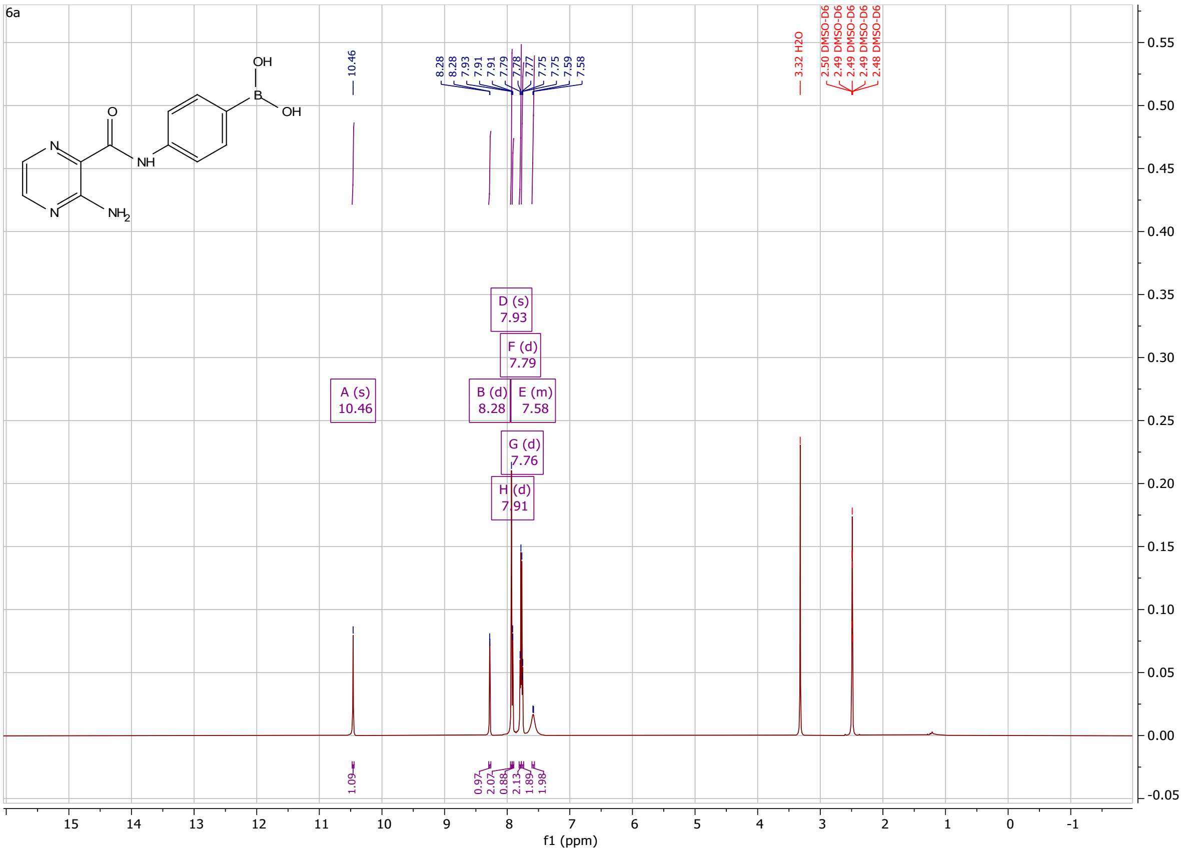

6a ^{13}C NMR spectrum

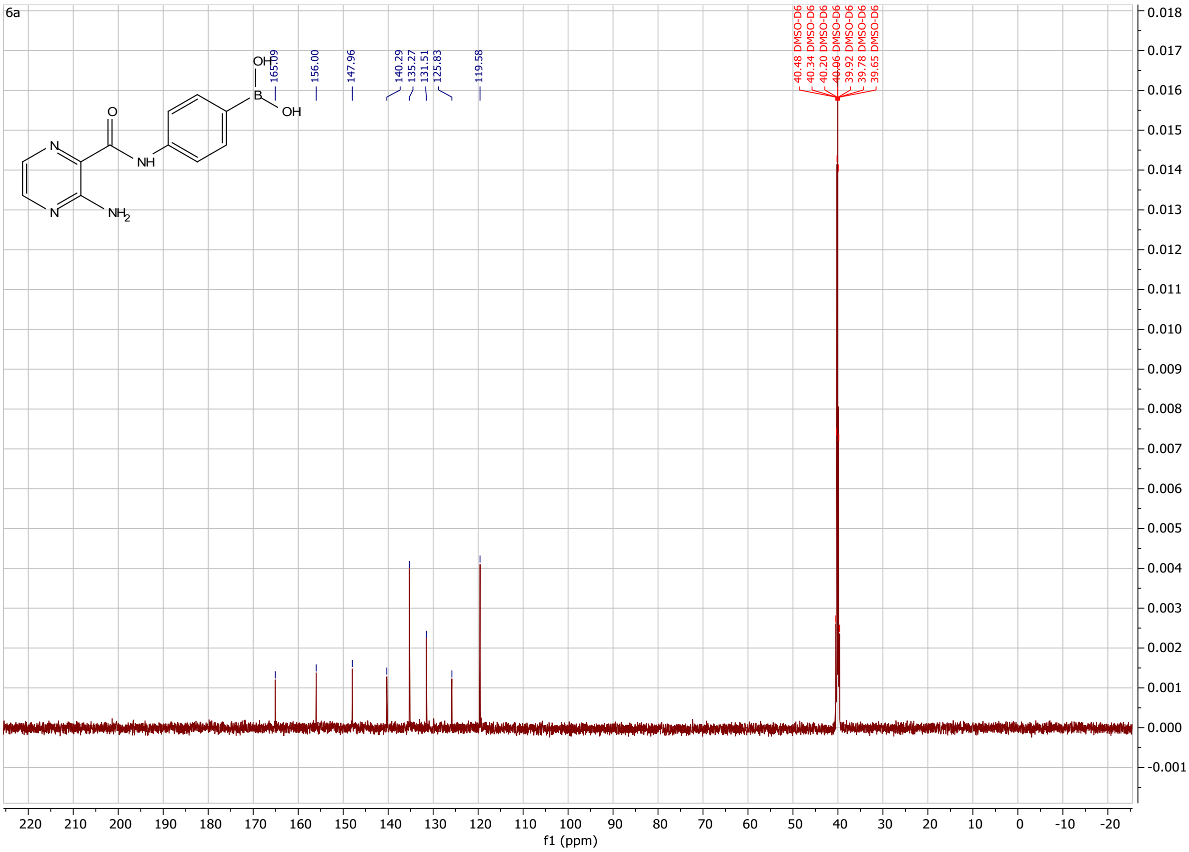

6a ^{11}B NMR spectrum

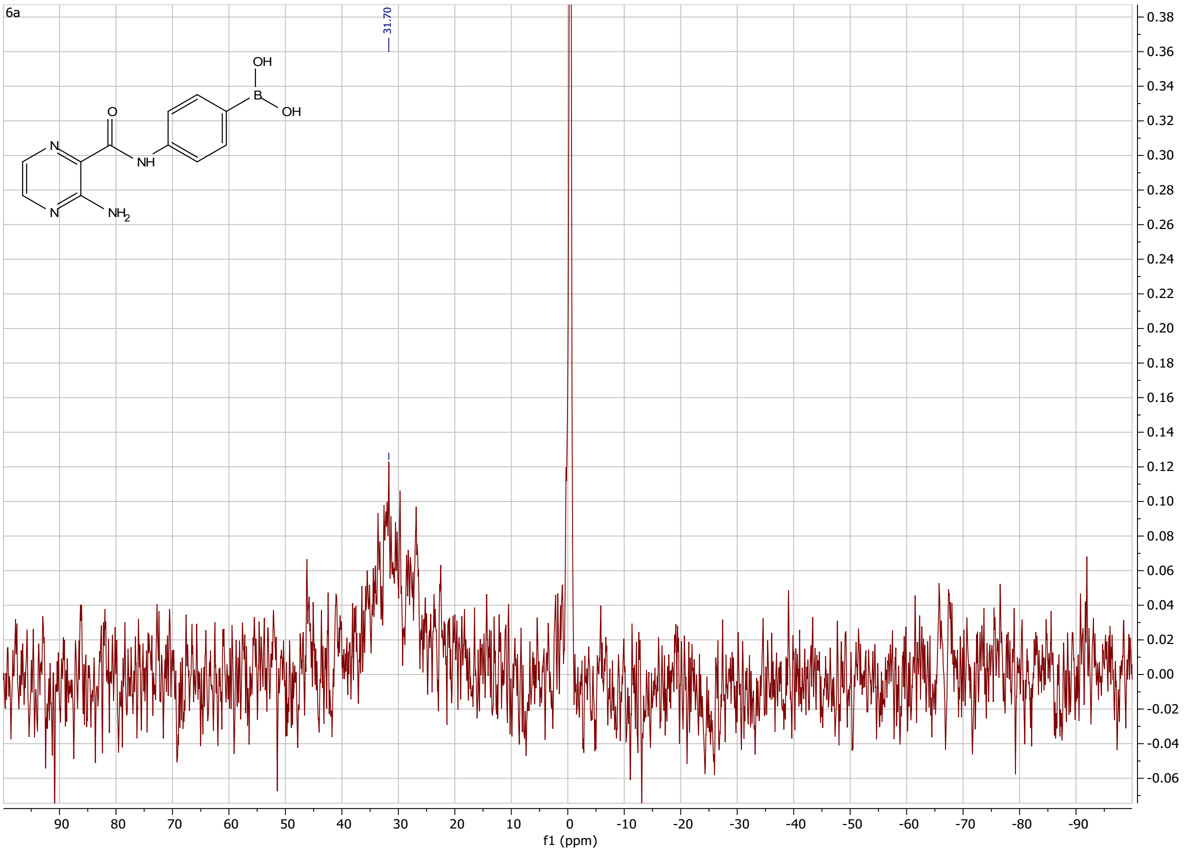

7a ^{1}H NMR spectrum

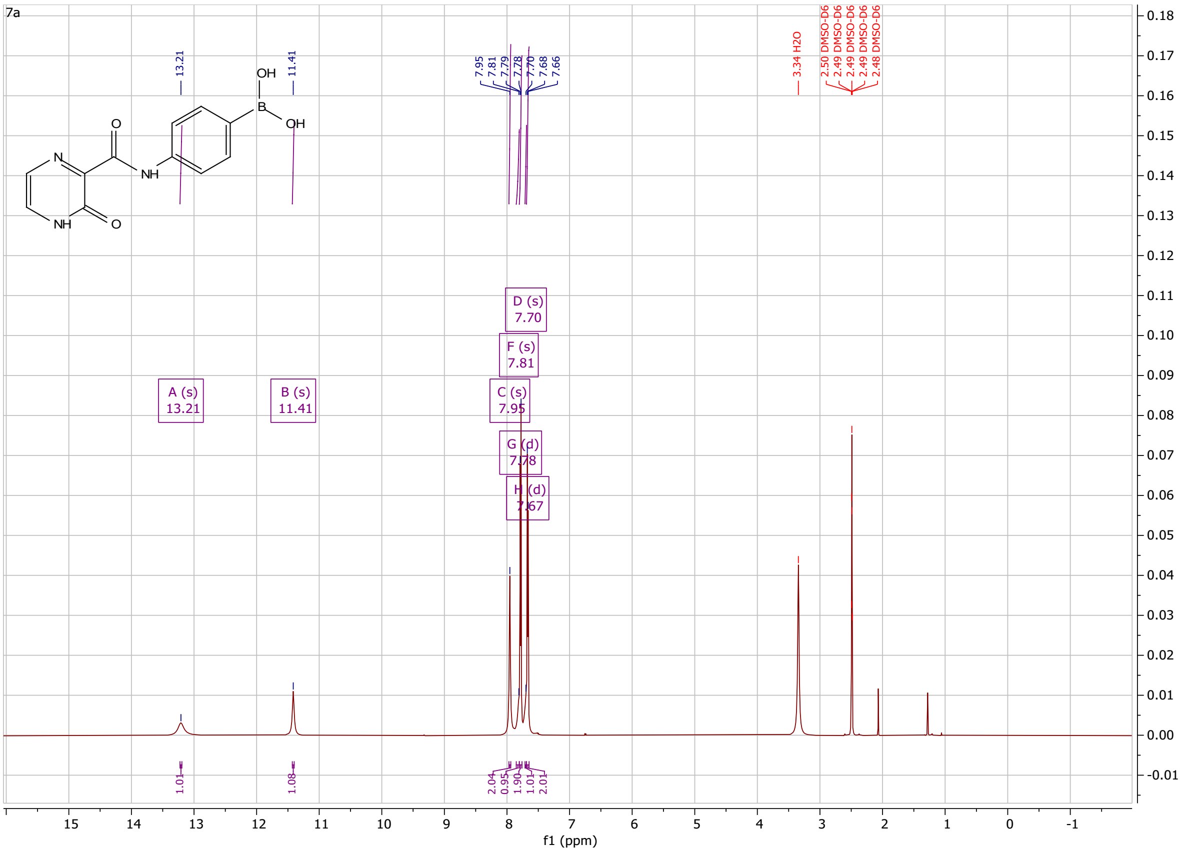

7a ^{11}B NMR spectrum

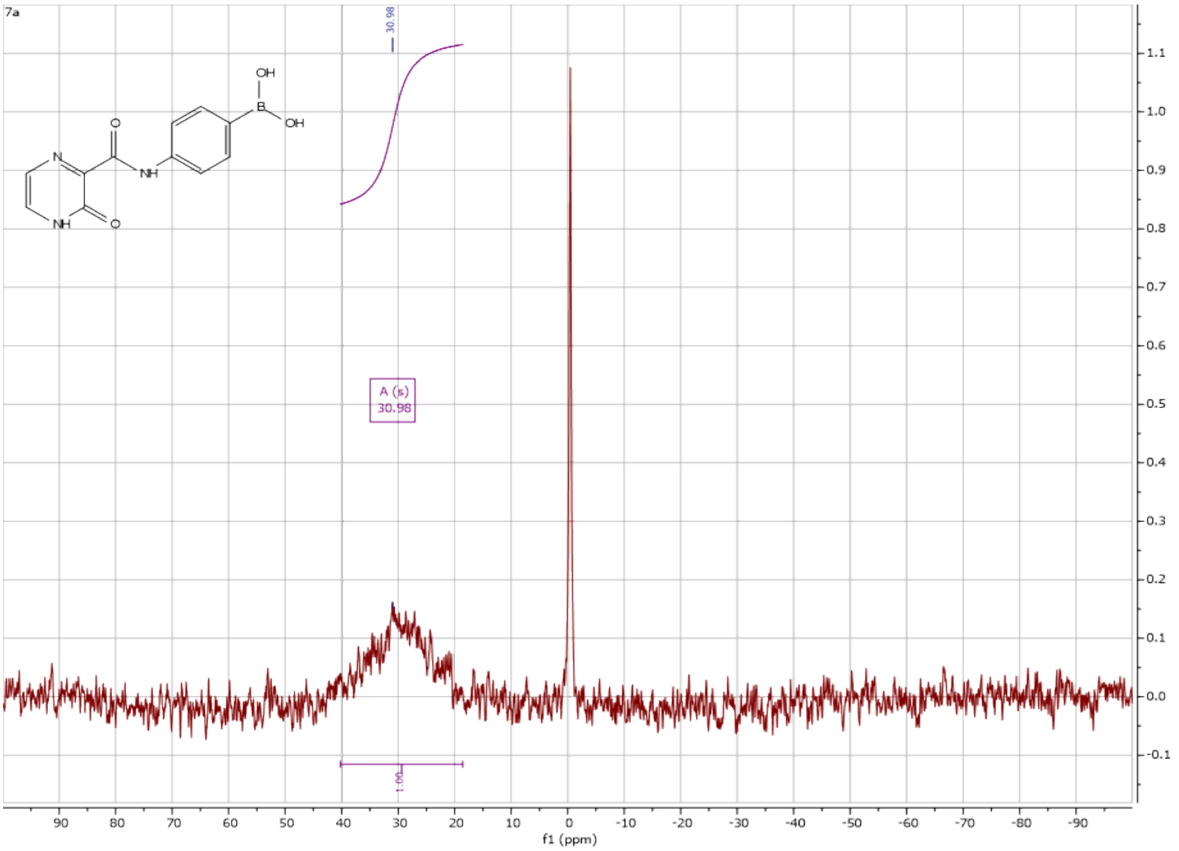

8a ^{1}H NMR spectrum

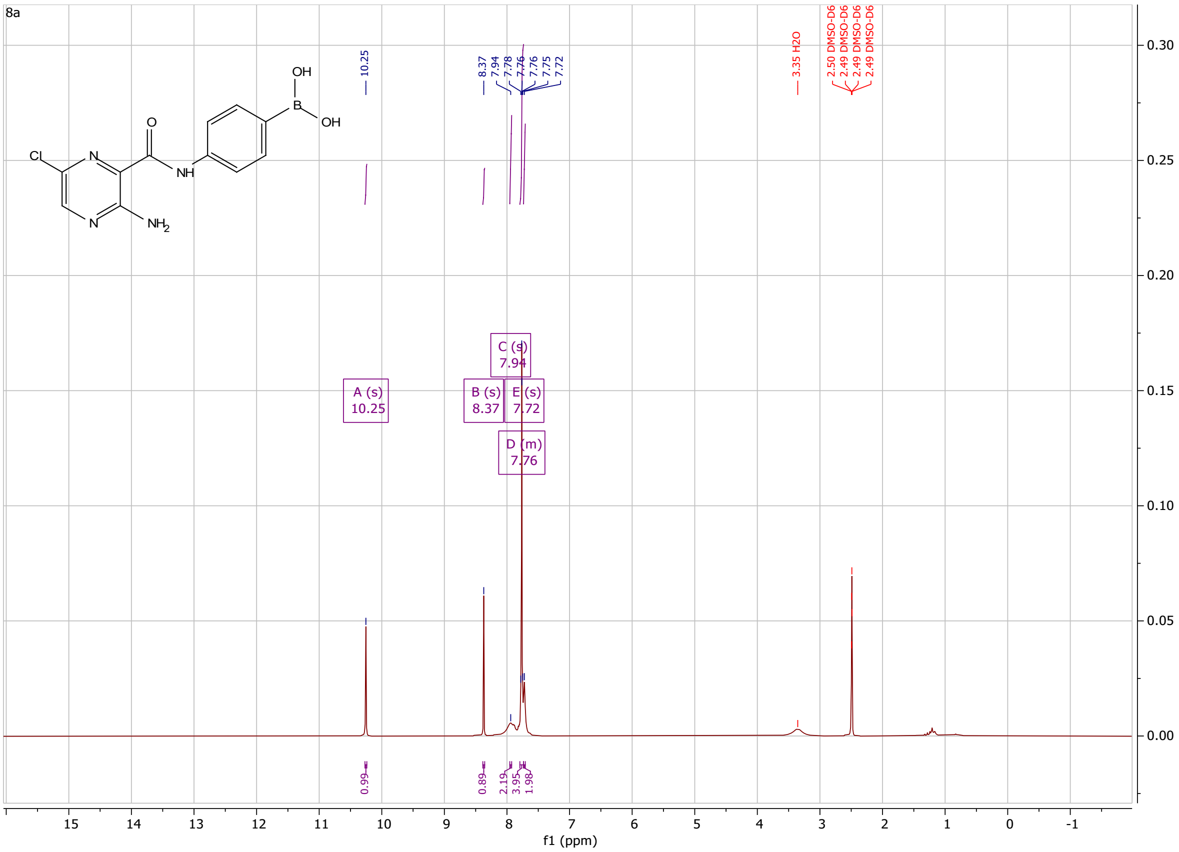

8a ^{13}C NMR spectrum

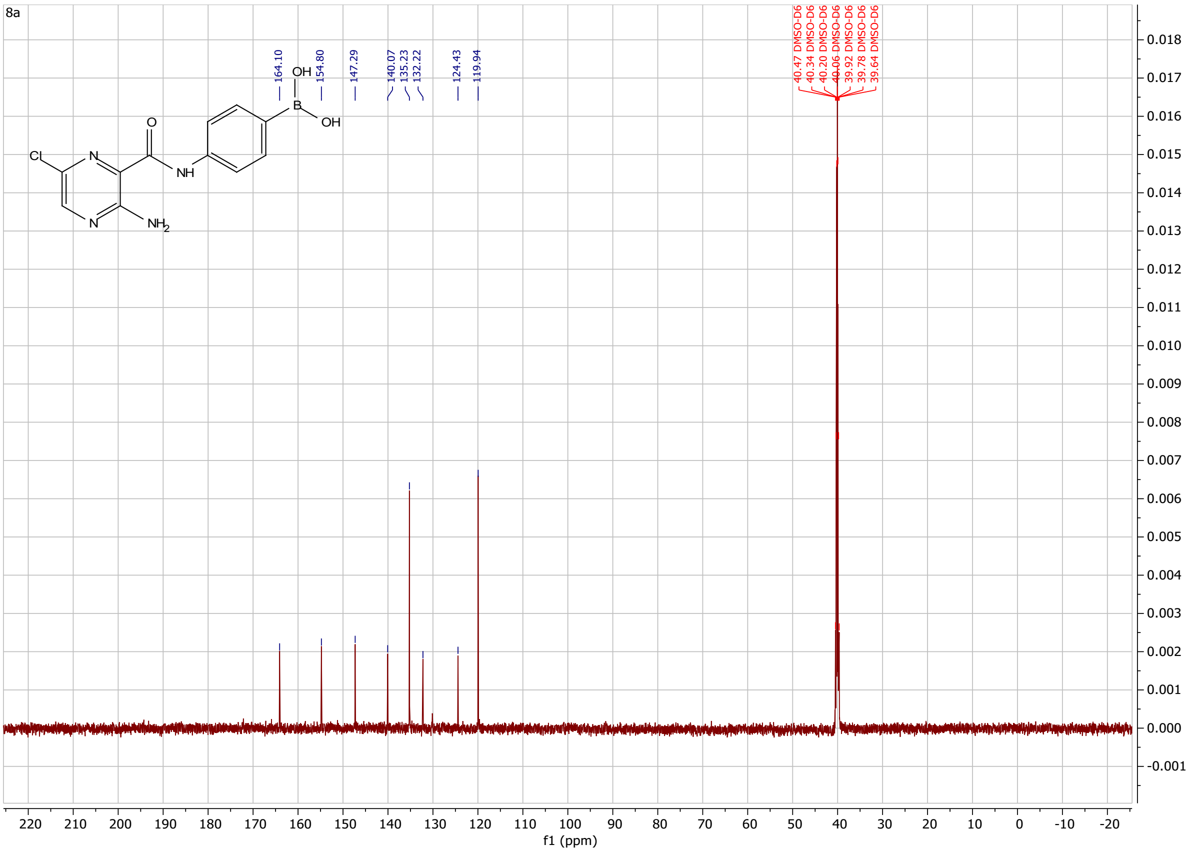

8a ^{11}B NMR spectrum

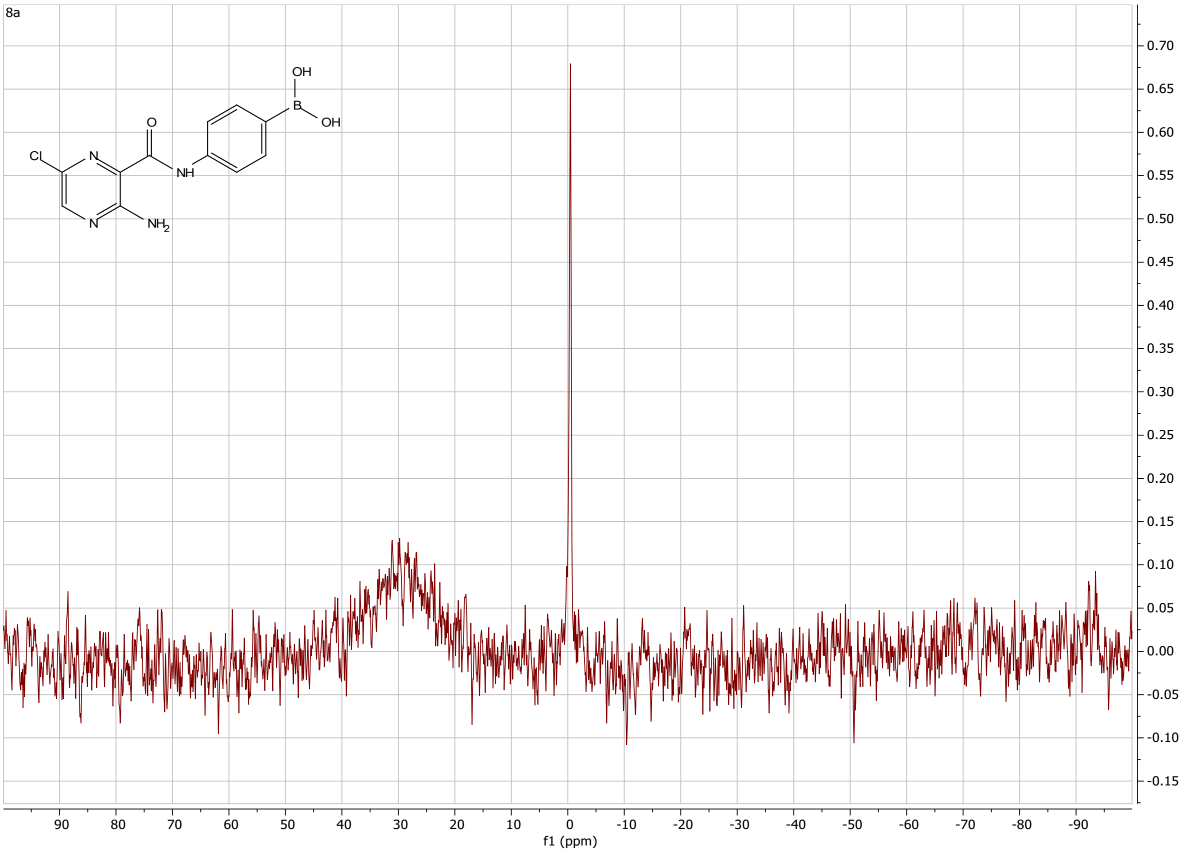

9a ^{1}H NMR spectrum

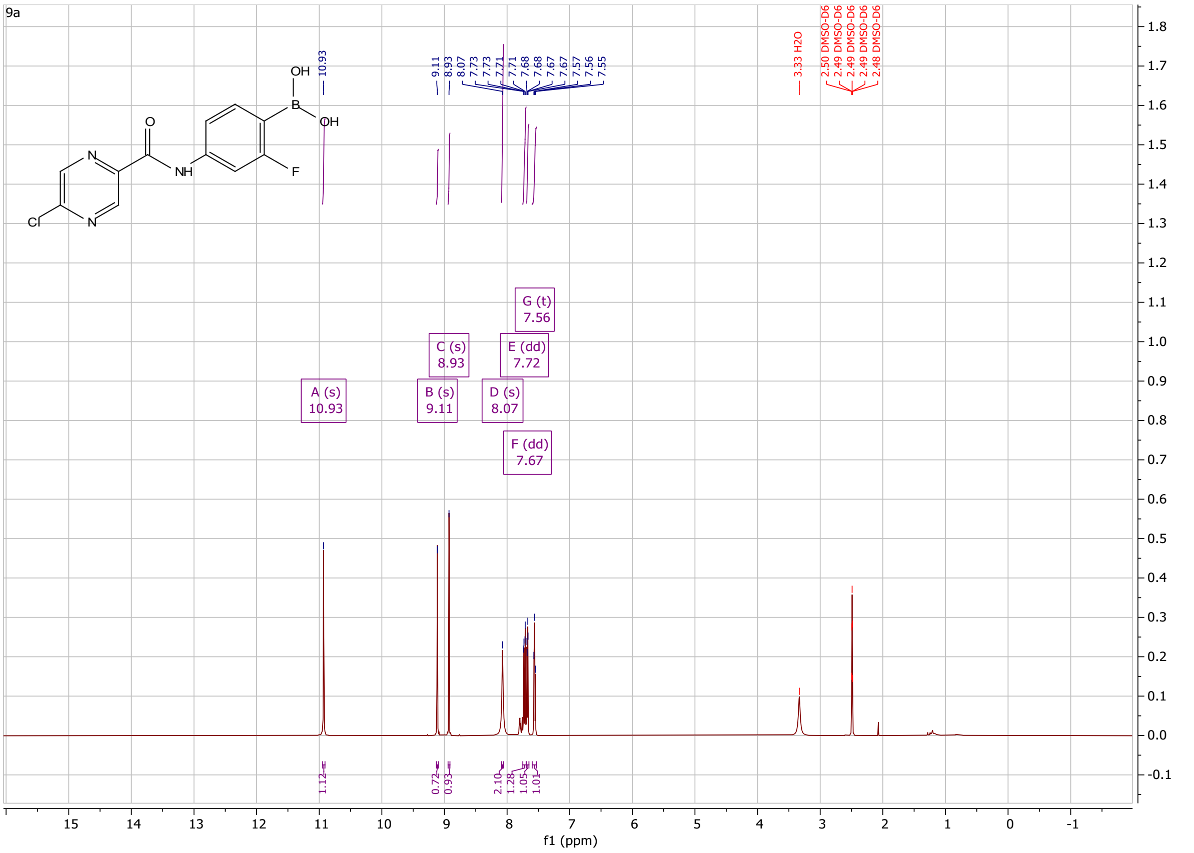

9a ^{13}C NMR spectrum

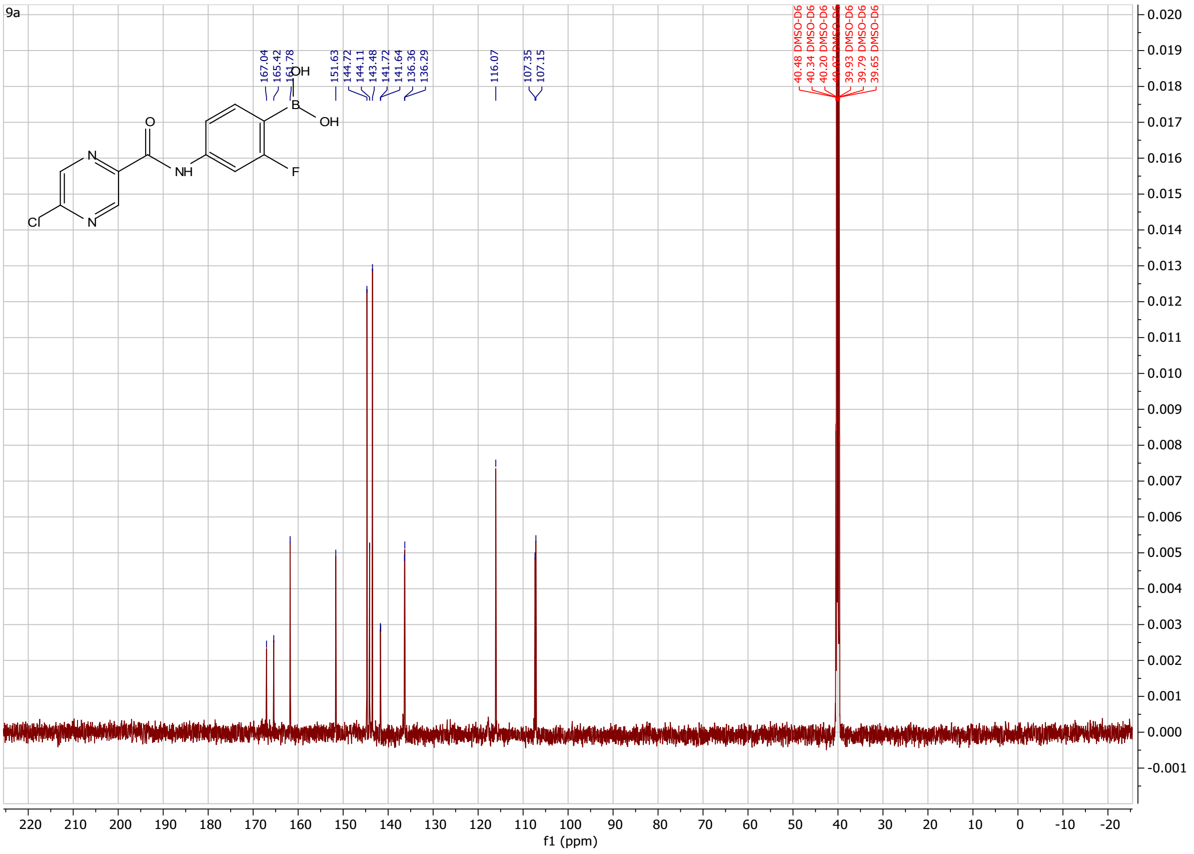

9a ^{11}B NMR spectrum

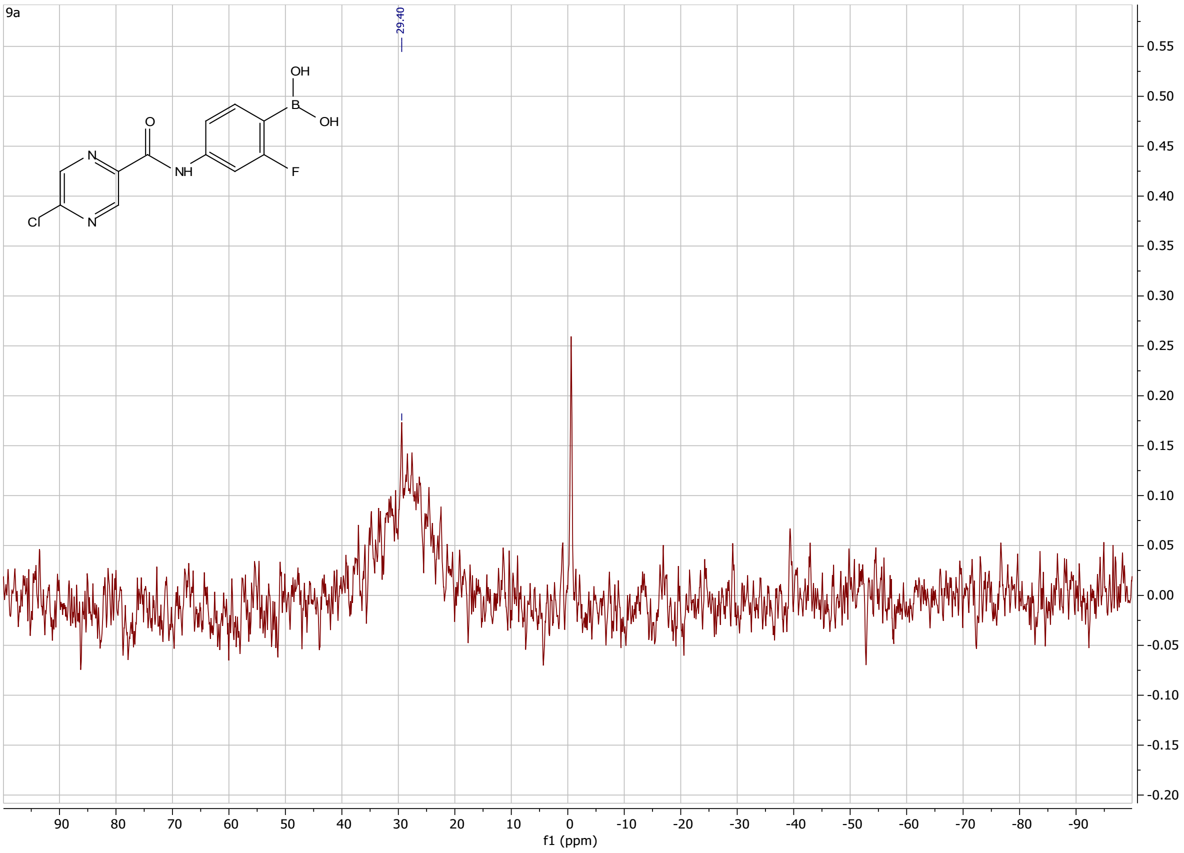

10a ^{1}H NMR spectrum

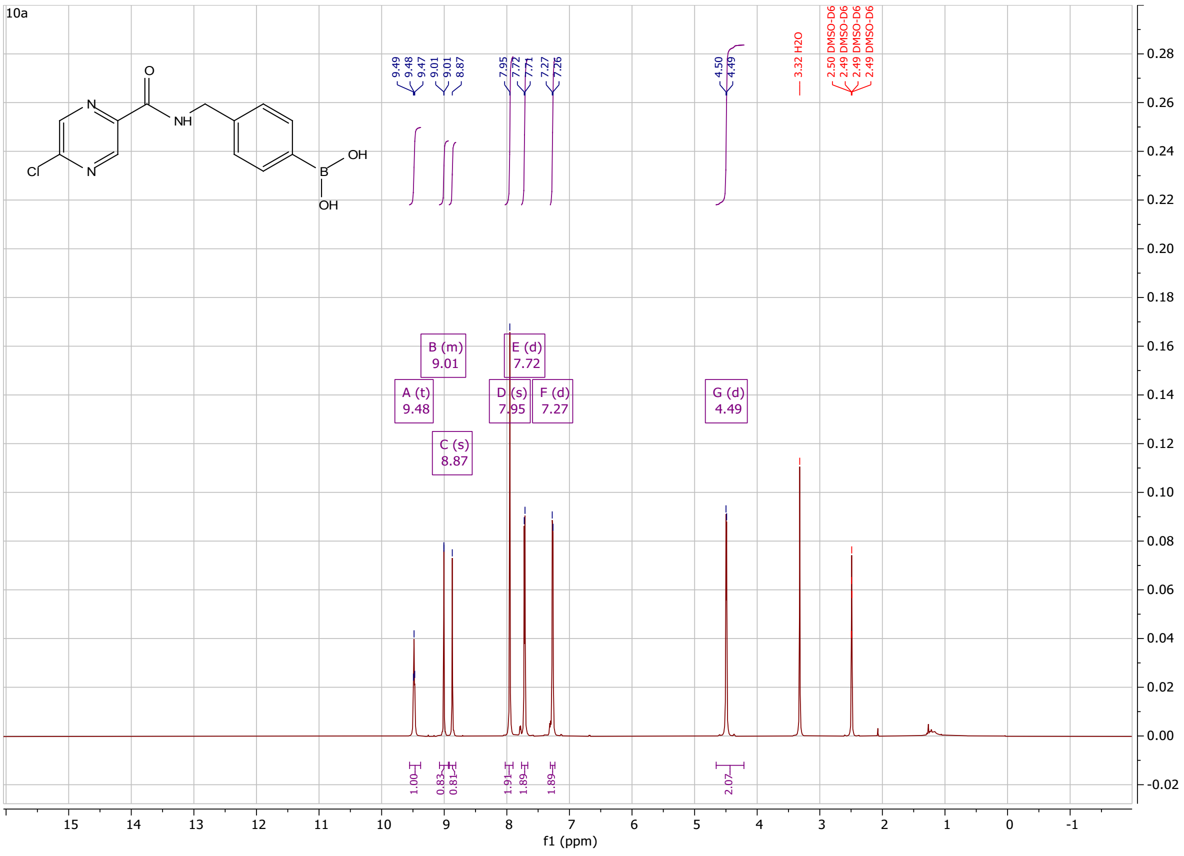

10a ^{13}C NMR spectru

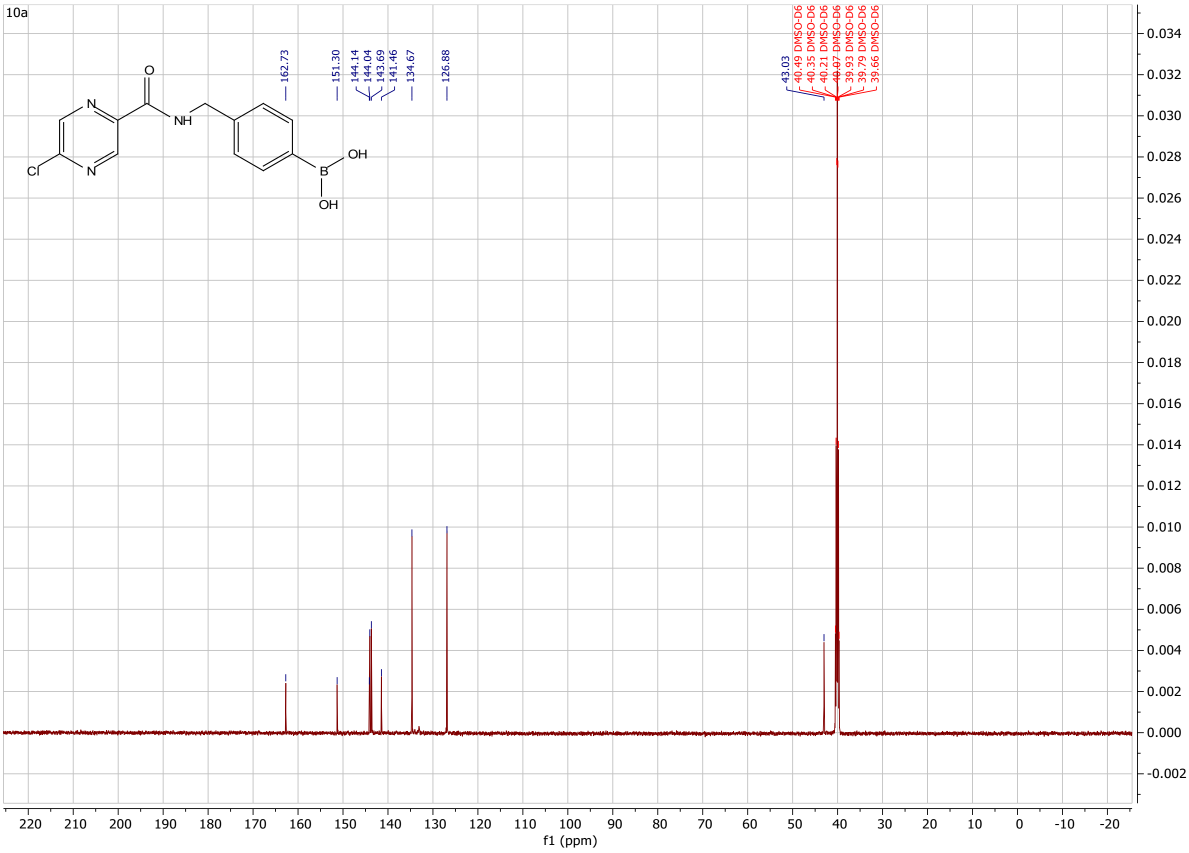

10a ^{11}B NMR spectrum

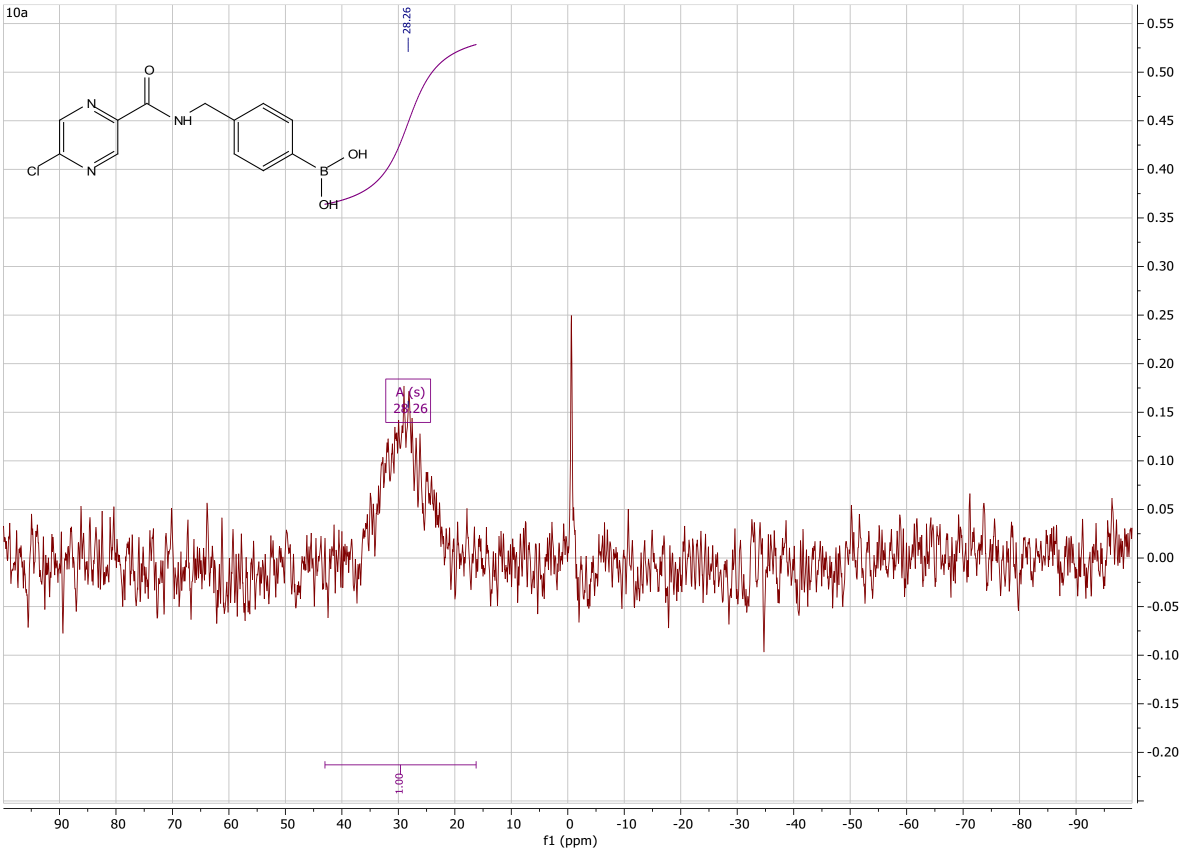

11a ^{1}H NMR spectrum

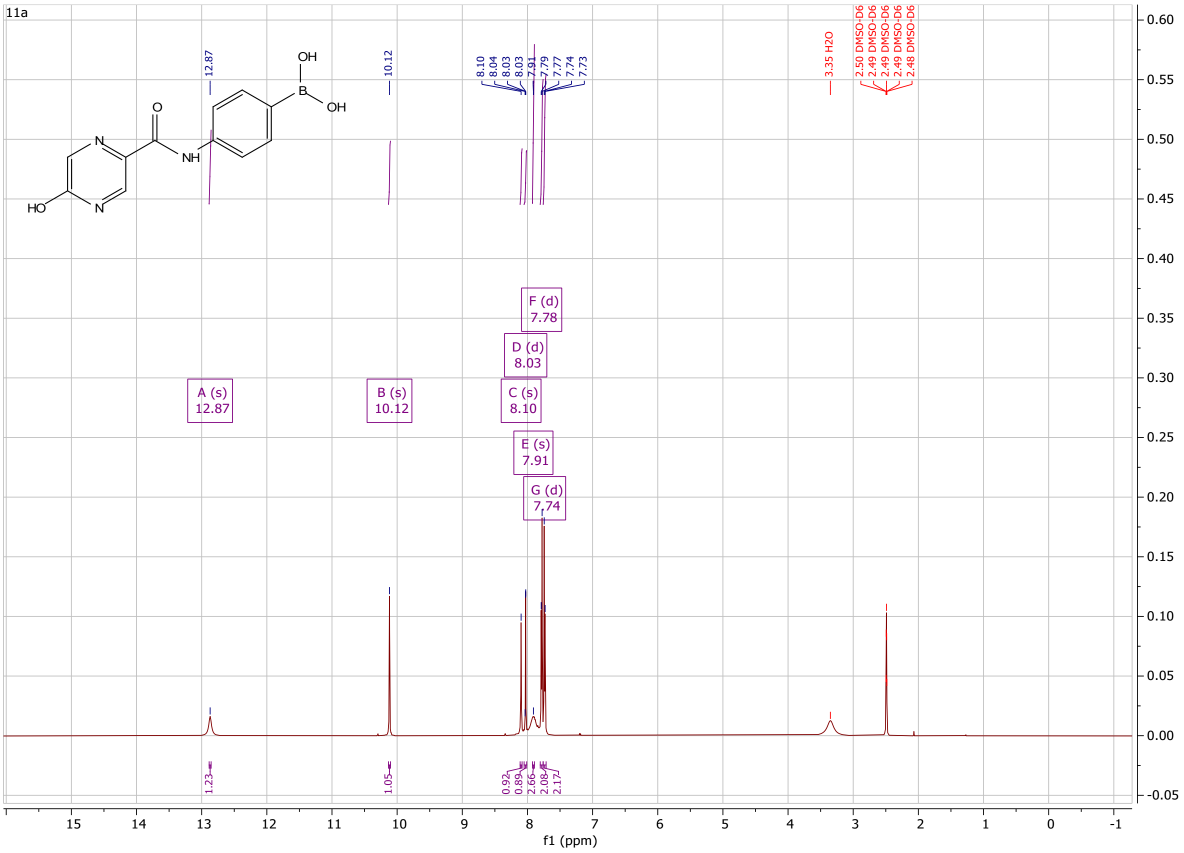

11a ^{13}C NMR spectrum

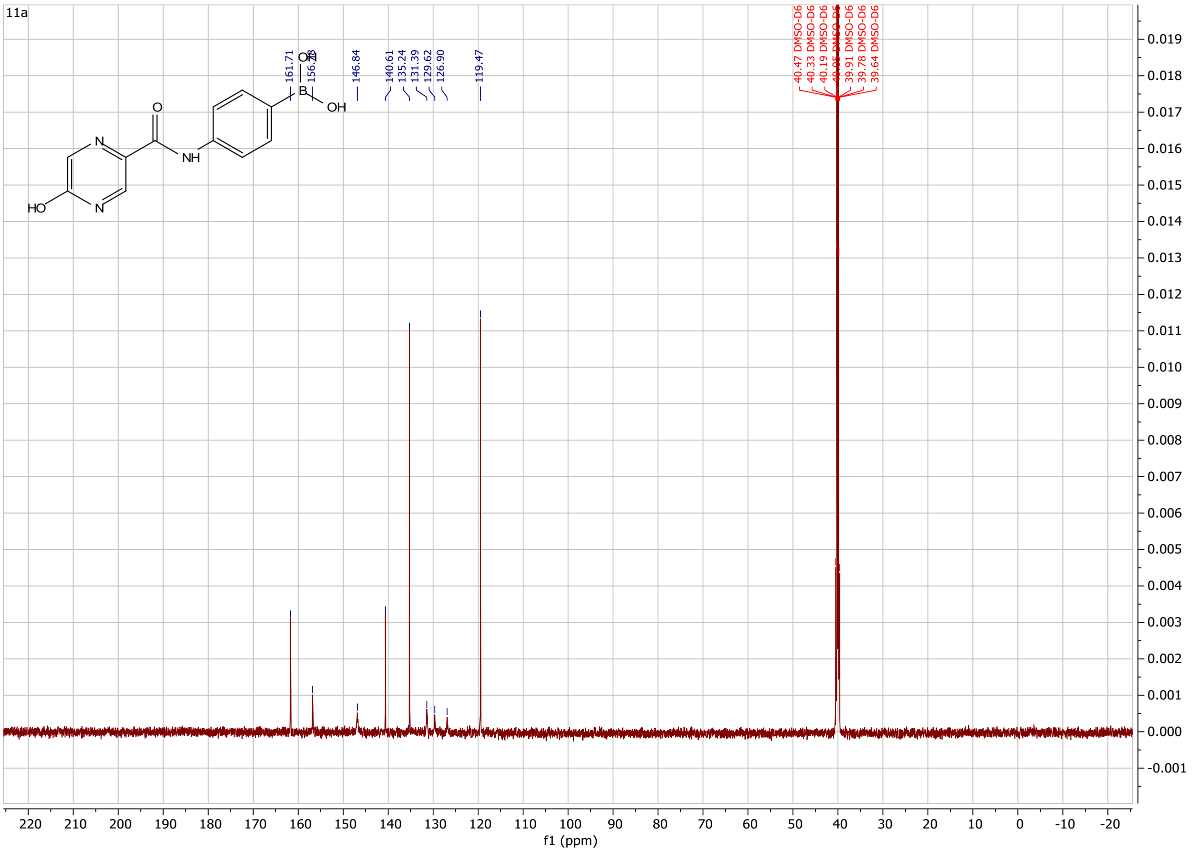

11a ^{11}B NMR spectrum

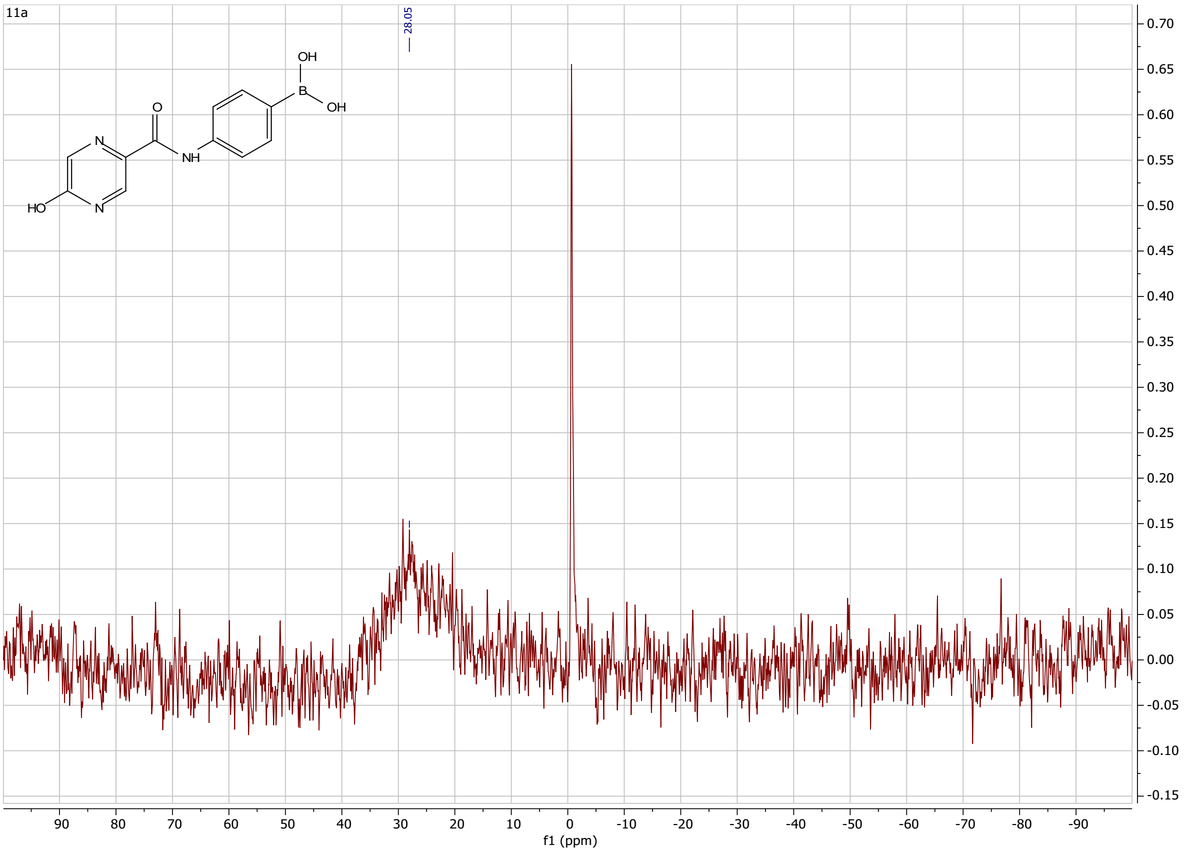

12a ^{1}H NMR spectrum

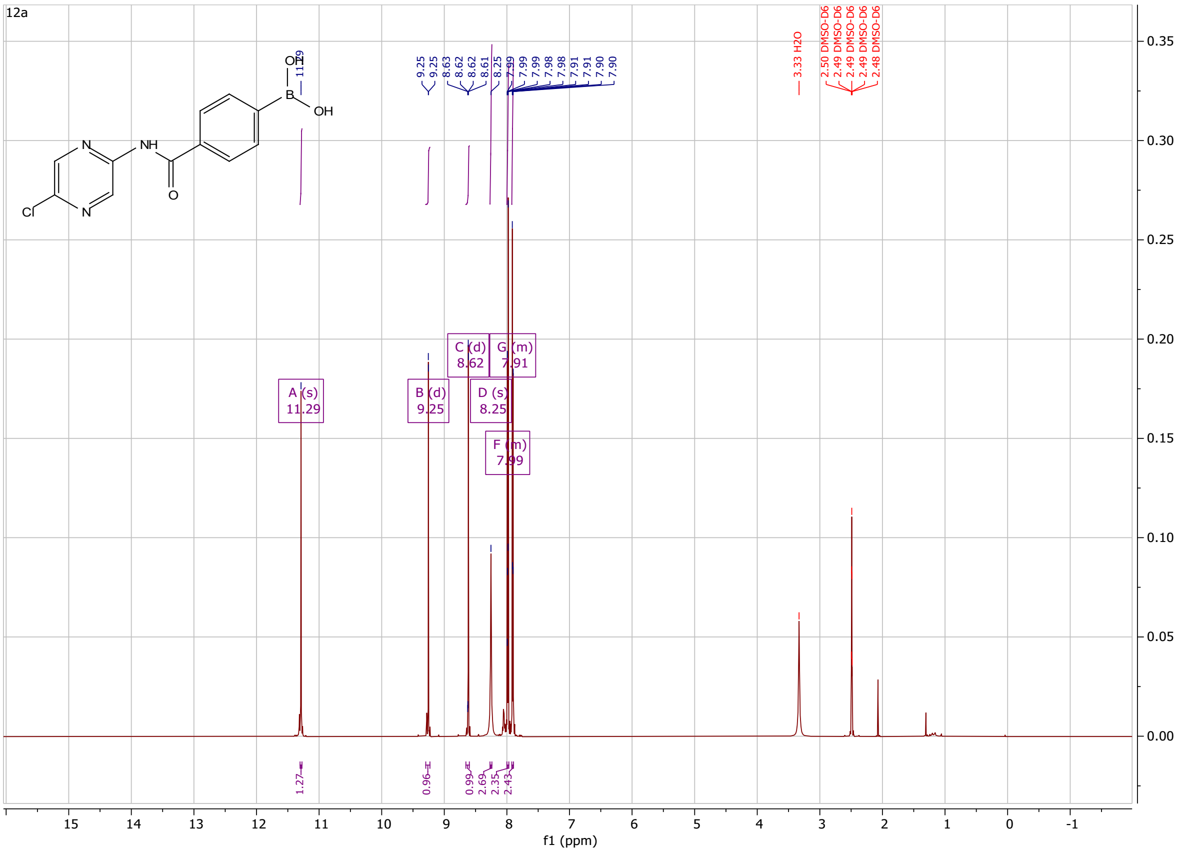

12a ^{13}C NMR spectrum

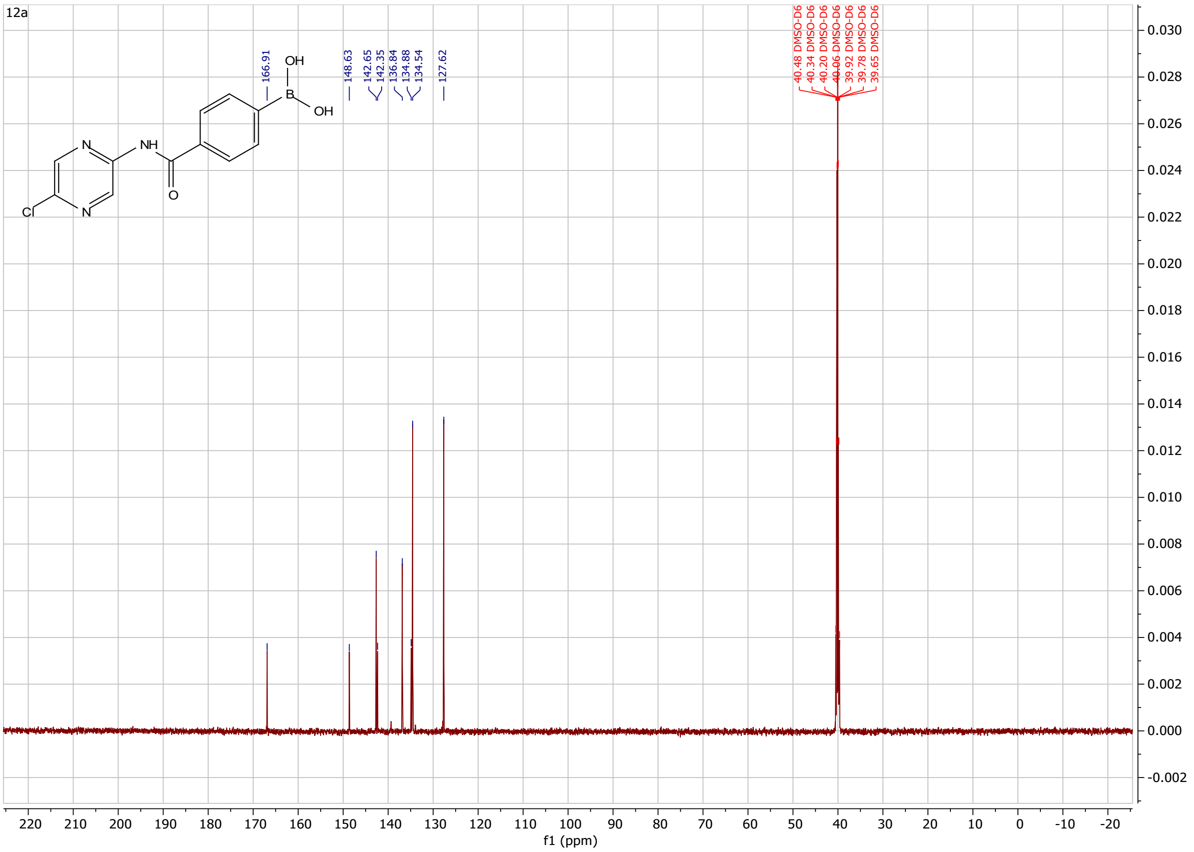

12a ^{11}B NMR spectrum

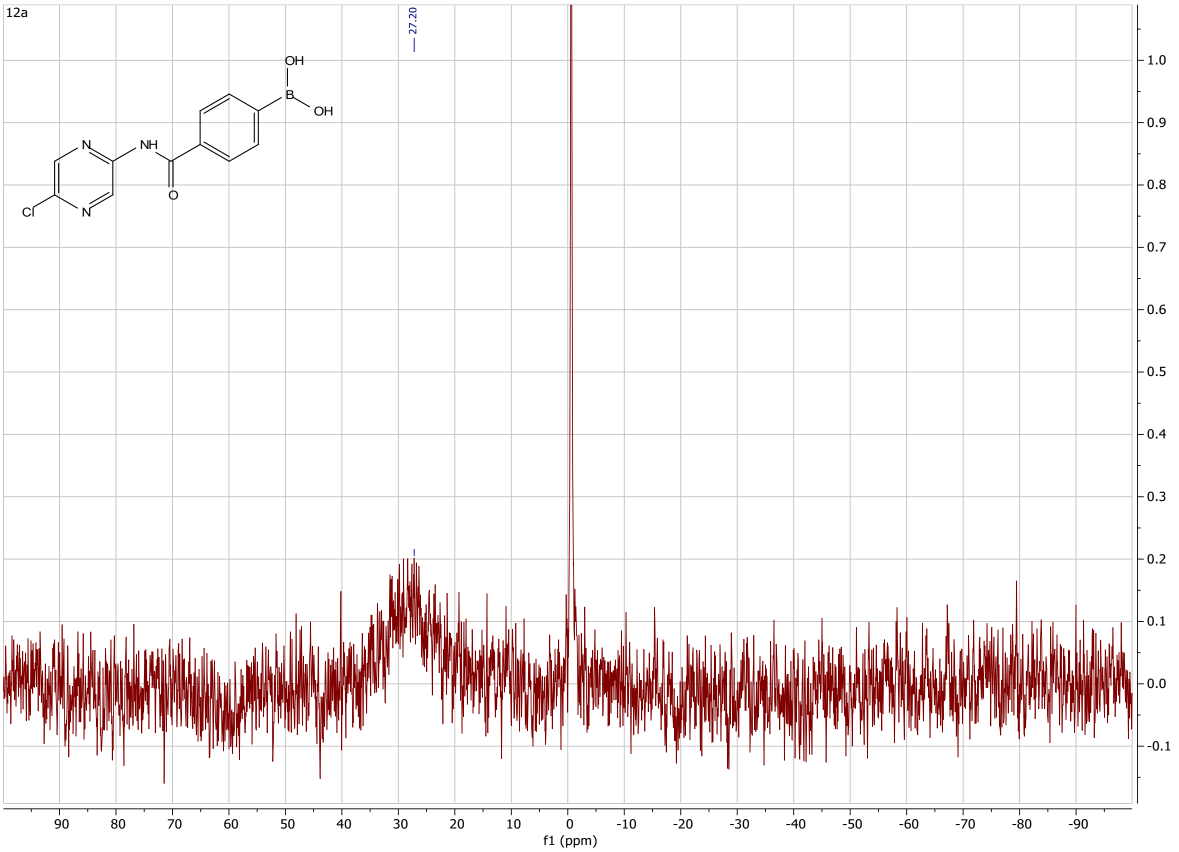

13a ^{1}H NMR spectrum

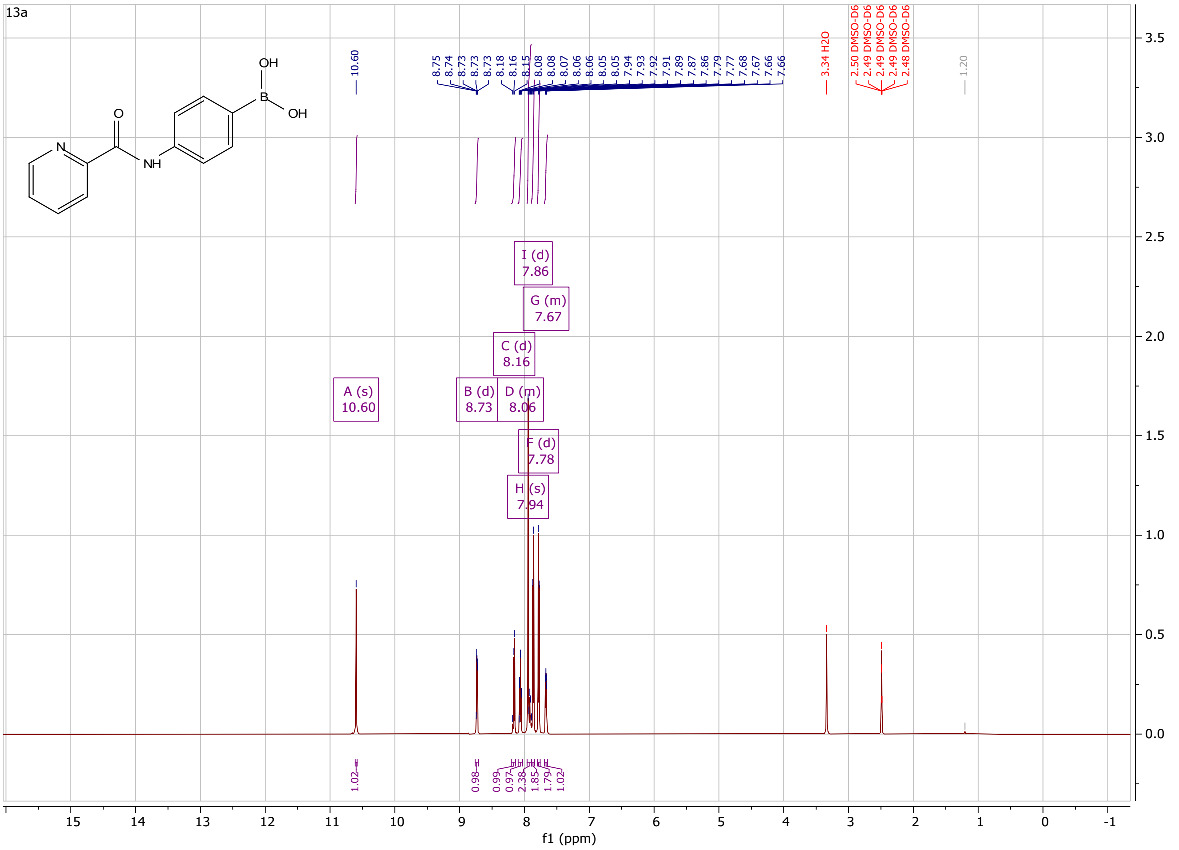

13a ^{13}C NMR spectrum

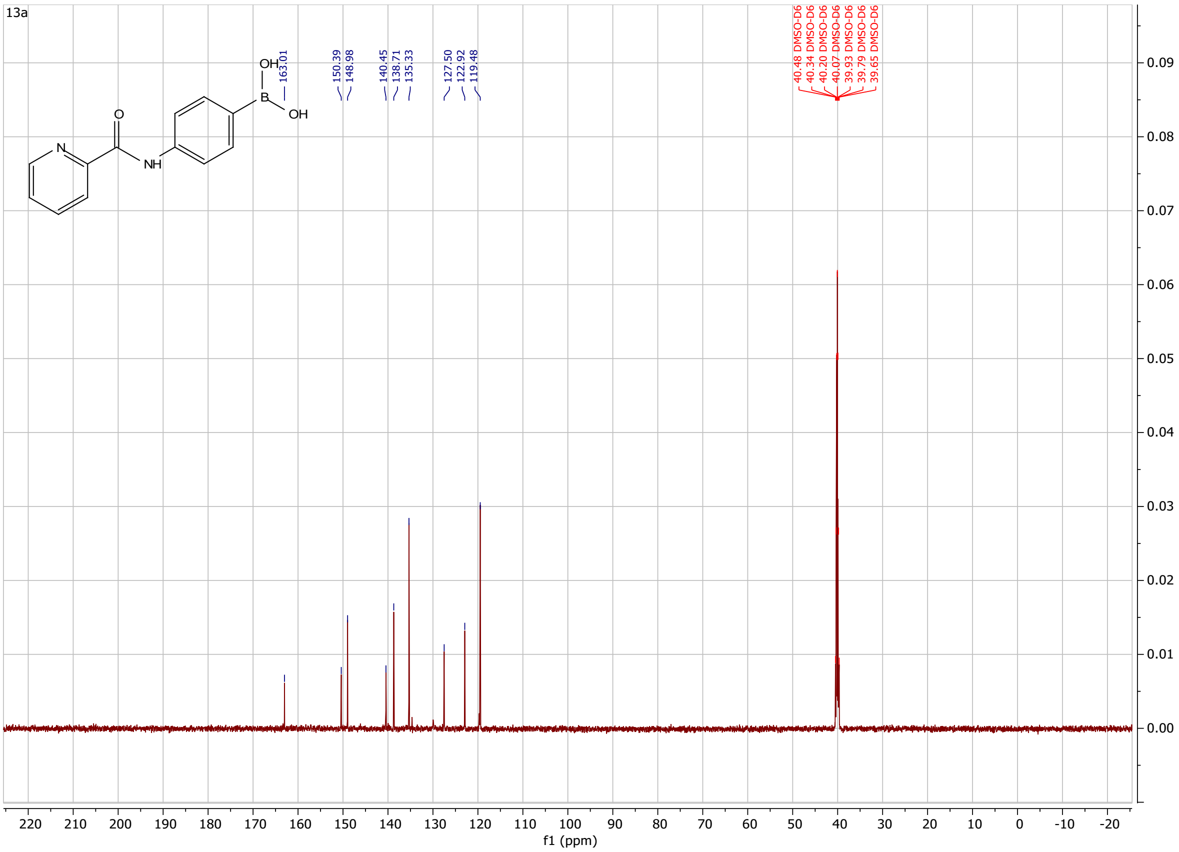

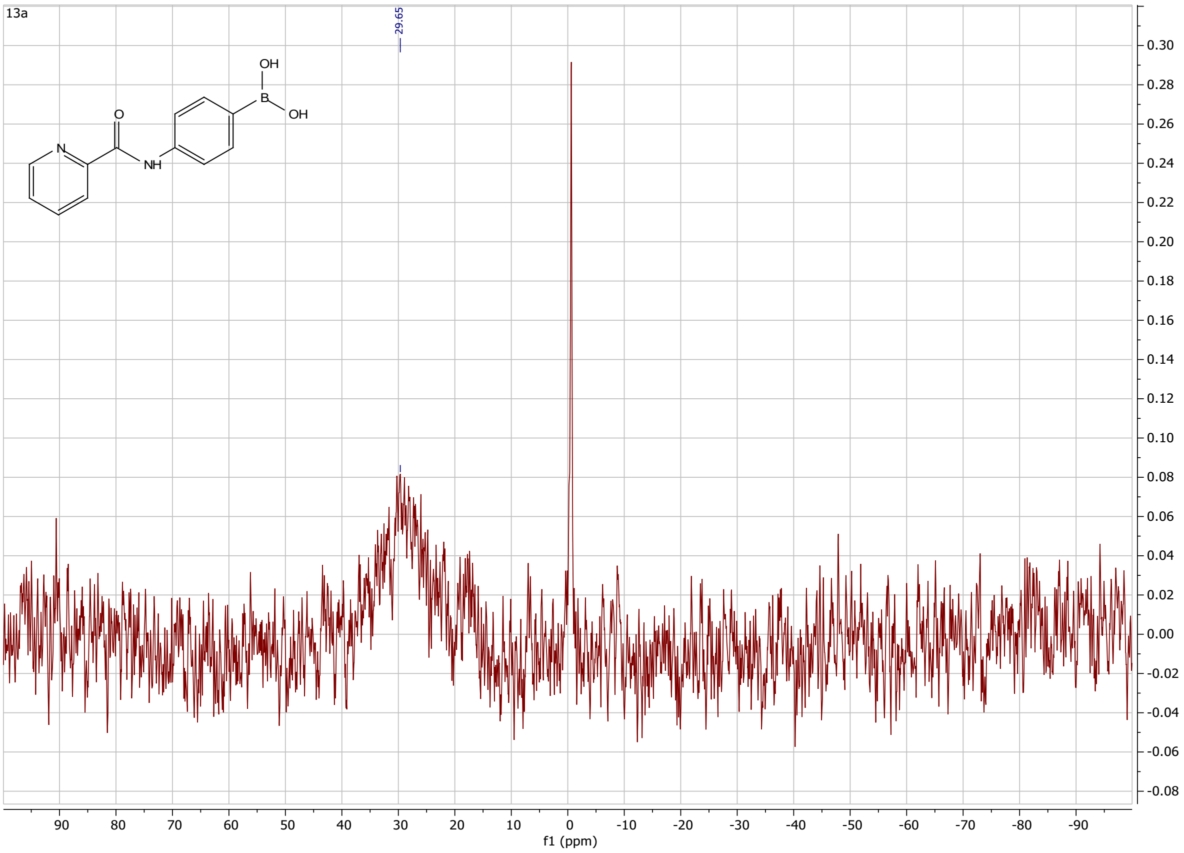

14a ^1H NMR spectrum

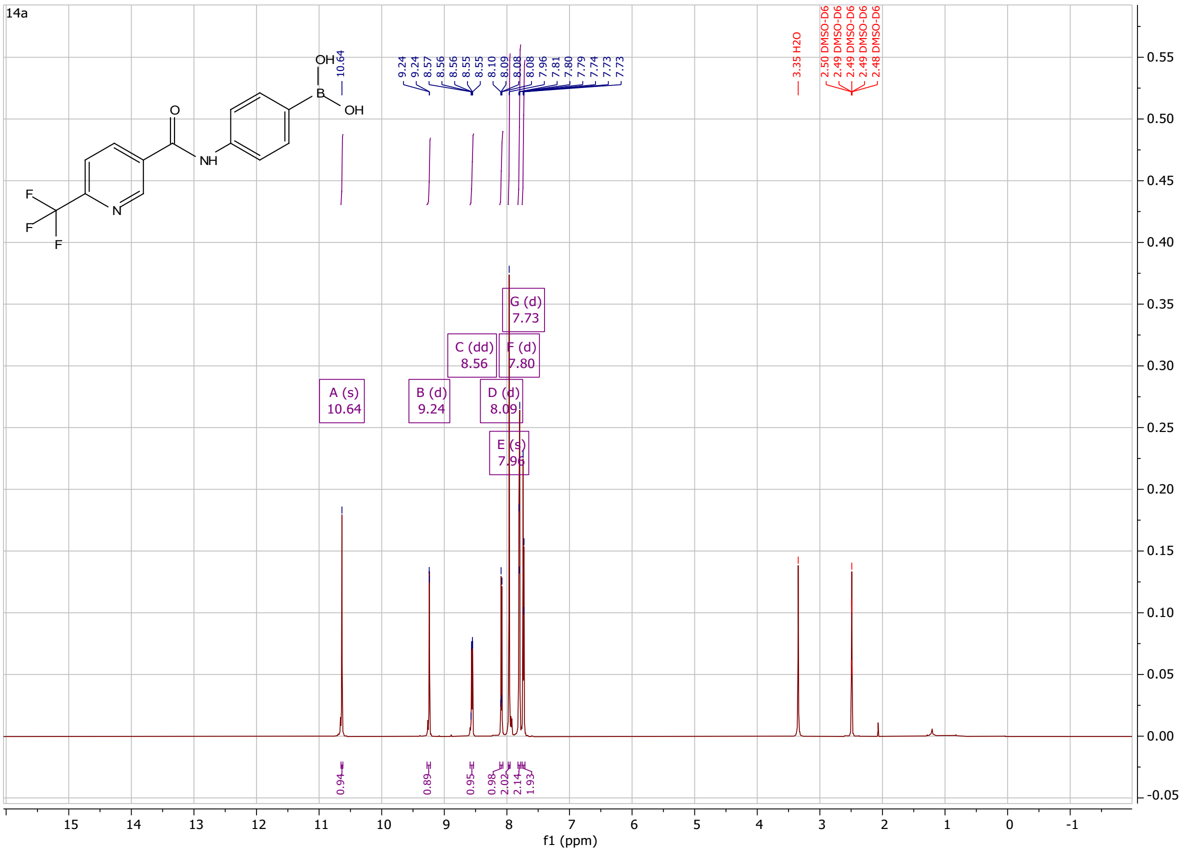

14a ^{13}C NMR spectrum

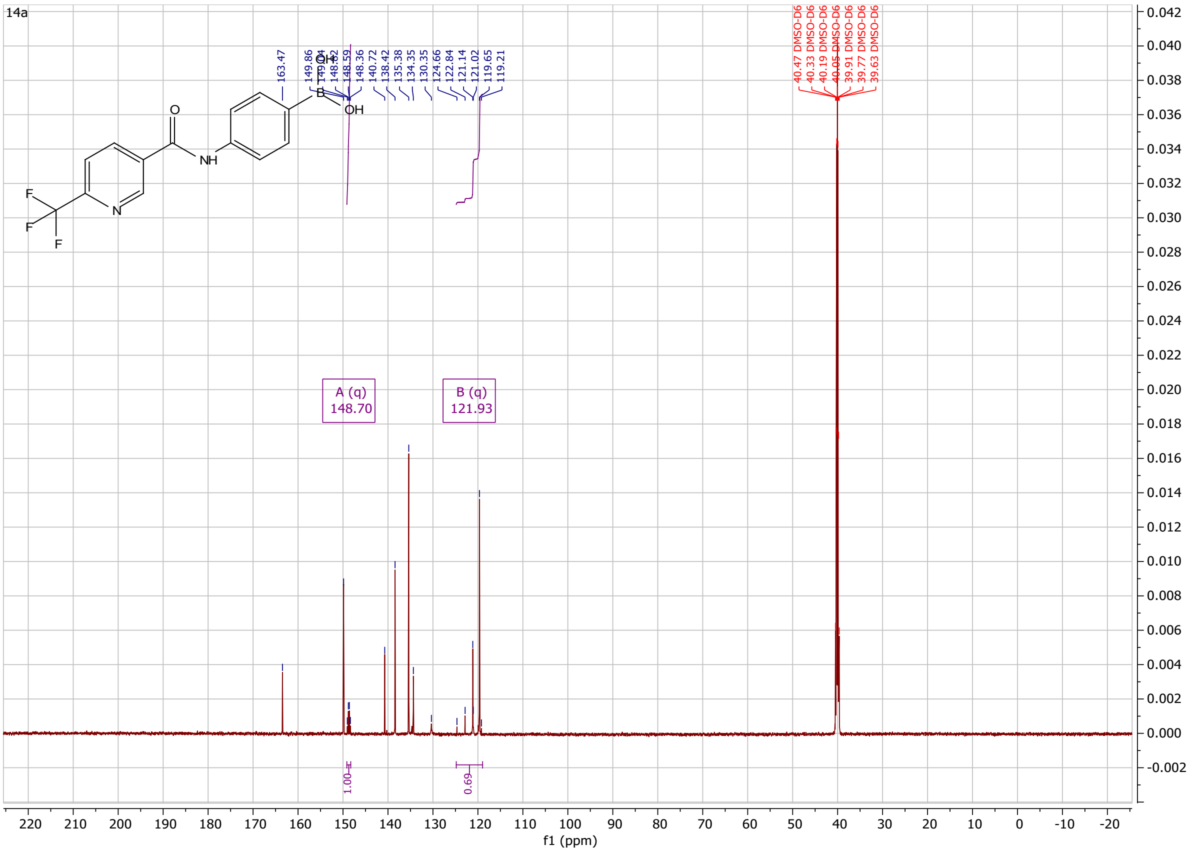

14a ^{11}B NMR spectrum

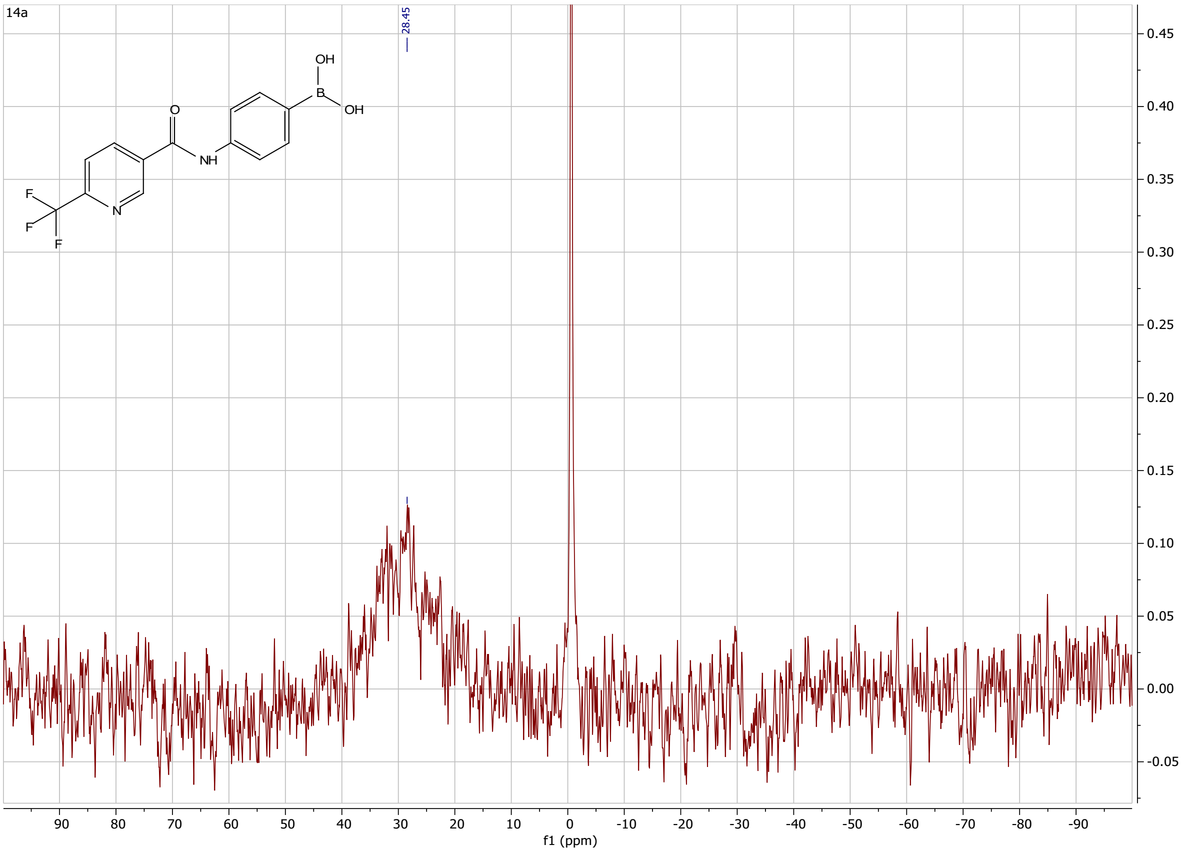

15a ^{1}H NMR spectrum

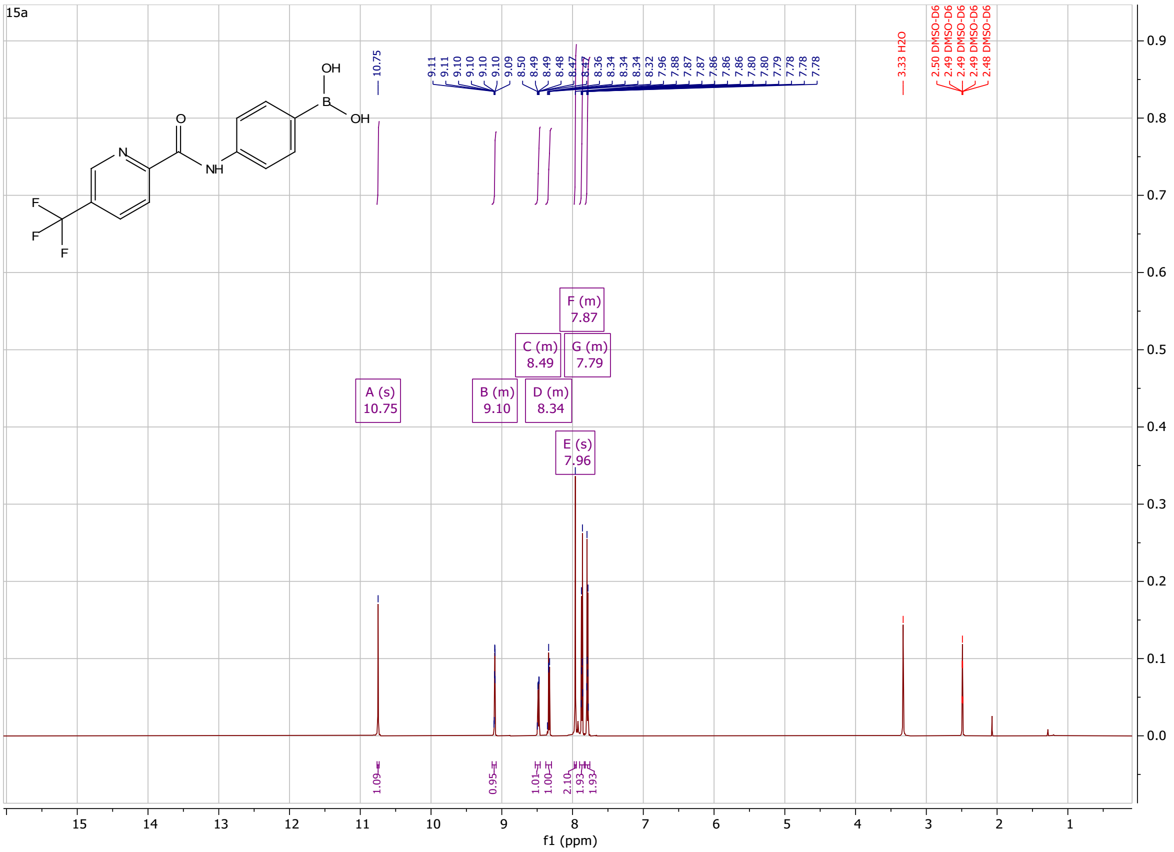

15a ^{13}C NMR spectrum

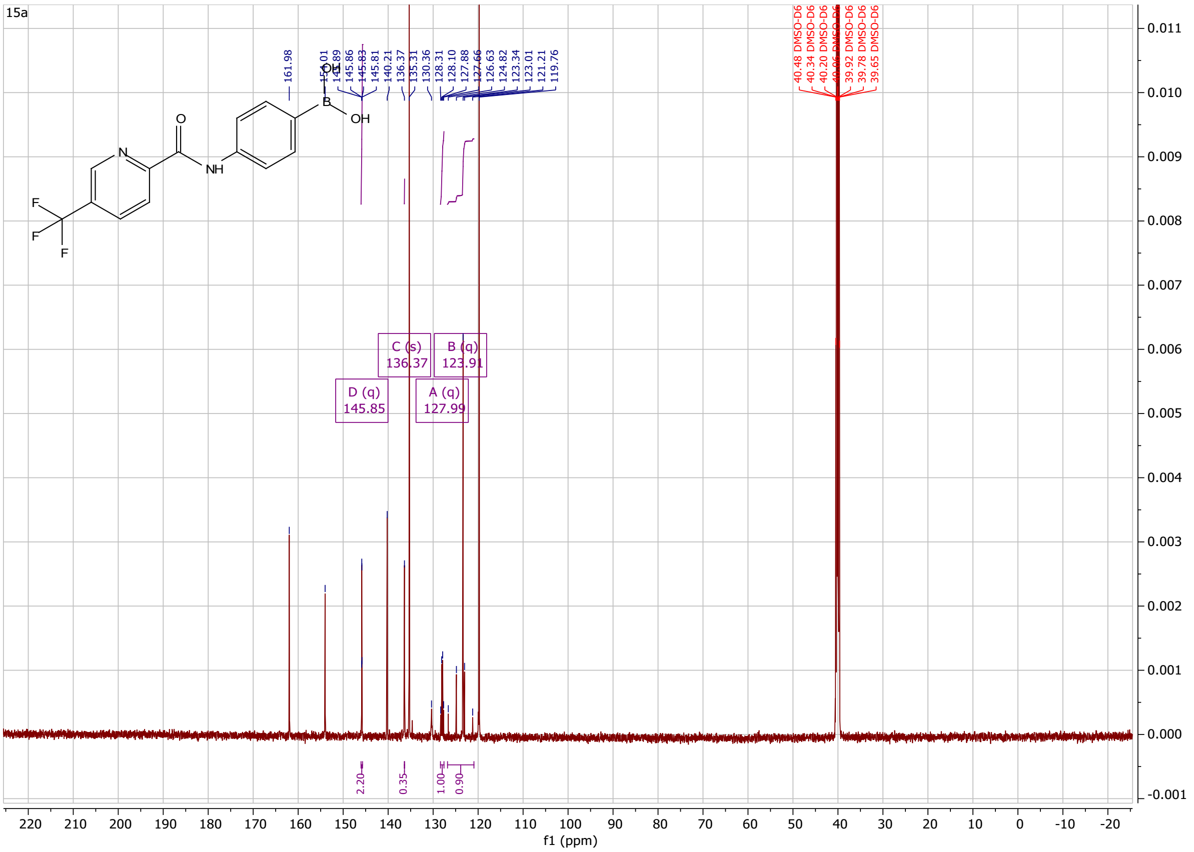

15a ^{11}B NMR spectrum

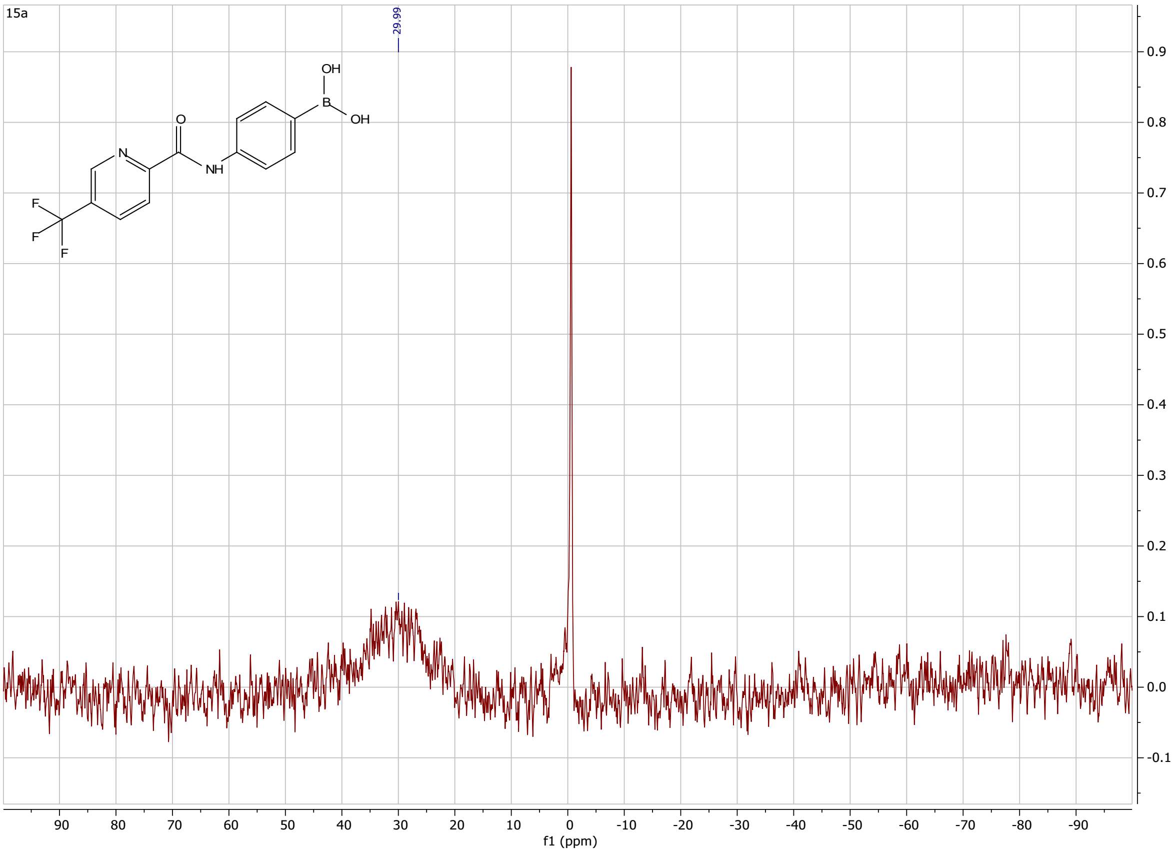

16a ¹H NMR spectrum

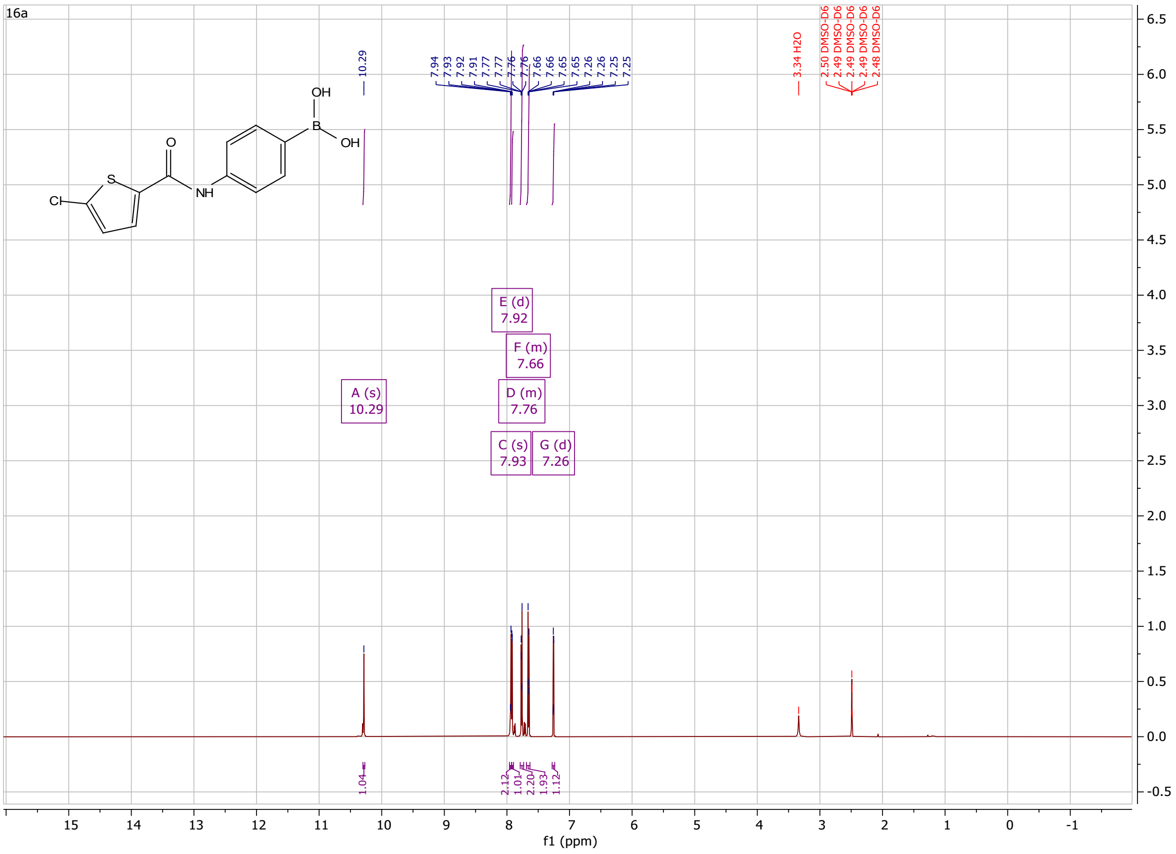

16a ^{13}C NMR spectrum

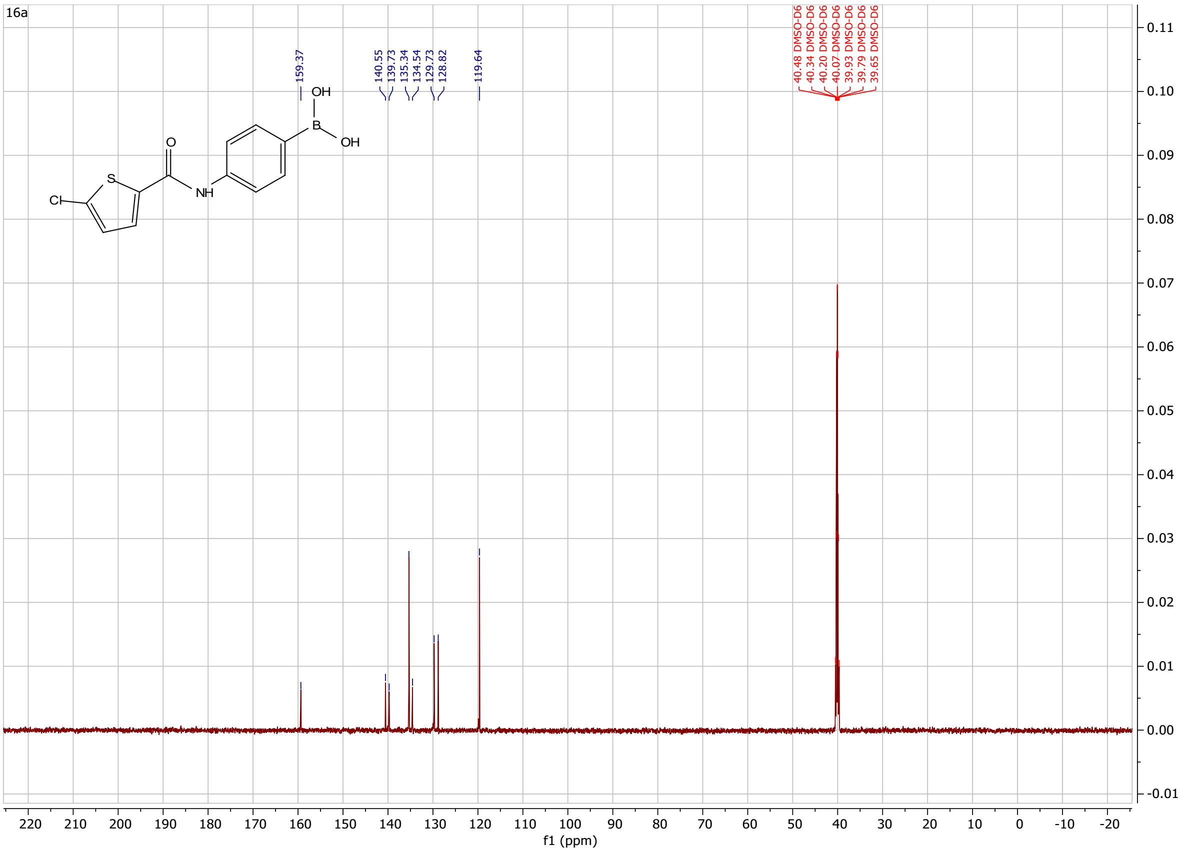

16a ^{11}B NMR spectrum

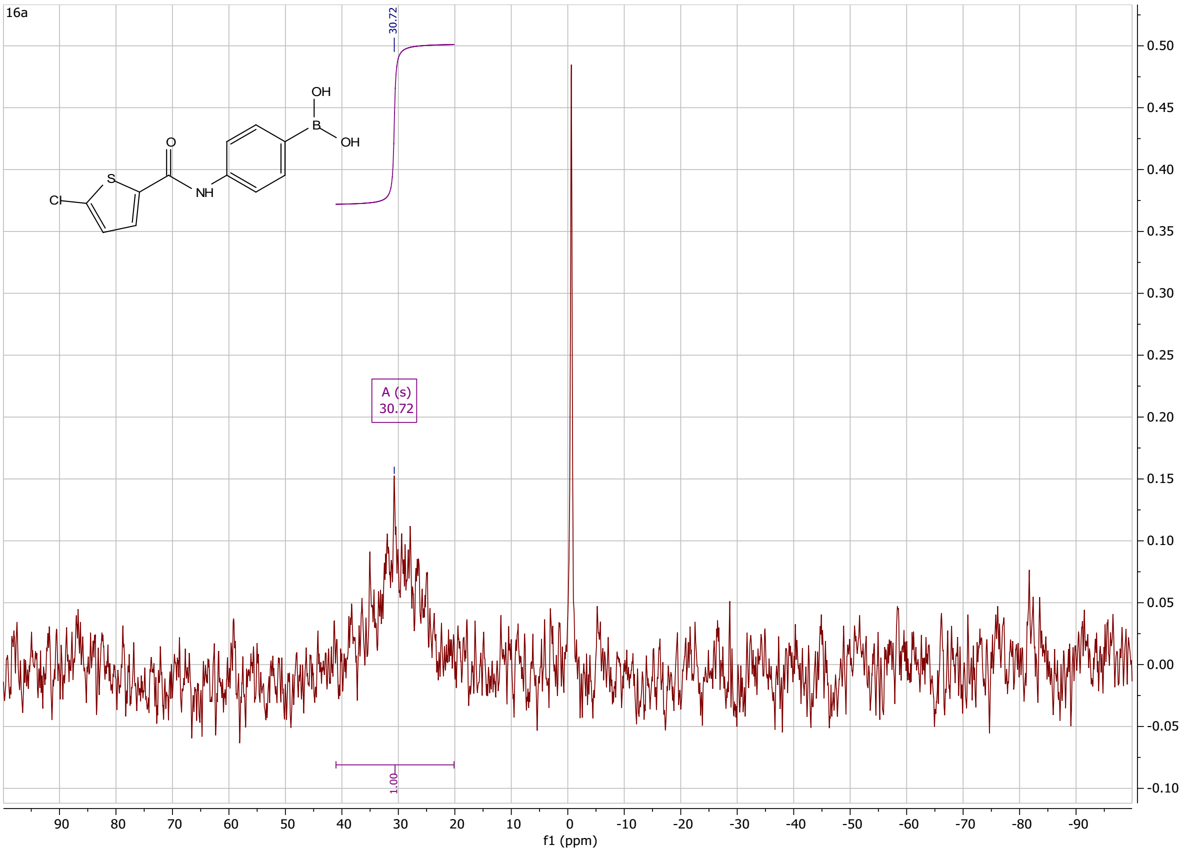

17a ^{1}H NMR spectrum

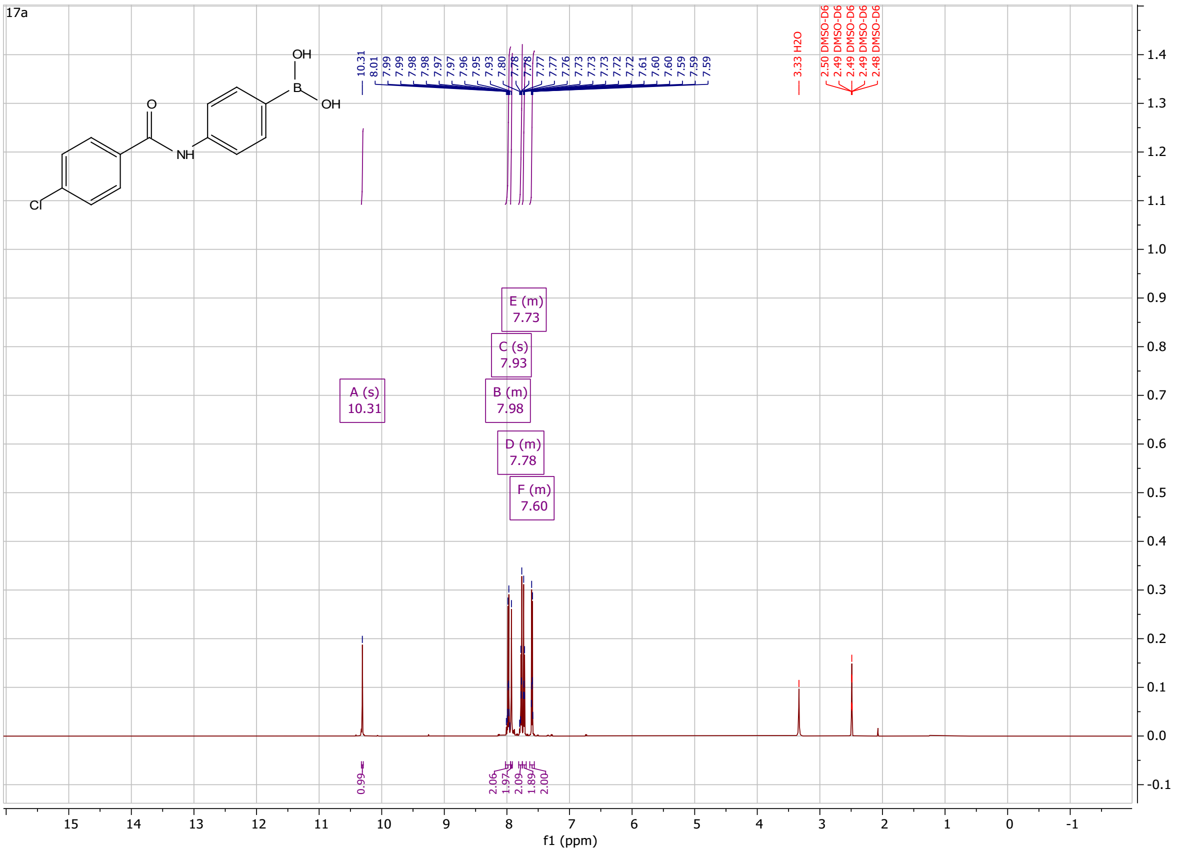

17a ^{13}C NMR spectrum

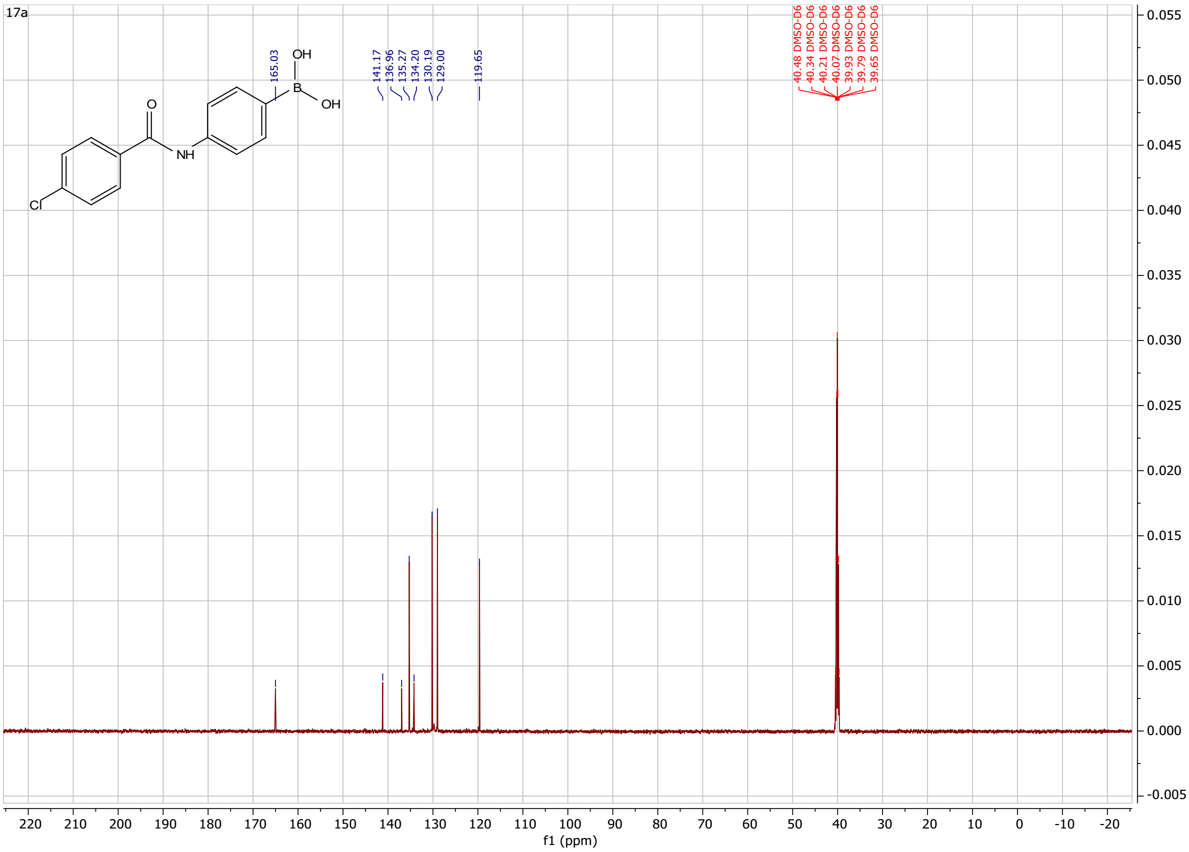

17a ^{11}B NMR spectrum

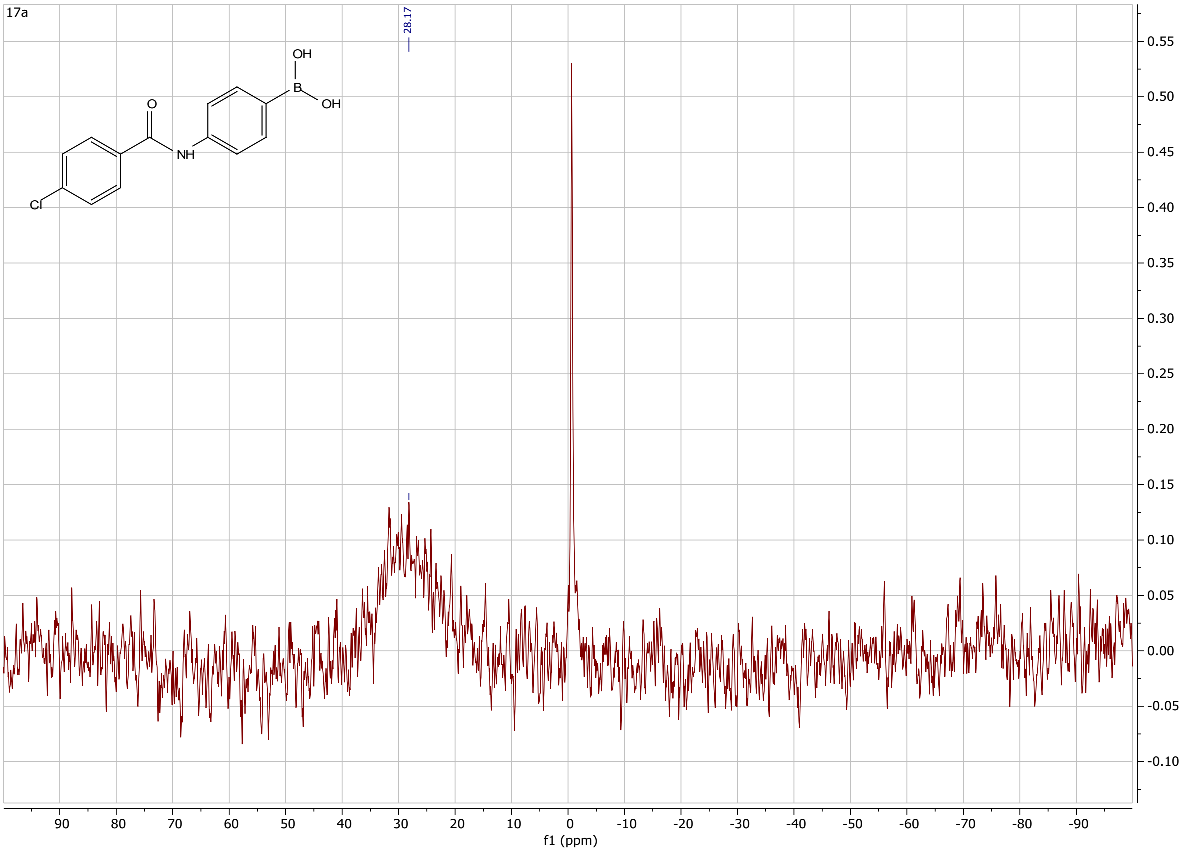

18a ^{1}H NMR spectrum

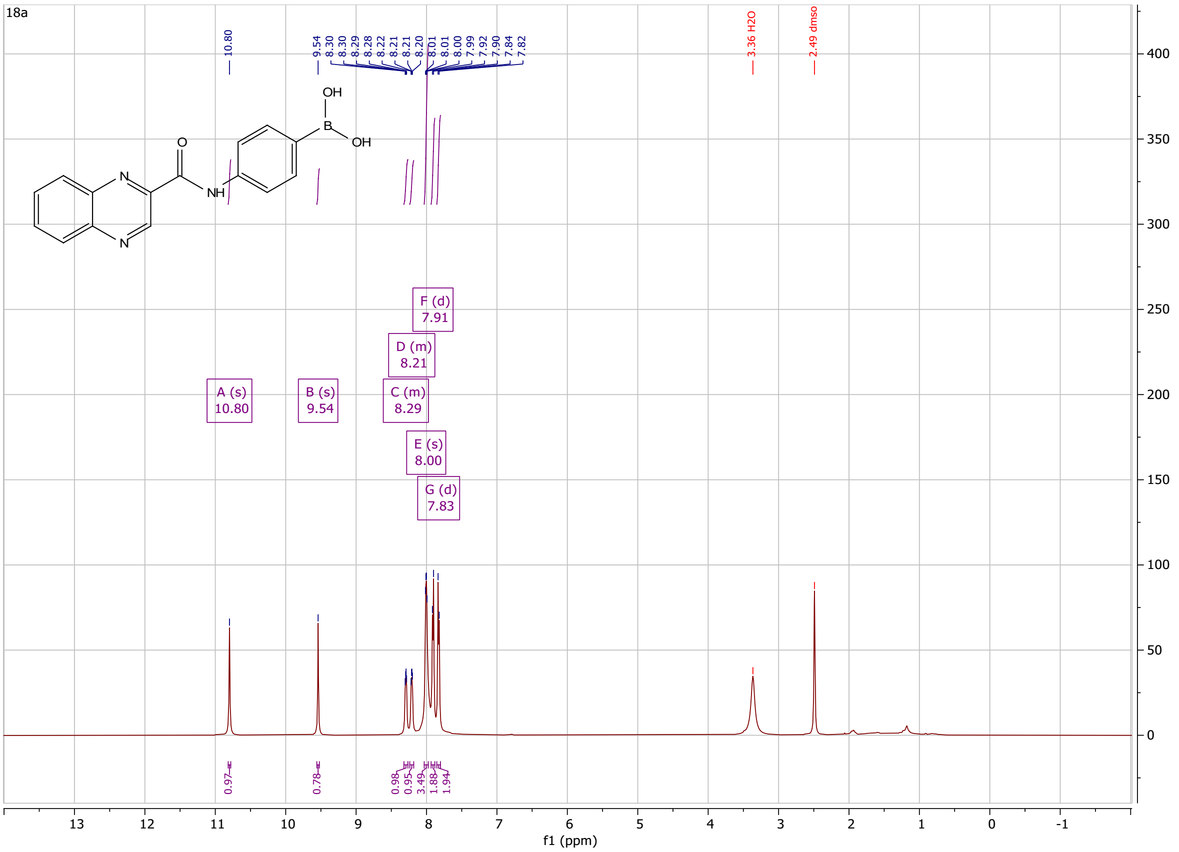

18a ^{13}C NMR spectrum

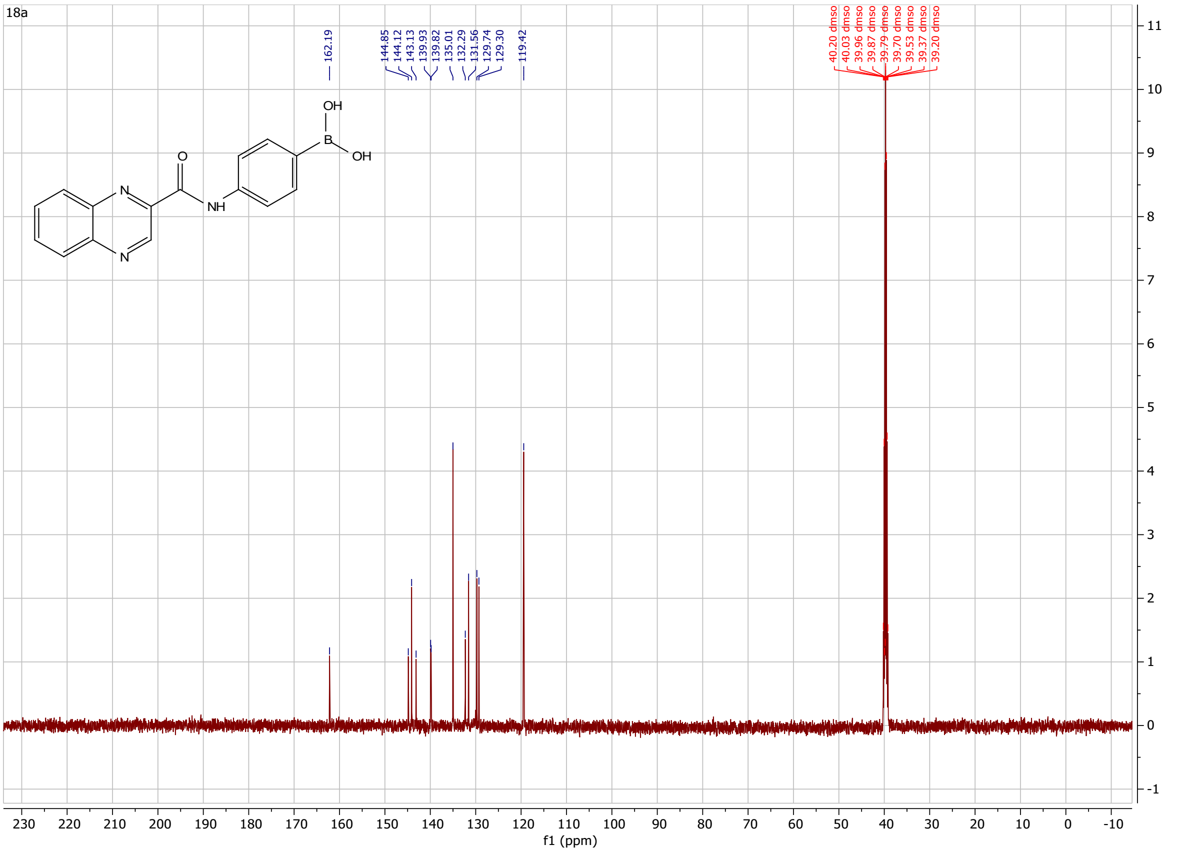

18a ^{11}B NMR spectrum

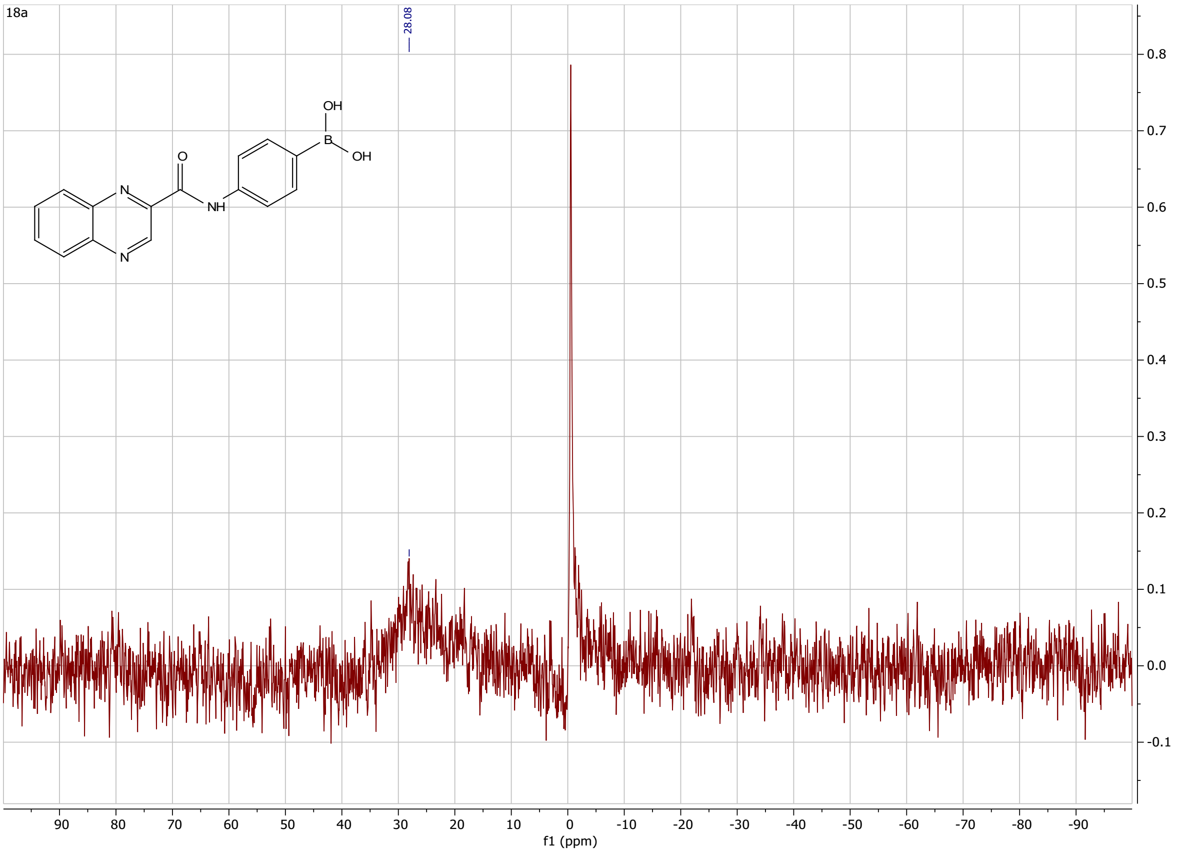

19a ^{1}H NMR spectrum

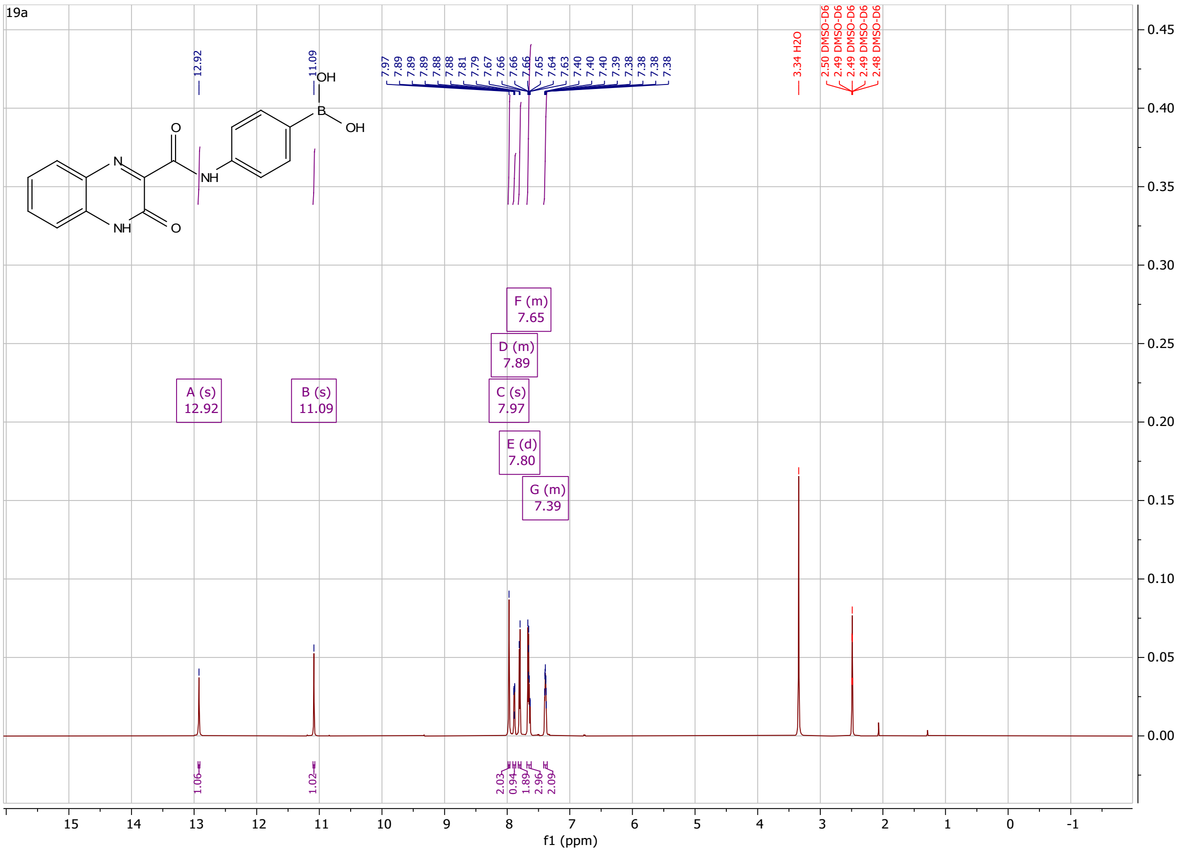

19a ^{13}C NMR spectrum

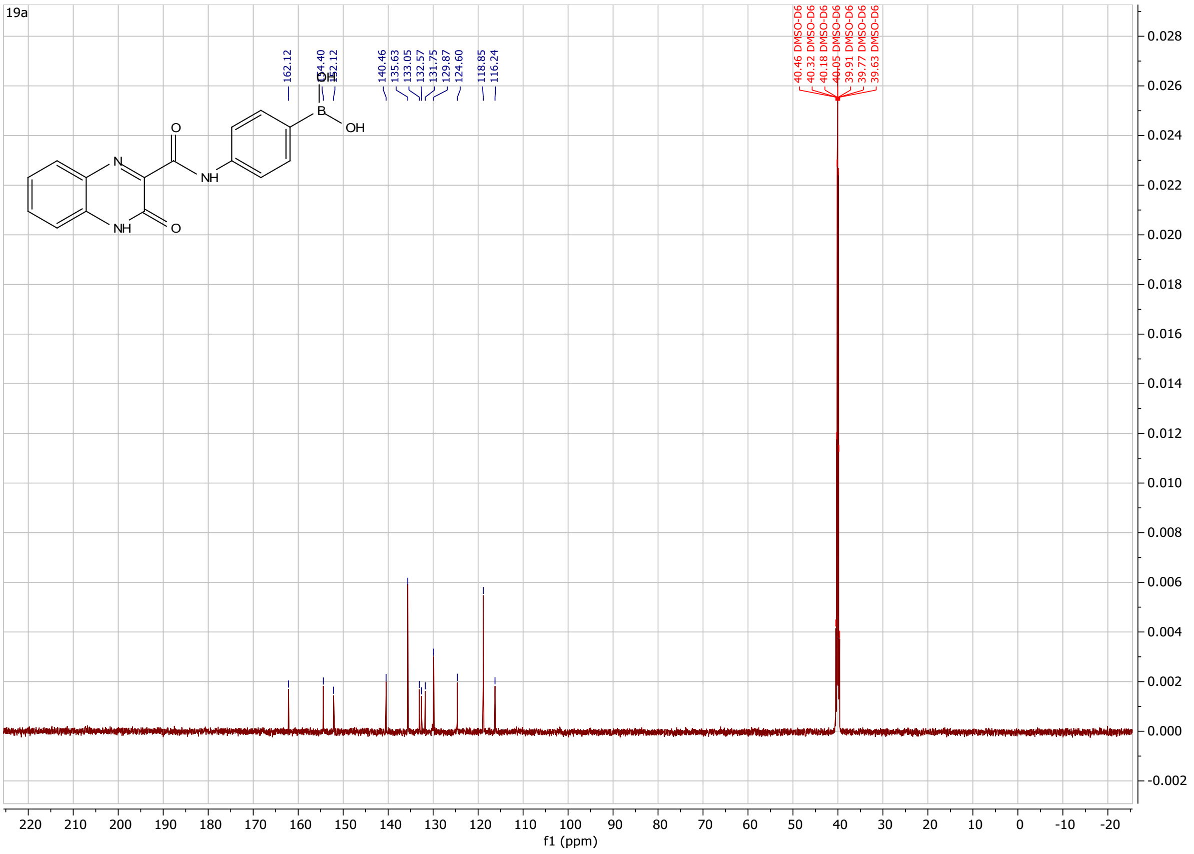

19 ^{11}B NMR spectrum

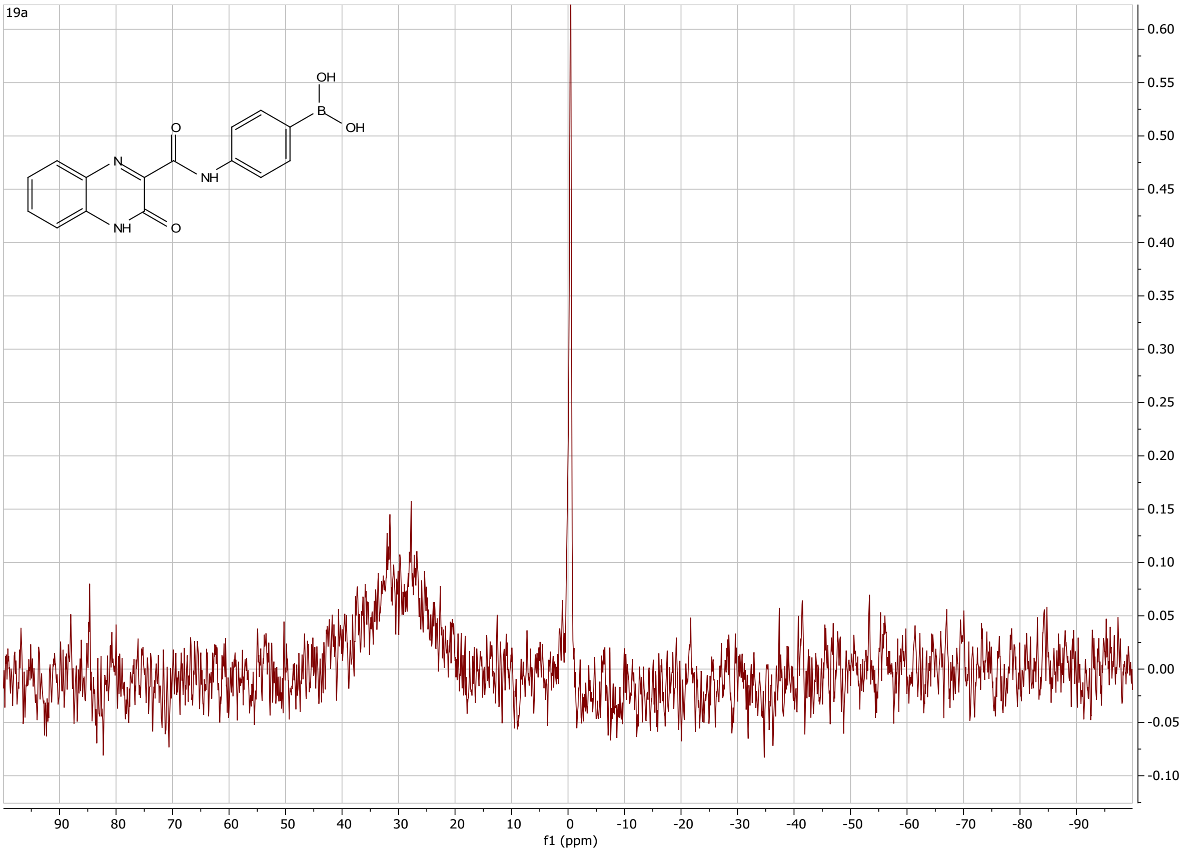

20a^{1}H NMR spectrum

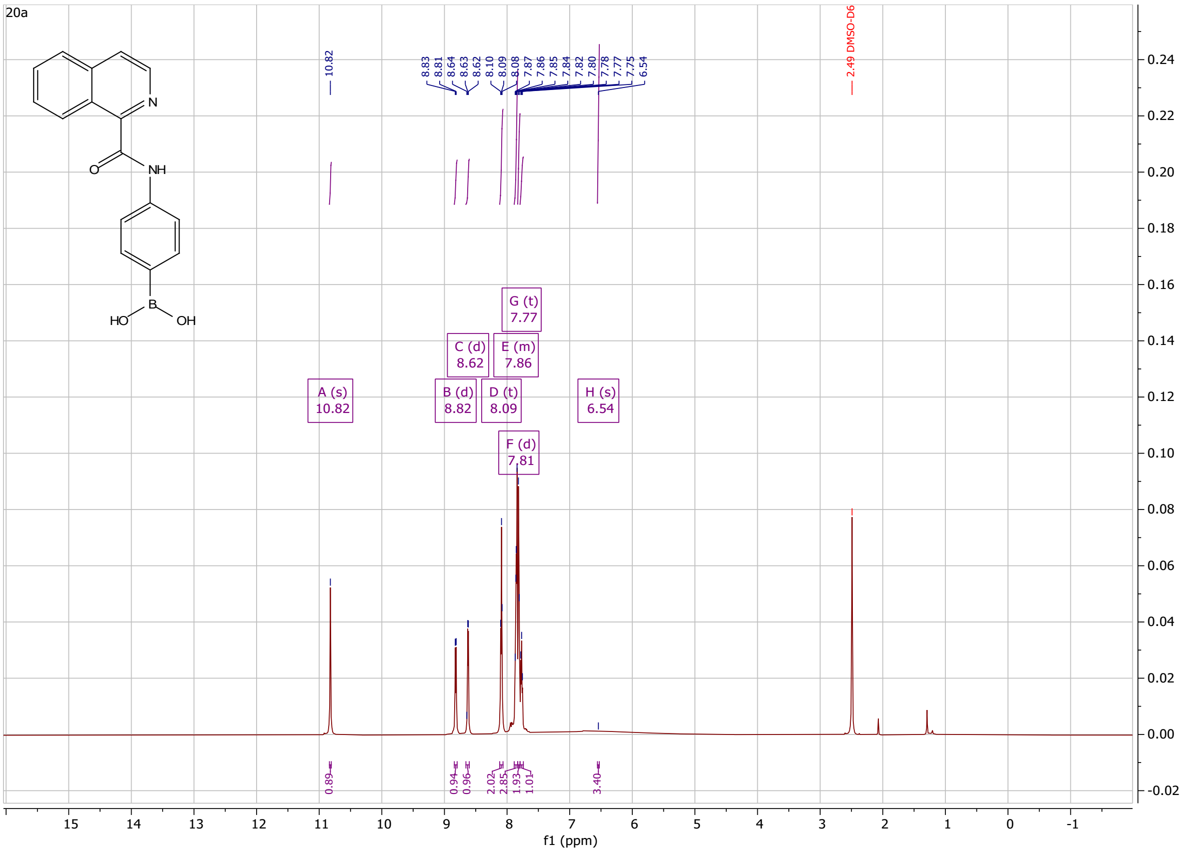

20a ^{13}C NMR spectrum

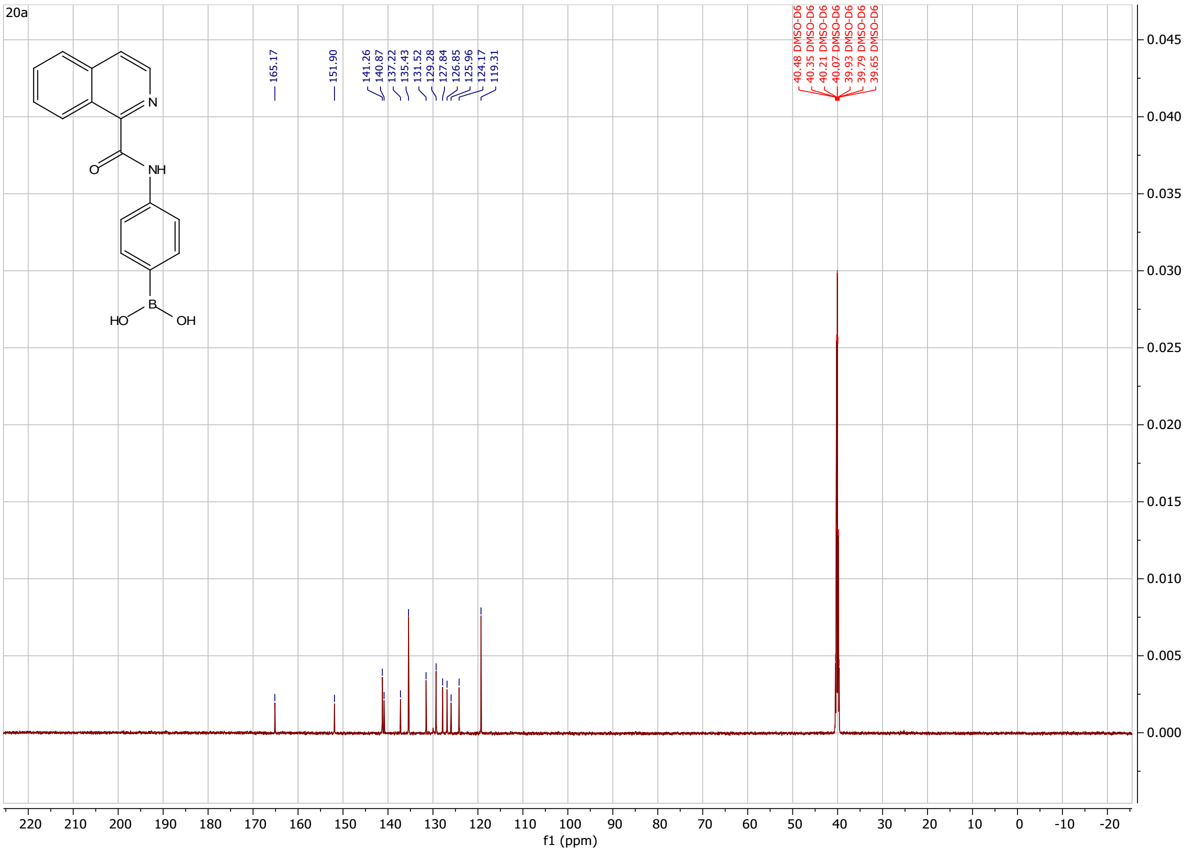

20a ^{11}B NMR spectrum

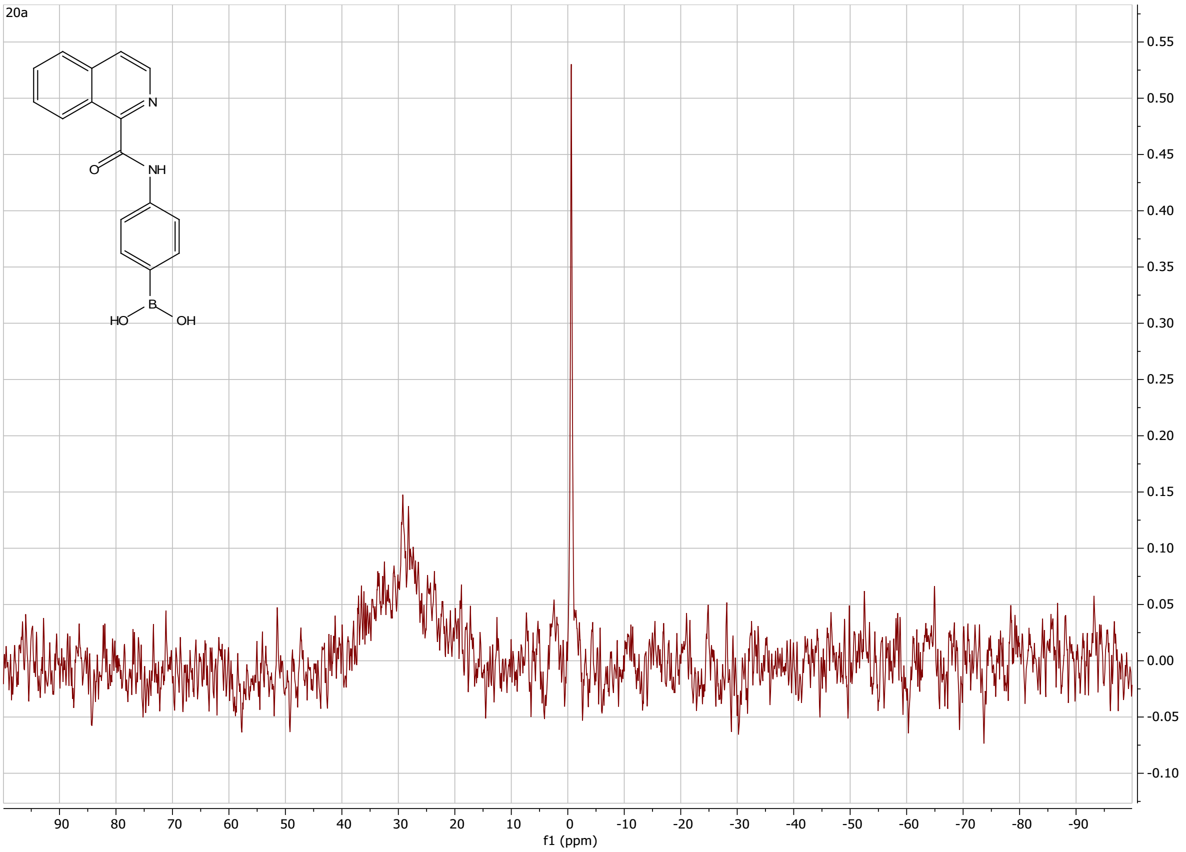

1b ^{1}H NMR spectrum

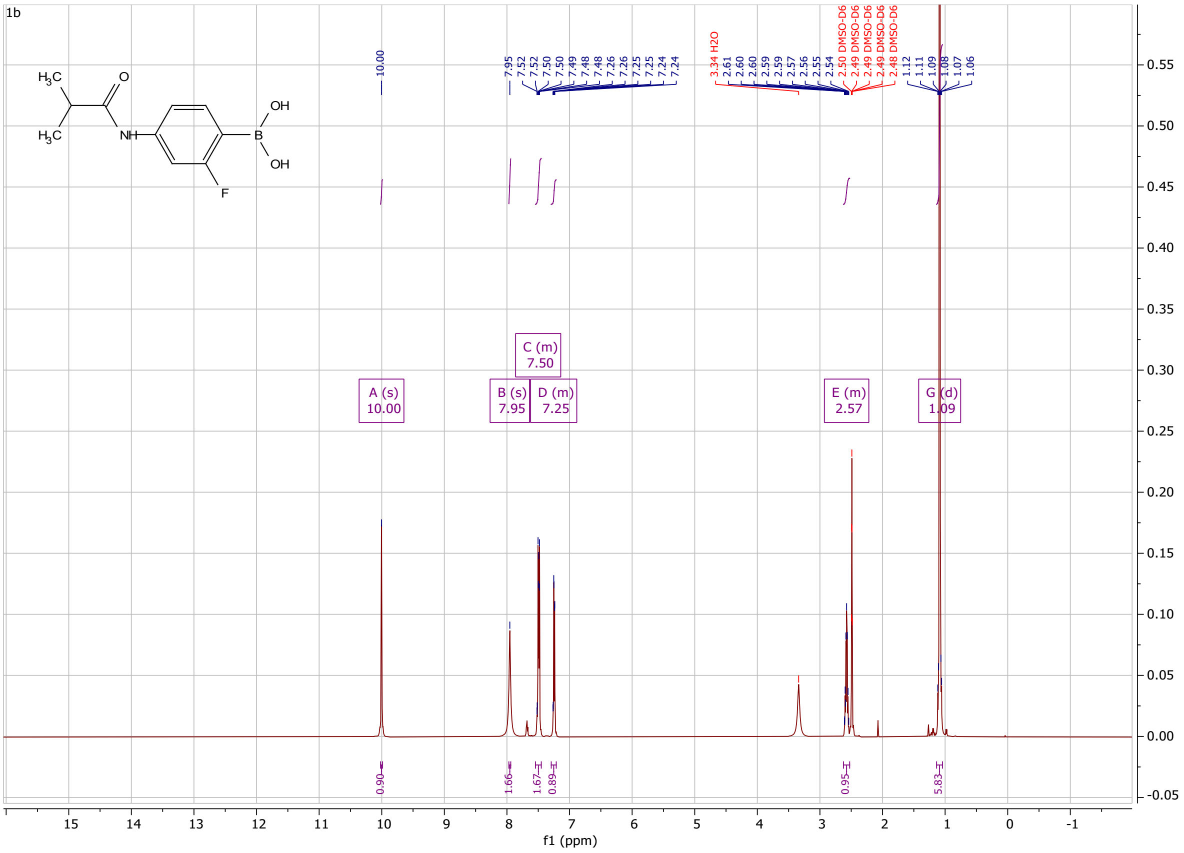

1b ^{13}C NMR spectrum

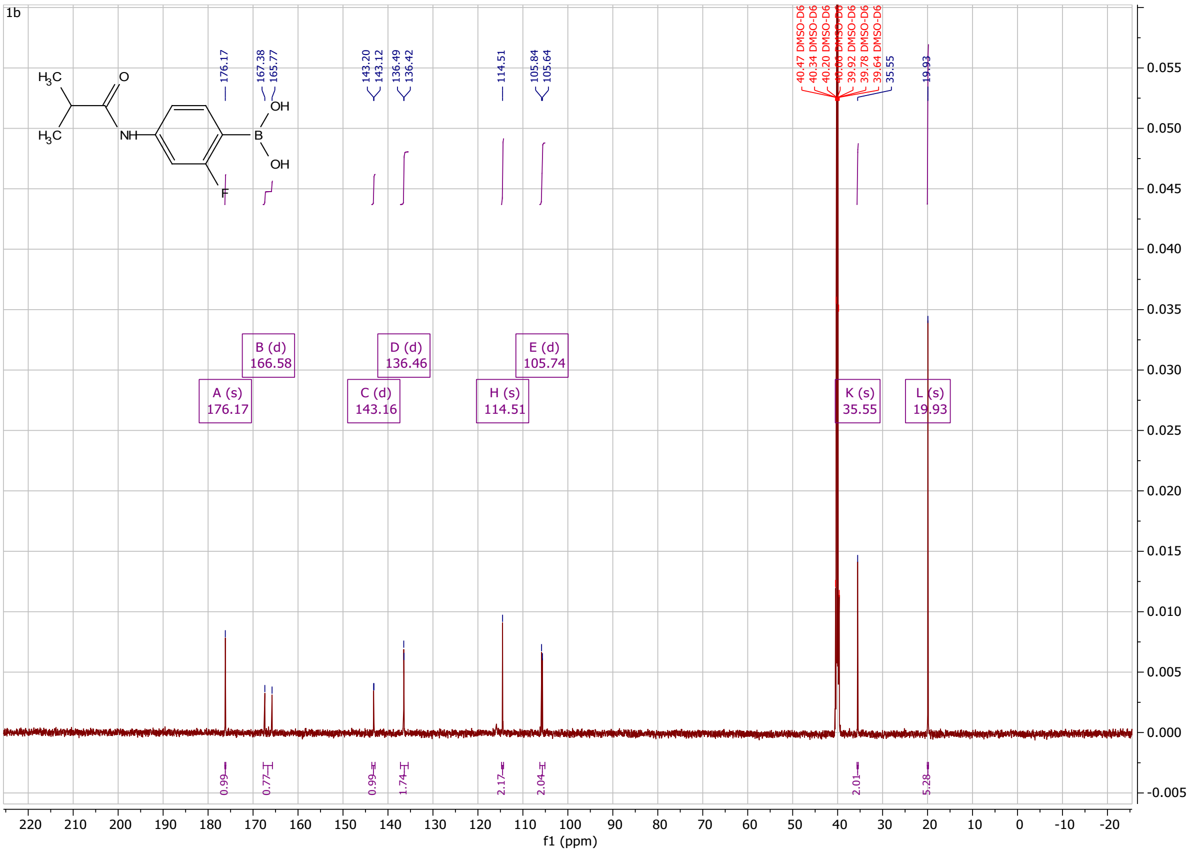

1b ^{11}B NMR spectrum

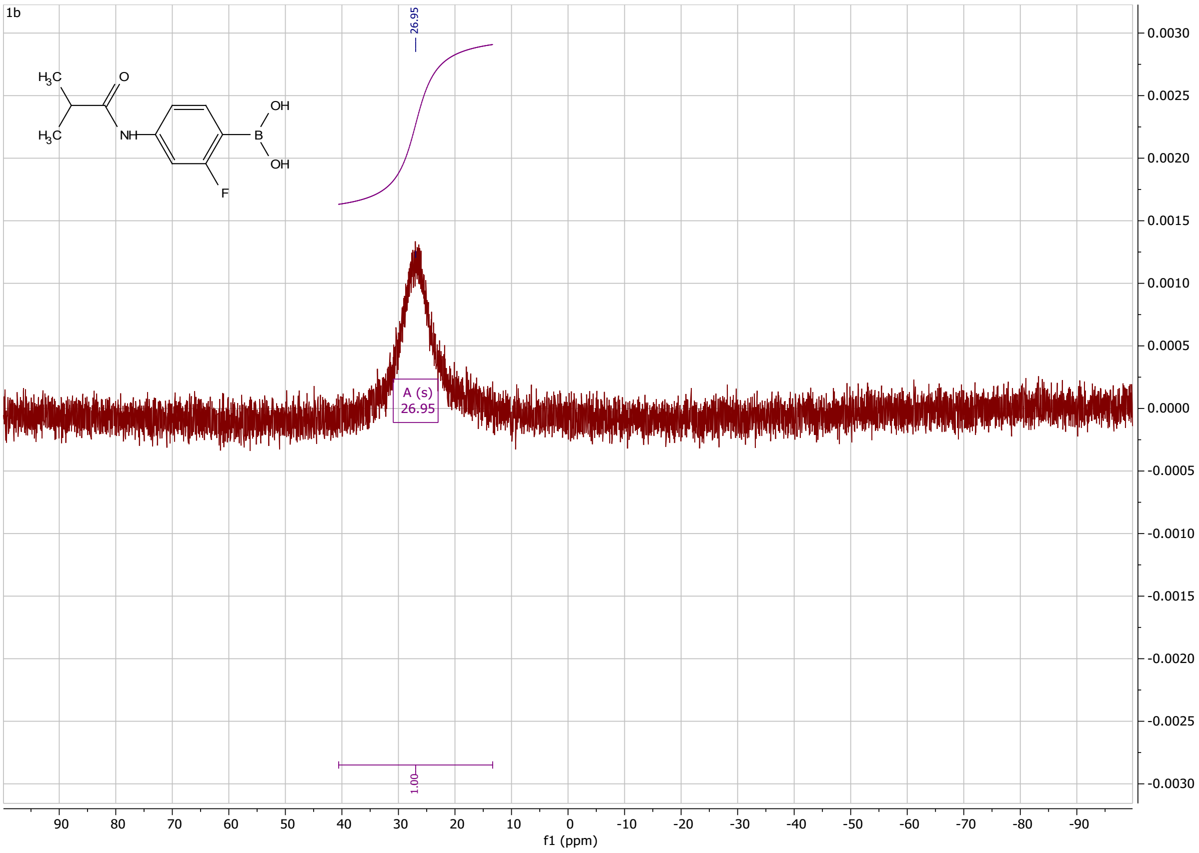

2b^{1}H NMR spectrum

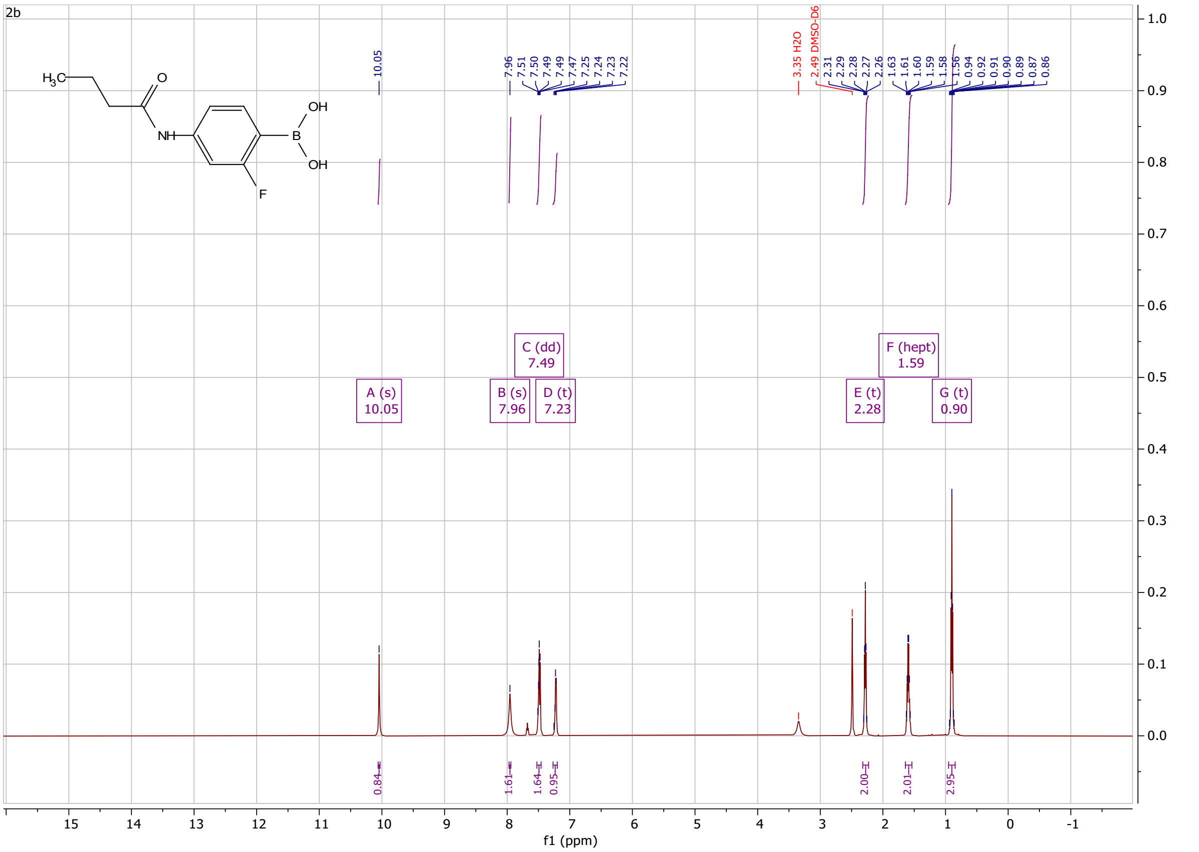

2b ^{13}C NMR spectrum

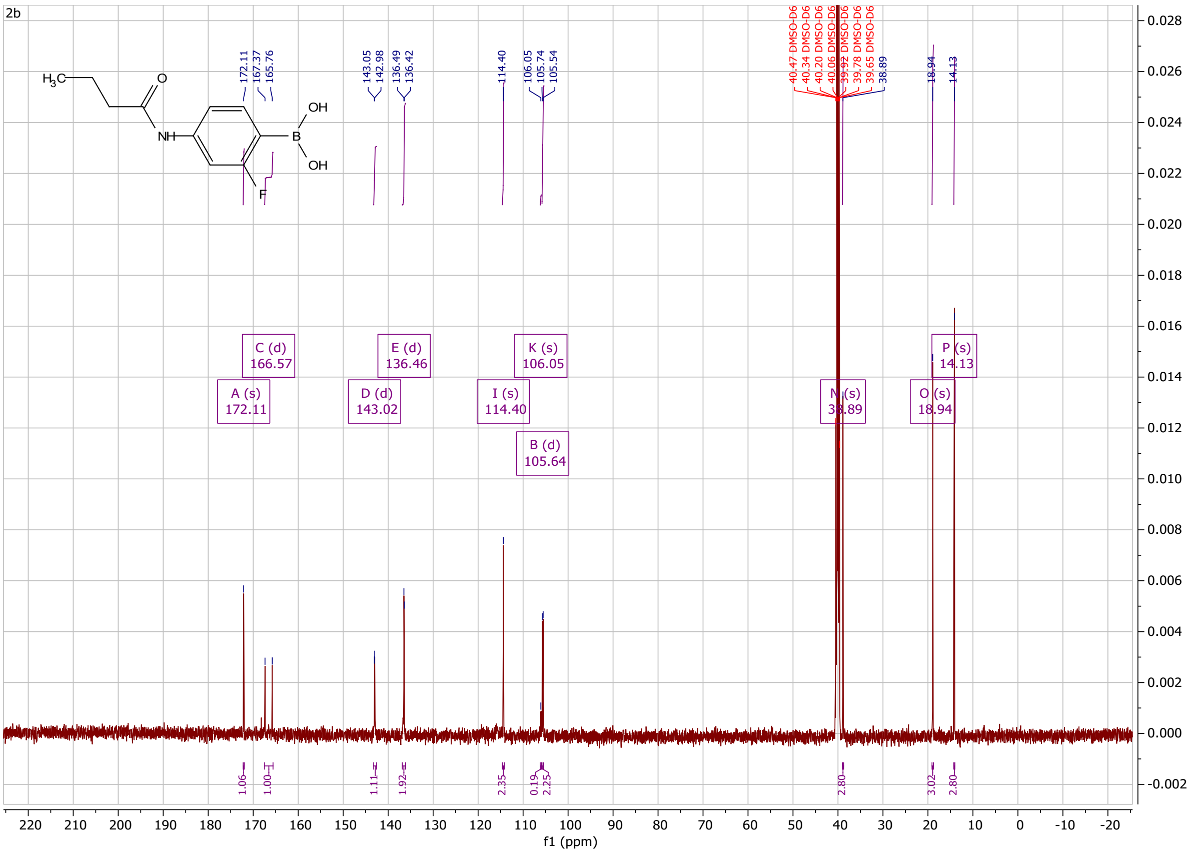

2b ^{11}B NMR spectrum

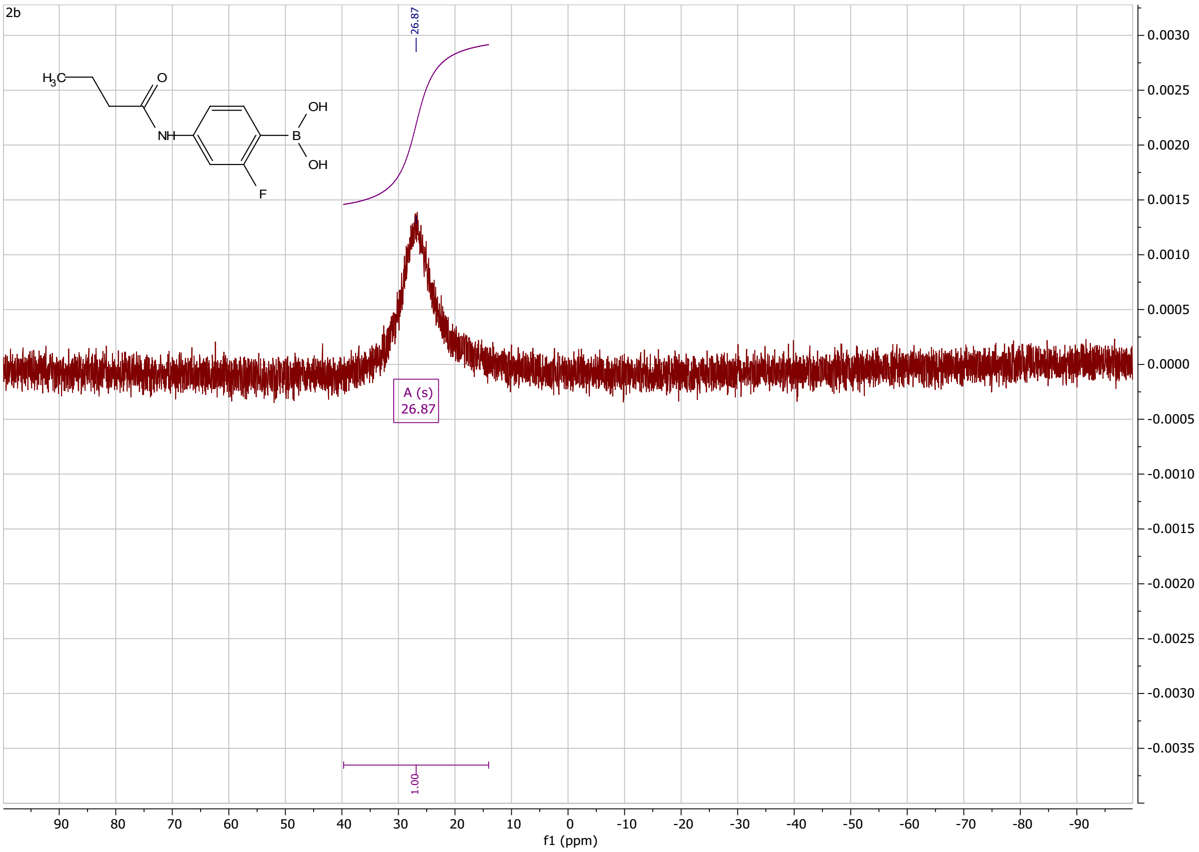

3b ^{1}H NMR spectrum

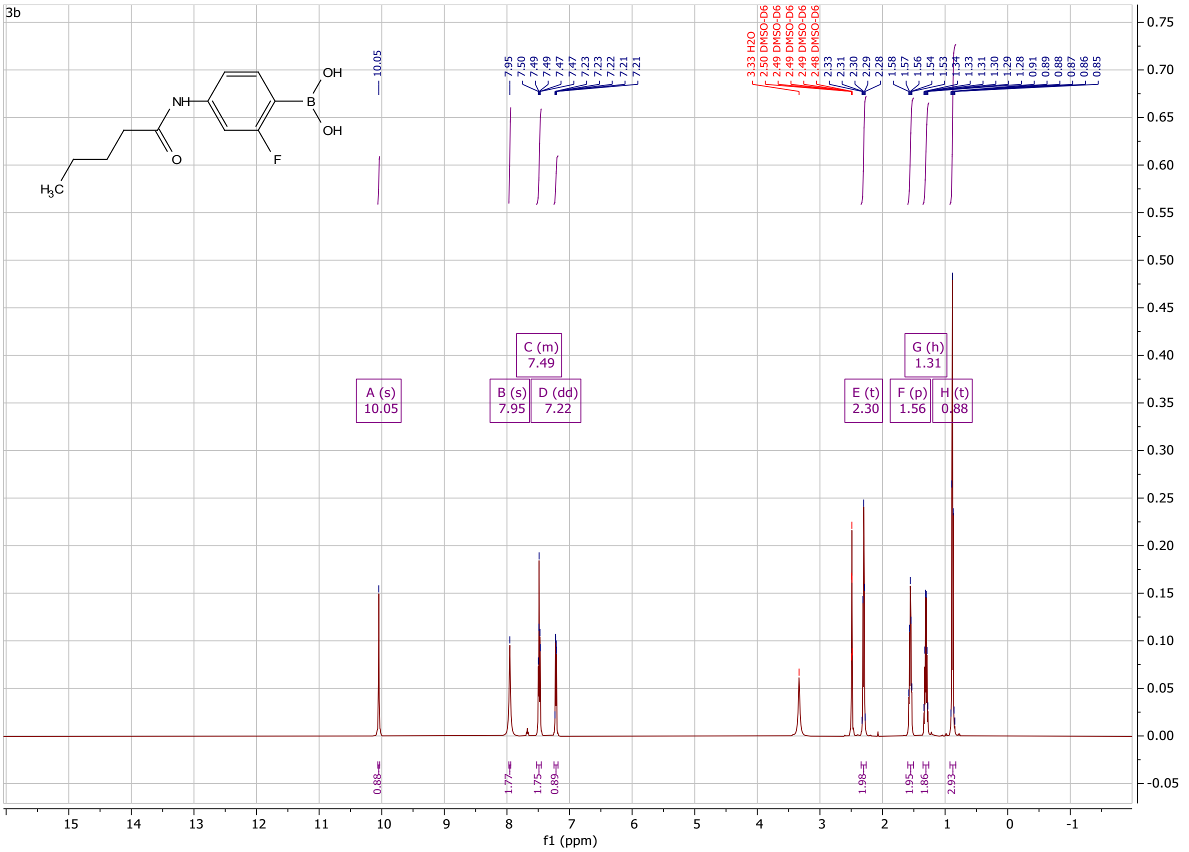

3b ^{13}C NMR spectrum

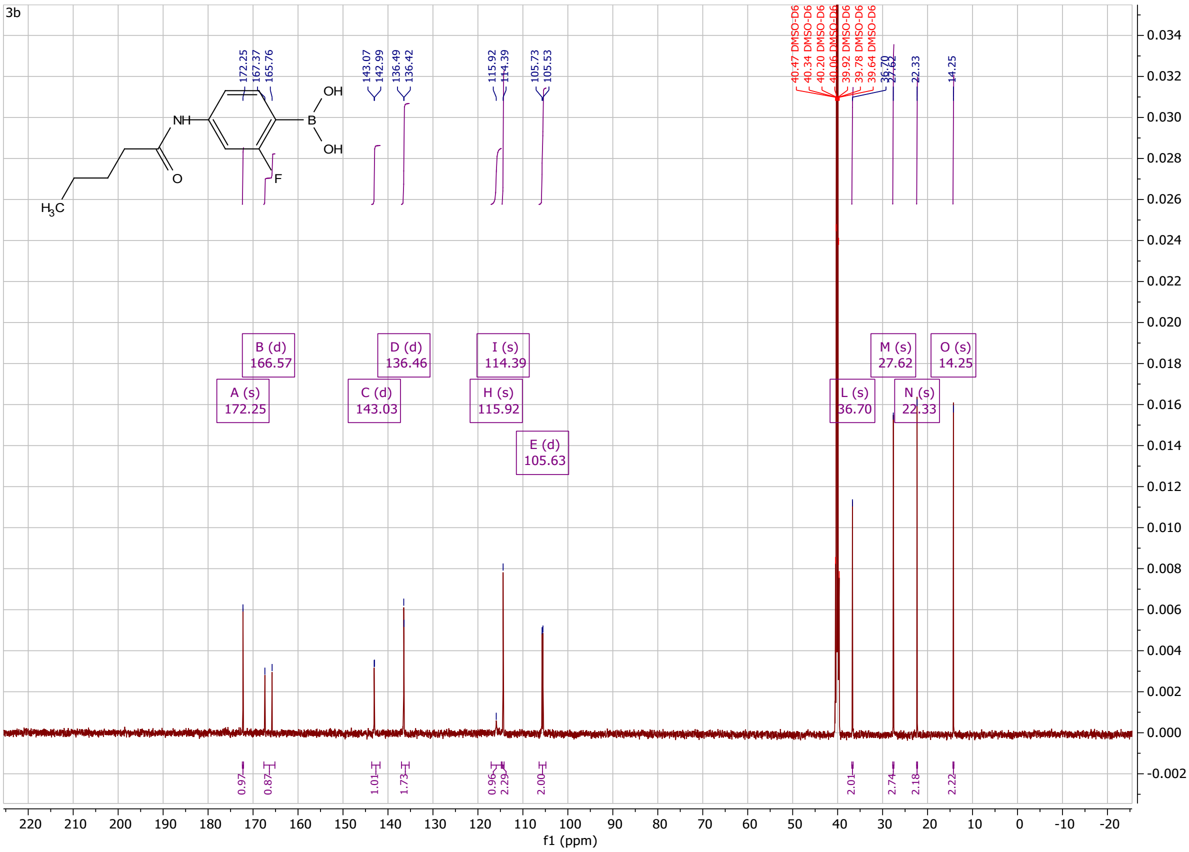

3b ^{11}B NMR spectrum

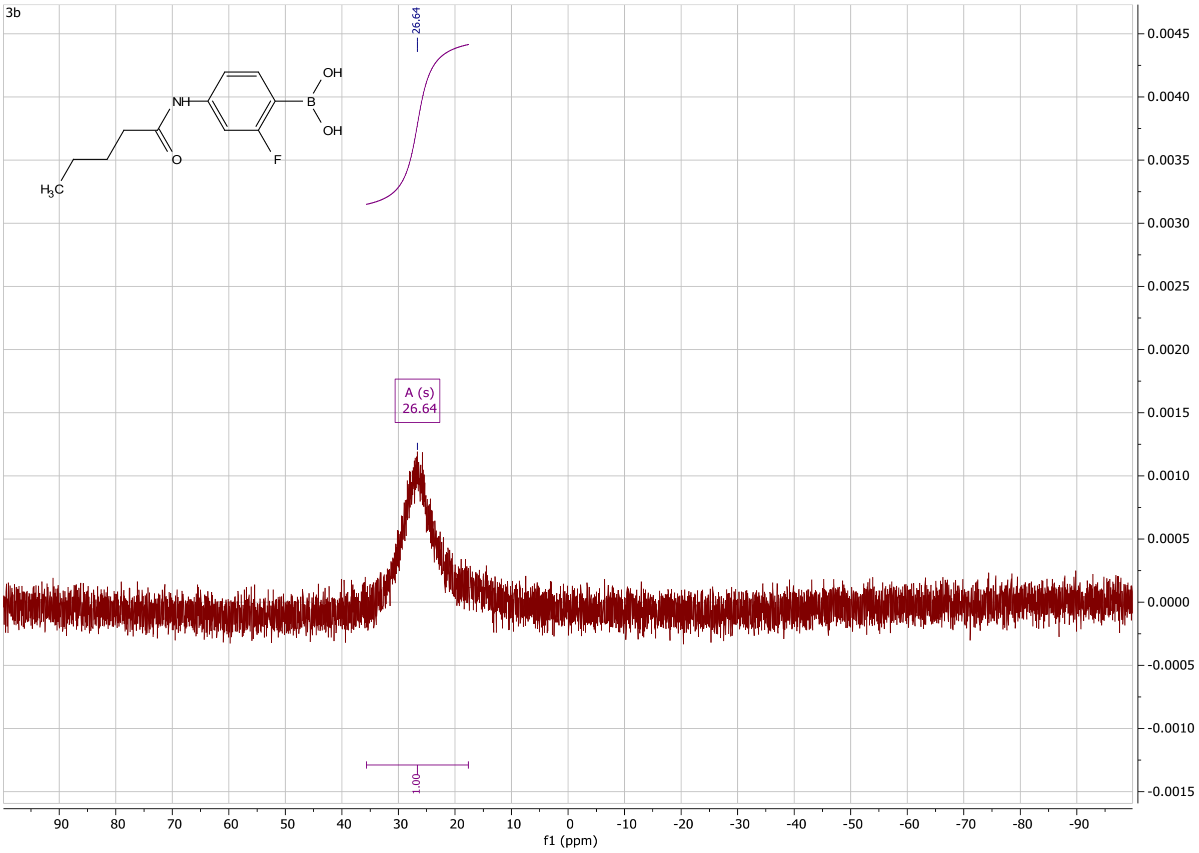

4b ^{1}H NMR spectrum

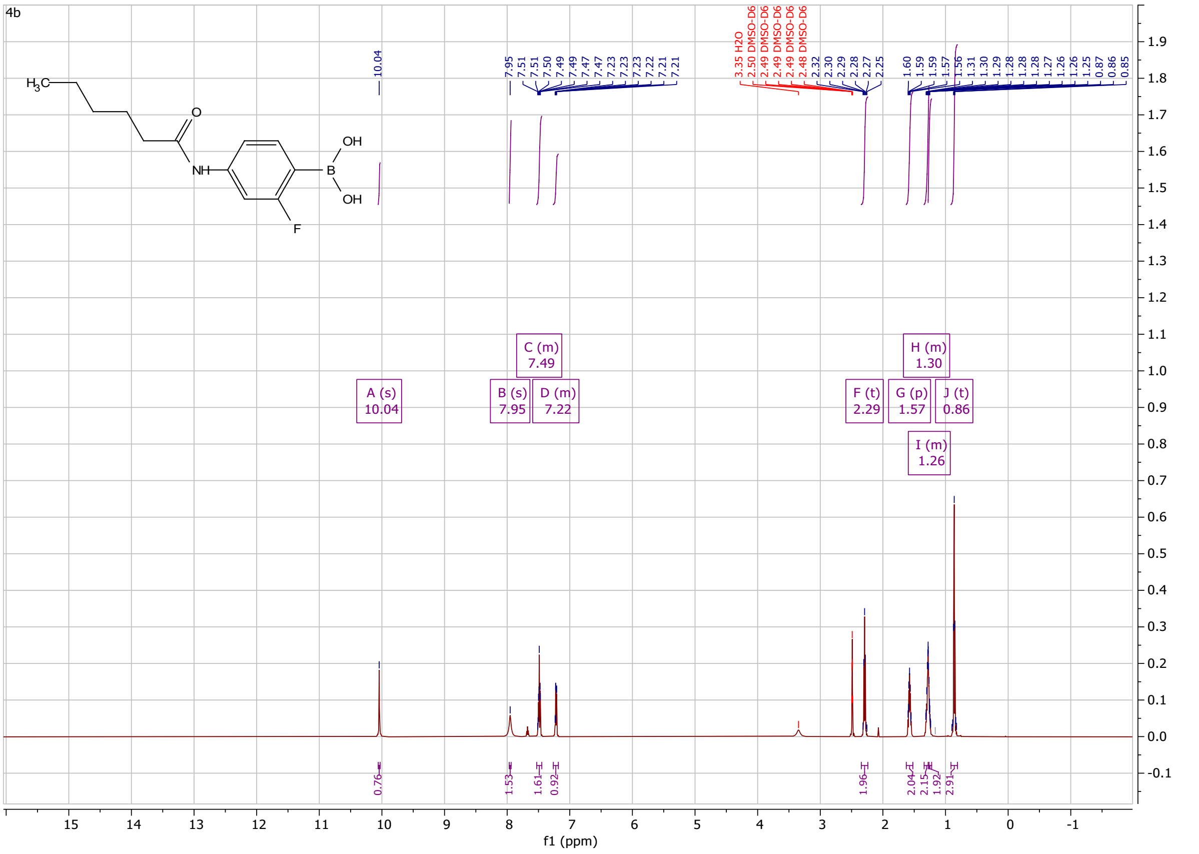

4b ^{13}C NMR spectrum

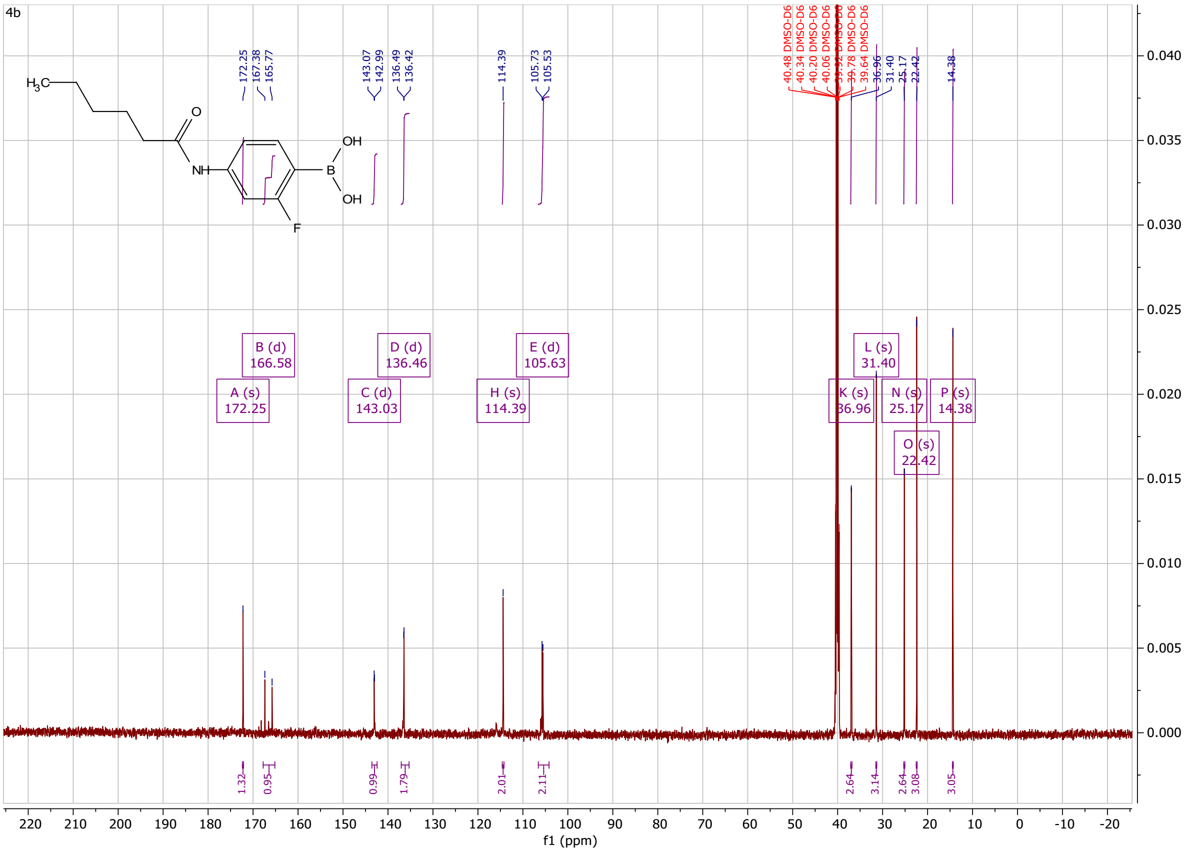

4b ^{11}B NMR spectrum

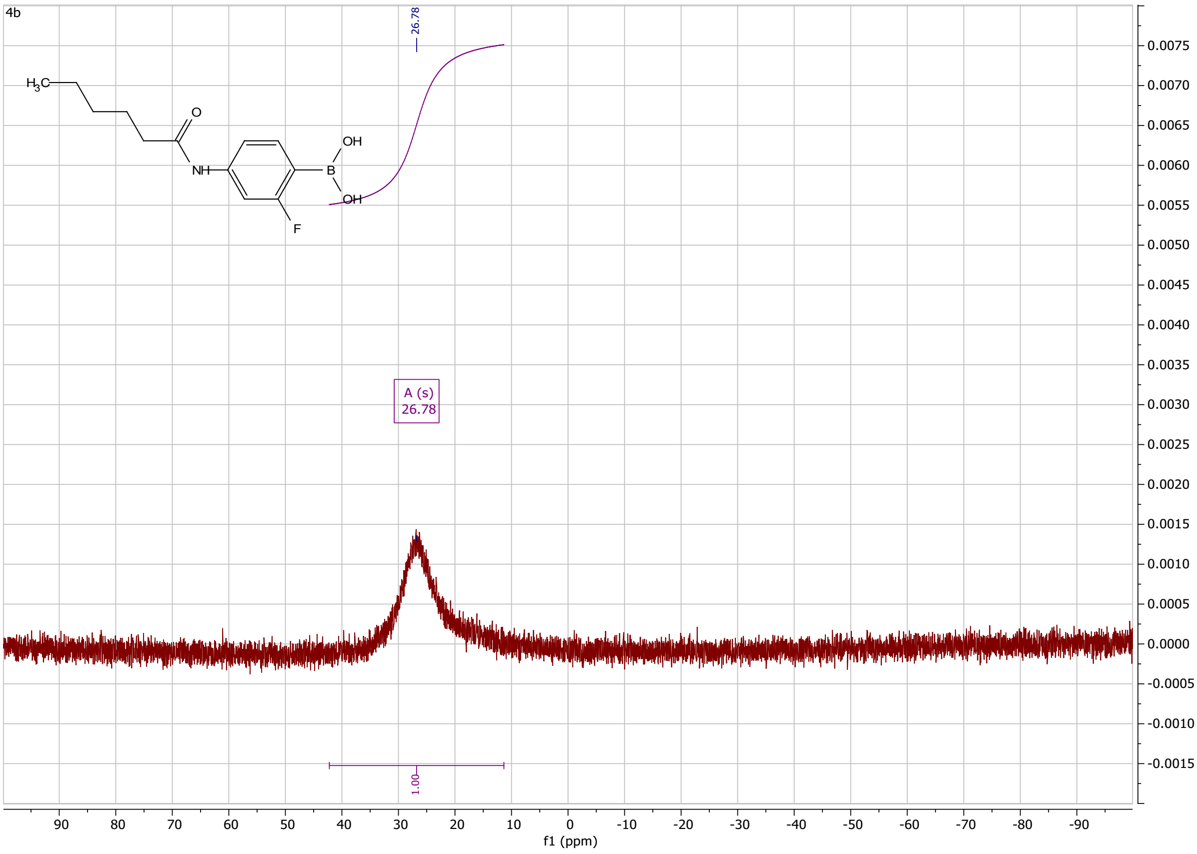

5b ^{1}H NMR spectrum

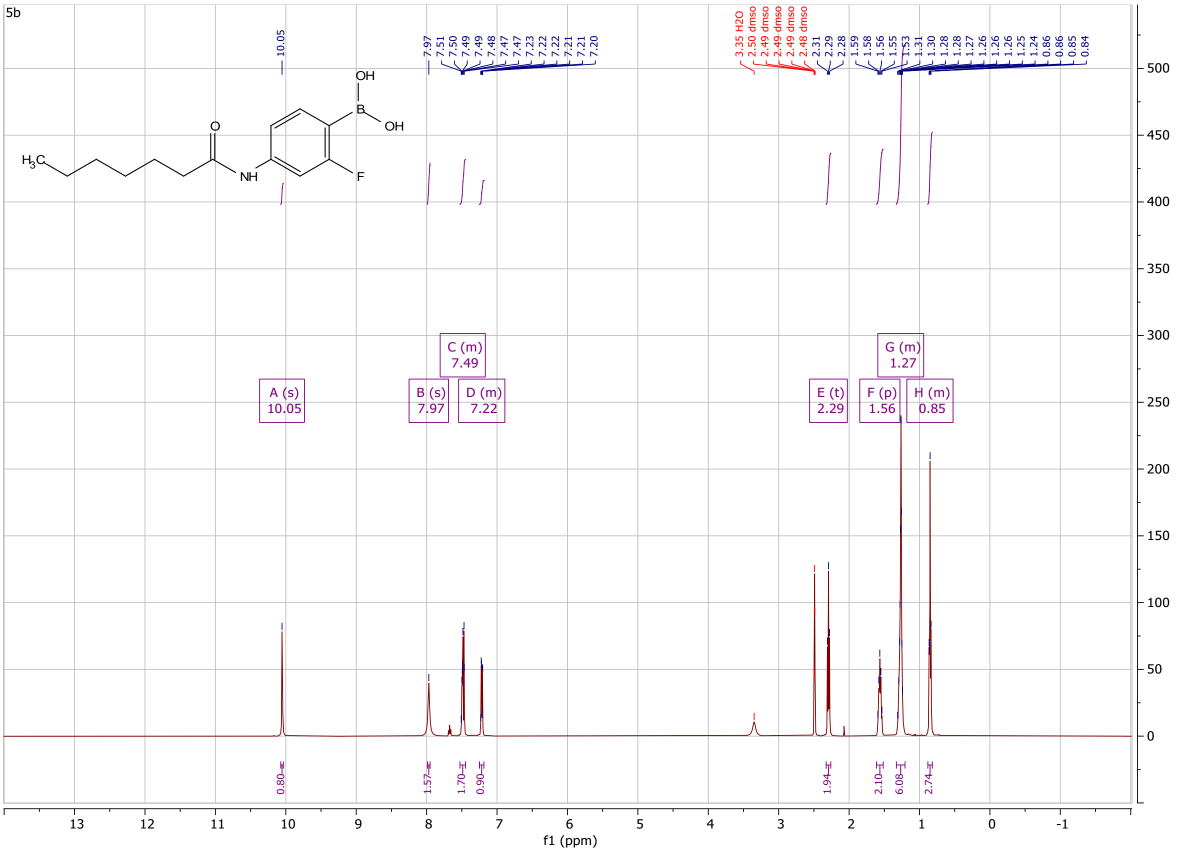

5b ^{13}C NMR spectrum

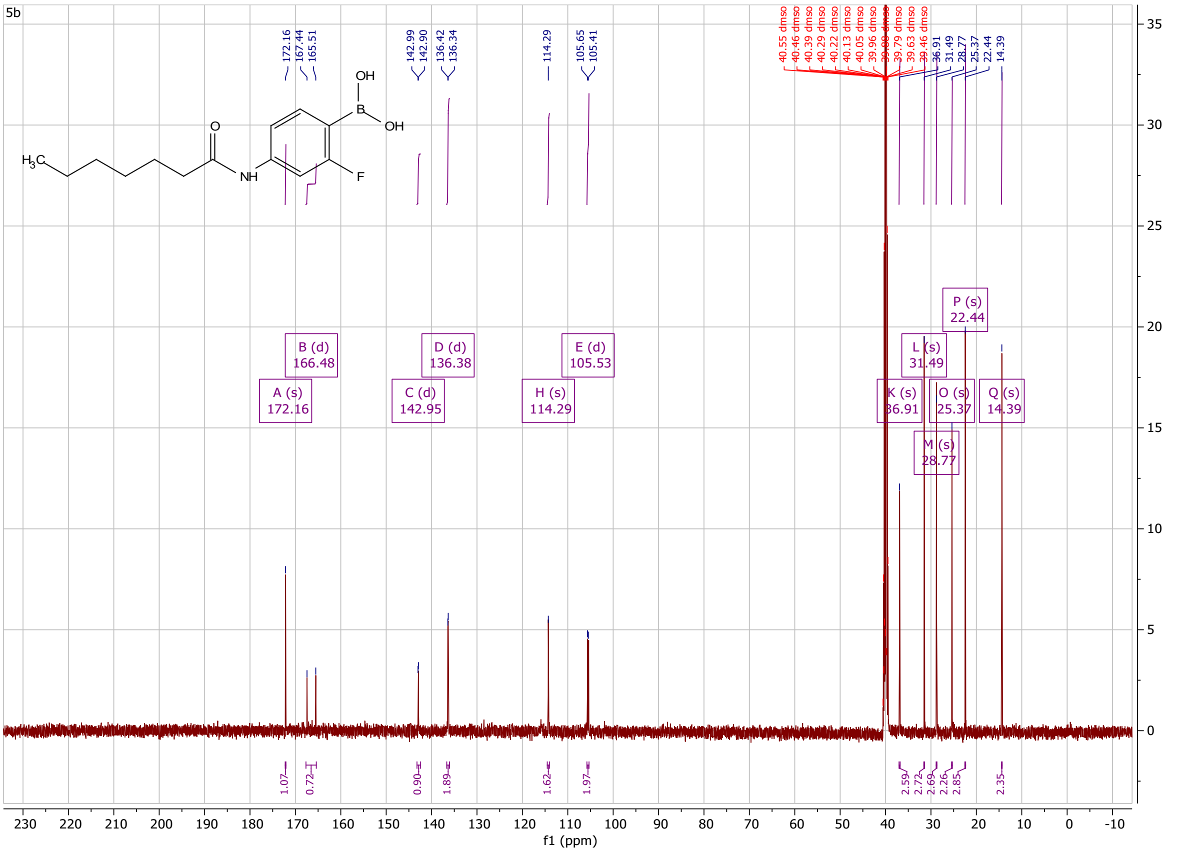

5b ^{11}B NMR spectrum

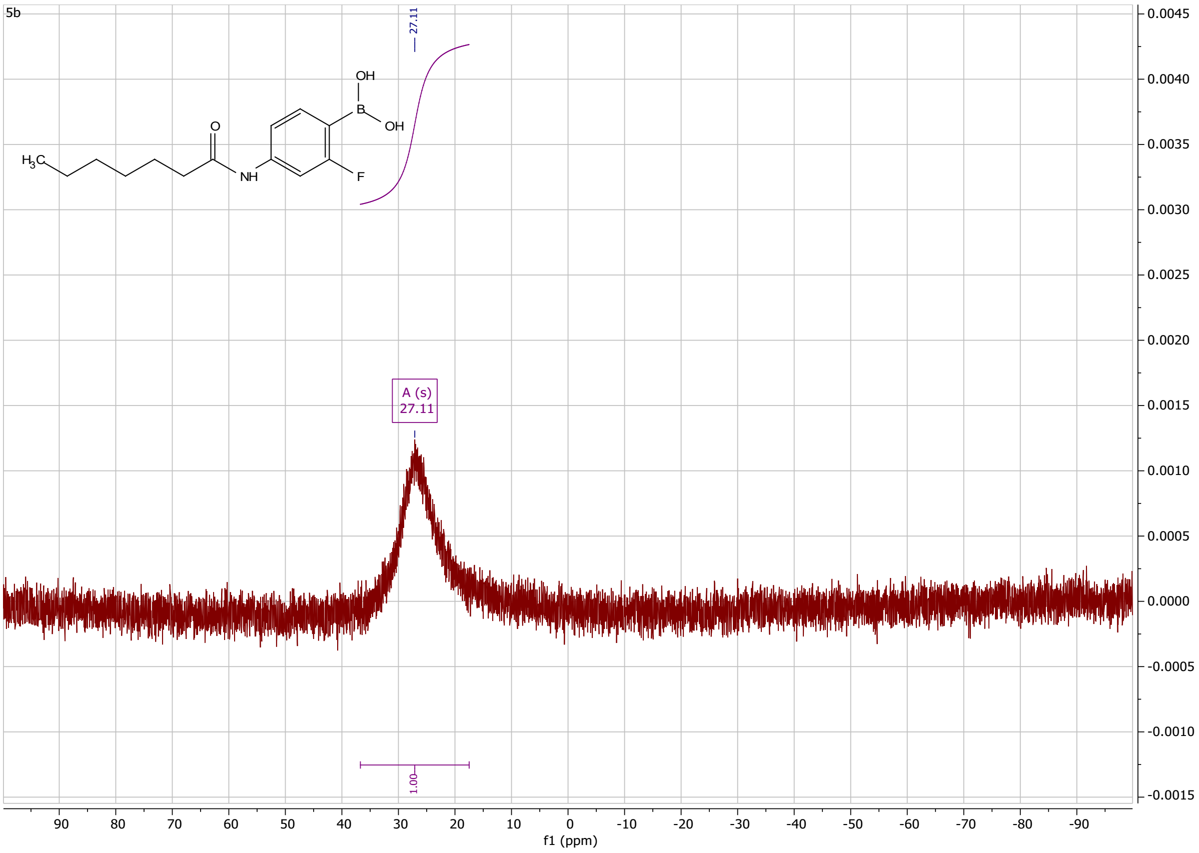

6b ^{1}H NMR spectrum

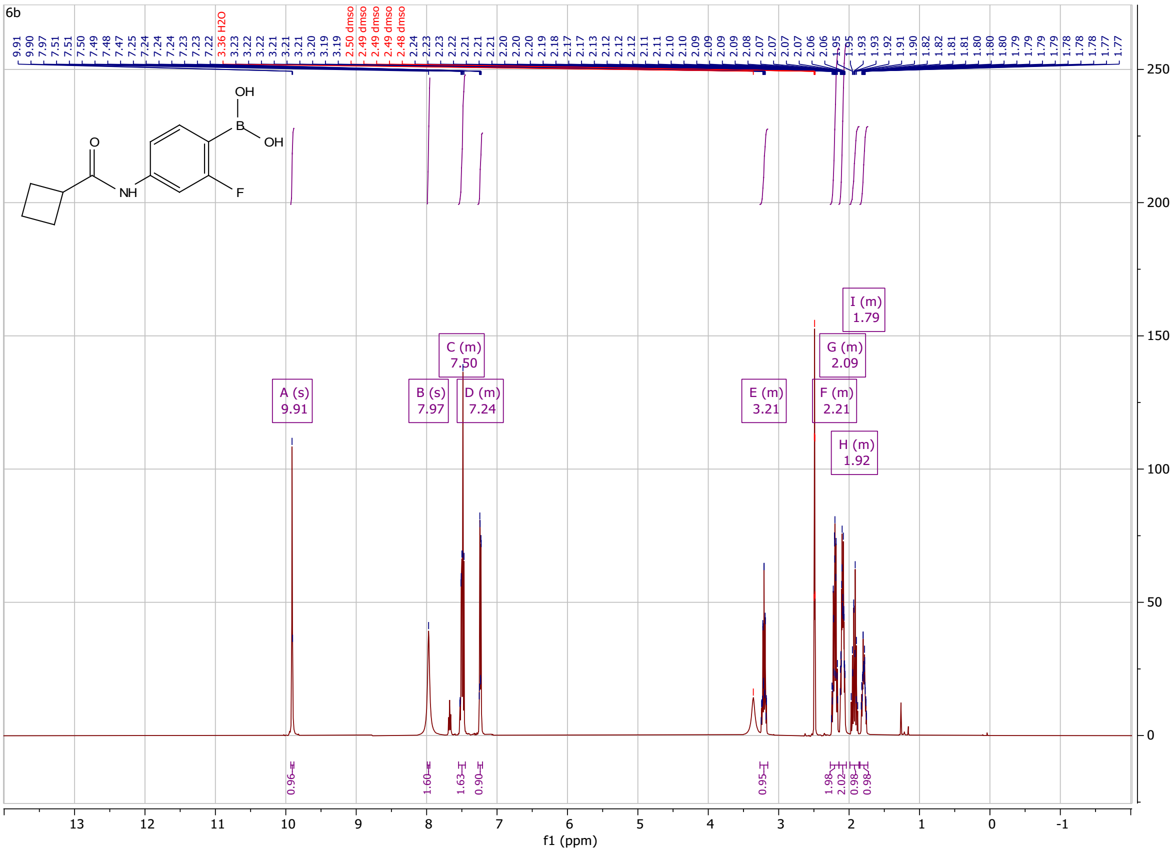

6b ^{13}C NMR spectrum

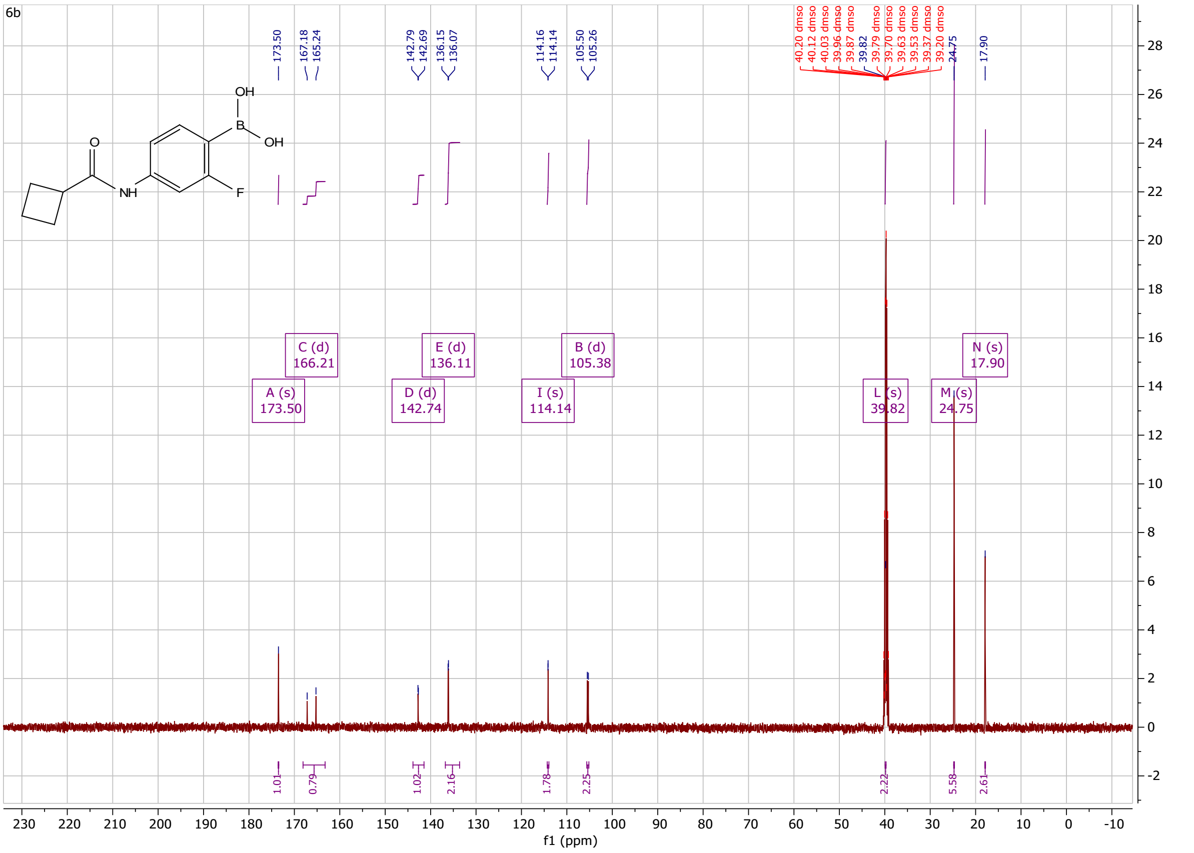

6b ^{11}B NMR spectrum

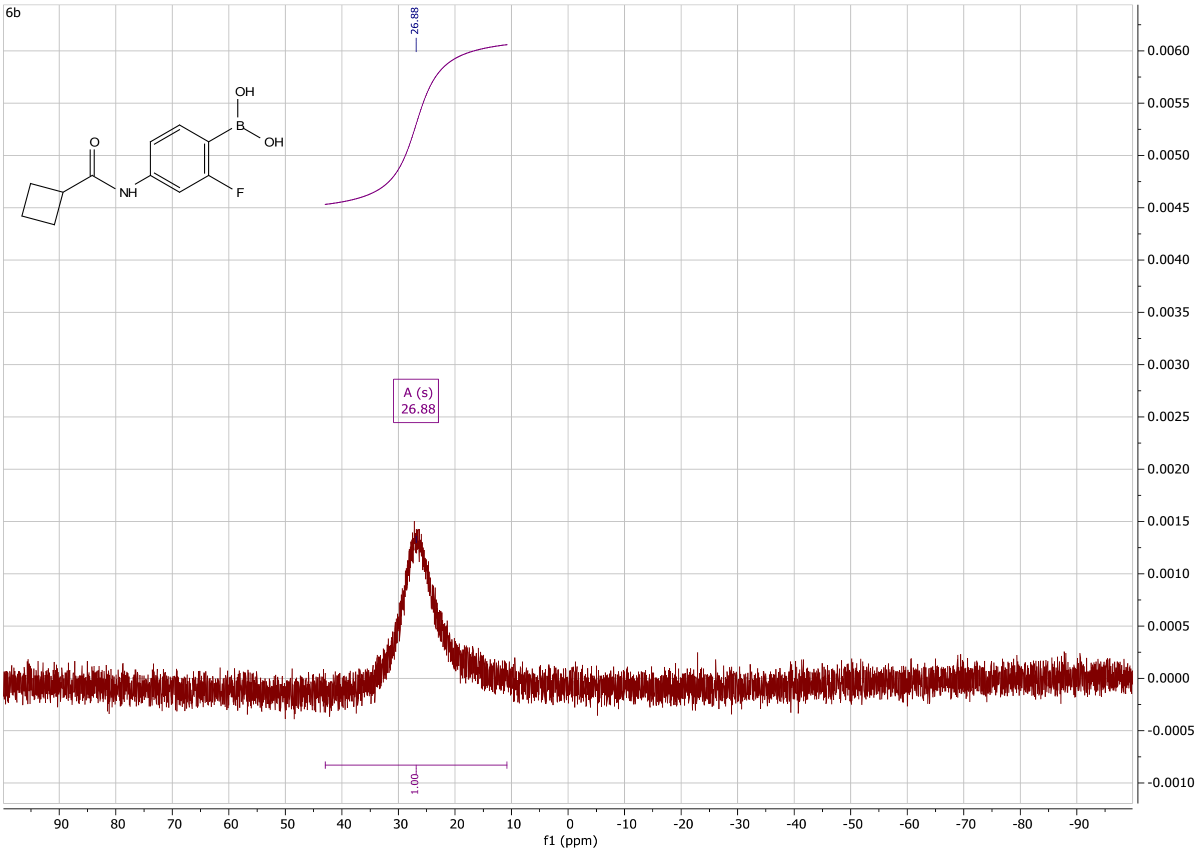

7b ^{1}H NMR spectrum

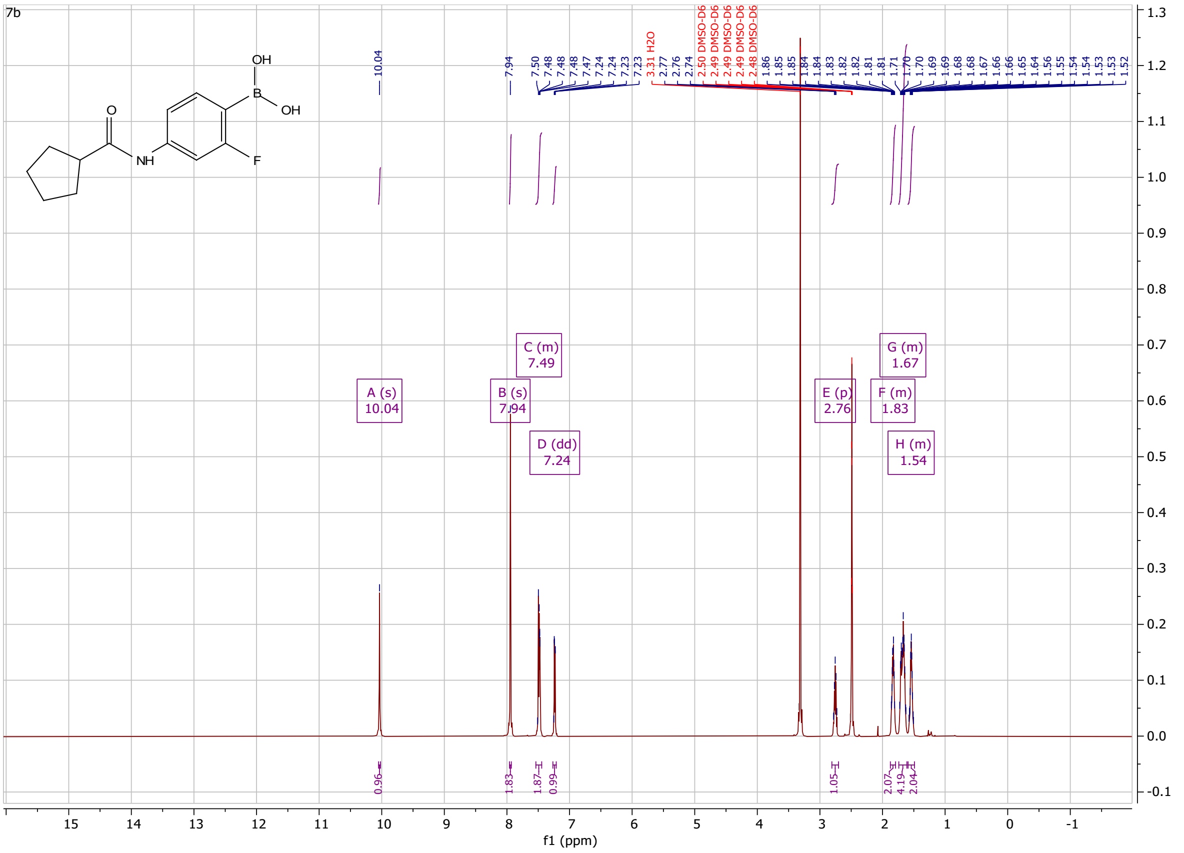

7b ^{13}C NMR spectrum

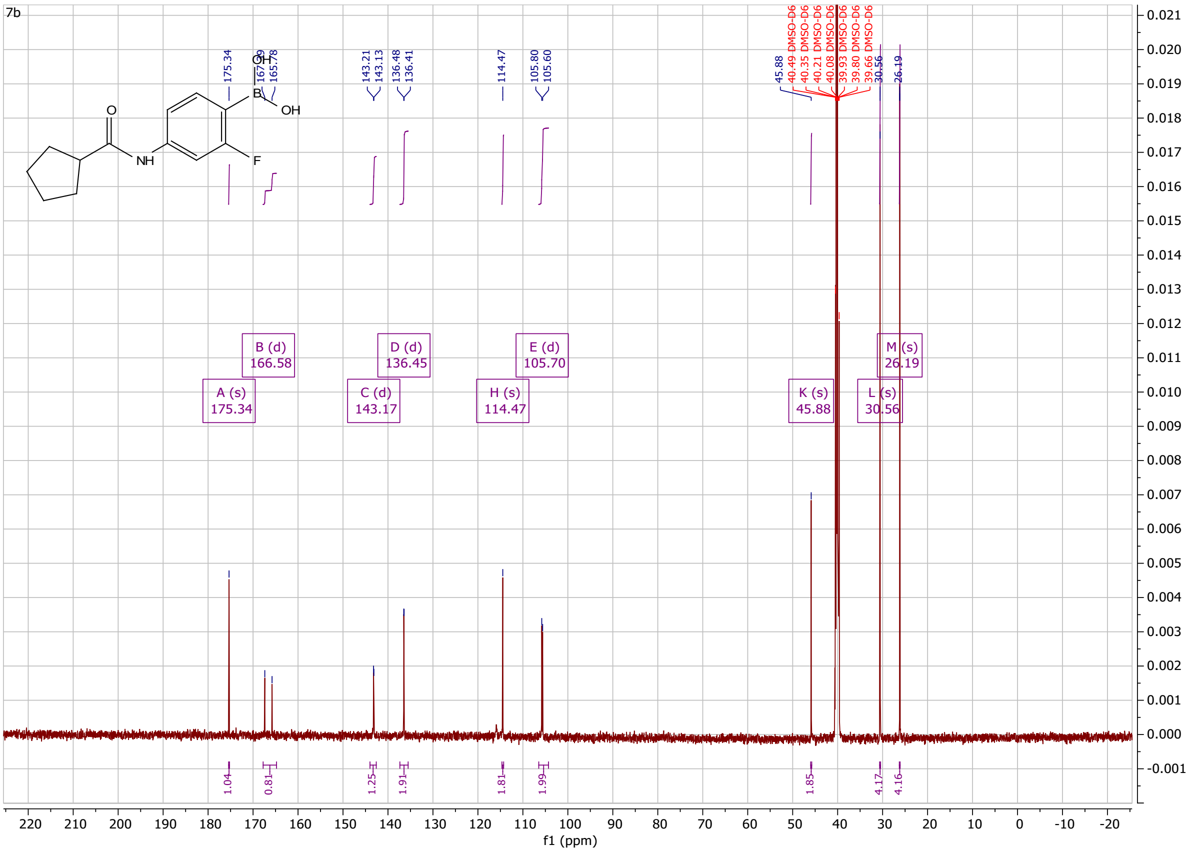

7b ^{11}B NMR spectrum

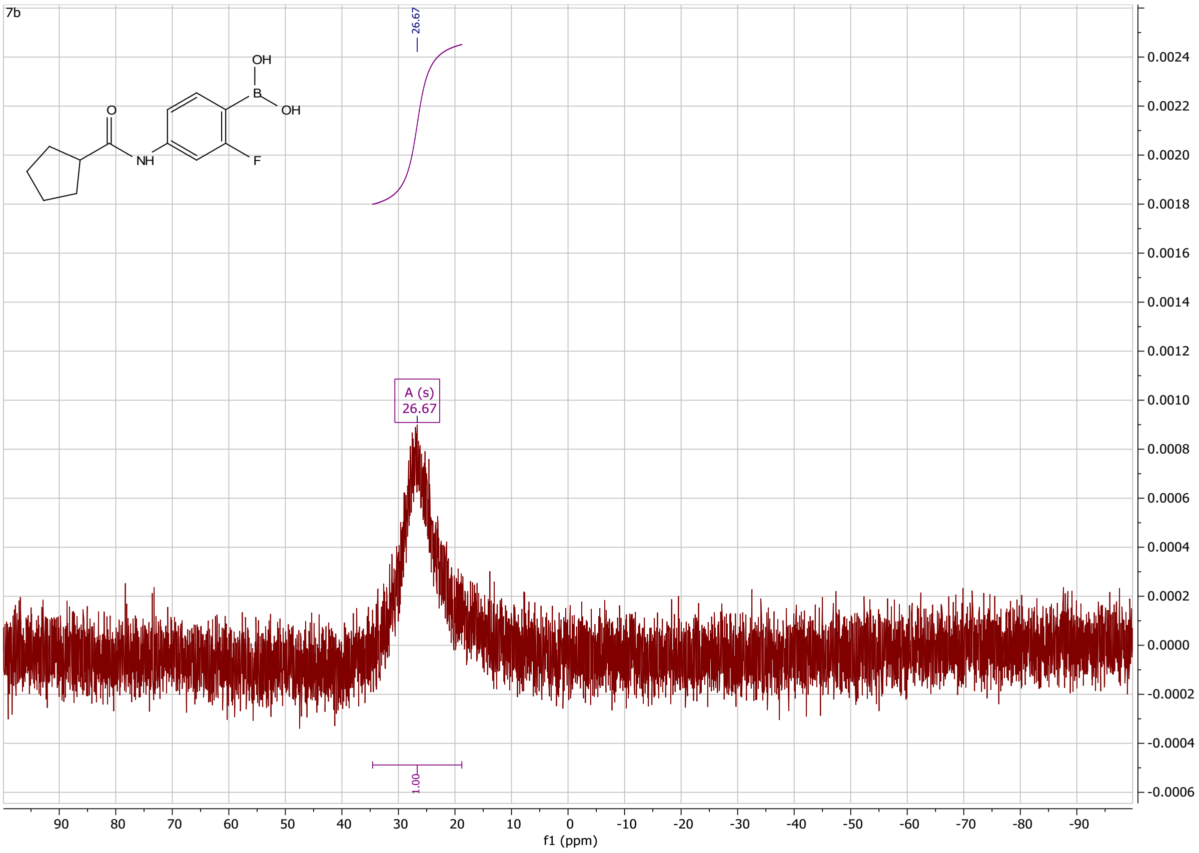

1c ^{1}H NMR spectrum

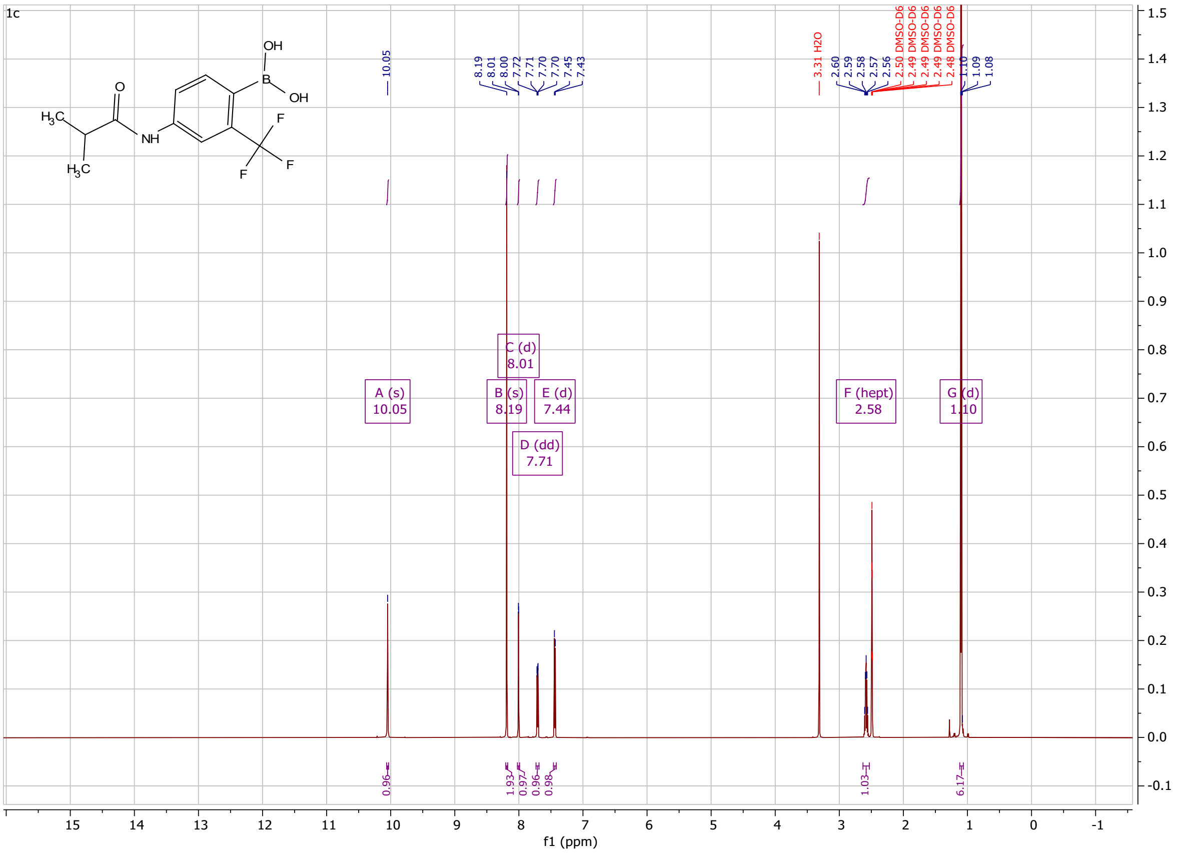

1c ^{13}C NMR spectrum

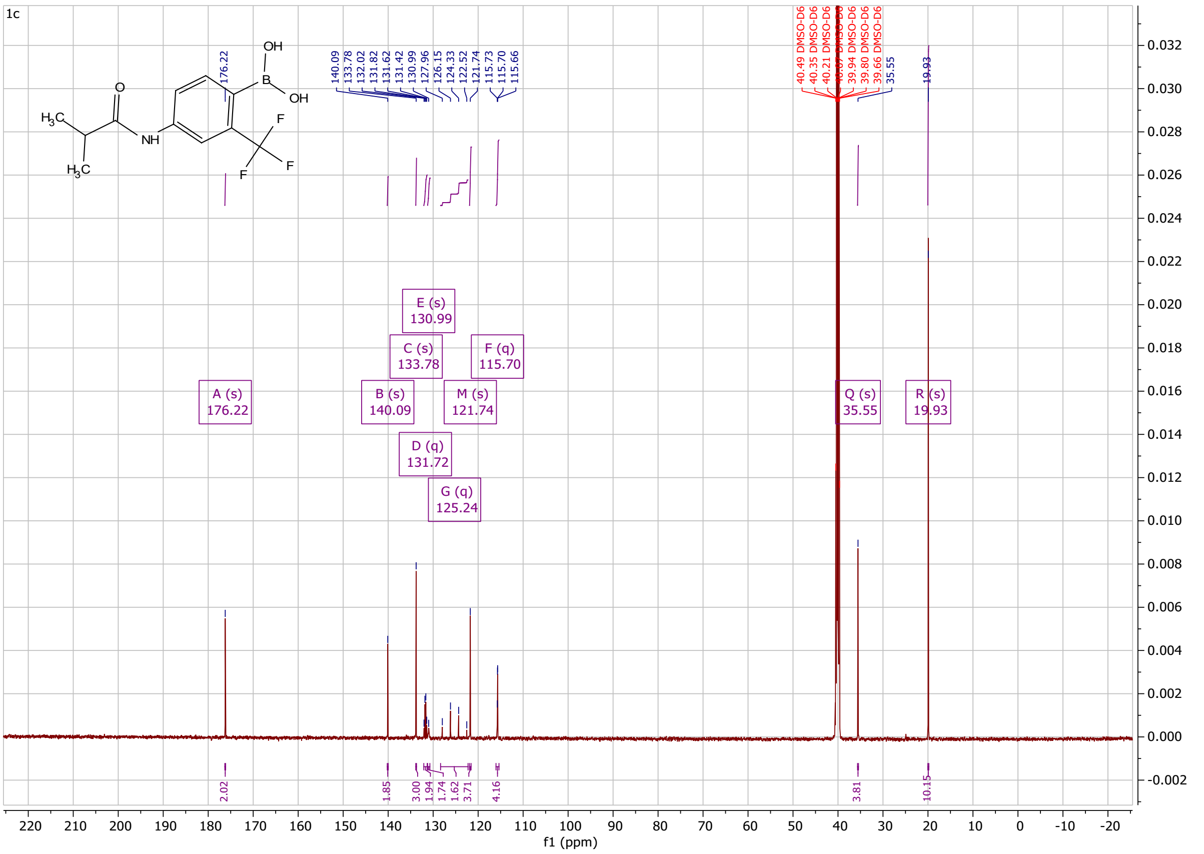

1c ^{11}B NMR spectrum

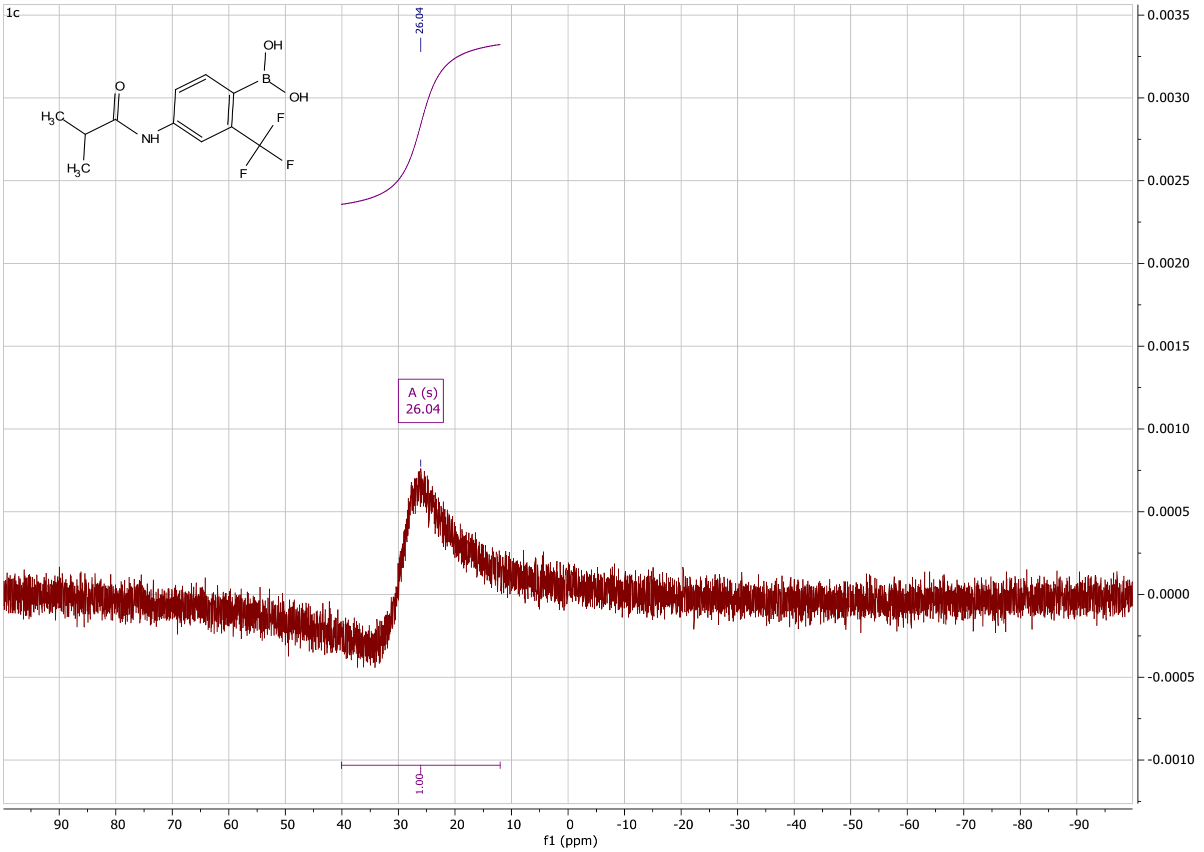

2c ^{1}H NMR spectrum

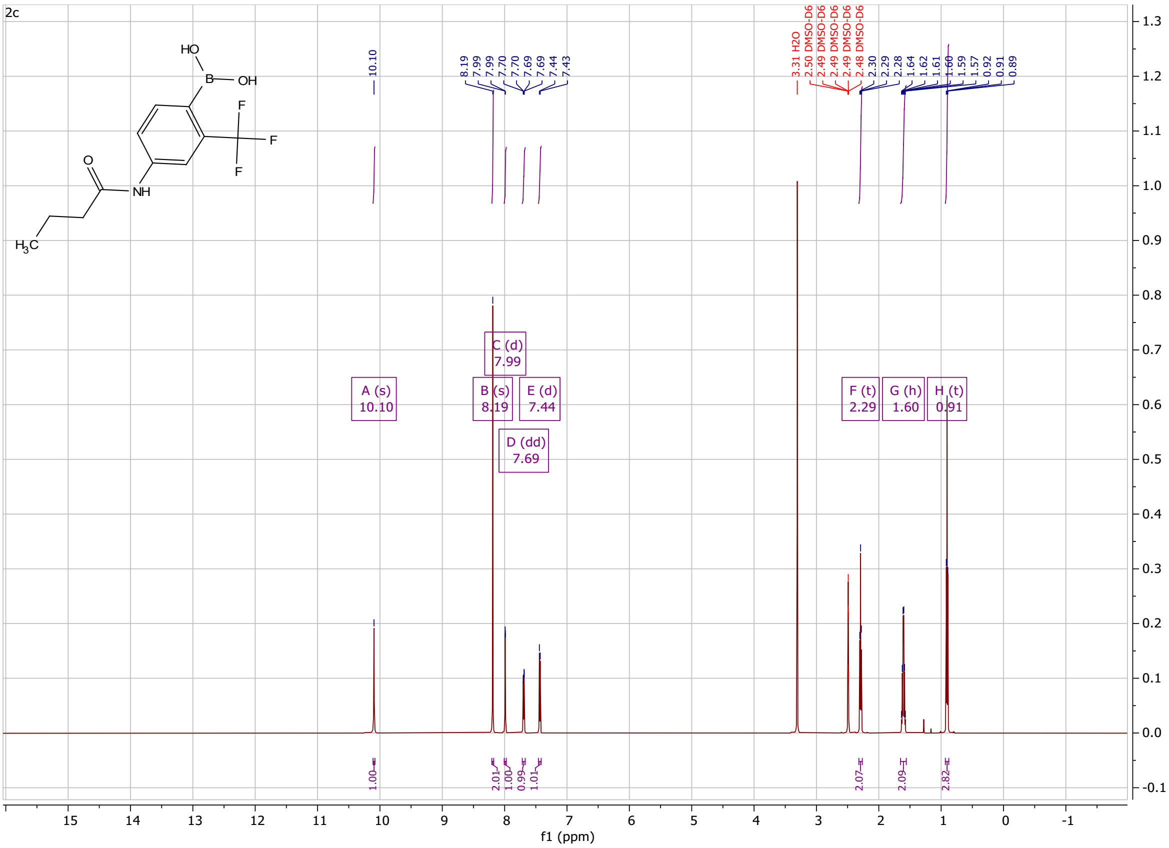

2c ^{13}C NMR spectrum

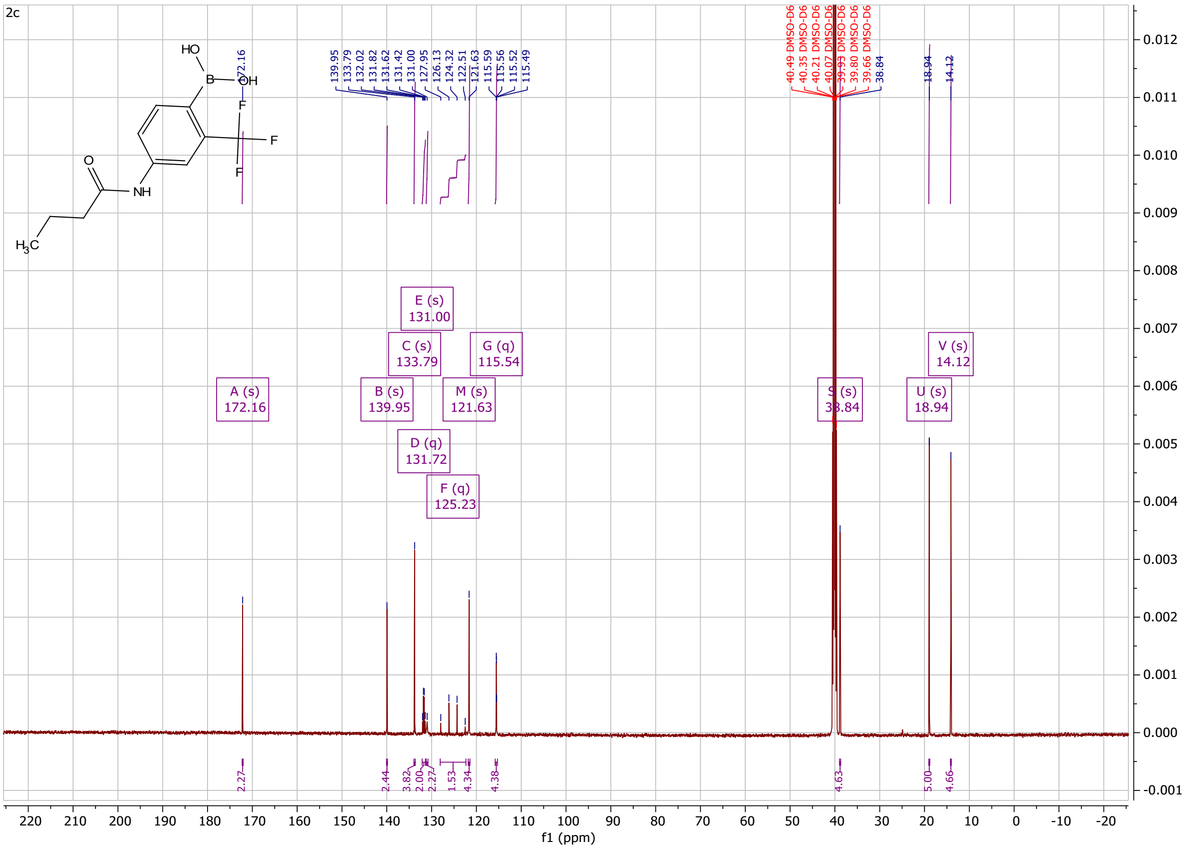

2c ^{11}B NMR spectrum

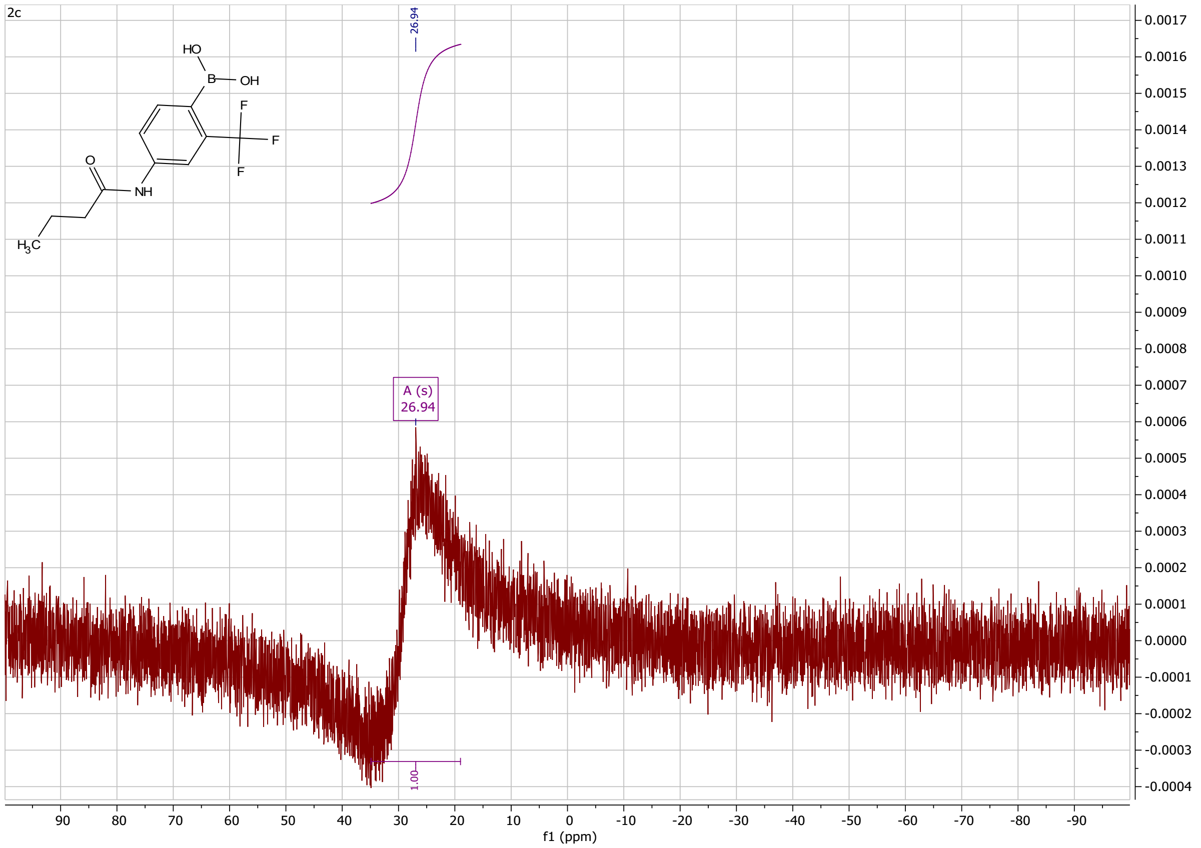

3c ^{1}H NMR spectrum

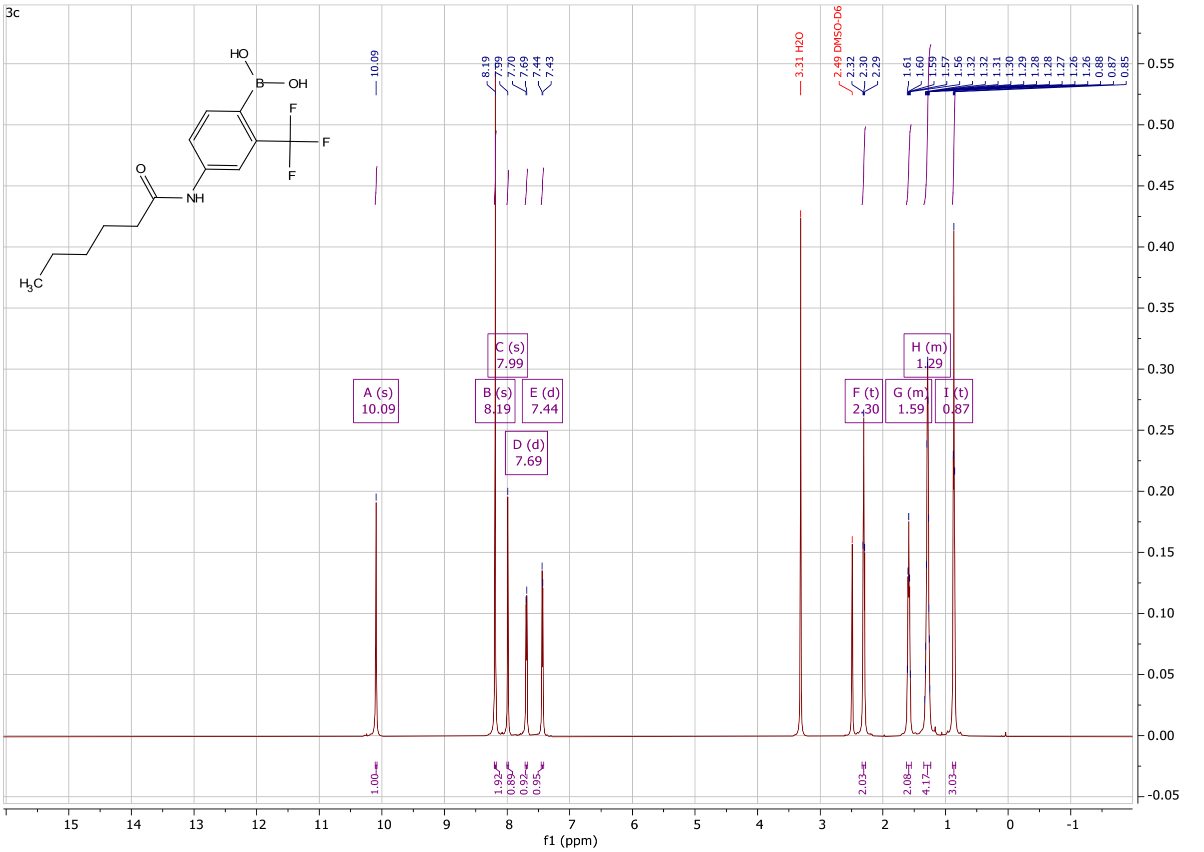

3c ^{13}C NMR spectrum

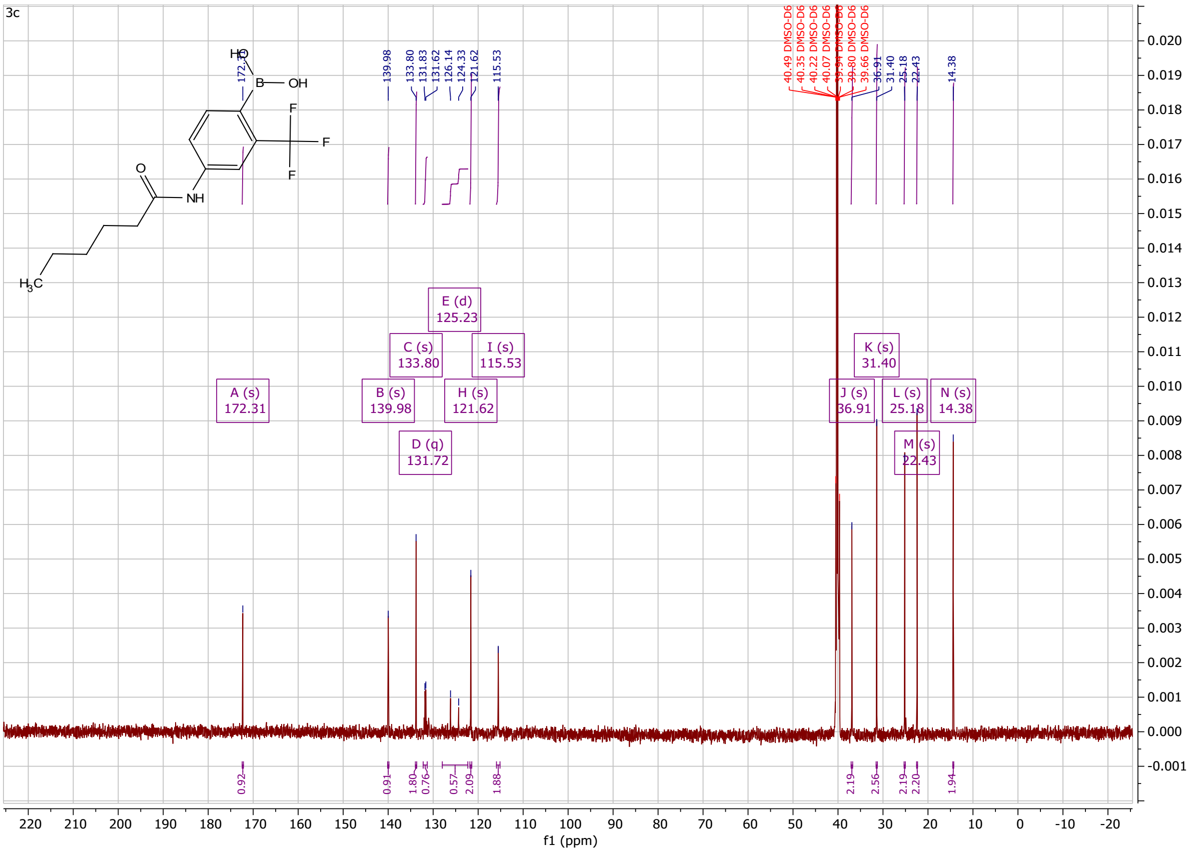

3c ^{11}B NMR spectrum

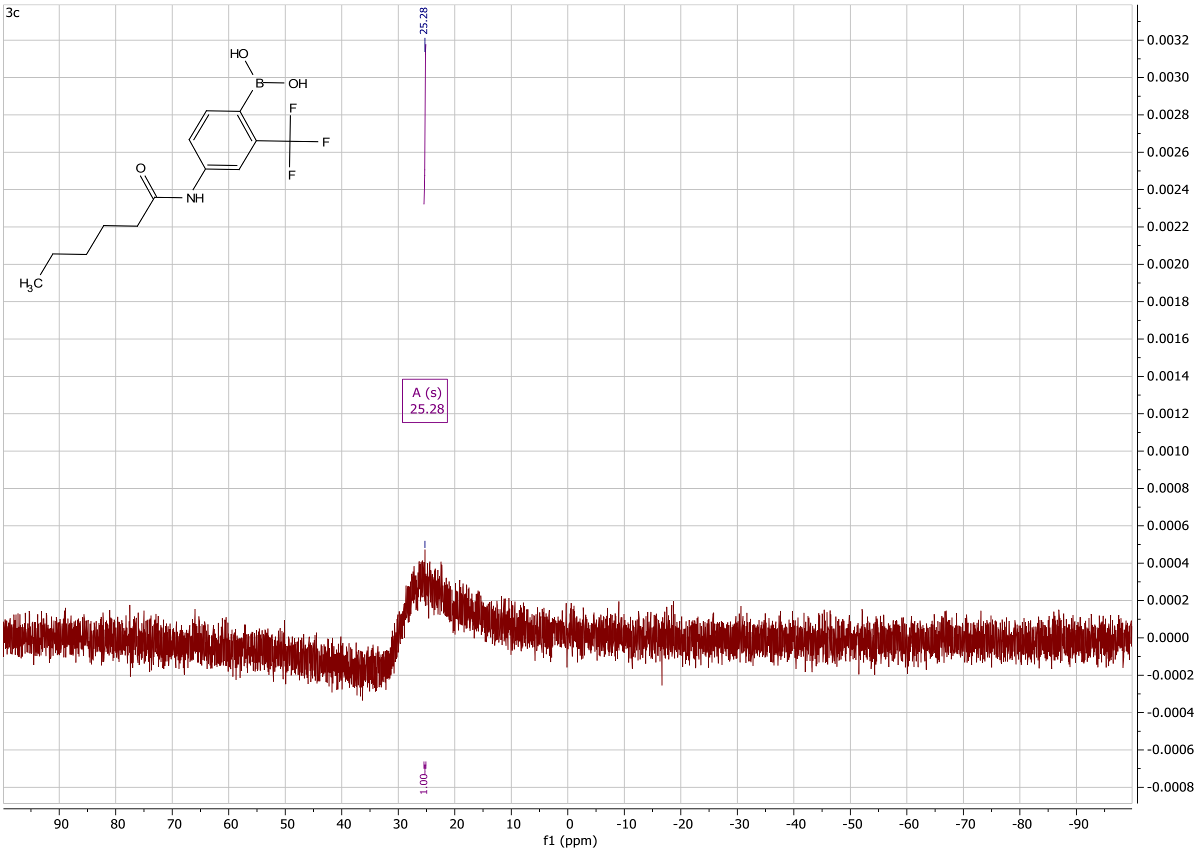

4c ^1H NMR spectrum

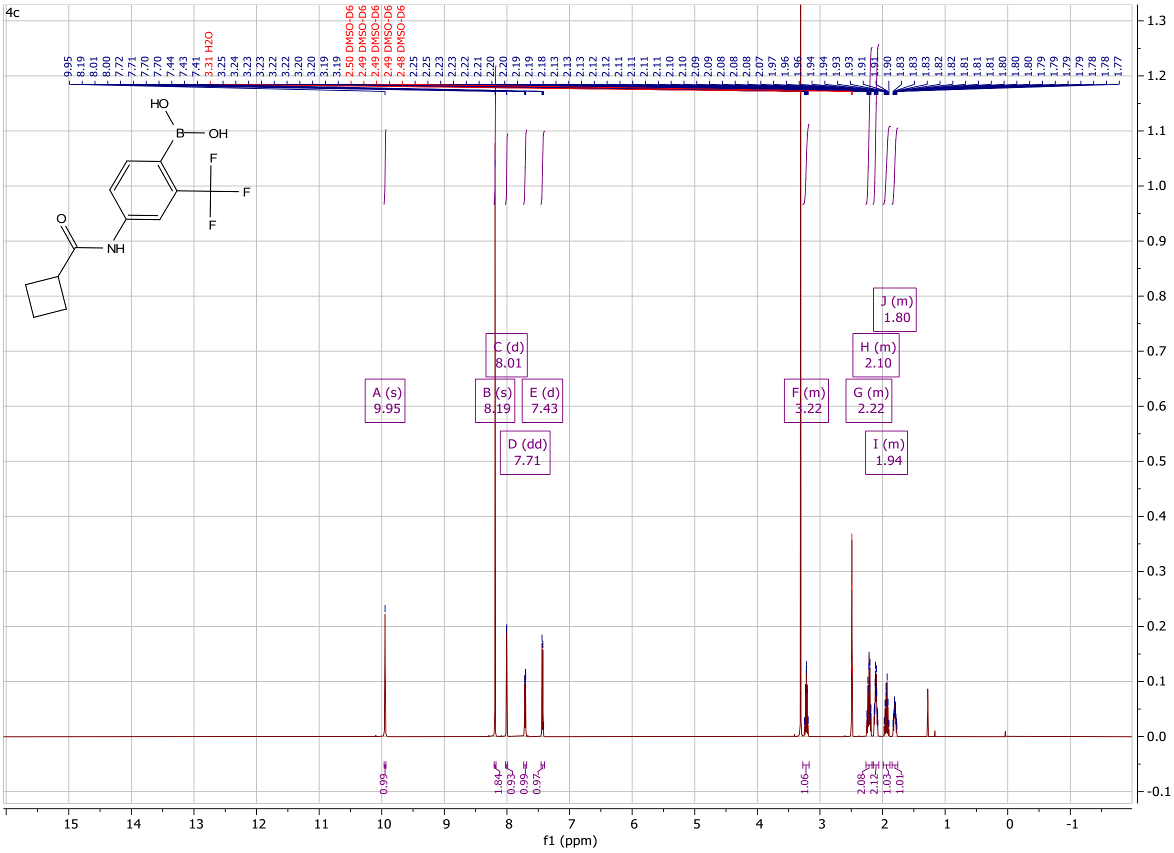

4c ^{13}C NMR spectrum

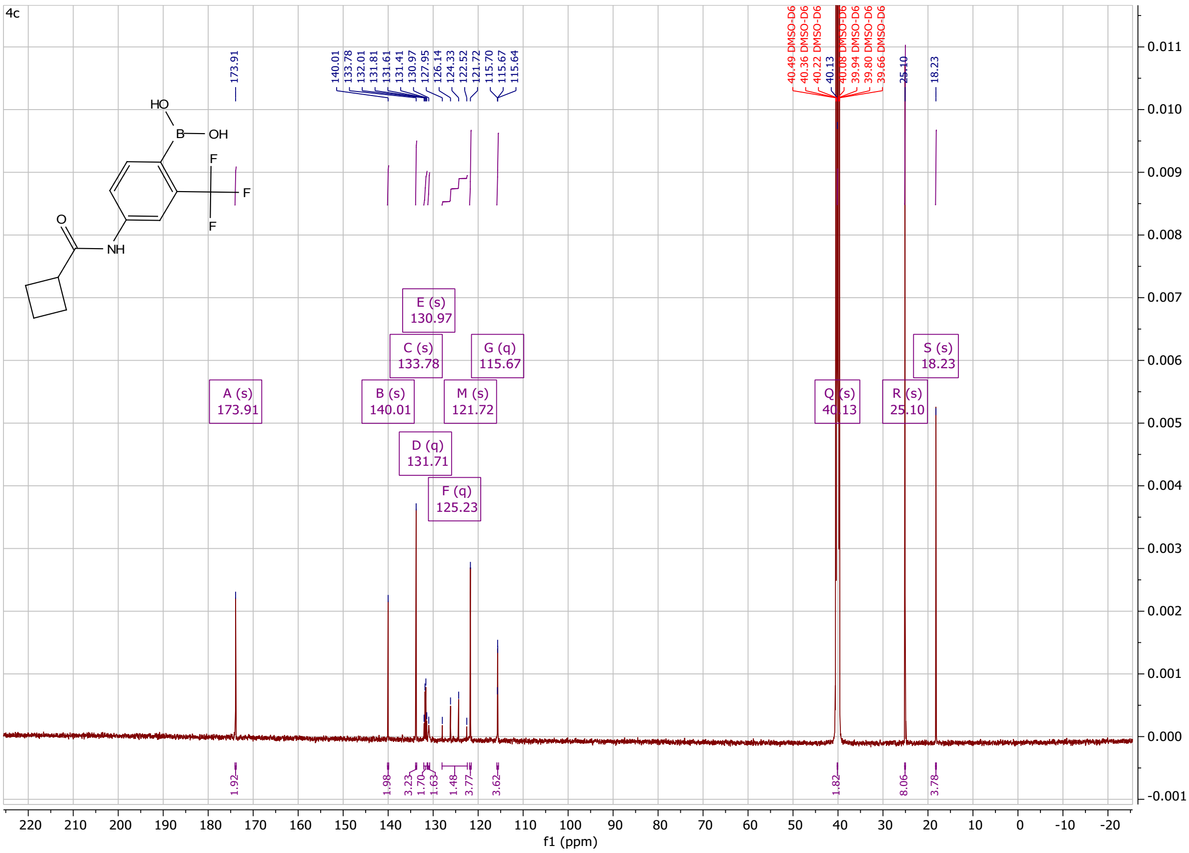

4c ^{11}B NMR spectrum

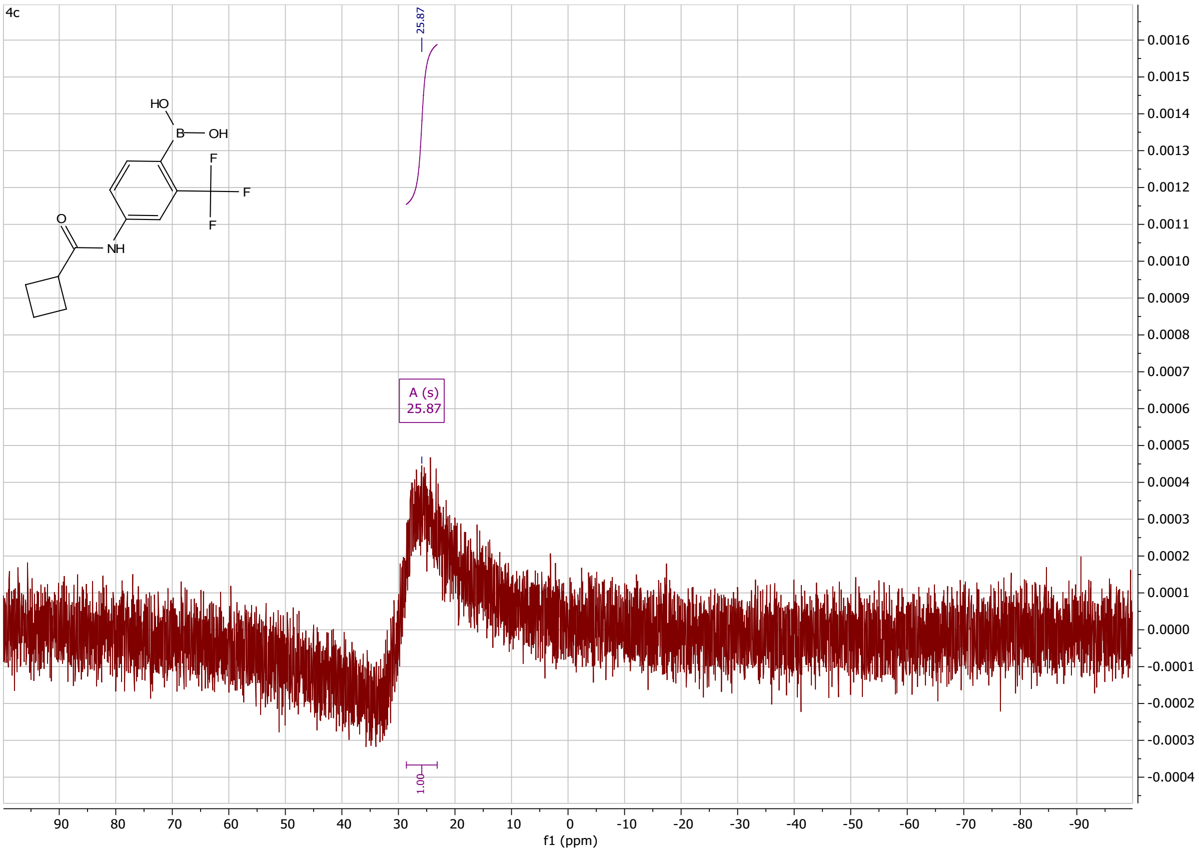

5c ^{1}H NMR spectrum

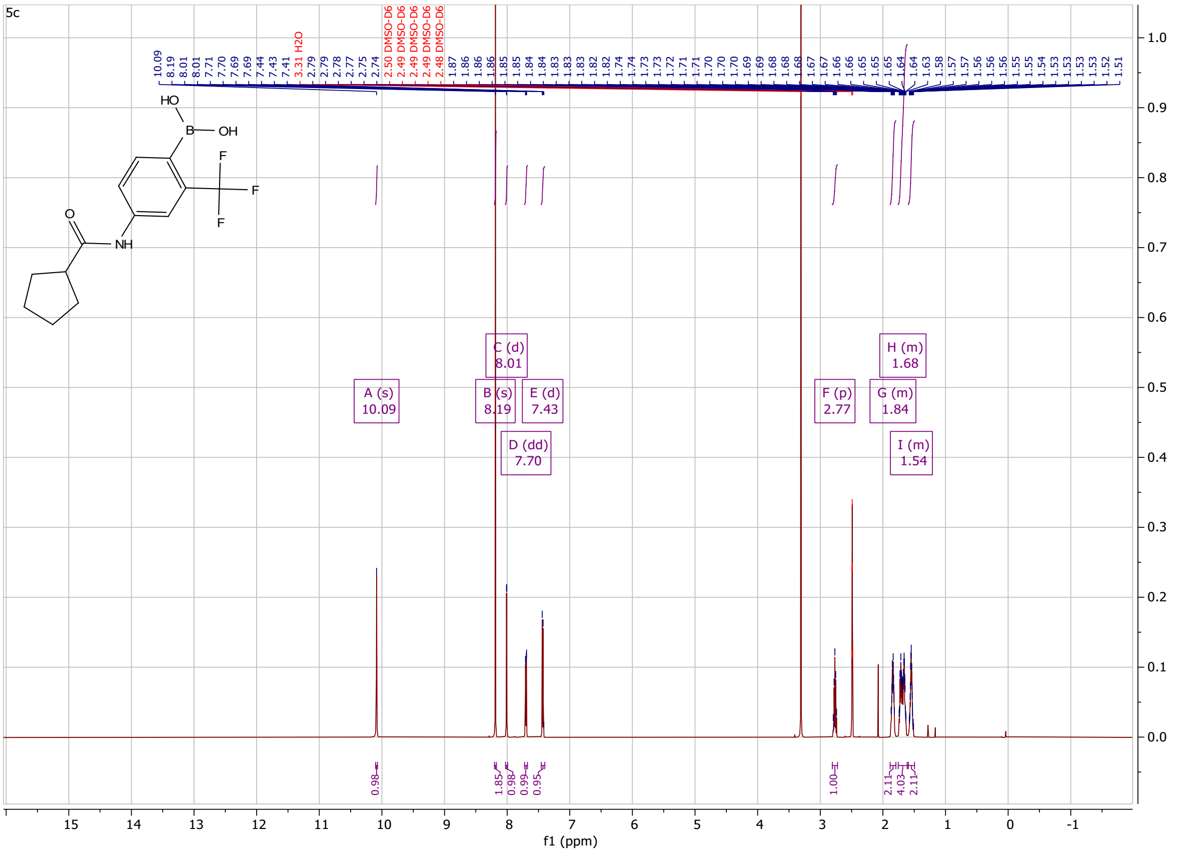

5c ^{13}C NMR spectrum

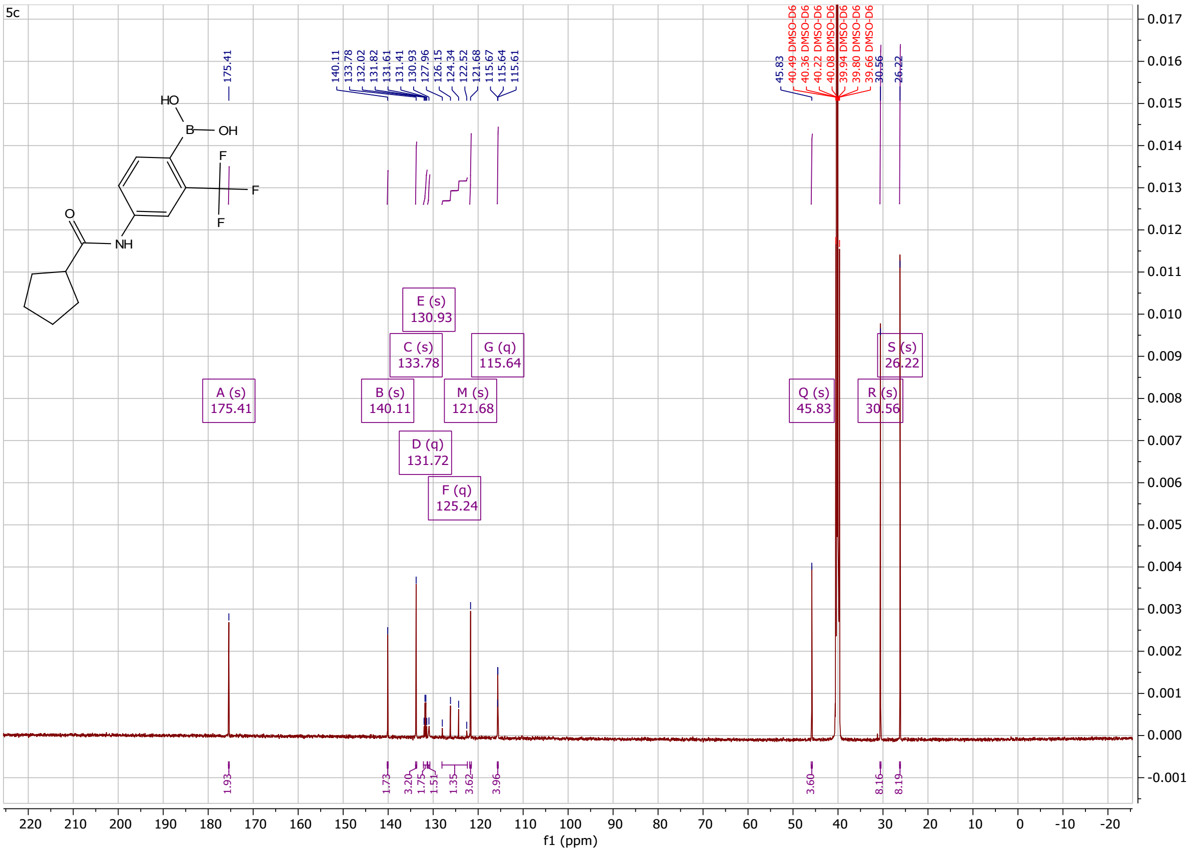

5c ^{11}B NMR spectrum

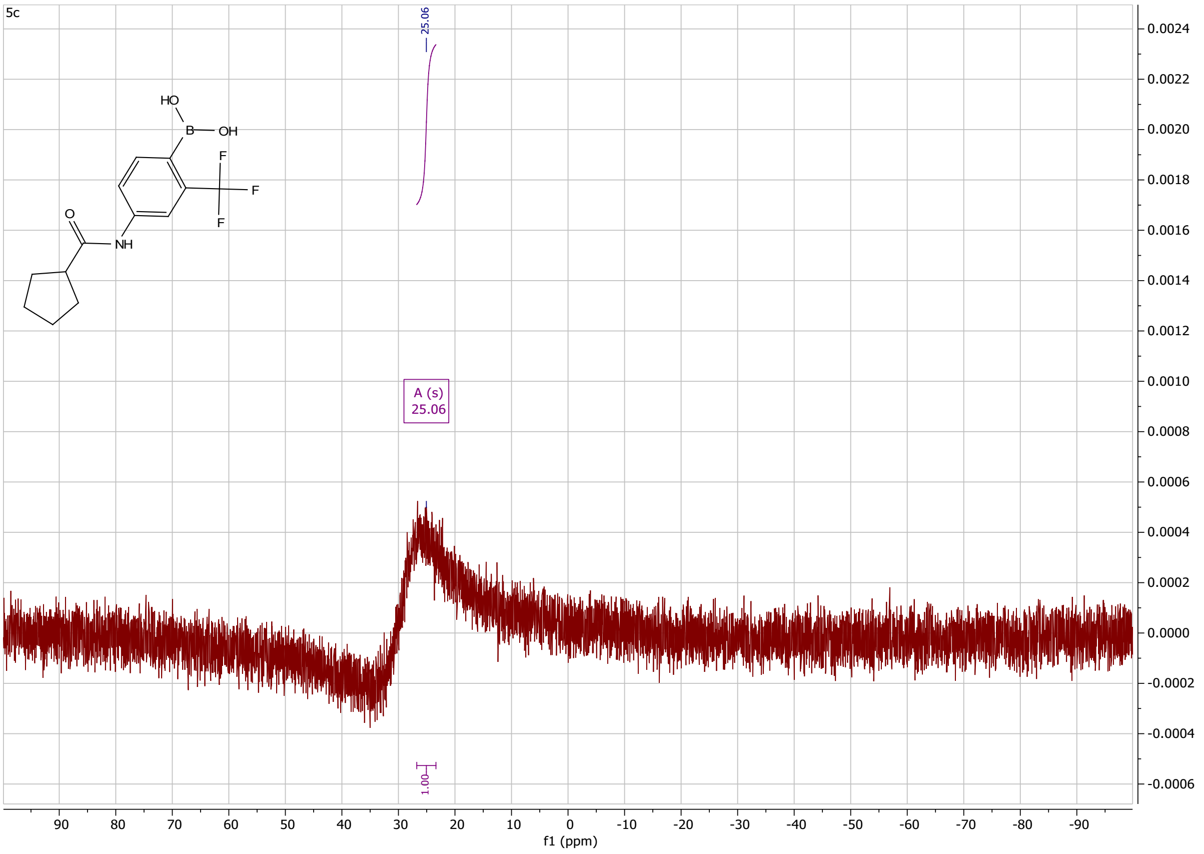

Supplement: MD-015-D4MD00343H-s002 [file MD-015-D4MD00343H-s002.pdf]
